# Discovery of STX-721, a Covalent, Potent, and Highly Mutant-Selective EGFR/HER2 Exon20 Insertion Inhibitor for the Treatment of Non-Small Cell Lung Cancer

**DOI:** 10.1021/acs.jmedchem.4c02377

**Published:** 2025-01-17

**Authors:** Benjamin C. Milgram, Deanna R. Borrelli, Natasja Brooijmans, Jack A. Henderson, Brendan J. Hilbert, Michael R. Huff, Takahiro Ito, Erica L. Jackson, Philip Jonsson, Brendon Ladd, Erin L. O’Hearn, Raymond A. Pagliarini, Simon A. Roberts, Sébastien Ronseaux, Darrin D. Stuart, Weixue Wang, Angel Guzman-Perez

**Affiliations:** † Scorpion Therapeutics, 1 Winthrop Square, Boston, Massachusetts 02110, United States; ‡ Scorpion Therapeutics, South San Francisco, California 94080, United States

## Abstract

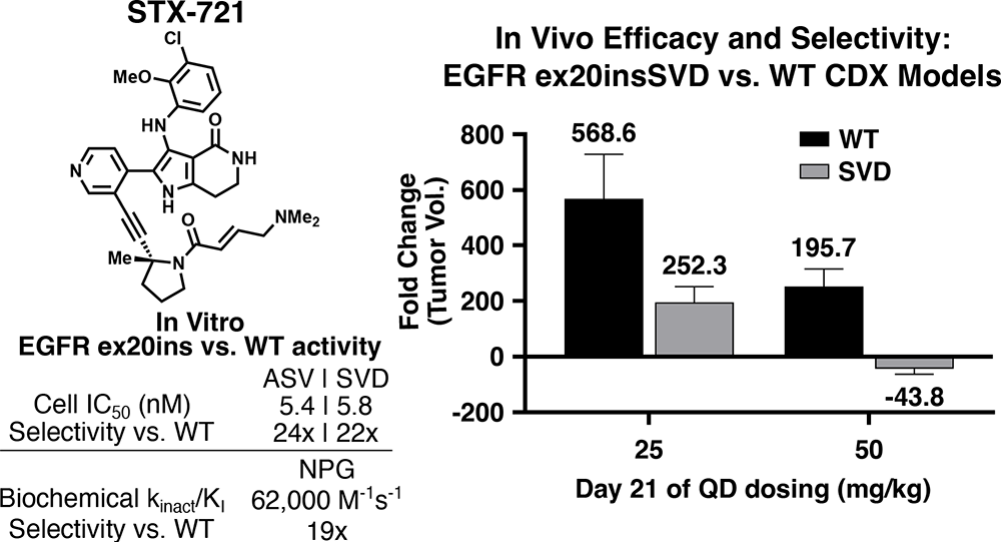

After L858R and ex19del epidermal growth factor receptor (EGFR) mutations, ex20ins mutations are the third most common class of driver-mutations in non-small cell lung cancer (NSCLC). Unfortunately, first-, second-, and third-generation EGFR tyrosine kinase inhibitors (TKIs) are generally ineffective for ex20ins patients due to insufficient mutant activity and selectivity over wild-type EGFR, leading to dose-limiting toxicities. While significant advances in recent years have been made toward identifying potent EGFR ex20ins mutant inhibitors, mutant vs wild-type EGFR selectivity remains a significant challenge. STX-721 (**53**) is a potent, irreversible inhibitor of the majority of EGFR/HER2 ex20ins mutants and demonstrates excellent mutant vs wild-type selectivity both in vitro and in vivo. STX-721 is currently in phase 1/2 clinical trials for EGFR/HER2 ex20ins-driven NSCLC.

## Introduction

Epidermal growth factor receptor (EGFR) is a receptor tyrosine kinase (RTK) and a member of the ERBB subfamily of growth factor receptors (EGFR/ERBB1, HER2/ERBB2, HER3/ERBB3, and HER4/ERBB4).^1^ Ligand binding to the extracellular domain of EGFR promotes formation of homo- and heterodimers, followed by tyrosine autophosphorylation, and activation of several downstream signal transduction pathways in control of cell proliferation, apoptosis, and cell death, among other cellular processes.^2^ Activating mutations in the tyrosine kinase domain of EGFR and HER2 lead to ligand-independent autophosphorylation and ultimately tumorigenesis.^3−5^

Lung cancer constitutes the leading cause of cancer-related deaths worldwide (18%) and non-small cell lung cancer (NSCLC) represents the majority of lung cancer cases (85%).^6,7^ Oncogenic mutations of the EGFR kinase domain are the most common activating driver mutations in lung adenocarcinomas.^8^ After in-frame deletions of exon 19 (ex19del) and single point mutations at codon 858 in exon 21 (L858R), insertion mutations within exon 20 (ex20ins) are the third most prevalent mutations of EGFR (4–10%).^4,9−11^

**Figure 1:**
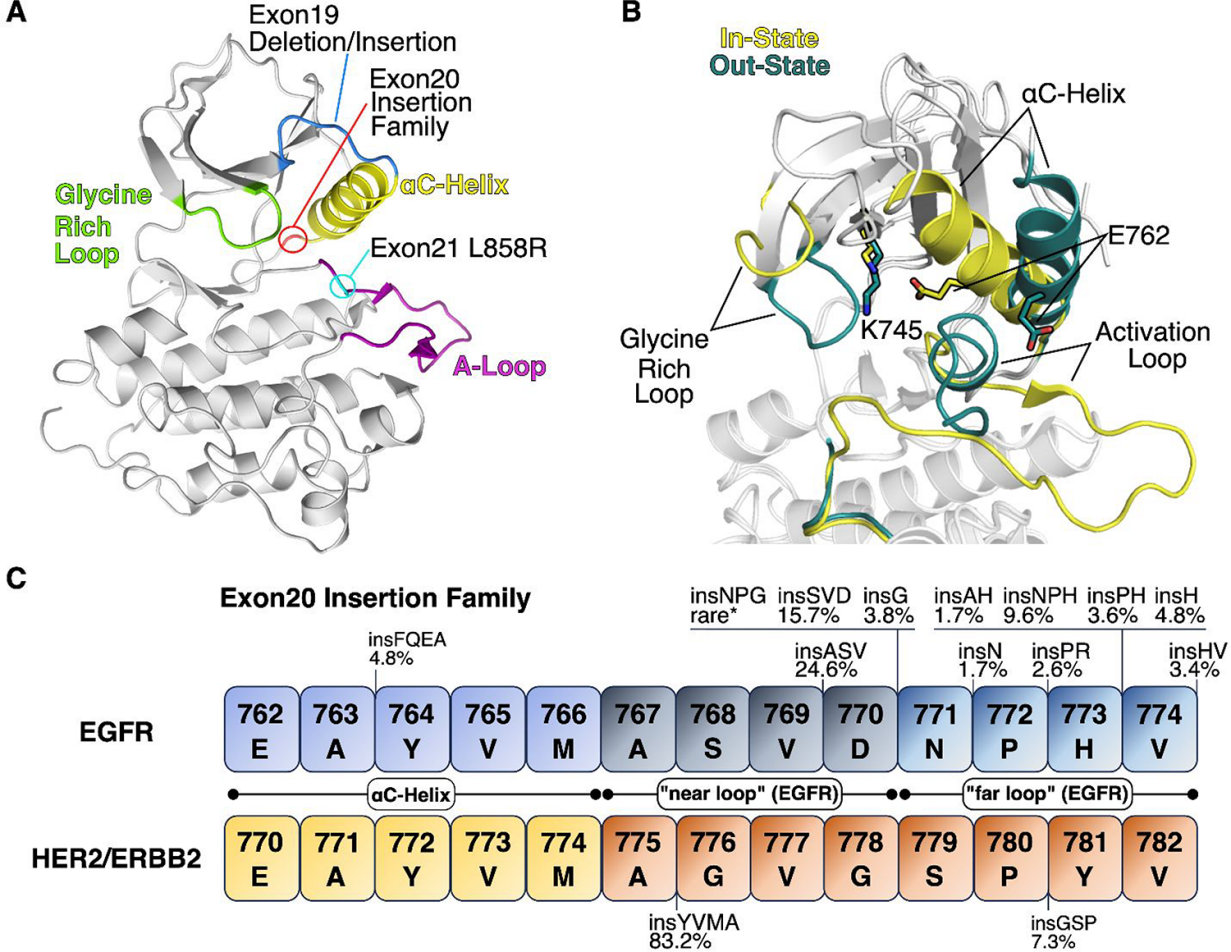
(A) Crystal structure of the WT-EGFR tyrosine kinase domain αC-helix in conformation (PDB 2GS2) with key regions and mutant locations highlighted. (B) Overlay of the N-lobe region comparing in-state (yellow; PDB 2GS2) and out-state (teal; PDB 1XKK with ligand removed) conformations. (C) Position and frequency of EGFR exon20 insertion mutants in non-small cell lung cancer. Mutational frequency distribution data from v99 of COSMIC, the Catalogue Of Somatic Mutations In Cancer (*n* = 415 for EGFR and 220 for ERBB2/HER2).^15,17^

EGFR ex20ins mutations constitute a diverse set of in-frame insertion or duplication mutations of 1–7 amino acids located between positions 762 and 774 of the EGFR kinase domain (Figure 1C).^12^ Ex20ins mutations primarily occur at the C-terminal end of the αC-helix (Figure 1A). Similar insertion mutations exist in the homologous HER2/ERBB2 protein.^13^ Over 80 different EGFR ex20ins mutants have been reported, the location and prevalence of the most common variants are summarized in Figure 1C.^14,15^ The heterogeneity of ex20ins mutations makes the design of an EGFR mutant-selective inhibitor particularly challenging. Mechanistically, it is hypothesized that ex20ins mutations “push” and “lock” the αC-helix of the kinase into an in-state (Figure 1B) active conformation that promotes ligand-independent EGFR dimerization, receptor activation, the constitutive activation of downstream signaling pathways, and oncogenesis.^12,16^

Osimertinib, an ATP-competitive third-generation EGFR tyrosine kinase inhibitor (TKI), offers clear patient benefit in first- and second-line settings for ex19del and L858R mutant-EGFR positive NSCLC patients with or without the secondary gatekeeper residue (T790M) mutation.^18−20^ Importantly, osimertinib provides an improved therapeutic window over wild-type (WT) EGFR related toxicity compared to prior first- and second-generation EGFR TKIs due to its enhanced mutant selectivity.^21−24^ Unfortunately, ex20ins mutant-EGFR positive NSCLC patients show poor response rates to first-, second-, and third-generation EGFR TKIs, including osimertinib, likely due to their suboptimal ex20ins mutant activity and selectivity vs WT-EGFR.^25−27^ While significant efforts have been focused on developing potent EGFR ex20ins mutant inhibitors, mutant selectivity remains a significant challenge.^28,29^

**Figure 2:**
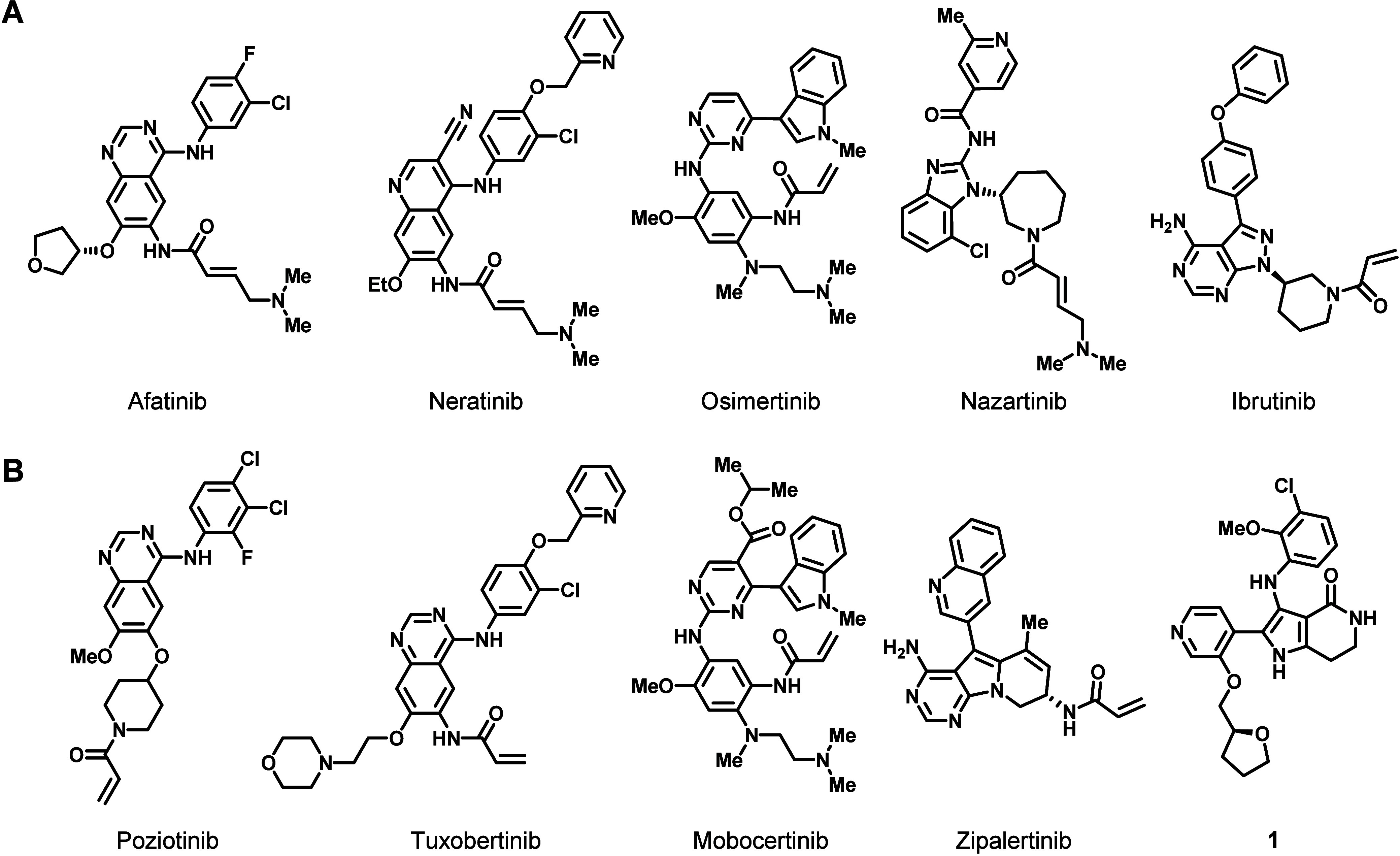
(A) Selected FDA-approved tyrosine kinase inhibitors with reported EGFR exon20 insertion mutant activity. (B) Selected examples of published EGFR exon20 insertion mutant-selective inhibitors.

We began our efforts to identify EGFR ex20ins mutant-selective chemical matter by screening a commercially available library of TKIs, including several clinical second- and third-generation EGFR inhibitors with reported EGFR ex20ins activity (Figure 2A).^30−34^ This screening collection was supplemented with clinical compounds designed to target EGFR ex20ins and compounds from the literature with reported ex20ins mutant selectivity (Figure 2B).^35−39^ Antiproliferative activity and selectivity were measured in isogenic Ba/F3 engineered cell lines overexpressing EGFR exon20 D770_N771 insNPG (insNPG) mutant- and WT-EGFR, in an effort to identify medicinal chemistry starting points with inherent selectivity. Isogenic Ba/F3 EGFR WT cells were chosen to profile selectivity because they require EGF ligand to activate the receptor, as in normal tissues, to drive proliferation. Through this screening effort we were able to identify a chemical series from the patent literature whose structure is exemplified by **1** that exhibited the highest level of mutant vs WT selectivity of the compounds tested (≥17-fold) (Figure 2 and Table 1) and whose ex20insNPG mutant selectivity approached the level observed for osimertinib toward its intended target EGFR L858R/T790M (29-fold; see Supporting Information, Table S8, for osimertinib Ba/F3 cell proliferation data and corresponding statistics).^38^

**Table 1:**
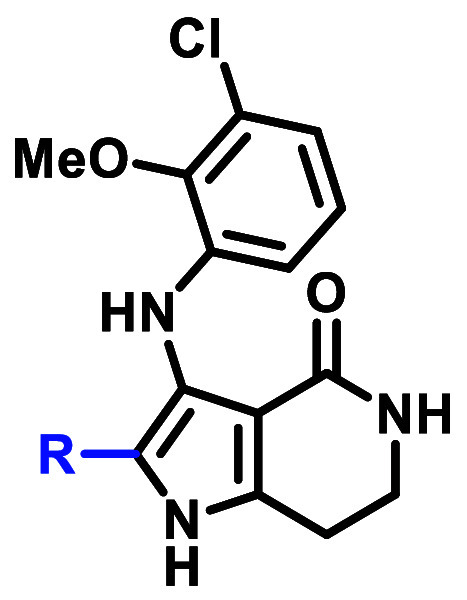

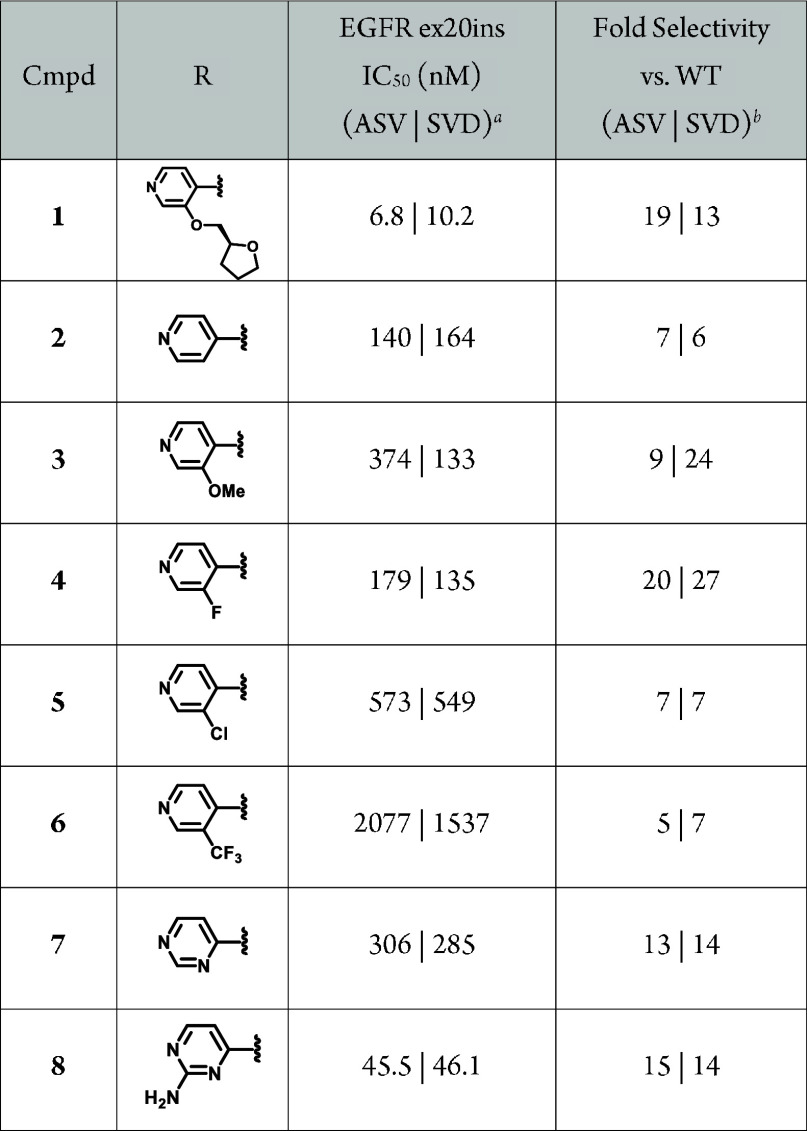
Structure–Activity Relationship of Hinge Binder Analogues of Compound **1**

a Antiproliferative activity in Ba/F3 via CellTiter-Glo.

b Fold selectivity against Ba/F3 EGFR WT. All data represents *n* ≥ 2. See Supporting Information, Tables S4–S6, for all IC_50_ statistical analyses.

## Results and Discussion

**Figure 3:**
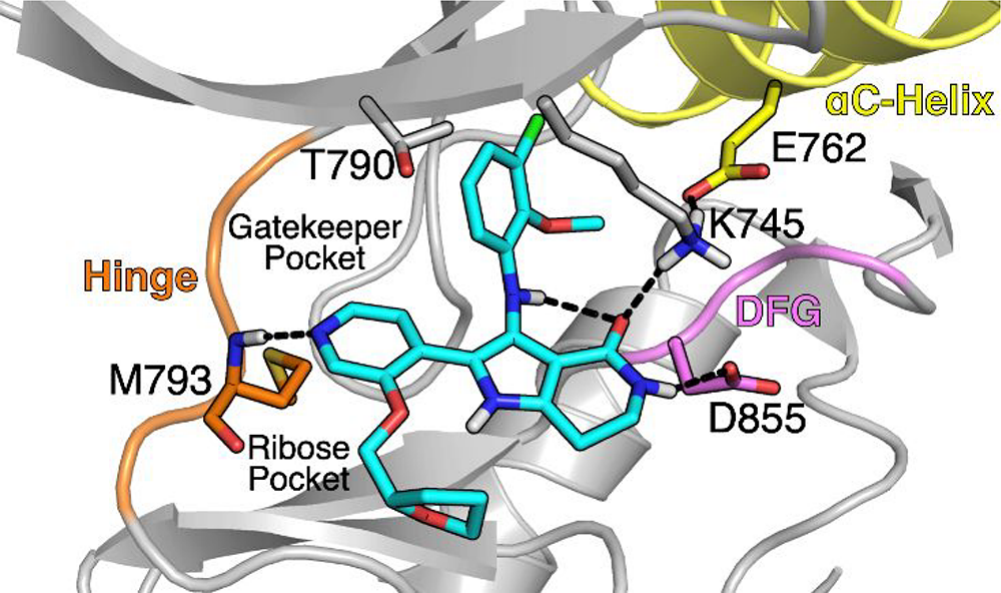
WT-EGFR XRC structure (PDB 1M17) was used to generate the docking model with **1** (see Supporting Information for docking protocol).

Medicinal chemistry efforts commenced with the goal to identify the key interactions for EGFR ex20ins activity and selectivity over WT-EGFR within the chemical matter represented by **1**. At the outset of our work, a sufficiently high-resolution EGFR ex20ins X-ray crystal (XRC) structure was not available for binding pose prediction.^40^ Given the high sequence homology between the EGFR ex20ins and WT catalytic active sites of the tyrosine kinase domain and the appreciable EGFR WT activity of **1**, Glide docking was used to generate binding poses for **1** using an αC-helix-in WT-EGFR XRC structure (Figure 3). The docking model for **1** suggested several key interactions that were envisioned to be critical to maintain activity: (1) a one-point hydrogen bond interaction between the pyridyl N and the hinge Met793, (2) a hydrogen bond network between the lactam moiety, the catalytic Lys745, and DFG Asp855, and (3) an intramolecular hydrogen bond network between the aniline N–H and the lactam carbonyl. Beyond these putative interactions, we sought to identify the minimum pharmacophore for ex20ins potency and selectivity over WT-EGFR.

We began these efforts by exploring the pyridyl hinge binder portion of the molecule through varying the substituent at the pyridyl 3-position. At this stage in the program, Ba/F3 cell lines for the clinically prevalent EGFR exon20 A769_V770 insASV (insASV) and EGFR exon20 D770_N771 insSVD (insSVD) mutants were established. Consequently, we pivoted from profiling insNPG to insASV and insSVD as representatives for investigating the EGFR ex20ins mutant activity and selectivity vs WT-EGFR structure–activity relationship (SAR) due to their higher clinical relevance.^15^ Modification of the pyridyl 3-position moiety led to a dramatic reduction in activity across both insASV and insSVD mutants (Table 1). Pyridyl 3-H analogue **2** lost both mutant activity and selectivity vs WT-EGFR, while 3-OMe **3**, lost a similar level of activity, but retained insSVD mutant-selectivity. Interestingly, as the steric bulk of the pyridyl 3-position substituent grew from −F (**4**) to −Cl (**5**) to −CF_3_ (**6**), a precipitous loss in mutant activity and selectivity vs WT-EGFR was observed. These data suggested 3-position groups such as −OMe and −F, capable of participating in an intramolecular hydrogen bond with the pyrrole N–H, promoted a preferred pseudocoplanar orientation between the pyridine hinge binder and pyrrololactam core. Accordingly, pyrimidine **7** and 2-aminopyrimidine **8** were prepared, with **8** demonstrating superior mutant activity and ∼15-fold selectivity vs WT-EGFR.

Having established preliminary hinge binder SAR, we turned our attention to modifying the aniline portion of the molecule in the context of the potent 2-aminopyridine hinge binder (Table 2). Removal of the aromatic substituents from **8** to give **9** led to a ∼10-fold loss in mutant activity and interestingly a complete loss of selectivity over WT-EGFR. Next, a chlorine walk revealed substitution of the 3-position found in **11** provided the best mutant activity. Optimization of the 3-position substituent was explored in the context of a fixed 2-OMe group. While 3-F analogue **13** lost only 3-fold in mutant activity, it retained selectivity. 3-Me and 3-CF_3_ analogues **14** and **15** led to a more substantial loss in mutant activity. The 2-position was then explored in the context of a fixed 3-Cl group. 2-F and 2-Me analogues **16** and **17** were modestly less potent than **8** but showed a pronounced loss in selectivity. Interestingly, 2-Et analogue **18** was reasonably tolerated in terms of potency and selectivity, but 2-OEt analogue **19** showed a significant loss in both end points. Overall, the initial 2-OMe, 3-Cl substitution pattern found in **8** provided the optimal balance of mutant activity and selectivity vs WT-EGFR and was consequently retained for the majority of subsequent analogues.

**Table 2:**
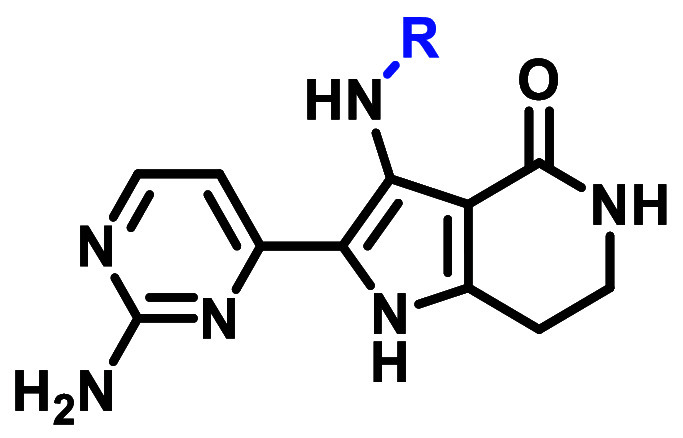

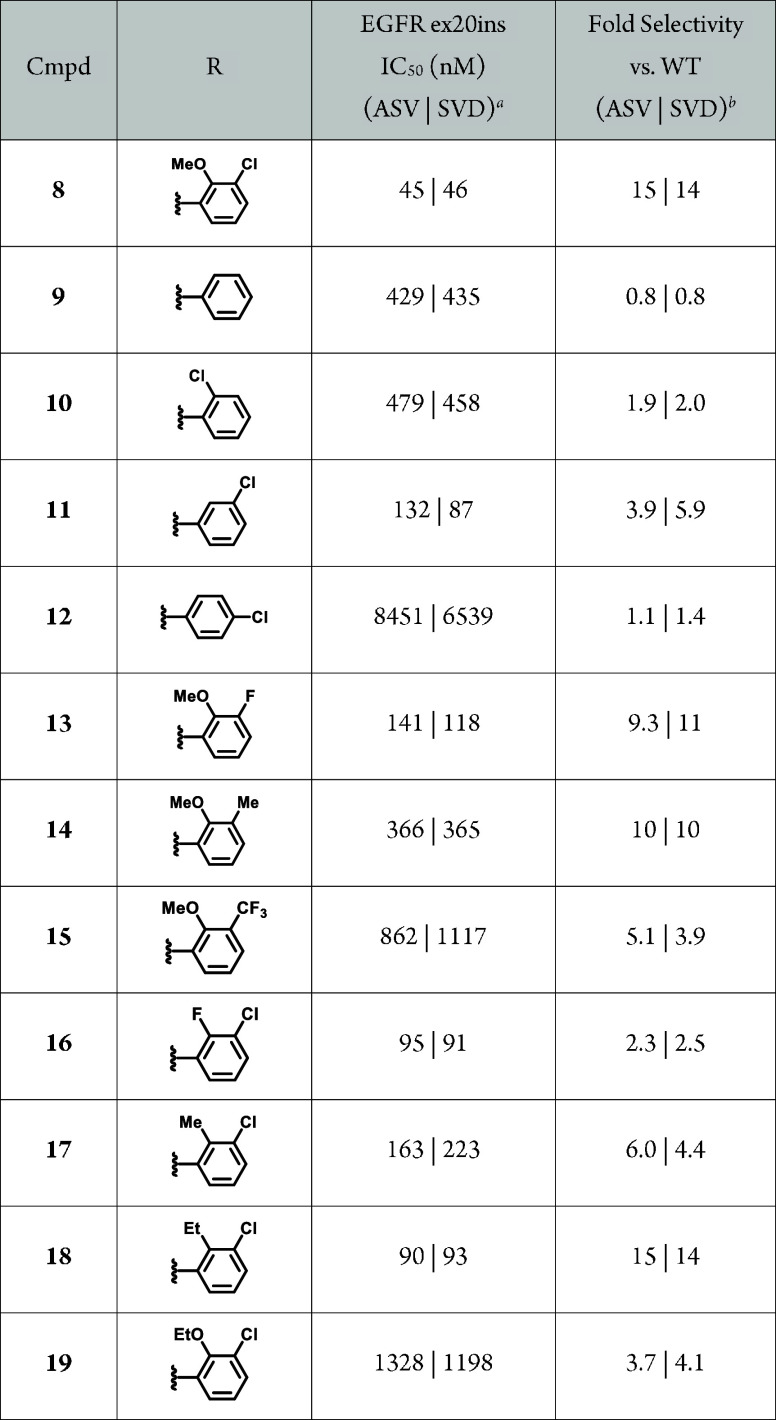
Structure–Activity Relationship of Aniline Analogues of Compound **8**

a Antiproliferative activity in Ba/F3 via CellTiter-Glo.

b Fold selectivity against Ba/F3 EGFR WT. All data represents *n* ≥ 2. See Supporting Information, Tables S4–S6, for all IC_50_ statistical analyses.

While the aniline SAR was explored in the context of the mutant potent 2-aminopyridine hinge binder, the 3-F pyridine hinge binder analogue **4** (Table 1) had the best combination of EGFR ex20ins mutant activity and selectivity vs WT-EGFR and hence represented a suitable minimum pharmacophore on which to continue to explore the SAR. We were interested to investigate if mutant potency could be improved by building into the ribose pocket, previously occupied by the tetrahydrofuran, from a novel vector, via substitution on the pyrrololactam core rather than the pyridine hinge binder (Table 3). Specifically, we explored filling the solvent-facing ribose pocket with a simple methyl ether by varying the stereochemistry about the lactam and the carbon-linker length. Gratifyingly, we observed ethyl methoxy **23** exhibited a 21- to 35-fold improvement in ex20ins potency relative to **4** with ∼15-fold selectivity over WT-EGFR.

**Table 3:**
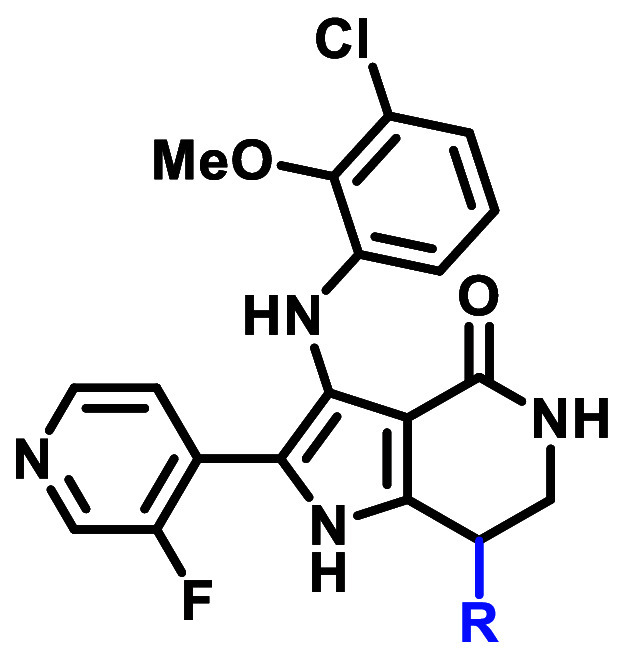

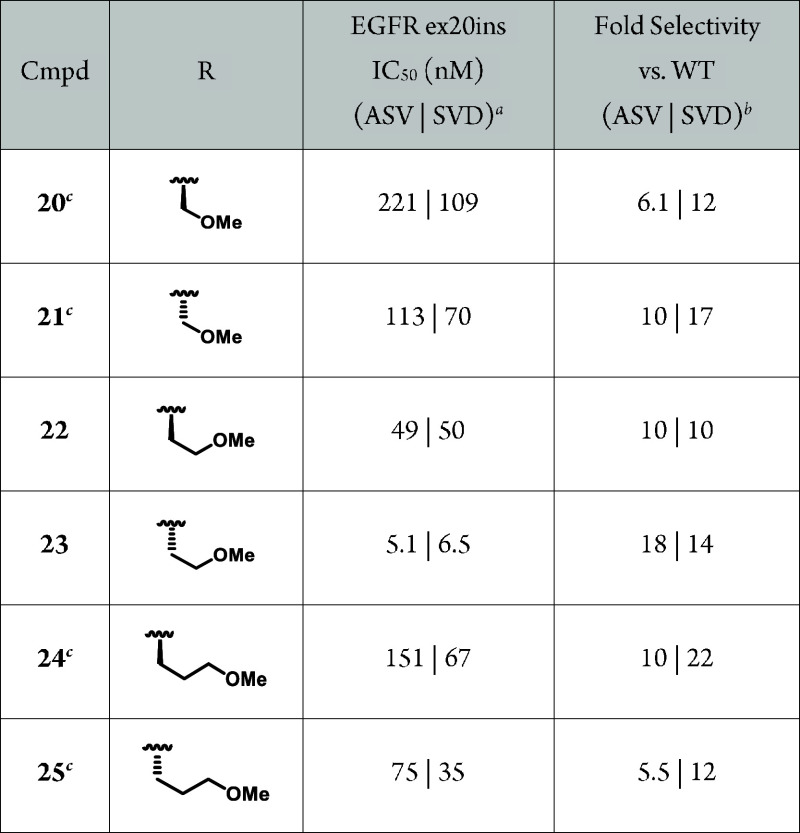
Structure–Activity Relationship of Pyrrololactam Core Substitution Analogues of Compound **4**

a Antiproliferative activity in Ba/F3 via CellTiter-Glo.

b Fold selectivity against Ba/F3 EGFR WT.

c Absolute stereochemistry arbitrarily assigned. All data represents *n* ≥ 2. See Supporting Information, Tables S4–S6, for all IC_50_ statistical analyses.

Compound **23** was then tested in a panel of EGFR ex20ins mutants (Table 4). Potent antiproliferation activity and favorable selectivity vs WT-EGFR was observed across most of the near-loop insertion mutants (insFQEA, insASV, insSVD, insNPG, and insN) and several far-loop insertion mutants (insPH, insNPH, insAH, and insHV), however a notable lack in activity for the less common far-loop insH was observed. Interestingly, **23** demonstrated exquisite potency and selectivity on several classical EGFR mutants (L858R and del19) and exhibited good potency and selectivity for the most common HER2 ex20ins mutant (insYVMA).

**Table 4:** Antiproliferative Activity and Selectivity of Compound **23**

Mutation	Mean IC_50_ (nM)^a^	EGFR WT fold selectivity^b^
EGFR WT	94	N/A
EGFR A763_V764 insFQEA	9.6	10
EGFR V769_D770 insASV	5.1	18
EGFR D770_N771 insSVD	6.5	15
EGFR D770_N771 insNPG	5.4	17
EGFR N771_P772 insN	3.2	29
EGFR H773_V774 insPH	2.8	34
EGFR H773_V774 insNPH	8.7	11
EGFR H773_V774 insAH	12	7.8
EGFR H773_V774 insH	689	0.1
EGFR V774_C775 insHV	25	3.8
EGFR L858R	0.24	392
EGFR del19	0.10	940
HER2 A775_G776 insYVMA	3.4	28

a Antiproliferative activity in Ba/F3 via CellTiter-Glo.

b Fold selectivity against Ba/F3 EGFR WT. All data represents *n* ≥ 2. See Supporting Information, Table S7, for all IC_50_ statistical analyses. N/A: not applicable.

**Figure 4:**
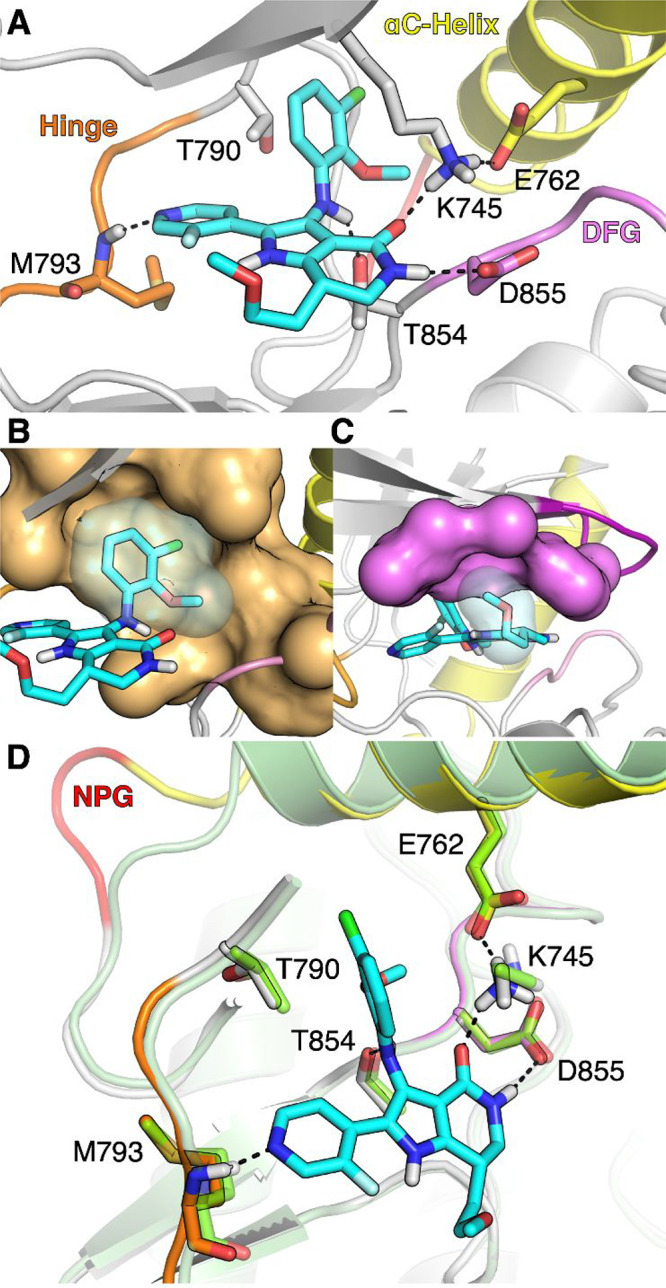
(A) X-ray cocrystal structure of **23** bound to EGFR ex20insNPG protein (PDB 9FQP). EGFR WT residue numbering. All hydrogens were modeled. The glycine-rich loop portion (AA715–729) and the N-terminal domain of the proteins were removed for image clarity. (B) Highlight of the van der Waals contacts between **23** and the gatekeeper pocket with a surface representation showing the back of the ATP-binding site (tan). (C) Highlight of the van der Waals contacts between the ethylmethoxy substituent of **23** (white) and the hydrophobic patch residues (G719, L718, V726, and F723) of the glycine-rich loop shown as a surface (pink). (D) Overlay of the above with X-ray cocrystal structure of **23** bound to EGFR WT protein (green) (PDB 9FRD).

To further understand the ex20ins mutant potency and selectivity vs WT-EGFR observed for **23**, X-ray cocrystal structures of **23** with the EGFR ex20insNPG mutant and WT proteins were obtained. The insNPG X-ray structure confirmed **23** binds to the αC-helix-in conformation of the protein in the catalytic active site where it makes several key interactions, including those predicted for **1** (Figure 4A). The 3-F pyridyl N of **23** forms a critical hydrogen bond with the backbone NH of Met793. Next, the lactam of **23** forms a hydrogen bond network with catalytic Lys745 and Asp855 of the DFG motif. Importantly, this hydrogen bond network also stabilizes the αC-helix-in conformation of the mutant via a salt bridge with the αC-helix (Glu762). Next, the lactam carbonyl also forms a key intramolecular hydrogen bond with the aniline NH, positioning the aryl ring alongside the Thr790 gatekeeper residue, where the 2-OMe and 3-Cl substituents occupy a hydrophobic pocket formed by the Leu788 and Met766 (Figure 4B). The lipophilic nature and asymmetric shape of the gatekeeper pocket helps rationalize the previously investigated aniline moiety SAR (Table 2). Furthermore, the aniline ring itself forms a cation−π interaction with the catalytic Lys745. Next, the core pyrrole NH forms a bifurcated intramolecular hydrogen bond network, including: (1) a nonclassical intramolecular hydrogen bond with the 3-F substituent of the pyridine, which was predicted based on our initial minimum pharmacophore SAR and promotes a favorable coplanar arrangement between the hinge binder and the core, and (2) an intramolecular H-bond with the oxygen atom of the ethylmethoxy group, positioning it into the solvent facing ribose pocket where it drives potency through van der Waals interactions with glycine-rich loop residues (Figure 4C), and is predicted to displace an energetically unfavorable water (see WaterMap in Supporting Information). Interestingly, inspection of the EGFR WT X-ray cocrystal structure with **23** (Figure 4D), also found to be in the αC-helix-in conformation, indicated no obvious structural differences that could account for the functional selectivity observed in cell proliferation experiments.

The αC-helix is a key regulatory element that controls the activation status of EGFR. The rotation of the αC-helix from an inactive αC-helix-out conformation to an active αC-helix-in confirmation leads to the formation of a key salt bridge between Glu762 on the αC-helix and the catalytic Lys745 of the β3 sheet (Figure 1B).^40^ As mentioned, ex20ins mutations are believed to “push” and “lock” the αC-helix into an active conformation leading to the activation of downstream signaling pathways and uncontrolled cell proliferation.^12,16^ We hypothesized that differences in protein dynamics, particularly with respect to movement of the αC-helix, could explain the observed selectivity for **23** and related chemical matter in the absence of structural differences in the compound binding site.

Protein dynamics-based selectivity takes advantage of the differential sampling of conformational states between two highly similar proteins. This occurs when the target protein displays a shift in ensemble state sampling resulting in the enrichment of a ligandable conformation or conformations relative to its antitarget (e.g., a closely related mutant or isoform). Two recent examples of protein dynamics-based selectivity include: (1) the discovery of lirafugratinib,^41^ a potent and selective FGFR2 vs FGFR1 inhibitor, and (2) the discovery of RLY-2608^42^ and STX-478,^43^ mutant-selective allosteric PI3Kα inhibitors. To gain insight into the mechanism by which **23** elicits functional EGFR ex20ins mutant vs WT selectivity in vitro, we conducted metadynamics simulations on apo EGFR WT and near-loop EGFR ex20ins mutant (insNPG, insASV, and insSVD) structures derived from a WT X-ray cocrystal structures with the *R* enantiomer of **1** (compound **S1**, see Supporting Information and ref (44) for additional details). The simulations showed that these near-loop ex20ins induced a shift in the conformational ensembles sampled by the αC-helix, preferentially stabilizing the αC-helix-in “active state,” and supporting the notation that **23** and related chemical matter derive their functional selectivity by interacting with this enriched conformational state.

Assessment of **23** in rat and dog PK studies showed moderate rat total and unbound plasma clearance (Cl_p_ and Cl_p,u_), however dog Cl_p_ was significantly above hepatic blood flow (Table 5). Consequently, strategies to improve Cl_p,u_ in both rat and dog, while maintaining ex20ins mutant potency and selectivity vs WT-EGFR, were explored. Human hepatocyte metabolite identification studies with **23** indicated that the two most prevalent oxidative metabolic pathways included: (1) lactam oxidation followed by elimination and (2) demethylation of the ethylmethoxy substituent, with the former representing the major metabolic pathway. Accordingly, the putative major human hepatocyte metabolite **26** was synthesized and profiled but found to be 20- to 25-fold less potent than **23**. Next, attempts were made to prevent oxidation of the 7-position of the pyrrololactam core through substitution. In particular, −Me substitution, exemplified by isomer **27**, led to a moderate loss in ex20ins mutant activity and selectivity over WT-EGFR, but unfortunately a significant increase in rat Cl_p_ (3.95 L h^1–^ kg^–1^), potentially related to increased log *D*. Next, given that polarity had been well tolerated with respect to mutant activity in the ribose pocket, we turned our attention toward modifications of the ethylmethoxy substituent that were predicted to lower log *D* and anticipated to reduce oxidative metabolism. Cyclization of ethylmethoxy group to afford oxetane **28** decreased mutant activity and selectivity and led to an increase in rat Cl_p_ above hepatic blood flow (6.17 L h^–1^ kg^–1^). Another cyclization strategy of the ethylmethoxy group through the synthesis of 1,4-dioxolane analogues **29** and **30** was explored. The 4-O of the 1,4-dioxolane was predicted to be solvent exposed and therefore its incorporation was not expected to pay a desolvation penalty. Accordingly, **29** and **30** were found to exhibit similar or slightly improved ex20ins mutant activity compared to **23** with similar selectivity. Of the two analogues, **30** possessed a superior combination of rat and dog Cl_p,u_ (48 and 22 L h^–1^ kg^–1^, respectively).

**Table 5:**
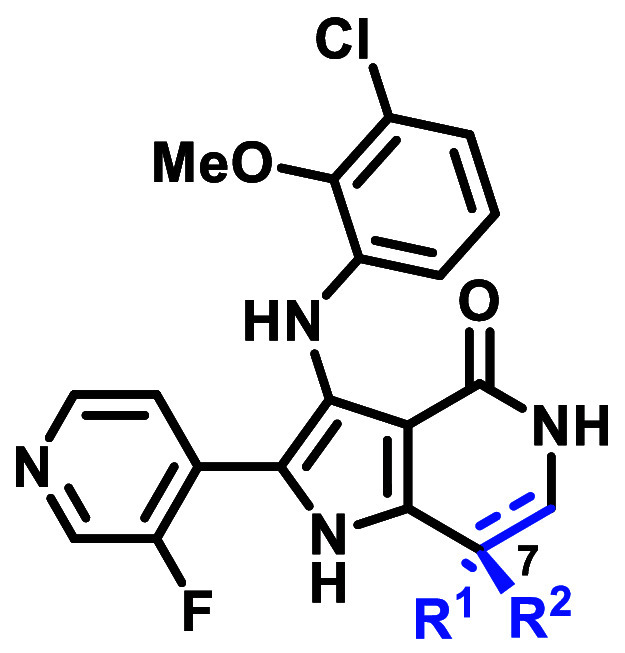

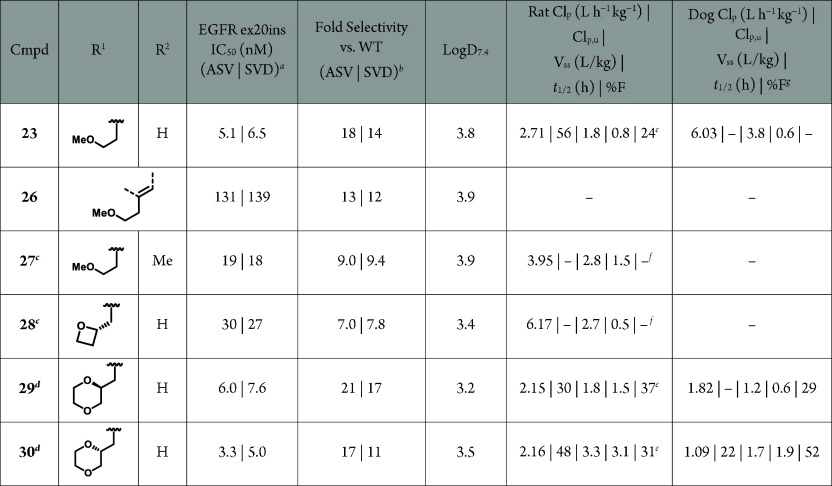
Optimization of Rat and Dog PK

a Antiproliferative activity in Ba/F3 via CellTiter-Glo.

b Fold selectivity against Ba/F3 EGFR WT. All data represents *n* ≥ 2. See Supporting Information, Tables S4–S6, for all IC_50_ statistical analyses.

c Stereochemistry arbitrarily assigned, most mutant active and selective isomers depicted (see Supporting Information for additional analogue data).

d Lactam stereochemistry based on biological activity analogy to **23** (vide infra).

e Sprague-Dawley rats (*n* = 3), IV dose = 2 mg/kg using 20% DMSO + 40% PEG400 in water and PO dose = 5 mg/kg using suspension of 1% Tween 80, 2% HPMC, 97% water, pH = 7.0.

f Sprague-Dawley rats (*n* = 3), IV cassette dose (4 compounds total) = 0.5 mg/kg using 20% DMSO + 40% PEG400 in water.

g Beagle dogs (*n* = 3), IV dose = 0.5 mg/kg using 20% DMSO + 60% PEG400 in water and PO dose = 1 mg/kg using suspension of 1% Tween 80, 1% HPMC, 98% water, pH = 7.0; Cl_p_, plasma clearance; Cl_p,u_, unbound in vivo clearance (rat or dog in vivo clearance/rat or dog free fraction), free fraction calculated from plasma protein binding determined by ultracentrifugation method. Cl_p,u_ not provided when Cl_p_ exceeded 2/3 hepatic blood flow for rat or dog.

With an optimal ribose pocket group in hand, pyrimidine hinge binders were reassessed. 4-pyrimidine analogue **31** and 2-methyl-4-pyrimidine analogue **32** demonstrated favorable ex20ins mutant activity and selectivity vs WT-EGFR with acceptable in vitro ADMET profiles (Table 6). In particular, 2-methyl-4-pyrimidine **32**, afforded a dramatic decrease in reversible CYP inhibition and hERG inhibition. Furthermore, it demonstrated the best combination of rat and dog Cl_p,u_ and oral bioavailability (*F*). The absolute stereochemistry of **32** with respect to the lactam stereocenter was confirmed to be the *S*-isomer by single X-ray crystallography, consistent with the previously determined stereochemistry of **23**.

**Table 6:**
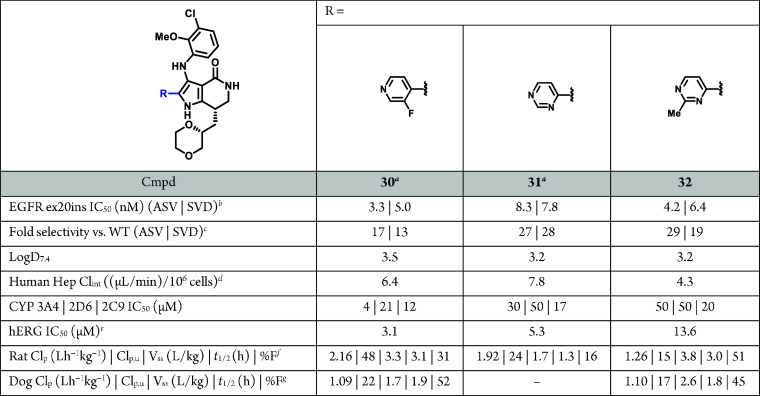
Optimization of Hinge Binder Group

a Lactam stereochemistry assigned based on biological activity analogy to **32** (see Supporting Information).

b Antiproliferative activity in Ba/F3 via CellTiter-Glo.

c Fold selectivity against Ba/F3 EGFR WT. All data represents *n* ≥ 2. See Supporting Information, Tables S4–S6, for all IC_50_ statistical analyses.

d Intrinsic clearance obtained from isolated human hepatocyte cells.

e hERG inhibition was measured using the SyncroPatch 384*i*/384 automated patch clamp system.

f Sprague-Dawley rats (*n* = 3), IV dose = 2 mg/kg using 20% DMSO + 40% PEG400 in water and PO dose = 5 mg/kg using suspension of 1% Tween 80, 2% HPMC, 97% water, pH = 7.0.

g Beagle dogs (*n* = 3), IV dose = 0.5 mg/kg using 20% DMSO + 60% PEG400 in water and PO dose = 1 mg/kg using suspension of 1% Tween 80, 1% HPMC, 98% water, pH = 7.0. Cl_p_, plasma clearance; Cl_p,u_, unbound plasma clearance (rat or dog plasma clearance/rat or dog free fraction), free fraction calculated from plasma protein binding determined by ultracentrifugation method.

**Figure 5:**
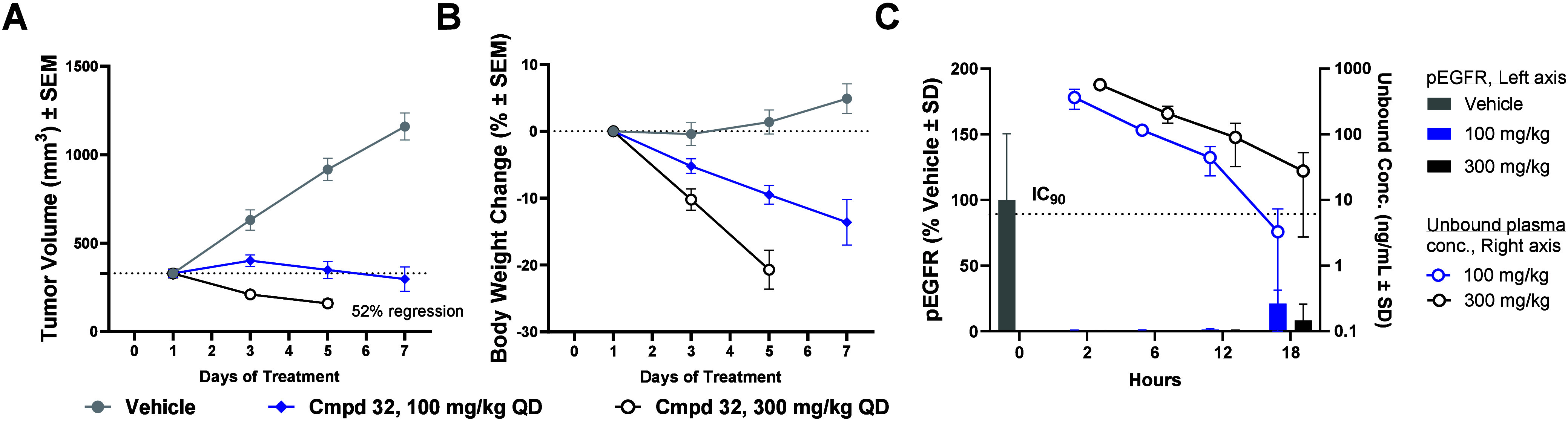
Compound **32** dosed orally once daily results in regression of EGFR ex20ins tumor allografts. (A) Effect of compound **32** on Ba/F3 EGFR V769_D770 insASV tumor allografts growth in female BALB/c nude mice (QD PO dosing). Data represent the mean tumor volume ± SEM (*n* = 6/group): all treated groups were *p* < 0.0001 using a two-way RM ANOVA followed by Tukey’s post hoc comparisons of the means. TGI and % regression calculations as noted in Supporting Information. (B) Tolerability, as reflected by body weight in female BALB/c nude mice. (C) PK/PD single dose study. Mean unbound plasma concentrations ± SD (*n* = 6/group). PD determined by % of vehicle control ± SD. IC_90_ = in vitro cellular antiproliferative activity in Ba/F3 via CellTiter-Glo in ng/mL.

The favorable overall profile of **32** prompted further evaluation in vivo in a mouse Ba/F3 EGFR V769_D770 insASV allograft tumor model (Figure 5). **32** at 100 mg/kg QD resulted in tumor stasis, while 300 mg/kg QD led to robust tumor regression, but was discontinued due to body weight loss. Greater than 10% body weight loss was observed at both doses, but was less pronounced at 100 mg/kg QD. A single dose PK/PD study was also conducted and found to be in good agreement with the in vivo tumor growth inhibition (TGI) results (Figure 5C). **32** at 300 mg/kg QD resulted in sustained unbound plasma coverage of the in vitro Ba/F3 EGFR insASV IC_90_ (up to 18 h) and accordingly robust pEGFR inhibition, with minor (8%, Figure 5C, black bar) rebound of pEGFR at 18 h. 100 mg/kg QD showed similar levels of pEGFR inhibition, with a higher (21%, Figure 5C, blue bar) rebound of pEGFR at 18 h, concomitant with unbound plasma coverage falling below IC_90_ levels beyond 12 h. Unfortunately, while we were able to observe a PK-PD-TGI relationship with **32**, all doses led to significant body weight loss in the animals (≥10%, Figure 5B), including separately tested lower doses of 60 mg/kg QD and 30 mg/kg BID (see Supporting Information, Figure S1).

**Figure 6:**
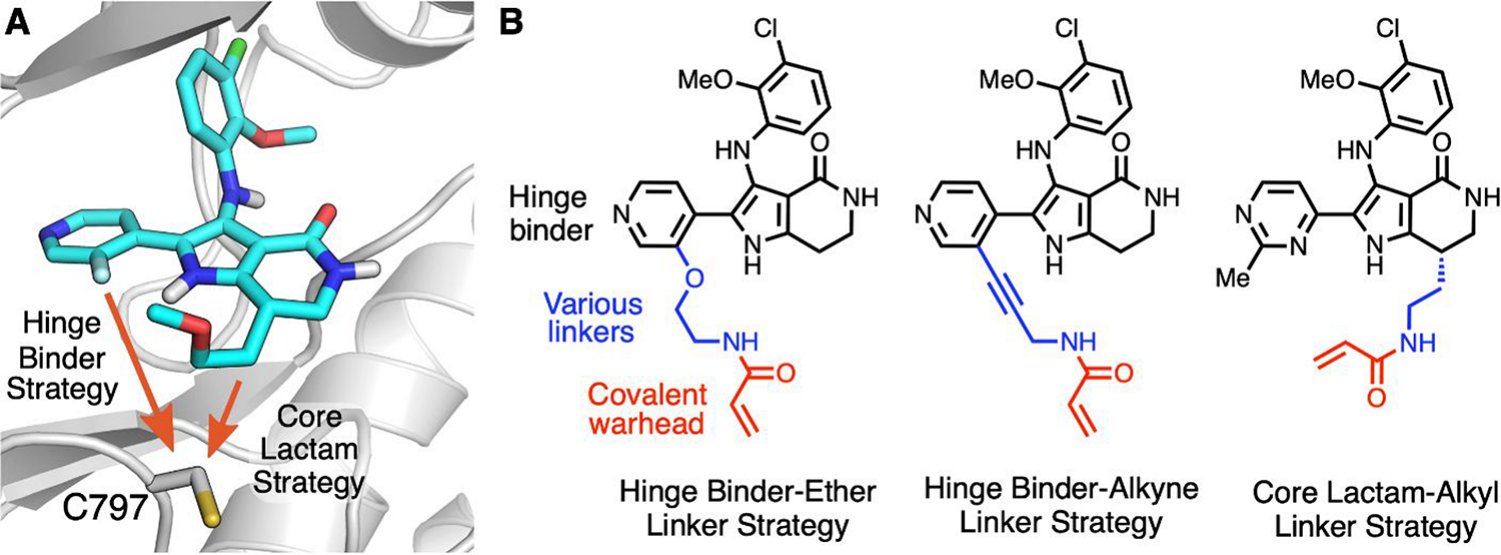
(A) X-ray cocrystal structure of **23** in with EGFR exon20insNPG protein (PDB 9FQP). (B) Examples of design strategies toward covalent bond formation with C797.

To assess if the inability of **32** to afford efficacy with concomitant tolerability in the mouse EGFR exon20insASV tumor model was related to the chemotype, four additional compounds **30**, **33**, **34**, and **35** were tested in vivo (Table 7). As with **32**, all compounds were able to achieve ≥20% regression), but significant (≥10%) body weight loss was simultaneously observed. Kinome selectivity profiles were measured for all five compounds via the KINOME*scan* screening platform (468 kinases). The kinome selectivity S(10) scores measured at 3 μM compound concentration ranged from 0.10 to 0.21 for the compound set.^45^ We surmised that one or multiple off-target kinases might be contributing to the poor observed mouse tolerability. Consequently, we hypothesized that lowering the S(10) and increasing the kinome selectivity would result in compounds with improved mouse tolerability at efficacious doses. We envisioned that the incorporation of a covalent warhead into our lead chemical series targeting Cys797 in the EGFR active site could impart added specificity and ultimately improved kinome selectivity. Sequence alignment indicated there are only 10 other kinases within the human kinome, including HER2 and HER4, that contain cysteines at the structurally equivalent position. The covalent warhead was anticipated to clash or be unable to engage in productive covalent bond formation with noncysteine residues located at structurally equivalent positions across the kinome, ultimately leading to improved kinome selectivity. Additionally, C797 is known to be the target of second- and third-generation EGFR TKIs and several published EGFR ex20ins mutant-selective inhibitors.^28,47^ Inspection of the EGFR exon20insNPG X-ray cocrystal structure with **23** suggested two vectors from which to engage C797 with a covalent warhead (e.g., acrylamide), namely via the hinge binder or the core lactam (Figure 6A). We explored all three strategies outlined in Figure 6B but will focus our discussion on the hinge binder-alkyne linker strategy as it ultimately was the most productive. We had previously demonstrated 3-alkynylpyridine analogue **33** maintained the required pseudoplanar orientation between hinge-binder and the pyrrololactam core to afford potent EGFR ex20ins mutant activity and selectivity over WT-EGFR. Based on this starting point, alkyne linker designs were assessed using a covalent docking model based on the WT-EGFR XRC structure (PDB 1M17). In particular, a propargylic amine linker was predicted to have the optimal spacing to position an acrylamide warhead for covalent bond formation with C797.^46^

**Table 7:**
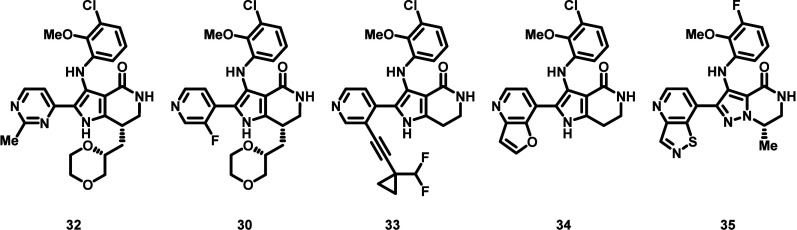
Evaluation of Mouse In Vivo Activity and Tolerability vs Kinome Selectivity

Cmpd	EGFR ex20ins IC_50_ (nM) (ASV | SVD)^a^	Fold selectivity vs. WT (ASV | SVD)^b^	S(10)^c^ at 3 μM	Tumor growth inhibition in mouse Ba/F3 EGFR ex20insASV allograft model^d^	Tolerability in mouse Ba/F3 EGFR ex20insASV allograft model^e^
**32**	4.2 | 6.4	29 | 19	0.10	Yes	No
**30**	3.3 | 5.0	17 | 11	0.15	Yes	No
**33**	11 | 14	17 | 15	0.10	Yes	No
**34**	15 | 12	14 | 17	0.10	Yes	No
**35**	11 | 7.3	14 | 23	0.21	Yes	No

a Antiproliferative activity in Ba/F3 via CellTiter-Glo.

b Fold selectivity against Ba/F3 EGFR WT. All data represents *n* ≥ 2. See Supporting Information, Tables S4–S6, for all IC_50_ statistical analyses.

c Eurofins KINOMEscan selectivity profiling at 3 μM, S(10) = (number of nonmutant kinases with %Ctrl <10)/(number of nonmutant kinases tested).

d Positive TGI in Ba/F3 mouse allograft model defined by ≥20% regression.

e Tolerability defined by body weight loss ≤10% in female BALB/c nude mice. Efficacy and tolerability defined within the same study for the same oral dose. See Supporting Information, Table S13, for detailed TGI and tolerability analyses.

**Figure 7:**
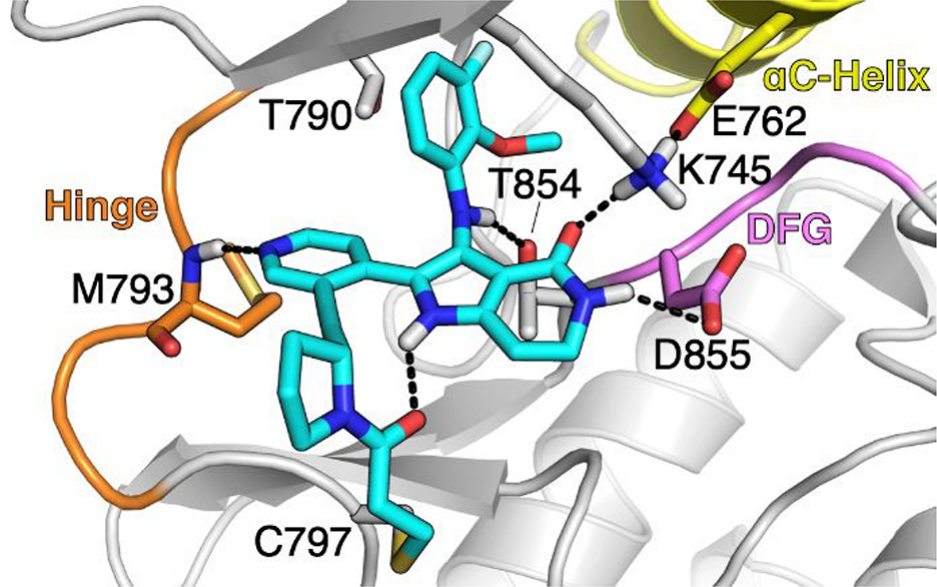
X-ray cocrystal structure of **39** bound to EGFR ex20insNPG protein. Hydrogens have been added and the glycine-rich loop (AA715–729) has been removed for image clarity (PDB 9FQS).

We began our covalent medicinal chemistry efforts within the alkyne linker subseries by exploring the acrylamide warhead vector for engaging C797. A series of 4- to 6-membered ring containing propargylic amine analogues of both *R*- and *S*-stereochemistry were prepared (Table 8). Of the analogues evaluated, *R*-pyrrolidine **38** stood out in terms of EGFR ex20ins mutant activity and selectivity vs WT-EGFR. Furthermore, the formation of the anticipated covalent bond was confirmed through an X-ray cocrystal structure of the F-analogue of **38** (**39**), which exhibited similar ex20ins mutant activity and selectivity (Figure 7).

**Table 8:**
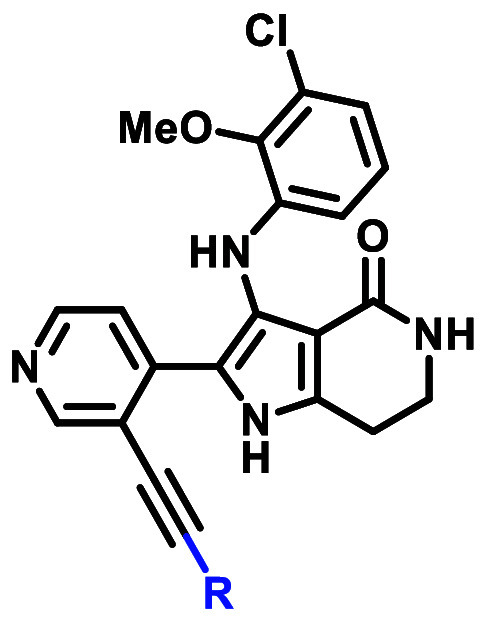

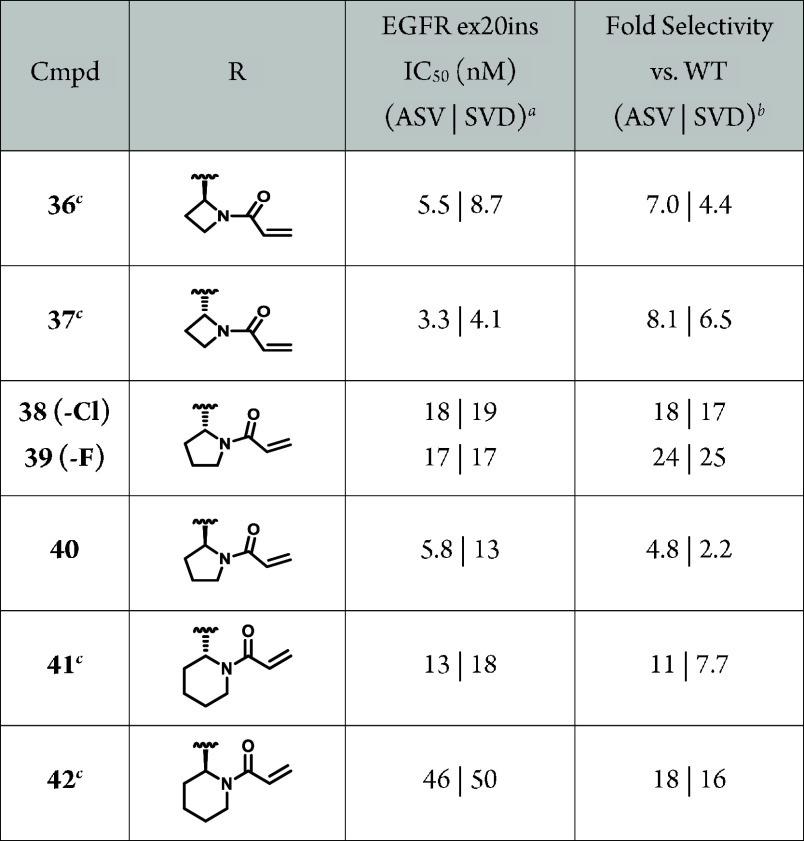
Exploration of Alkyne Linker Structure–Activity Relationship

a Antiproliferative activity in Ba/F3 via CellTiter-Glo.

b Fold selectivity against Ba/F3 EGFR WT. All data represents *n* ≥ 2. See Supporting Information, Tables S4–S6, for all IC_50_ statistical analyses.

c Absolute stereochemistry arbitrarily assigned.

Gratifyingly, the introduction of the covalent warhead in **38** led to dramatically improved kinome selectivity (S(10) at 3 μM = 0.032, Table 9) compared to noncovalent chemical matter. To further confirm covalent bond formation, the biochemical *k*_inact_/*K*_I_ was determined using a chelation enhanced fluorescence (ChEF) assay with both EGFR ex20insNPG mutant and WT protein.^48^ The level of mutant biochemical activity and selectivity was in good agreement with the cellular antiproliferation data. Unfortunately, rat PK of **38** indicated that it suffered from high plasma clearance in rats (Cl_p_ = 12.7 L h^–1^ kg^–1^), significantly above rat hepatic blood flow. To understand this result, hepatocyte, glutathione (GSH), and whole blood stability (WBS) were measured. While the GSH *t*_1/2_ was acceptable (281 min), the rat hepatocyte stability and WBS were poor. We hypothesized that low rat WBS (*t*_1/2_ < 1 h) was having a significant impact on the rat in vivo clearance.

**Table 9:** Compound **38** in Vitro and in Vivo Profile

S(10) at 3 μM^a^	0.032
Biochemical EGFR ex20insNPG *k*_inact_/*K*_I_ at 1 mM ATP (1/(M*s))^b^	1.02 × 10^5^
Biochemical EGFR WT *k*_inact_/*K*_I_ at 1 mM ATP (1/(M*s))^b^	1.54 × 10^3^
Biochemical EGFR ex20insNPG fold selectivity vs. WT^b^	66
Rat Hep Cl_int_ ((μL/min)/10^6^ cells)^c^	237
5 mM GSH, 37 °C, pH 7.4 *t*_1/2_ (min)^d^	281
Rat whole blood *t*_1/2_ (min)^e^	59
rat Cl_p_ (L h^–1^ kg^–1^) | *V*_ss_ (L/kg) | *t*_1/2_ (h)^f^	12.7 | 2.9 | 0.4

a Eurofins KINOMEscan selectivity profiling at 3 μM, S(10) = (number of nonmutant kinases with %Ctrl < 10)/(number of nonmutant kinases tested).

b *K*_inact_/*K*_I_ was determined using a chelation enhanced fluorescence (ChEF) biochemical assay with AQT0001 peptide substrate. Data is geometric mean ± geometric standard deviation. See Supporting Information, Table S3, for individual *k*_inact_ and *K*_I_ parameters.

c Intrinsic clearance obtained from isolated rat hepatocyte cells.

d *t*_1/2_ determined by parent depletion (MS detection) in 5 mM GSH, 37 °C, pH 7.4 aqueous phosphate buffer.

e *t*_1/2_ determined by parent depletion (MS detection) of 5 μM compound concentration in rat whole blood at 37 °C.

f Sprague-Dawley rats (*n* = 3), IV cassette dose (4 compounds total) = 0.5 mg/kg using 20% DMSO + 40% PEG400 in water. Cl_p_, plasma clearance.

To improve the rat WBS, and ultimately the rat in vivo clearance, we explored the impact of pyrrolidine substitution. We anticipated steric changes around the pyrrolidine could modulate GST recognition and GST-mediated GSH conjugation (Table 10). Additionally, pyrrolidine substitution was predicted to increase the p*K*_a_ of the parent amine of the acrylamide, which is known to correlate with decreased warhead reactivity and increased GSH *t*_1/2_.^49^ Of the cyclopropyl substituted analogues prepared with consistent relative stereochemistry with respect to the alkyne, **43**, **44**, and **45**, only **45** exhibited acceptable mutant potency and selectivity, however the GSH *t*_1/2_ and rat WBS was not significantly improved compared to **38**. Bridged pyrrolidine **46** significantly improved the intrinsic GSH stability but exhibited only modestly improved rat WBS. Finally, methyl pyrrolidine **47**, demonstrated the best combination of cellular EGFR ex20ins mutant potency, selectivity, GSH stability, and rat WBS, but unfortunately rat plasma clearance remained close to hepatic blood flow (rat Cl_p_ = 5.1 L h^–1^ kg^–1^).

**Table 10:**
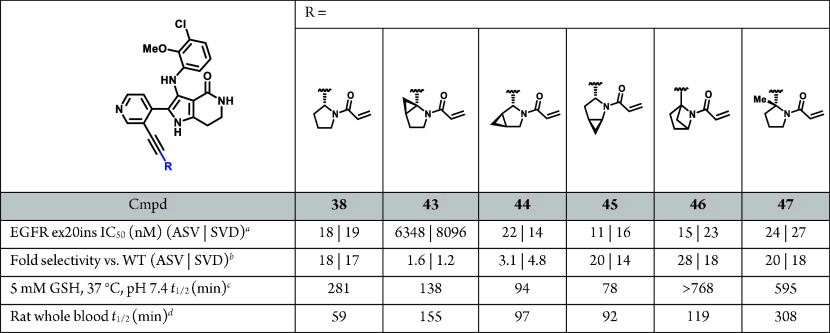
Modulation of GSH and Rat WBS Through Pyrrolidine Linker Substitution

a Antiproliferative activity in Ba/F3 via CellTiter-Glo.

b Fold selectivity against Ba/F3 EGFR WT. All data represents *n* ≥ 2. See Supporting Information, Tables S4–S6, for all IC_50_ statistical analyses.

c *t*_1/2_ determined by parent depletion (MS detection) in 5 mM GSH, 37 °C, pH 7.4 aqueous phosphate buffer.

d *t*_1/2_ determined by parent depletion (MS detection) of 5 μM compound concentration in rat whole blood at 37 °C.

Consequently, we turned our attention to modification of the acrylamide warhead toward improved intrinsic GSH stability and rat WBS (Table 11).^50,51^ While 2-F acrylamide **48** significantly improved the rat WBS (*t*_1/2_ > 512 min) it was not tolerated in terms of EGFR ex20ins mutant potency or selectivity vs WT-EGFR. Butynamide **49** exhibited good GSH stability, but only modestly improved rat WBS. Interestingly, while β-dimethylamino acrylamide **50** exhibited a modest reduction in cellular mutant activity, it maintained good selectivity and demonstrated a dramatic increase in rat WBS (*t*_1/2_ > 512 min). Dimethylamino group modifications, exemplified by pyrrolidine **51** and morpholine **52**, were explored and generally led to acceptable to good rat WBS but a reduction in cellular EGFR ex20ins mutant activity and selectivity.

**Table 11:**
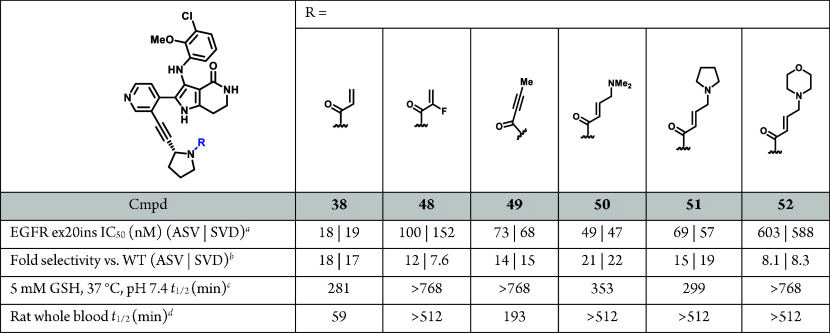
Modulation of GSH and Rat WBS through Covalent Warhead Modification

a Antiproliferative activity in Ba/F3 via CellTiter-Glo.

b Fold selectivity against Ba/F3 EGFR WT. All data represents *n* ≥ 2. See Supporting Information, Tables S4–S6, for all IC_50_ statistical analyses.

c *t*_1/2_ determined by parent depletion (MS detection) in 5 mM GSH, 37 °C, pH 7.4 aqueous phosphate buffer.

d *t*_1/2_ determined by parent depletion (MS detection) of 5 μM compound concentration in rat whole blood at 37 °C.

Given methyl pyrrolidine linker **47** and β-dimethylamino acrylamide **50** demonstrated the best combination of cellular EGFR ex20ins mutant activity, selectivity, and rat WBS within their respective series, these modifications were combined to give compound **53** (Table 12). **53** exhibited a synergistic improvement in cellular Ba/F3 EGFR ex20insASV and insSVD mutant activity and demonstrated excellent selectivity vs WT-EGFR (24- and 22-fold, respectively), approaching osimertinib’s level of selectivity for EGFR L858R/T790 M (29-fold). Relative cellular activity across a panel of Ba/F3 EGFR and HER2 ex20ins mutants was analogous to **23** (see ref (44) for additional details). Moreover, **53** retained good biochemical EGFR ex20insNPG activity, selectivity over WT-EGFR, and excellent kinome selectivity (S(10) at 3 μM = 0.027). Finally, **53** demonstrated favorable rat WBS (*t*_1/2_ > 512 min) and combined with a rat blood-to-plasma ratio of 2.54 (Table 14) this translated into acceptable in vivo rat blood clearance (Cl_b_) of 2.57 L h^–1^ kg^–1^ (Table 14; Cl_b_ = Cl_p_/blood-to-plasma ratio).

**Table 12:**
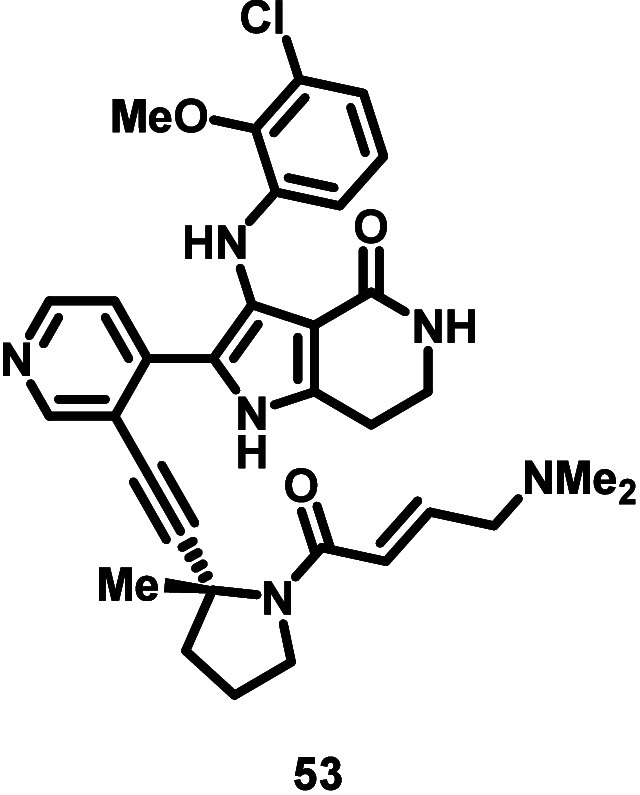
Combination of Optimal Pyrrolidine Substitution and Covalent Warhead

EGFR exon20ins IC_50_ (nM) (ASV | SVD)^a^	5.4 | 5.8
Fold selectivity vs. WT (ASV | SVD)^b^	24 | 22
Biochemical EGFR exon20insNPG *k*_inact_/*K*_I_ at 1 mM ATP (1/(M*s))^c^	6.2 × 10^4^
Biochemical EGFR WT *k*_inact_/*K*_I_ at 1 mM ATP (1/(M*s))^c^	3.24 × 10^3^
Biochemical EGFR exon20insNPG fold selectivity vs. WT^c^	19
S(10) at 3 μM^d^	0.027
Rat whole blood *t*_1/2_ (min)^e^	>512

a Antiproliferative activity in Ba/F3 via CellTiter-Glo.

b Fold selectivity against Ba/F3 EGFR WT. All data represents *n* ≥ 2. See Supporting Information, Tables S4–S6, for all IC_50_ statistical analyses.

c *K*_inact_/*K*_I_ was determined using a chelation enhanced fluorescence (ChEF) biochemical assay with AQT0001 peptide substrate. Data is geometric mean ± geometric standard deviation. See Supporting Information, Table S3, for individual *k*_inact_ and *K*_I_ parameters.

d Eurofins KINOMEscan selectivity profiling at 3 μM, S(10) = (number of nonmutant kinases with %Ctrl <10)/(number of nonmutant kinases tested).

e *t*_1/2_ determined by parent depletion (MS detection) of 5 μM compound concentration in rat whole blood at 37 °C.

**Figure 8:**
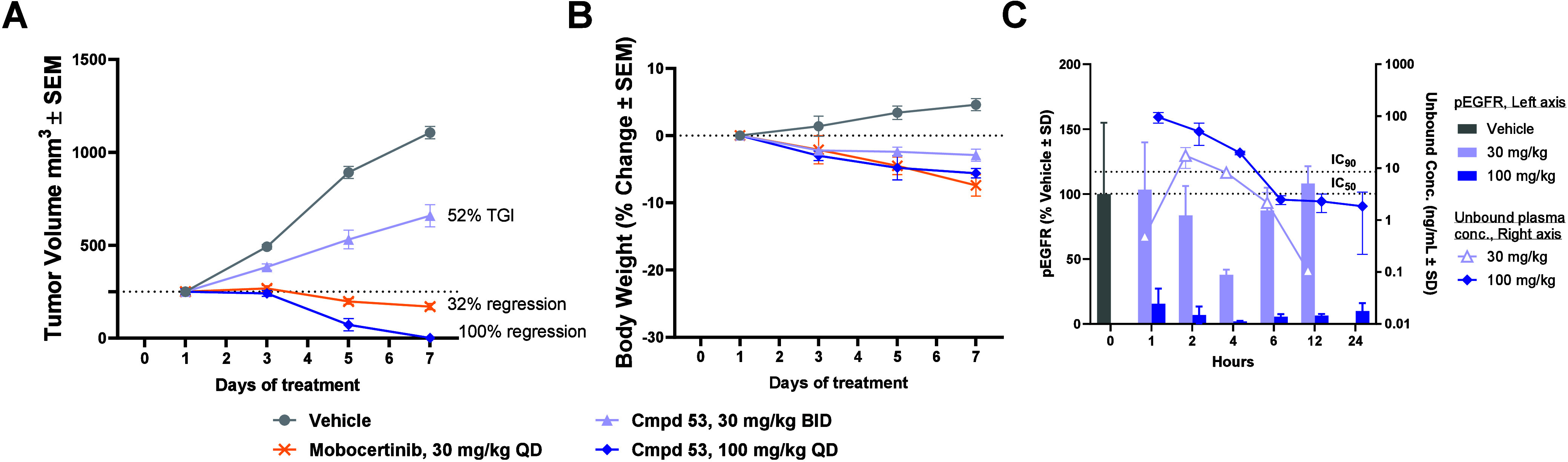
Compound **53** dosed orally once daily results in regression of EGFR ex20ins tumor allografts. (A) Effect of compound **53** and mobocertinib on Ba/F3 EGFR V769_D770 insASV tumor allografts growth in female BALB/c nude mice (QD and BID PO dosing). Data represent the mean tumor volume ± SEM (*n* = 6/group): all treated groups were *p* < 0.0001 using a two-way RM ANOVA followed by Tukey’s post hoc comparisons of the means. TGI and % regression calculations as noted in Supporting Information. (B) Tolerability, as reflected by body weight in female BALB/c nude mice during the study. (C) PK/PD single dose study. Mean unbound plasma concentrations ± SD (*n* = 3/group). PD determined by % of vehicle control ± SD. IC_50_ and IC_90_ = in vitro cellular antiproliferative activity in Ba/F3 via CellTiter-Glo in ng/mL.

Given the favorable profile of **53**, including the improved kinome selectivity, it was advanced into the mouse Ba/F3 EGFR ex20insASV allograft model. **53** (30 mg/kg BID) resulted in 52% TGI, while 100 mg/kg QD afforded 100% tumor regression over 7 days of dosing, greater than the benchmark clinical phase ex20ins mutant inhibitor mobocertinib at a clinically representative dose (30 mg/kg QD)^52^ (Figure 8A). Gratifyingly, both 30 mg/kg BID and 100 mg/kg QD doses of **53** demonstrated robust efficacy with minimal (<6%) body weight loss in the animals (Figure 8B). Consistent with our hypothesis, the improved kinome selectivity of **53** translated into the ability to merge robust efficacy with acceptable tolerability at 100 mg/kg QD in the mouse allograft model.

A single dose PK/PD study on **53** in Ba/F3 EGFR ex20insASV allograft mice aligned well with the in vivo efficacy results (Figure 8C). **53** at 30 mg/kg QD gave a modest level of pEGFR signal inhibition at 4 h postdose and signal rebound by 12 h, resulting in tumor progression (with BID dosing), while **53** at 100 mg/kg QD afforded strong pEGFR signal inhibition for 24 h and led to robust tumor regression. We noted from this experiment that the PK data was somewhat variable due to composite terminal sampling of each time point from individual animals. Nonetheless, the 100 mg/kg QD result indicated that covalent inhibition could achieve persistent pharmacodynamic effect for 24 h without sustained unbound plasma coverage of the in vitro Ba/F3 EGFR ex20insASV IC_50_. As suggested by prior analyses of covalent inhibitors,^53^ rather than the duration of IC_50_ coverage, a combination of the inhibitor unbound exposure area under the curve (AUC_u_), inhibitor *k*_inact_/*K*_I_, and protein half-life and resynthesis rate governs the covalent occupancy and ultimately the pharmacodynamic effect of the inhibitor; this phenomenon has also been described for several other covalent small molecule inhibitors.^54,55^

To further assess the EGFR ex20ins mutant vs WT selectivity in a more physiologically relevant isogenic in vitro setting, a CRISPR/Cas9 approach was used to knock-in the V769_D770 insASV and V770_D771 insSVD mutations homozygously into an EGFR WT human cancer cell line background (NCI-H2073). The cellular antiproliferative activity for **53** was assessed in vitro in this set of isogenic cell lines and the selectivity was compared to that of osimertinib for the NCI-H1975 EGFR L858R/T790M human cancer cell line (Table 13). In this system, the mutant selectivity for **53** compared favorably to that of osimertinib.

**Table 13:** Cellular Antiproliferative Activity and Selectivity of Compound **53** and Osimertinib in Human Cancer Cell Lines^a^

	IC_50_ (nM)				
Cmpd	NCI-H1975 EGFR LR/TM^b^	NCI-H2073 EGFR ex20insASV KI^c^	NCI-H2073 EGFR ex20insSVD KI^c^	NCI-H2073 EGFR WT parental	Fold selectivity vs. WT^d^
Osimertinib	9.0	N/A	N/A	55.8	6.2
**53**	N/A	10.1	6.1	55.2	5.5, 9.1

a Antiproliferative activity measured via CellTiter-Glo.

b LR/TM = L858R/T790M.

c KI = knock-in.

d Fold selectivity is proliferation IC_50_ in NCI-H2073 EGFR wild-type parental divided by the IC_50_ of the noted EGFR mutant cell lines. All data represents *n* ≥ 2. See Supporting Information, Tables S9–S12, for all IC_50_ statistical analyses.

**Figure 9:**
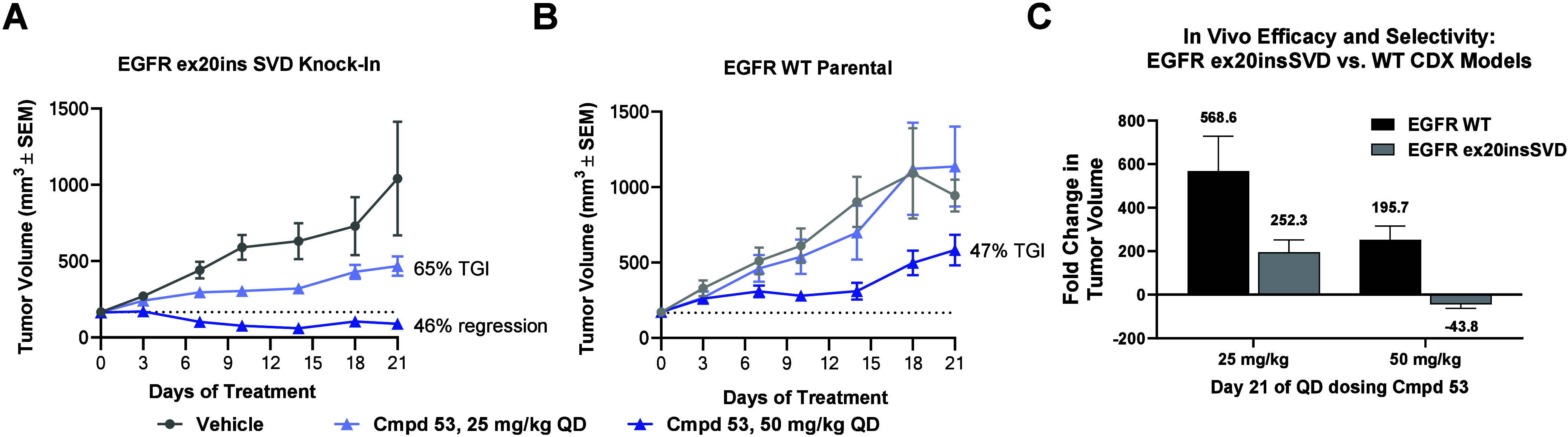
Compound **53** dosed orally results in selective regression of EGFR ex20insSVD tumor xenografts relative to EGFR WT control xenografts. (A) Effect of **53** on NCI-H2073 EGFR D770_N771 insSVD homozygous knock-in tumor xenografts growth in female BALB/c nude mice (QD PO dosing). *p* ≤ 0.0001 for 50 mg/kg and ≤0.05 for 25 mg/kg. (B) Effect of **53** on NCI-H2073 parental (EGFR wild-type) tumor xenografts growth in female BALB/c nude mice (QD PO dosing). Data represent the mean tumor volume ± SEM (*n* = 6/group): ≤ 0.05 for 50 mg/kg and not significant for 25 mg/kg. For both (A) and (B) data represent the mean tumor volume ± SEM (*n* = 6/group), *p*-value was calculated using a two-way RM ANOVA followed by Tukey’s post hoc comparisons of the means. TGI and % regression calculations as noted in Supporting Information. (C) Fold change in tumor growth from (A) and (B). Data represents the mean of fold change in tumor volume change in individual tumors at day ±21 SEM as described in Supporting Information.

To determine the level of EGFR WT-sparing in vivo, we developed a pair of mouse xenograft models derived from the parent NCI-H2073 (EGFR WT) and isogenic EGFR knock-in V770_D771 insSVD cell lines. In the insSVD knock-in mouse xenograft model, **53** showed significantly greater efficacy when compared to the WT model at 25 and 50 mg/kg QD (Figure 9), however the WT model was sensitive to a high dose of **53** (100 mg/kg QD, data not presented in Figure 9). All doses tested were well tolerated in both models (≤5% body weight loss at end of dosing period). In particular, the 50 mg/kg dose of **53** afforded 46% tumor regression in the insSVD mutant model (Figure 9A), but only 47% TGI in the WT model (Figure 9B), which translates into a 44-fold decrease in tumor volume in the insSVD model and a 196-fold increase in the WT model (Figure 9C), thus clearly demonstrating a selectivity window in vivo. PK/PD analysis was also performed at an early 6 h time point where robust inhibition was expected for both mutant and WT EGFR. This analysis showed that despite similar exposures in animals bearing either WT or insSVD tumors, a stronger inhibition of pEGFR was observed in the insSVD tumors relative to WT at both tested doses (see Supporting Information, Figure S2).

Table 14 summarizes the in vitro ADME and preclinical PK profile of compound **53**. **53** exhibited good solubility in PBS, SGF, and FeSSIF. The MDCK permeability was moderate with an A–B value of 7.9 × 10^–6^ cm/s and Caco-2 efflux that saturated with increased compound concentration. Moderate hepatocyte stability was observed across species tested. Importantly, the whole blood *t*_1/2_ was greater than 5 h in all preclinical species tested and >512 min in humans. The blood-to-plasma ratio for **53** in rat was slightly elevated at 2.54 compared to dog, monkey, and human. Assessment of rat and dog PK parameters for **53**, showed moderate Cl_b_, moderate volume of distribution, and modest to good oral bioavailability (*F*) in rat and dog, respectively. Monkey exhibited high Cl_b_ near hepatic blood flow, which was significantly underpredicted by in vitro hepatocyte data using the well-stirred model,^56^ and low *F*. For sensitive CYP3A4/5 substrates it is known monkey *F* may underpredict human *F*.^57^ Consequently, single species scaling methods based on the monkey alone were not used for **53** as they were anticipated to overpredict human Cl_p_ and underpredict human *F*. Instead, a combination of the *f*_u_ intercept correction method (FCIM) for in vivo-scaling based on rat, dog, and monkey PK^58^ and in vitro extrapolation from human hepatocyte data were chosen to predict human Cl_p_.^59^ These different methodologies showed a reasonable level of agreement and predicted **53** to have moderate clearance (∼36% of *Q*_H_). Based on this projection and using additional in vitro/in vivo extrapolations and in vivo-scaling methods to project human volume of distribution^60^ the terminal half-life of **53** was predicted to be 9 h. Consistent with the covalent nature of **53**, the projected human *C*_eff,u_ was modeled based on the AUC_u_^53^ capable of delivering ≥80% tumor regression, concomitant with 24 h inhibition of EGFR pharmacodynamic activity, in several additional in vivo mouse CDX and PDX models.^44^ Taken together, the projected human PK and *C*_eff,u_ afforded an acceptable human dose projection.

**Table 14:** In Vitro and In Vivo Profile of **53**

MW | log *D*_7.4_ | TPSA	587 | 3.4 | 103
HT solubility (μM, PBS | SGF | FeSSIF)	278 | 303 | 278
R | D | M | H Hep Cl_int_ ((μL/min)/10^6^ cells)^a^	118 | 18.8 | 61.4 | 27.7
MDCK *P*_app_ A–B (μcm/s)	7.9
Caco-2 efflux ratio (5 | 50 | 100 | 250 μM)	529 | 93.2 | 14.5 | 4.0
R | D | M | H whole blood *t*_1/2_ (min)^b^	>512 | 301 | 337 | >512
R | D | M | H blood-to-plasma ratio	2.54 | 1.03 | 1.11 | 0.708
R | D | M | H PPB (1.0 μM, UC, *f*_u_)	0.065 | 0.055 | 0.039 | 0.016
Rat Cl_b_ (L h^–1^ kg^–1^) | *V*_ss,b_ (L/kg) | *t*_1/2_ (h) | %*F*^c^	2.57 | 2.74 | 1.79 | 13.04
Dog Cl_b_ (L h^–1^ kg^–1^) | *V*_ss,b_ (L/kg) | *t*_1/2_ (h) | %*F*^d^	0.971 | 4.67 | 4.17 | 65.2
Monkey Cl_b_ (L h^–1^ kg^–1^) | *V*_ss,b_ (L/kg) | *t*_1/2_ (h) | %*F*^e^	2.18 | 7.36 | 2.53 | 3.53

a Intrinsic clearance obtained from isolated human hepatocyte cells.

b *t*_1/2_ determined by parent depletion (MS detection) of 5 μM compound concentration in rat whole blood at 37 °C.

c Sprague–Dawley rats (*n* = 3), IV dose = 2 mg/kg using 20% DMSO + 40% PEG400 in water and PO dose = 5 mg/kg using solution of 50 mM citrate buffer, pH = 3.0.

d Beagle dogs (*n* = 3), IV dose = 2 mg/kg using 20% DMSO + 60% PEG400 in water and PO dose = 5 mg/kg using solution of 50 mM citrate buffer, pH = 3.5.

e Cynomologus monkeys (*n* = 3), IV dose = 0.5 mg/kg using 20% DMSO + 60% PEG400 in water and PO dose = 1 mg/kg using solution of 50 mM citrate buffer, pH = 3.0; Cl_b_, blood clearance (Cl_b_ = Cl_p_/blood-to-plasma ratio). *V*_ss,b_, blood volume of distribution at steady-state (*V*_ss,b_ = *V*_ss,p_/blood-to-plasma ratio).

The compelling EGFR ex20ins mutant activity and selectivity vs WT-EGFR observed for **53**, both in vitro and in vivo, combined with acceptable biopharmaceutical properties, favorable human PK projection, and good tolerability observed in preclinical toxicology models, led to our progression of **53** into clinical development as STX-721. STX-721 entered human clinical trials in October 2023 with a phase I/II trial evaluating its safety, tolerability, pharmacokinetic properties, and efficacy in EGFR/HER2 ex20ins mutant tumors (NCT06043817).^61^

## Chemistry

**Scheme 1:**
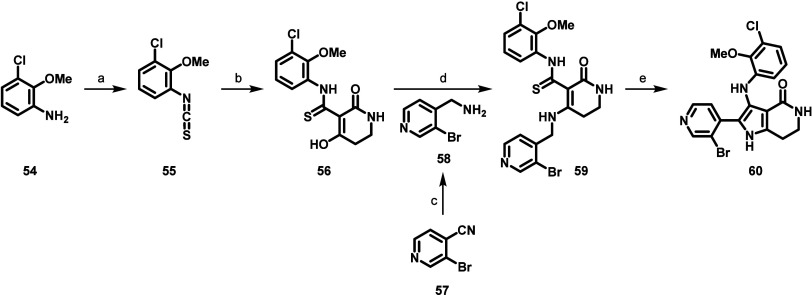
Synthesis of Pyrrololactam Core **60** Reagents and conditions: (a) Thiophosgene, NaHCO_3_, CH_2_Cl_2_, H_2_O, 0 °C to rt, 77%; (b) piperidine-2,4-dione, DBU, MeCN, 0 °C to rt, 69%; (c) Raney nickel, H_2_, MeOH, AcOH, rt, 68%; (d) PyBOP, Et_3_N, DMF, 10 °C to rt, 59%; (e) H_2_O_2_, MeOH, H_2_O, 70 °C, 22%.

The synthesis of advanced pyrrololactam core intermediate **60** (Scheme 1) commenced with a reaction between aniline **54** and thiophosgene under mildly basic conditions to afford isothiocynate **55**. Next, a mixture of **55** and piperidine-2,4-dione was treated with DBU to give enol **56**. (3-bromopyridin-4-yl)methanamine (**58**), which can be prepared in one step from 3-bromopyridine-4-carbonitrile (**57**) via a Raney nickel catalyzed hydrogenation, was combined with enol **56** under amide coupling conditions using PyBop and triethylamine to provide enamine thioamide **59**. Finally, intramolecular oxidative cyclization of **59**, promoted by hydrogen peroxide, resulted in pyrrolactam core intermediate **60**.

**Scheme 2:**
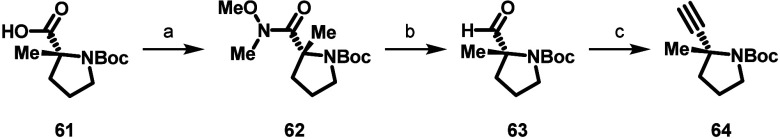
Synthesis of Pyrrolidine Alkyne 64 Reagents and conditions: (a) DMAP, EDCI, *N*,*O*-dimethylhydroxylamine hydrochloride, Py; (b) DIBAL-H, CH_2_Cl_2_, −78 °C followed by MeOH, −78 °C; (c) K_2_CO_3_, 1-diazo-1-dimethoxyphosphoryl-propan-2-one, MeOH, 0 °C to rt, 59% over two steps.

Pyrrolidine alkyne **64** was available from commercially available enantiopure (*R*)-1-(*tert*-butoxycarbonyl)-2-methylpyrrolidine-2-carboxylic acid (**61**) in three steps (Scheme 2). Carboxylic acid **61** was converted to the Weinreb amide **62** under standard amide coupling conditions with EDCI and catalytic DMAP. Amide **62** was then carefully reduced with DIBAL-H at −78 °C and the excess reducing agent was quenched in situ with MeOH to afford aldehyde **63**. Finally, Horner–Wadsworth–Emmons reaction of the aldehyde **63** with 1-diazo-1-dimethoxyphosphoryl-propan-2-one in the presence of K_2_CO_3_ yielded pyrrolidine alkyne **64**.

**Scheme 3:**
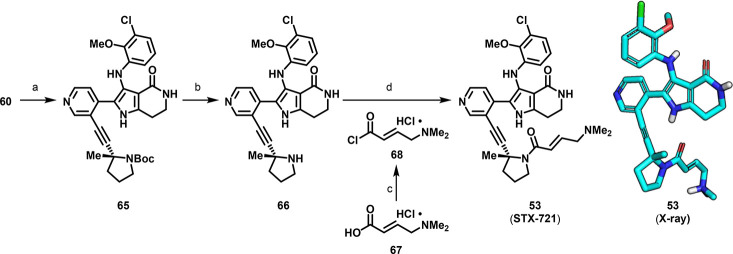
Synthesis of **53** (STX-721) Reagents and conditions: (a) **64**, *N*,*N*-diisopropylethylamine; DMF, CuI, Pd(dppf)Cl_2_·CH_2_Cl_2_, 90 °C, 49%; (b) HCl, 2-MeTHF, H_2_O, 50 °C; (c) oxalyl chloride, THF, DMF (cat.), 5 °C to rt; (d) NaOH, CH_2_Cl_2_, H_2_O, 0 °C to rt, 58% over two steps. Non-nitrogen hydrogens omitted for clarity in the X-ray structure of **53**. X-ray image generated using Maestro (release 2023–3) and visualized in PyMOL v3.0.3.

The final steps of the synthesis sequence began with Sonogashira coupling between pyridyl bromide **60** and alkyne **64** to provide boc-protected pyrrolidine **65**. The boc group was then removed under acidic conditions with HCl and the corresponding amine **66** was treated with (*E*)-4-(dimethylamino)but-2-enoyl chloride hydrochloride (**68**) under Schotten–Baumann conditions to afford STX-721 (Scheme 3).

## Conclusion

Herein we report the medicinal chemistry optimization efforts leading to the discovery of STX-721, an irreversible covalent, potent, and highly selective EGFR/HER2 ex20ins mutant inhibitor for the treatment of NCSLC. Several key concepts and learnings were made during the course of this work. The establishment of isogenic cell-based systems was critical to our ability to reliably measure mutant vs WT selectivity. Using an isogenic Ba/F3 cell-based system, a screening campaign led to the identification of a pyrrololactam core series with inherent EGFR ex20ins mutant selectivity. The use of isogenic models afforded the most consistent and objective method to measure mutant selectivity. Computational studies indicated that selectivity for the series was likely a result of a conformational-dynamic preference for interaction with the αC-helix-in “active state” conformation, enriched in EGFR ex20ins mutants vs WT. Unfortunately, it was challenging to achieve tolerability at efficacious doses in an in vivo mouse allograft tumor model across structurally differentiated examples of this chemical matter. Mouse tolerability was improved by increasing the overall kinome selectivity through the introduction of a covalent acrylamide warhead targeting C797. However, initial analogues within a covalent alkyne-pyrrolidine lead series suffered from poor warhead stability in rat whole blood that translated into rat clearance above hepatic blood flow. Fortunately, both pyrrolidine substitution and acrylamide modification were found to increase rat whole blood stability. Ultimately, a synergistic combination of pyrrolidine and acrylamide substitution led to the identification STX-721 that demonstrated potent EGFR/HER2 ex20ins mutant activity, excellent mutant vs WT and overall kinome selectivity, with favorable rat whole blood stability and PK. Importantly, EGFR WT-sparing for STX-721 could be confirmed first in vitro and then in vivo using a pair of mouse xenograft models developed from NCI-H2073 EGFR WT and isogenic EGFR knock-in ex20ins mutant cell lines. Overall, STX-721 possesses acceptable drug-like properties, a favorable projected human dose, and potentially best-in-class EGFR ex20ins mutant vs WT selectivity. Efforts are currently underway to demonstrate the clinical potential of STX-721.

## Experimental Section

### General Procedures

All materials were obtained from commercial suppliers and used without further purification unless otherwise noted. Anhydrous solvents were obtained from Sigma-Aldrich or WuXi-EHS and used directly. Reactions involving air- or moisture-sensitive reagents were performed under a nitrogen or argon atmosphere. Silica gel chromatography was performed using prepacked silica gel cartridges (Sanpont or Agela-CS). NMR spectra were acquired on Bruker Avance or Quantum-1 Plus 400 MHz spectrometer equipped with 5 mm BBFO probes. Chemical shifts are reported in parts per million (ppm, δ units). All final compounds were purified to >95% purity as determined by liquid chromatography–mass spectrometry (LC–MS) using several instruments and methods: (A) Shimadzu LCMS-2020 system using an ESI model mass spectrometer utilizing ESI ionization fitted with an Advanced Materials Technology HALO C18 column (30 mm × 3.0 mm, 2.0 μm) at 40 °C with a 1.5 mL/min flow rate using a 5–100% gradient of acetonitrile/water with 0.1% formic acid over 1.5 min. (B) Shimadzu LCMS-2020 system using a SCAN model mass spectrometer utilizing ES-API ionization fitted with an Advanced Materials Technology Halo 90A C18 column (30 mm × 3.0 mm, 5.0 μm) at 50 °C with a 1.5 mL/min flow rate using a 5–95% gradient of acetonitrile/water with 0.04% trifluoracetic acid over 1.0 min. (C) Agilent 1260–6025 Halo 90A C18 column (30 mm × 3.0 mm, 5.0 μm) at 50 °C with a 1.5 mL/min flow rate using a 5–95% gradient of acetonitrile/water with 0.04% trifluoracetic acid over 1.0 min. (D) Shimadzu LCMS-2020 Kinetex EVO C18 column (30 mm × 2.1 mm, 5.0 μm) at 50 °C with a 1.5 mL/min flow rate using a 5–95% gradient of acetonitrile/water with 0.025% NH_3_·H_2_O over 1.5 min.

### Synthesis of STX-721 (**53**), (*R*,*E*)-3-((3-chloro-2-methoxyphenyl)amino)-2-(3-((1-(4-(dimethylamino)but-2-enoyl)-2-methylpyrrolidin-2-yl)ethynyl)pyridin-4-yl)-6,7-dihydro-1*H*-pyrrolo[3,2-*c*]pyridin-4(5*H*)-one

#### 1-(3-Bromopyridin-4-yl)methanamine (**58**)

Raney Nickel (50 g, 584 mmol) was added to 3-bromopyridine-4-carbonitrile (500 g, 2732 mmol, **57**) in MeOH (5 L) and AcOH (500 mL) under a N_2_ atmosphere. The reaction vessel was purged with H_2_ and the reaction mixture was stirred under a H_2_ atmosphere at rt for 16 h, filtered through Celite, and the filtrate was concentrated under reduced pressure. The residue was resuspended in DCM (500 mL), filtered, and the filter cake was dissolved in MeOH (500 mL) and H_2_O (500 mL) before NaOH (110 g) was introduced. The resultant mixture was concentrated under reduced pressure, then diluted with DCM (1 L). The resultant mixture was filtered, and the filter cake was washed with DCM (100 mL). The filter cake was dried under reduced pressure to afford 1-(3-bromopyridin-4-yl)methanamine (345 g, 68%) as a yellow solid. ^1^H NMR (400 MHz, CDCl_3_) δ = 8.64 (s, 1H), 8.49 (d, *J* = 4.9 Hz, 1H), 7.41 (d, *J* = 4.9 Hz, 1H), 3.93 (s, 2H). *m*/*z* (ESI, +ve ion): 187.1 (M + H)^+^.

#### 1-Chloro-3-isothiocyanato-2-methoxybenzene (**55**)

Thiophosgene (368 g, 3198 mmol) was added dropwise to 3-chloro-2-methoxyaniline (480 g, 3046 mmol, **54**) and NaHCO_3_ (281 g, 3350 mmol) in DCM (2.4 L) and H_2_O (2.4 L) at 0 °C under a N_2_ atmosphere. The resulting mixture was stirred for 2 h at rt before ice-cold saturated aqueous NaHCO_3_ solution (2 L) was introduced. The layers were separated and the aqueous layer was extracted with DCM (2 × 2 L). The combined organic layers were washed with brine (3 L), dried over anhydrous Na_2_SO_4_, filtered, and concentrated under reduced pressure to afford 1-chloro-3-isothiocyanato-2-methoxybenzene (470 g, 77% yield) as a light-brown oil, which was used without further purification. ^1^H NMR (400 MHz, CDCl_3_) δ = 7.28 (dd, *J* = 7.8, 1.9 Hz, 1H), 7.04 (dd, *J* = 8.1, 1.9 Hz, 1H), 7.00 (t, *J* = 7.9 Hz, 1H), 3.95 (s, 3H). *m*/*z* (ESI, +ve ion): 385.1 (M + H)^+^.

#### *N*-(3-Chloro-2-methoxyphenyl)-4-hydroxyl-2-oxo-5,6-dihydro-1*H*-pyridine-3-carbothioamide (**56**)

DBU (430 g, 2825 mmol) was added dropwise to 1-chloro-3-isothiocyanato-2-methoxybenzene (470 g, 2354 mmol, **55**) and piperidine-2,4-dione (293 g, 2590 mmol) in MeCN (4.7 L) at 0 °C under a N_2_ atmosphere. The resulting mixture was stirred for 3 h at rt before ice-cold water (2 L) was introduced. The resulting mixture was extracted with EtOAc (2 × 3 L). The combined organic layers were washed with brine (3 L), dried over anhydrous Na_2_SO_4_, filtered, and concentrated under reduced pressure. The residue was resuspended in EtOAc (1 L), filtered, and the filter cake further dried under reduced pressure to afford *N*-(3-chloro-2-methoxyphenyl)-4-hydroxyl-2-oxo-5,6-dihydro-1*H*-pyridine-3-carbothioamide (510 g, 69%) as a yellow solid, which was used without further purification. *m*/*z* (ESI, +ve ion): 313.1 (M + H – Boc)^+^.

#### 4-(((3-Bromopyridin-4-yl)methyl)amino)-*N*-(3-chloro-2-methoxyphenyl)-2-oxo-5,6-dihydro-1*H*-pyridine-3-carbothioamide (**59**)

Benzotriazol-1-yl-oxy-tris-pyrrolidino-phosphonium hexafluorophosphate (PyBOP) (1273 g, 2446 mmol) and triethylamine (330 g, 3261 mmol) were added portion-wise to a solution of *N*-(3-chloro-2-methoxyphenyl)-4-hydroxy-2-oxo-5,6-dihydro-1*H*-pyridine-3-carbothioamide (510 g, 1631 mmol, **56**), 1-(3-bromopyridin-4-yl)methanamine (335 g, 1794 mmol, **58**), in DMF (5.1 L) at 10 °C under a N_2_ atmosphere. The resulting mixture was stirred for 16 h at rt, before ice-cold water (3 L) was introduced. The resulting mixture was extracted with EtOAc (2 × 3 L). The combined organic layers were washed with brine (2 × 3 L), dried over anhydrous Na_2_SO_4_, filtered, and concentrated under reduced pressure, and filtered through a silica gel plug (eluent: 67% EtOAc/petroleum ether). The residue was resuspended in EtOAc (500 mL) and filtered to afford 4-(((3-bromopyridin-4-yl)methyl)amino)-*N*-(3-chloro-2-methoxyphenyl)-2-oxo-5,6-dihydro-1*H*-pyridine-3-carbothioamide (460 g, 59%) as a light-yellow solid that was used without further purification. ^1^H NMR (400 MHz, DMSO-*d*_6_) δ = 13.81–12.56 (m, 2H), 8.91–8.67 (m, 1H), 8.64–8.51 (m, 1H), 7.92–7.69 (m, 1H), 7.51–7.42 (m, 1H), 7.40–7.33 (m, 1H), 7.22–7.11 (m, 1H), 4.86–4.65 (m, 2H), 3.77 (s, 3H), 3.70–3.62 (m, 2H), 2.92–2.78 (m, 2H), 1.47 (s, 9H). *m*/*z* (ESI, +ve ion): 481.0 (M + H – Boc)^+^.

#### 2-(3-Bromopyridin-4-yl)-3-[(3-chloro-2-methoxyphenyl)amino]-1*H*,5*H*,6*H*,7*H*-pyrrolo[3,2-*c*]pyridin-4-one (**60**)

Hydrogen peroxide (30 wt % in H_2_O, 146 mL, 1432 mmol) was added dropwise to a solution of 4-(((3-bromopyridin-4-yl)methyl)amino)-*N*-(3-chloro-2-methoxyphenyl)-2-oxo-5,6-dihydro-1*H*-pyridine-3-carbothioamide (460 g, 955 mmol, **59**) in MeOH (4.6 L). The resulting mixture was stirred at 70 °C under a N_2_ atmosphere. After 16 h, the reaction mixture was allowed to cool to rt before it was partially concentrated under reduced pressure to approximately 100 mL total volume. A saturated aqueous solution of Na_2_SO_3_ (100 mL) was introduced and the resulting mixture was extracted with DCM (3 × 500 mL). The combined organic layers were dried over anhydrous Na_2_SO_4_, filtered, and concentrated under reduced pressure. The residue was filtered through a silica gel plug (eluent: 100% EtOAc) and the filtrate was concentrated under reduced pressure. The residue was resuspended in MeCN (200 mL) and stirred at ambient temperature. After 1 h, the resulting precipitate was filtered to afford 2-(3-bromopyridin-4-yl)-3-[(3-chloro-2-methoxyphenyl)amino]-1*H*,5*H*,6*H*,7*H*-pyrrolo[3,2-*c*] pyridin-4-one (100 g, 22%) as a light-brown solid. ^1^H NMR (400 MHz, DMSO-*d*_6_) δ = 11.76–11.48 (m, 1H), 8.70 (s, 1H), 8.53–8.30 (m, 1H), 7.55–7.31 (m, 2H), 7.23–6.99 (m, 1H), 6.59 (br d, *J* = 1.3 Hz, 2H), 6.18–5.94 (m, 1H), 3.96–3.65 (m, 3H), 3.51–3.39 (m, 2H), 2.94–2.74 (m, 2H). *m*/*z* (ESI, +ve ion): 449.0 (M + H)^+^.

#### *tert*-Butyl (*R*)-2-(Methoxy(methyl)carbamoyl)-2-methylpyrrolidine-1-carboxylate (**62**)

DMAP (13.3 g, 109 mmol) and EDCI (157 g, 818 mmol) were added to a solution of (*R*)-1-(*tert*-butoxycarbonyl)-2-methylpyrrolidine-2-carboxylic acid (125 g, 545 mmol, **61**) and *N*,*O*-dimethylhydroxylamine hydrochloride (160 g, 1640 mmol) in pyridine (2 L) at rt. After the addition was complete, the resultant reaction mixture was warmed to 40 °C for 2 h. An aqueous solution of HCl (1 N, 3 L) was introduced and extracted with EtOAc (3 × 1 L). The combined organic layers were washed with brine (1 L), dried over anhydrous Na_2_SO_4_, filtered, and concentrated under reduced pressure to afford *tert*-butyl (*R*)-2-(methoxy(methyl)carbamoyl)-2-methylpyrrolidine-1-carboxylate (281 g, crude) as a yellow oil, which was used without further purification. *m*/*z* (ESI, +ve ion): 173.2 (M + H)^+^.

#### *tert*-Butyl (*R*)-2-Ethynyl-2-methylpyrrolidine-1-carboxylate (**64**)

Diisobutylaluminum hydride solution in DCM (1.0 M, 514 mL) was added dropwise to a solution of *tert*-butyl (*R*)-2-(methoxy(methyl)carbamoyl)-2-methylpyrrolidine-1-carboxylate (281 g, **62**) in DCM (500 mL) at −78 °C over 2 h under a N_2_ atmosphere. The resultant reaction mixture was stirred for an additional 30 min at −78 °C before MeOH (250 mL) was introduced dropwise at −78 °C over 2 h. A mixture of potassium carbonate (71.3 g, 516 mmol) and 1-diazo-1-dimethoxyphosphoryl-propan-2-one (74.3 g, 387 mmol) in MeOH (500 mL) was then added to the reaction mixture at 0 °C. The resultant reaction mixture was allowed to warm to rt over 12 h. A saturated aqueous solution of potassium sodium tartrate (500 mL) and EtOAc (500 mL) were introduced and the resultant mixture was stirred for 2 h. The mixture was filtered and the filtrate was concentrated under reduced pressure. The residue was diluted with H_2_O (500 mL) and extracted with EtOAc (3 × 800 mL). The combined organic layers were washed with brine (3 × 1 L), dried over anhydrous Na_2_SO_4_, filtered, and concentrated under reduced pressure. Chromatographic purification of the residue (silica gel, 0–5% EtOAc/petroleum ether) afforded *tert*-butyl (*R*)-2-ethynyl-2-methylpyrrolidine-1-carboxylate (130 g, 59% yield) as a yellow oil. ^1^H NMR (400 MHz, CDCl_3_) δ = 3.47 (br d, *J* = 2.9 Hz, 1H), 3.38–3.25 (m, 1H), 2.28–2.12 (m, 2H), 1.97–1.79 (m, 2H), 1.78–1.66 (m, 1H), 1.56 (s, 3H), 1.41 (s, 9H). *m*/*z* (ESI, +ve ion): 154.1 (M + H)^+^.

#### *tert*-Butyl(2*R*)-2-[2-[4-[3-(3-chloro-2-methoxy-anilino)-4-oxo-1,5,6,7-tetrahydropyrr-olo[3,2-*c*]pyridin-2-yl]-3-pyridyl]ethynyl]-2-methyl-pyrrolidine-1-carboxylate (**65**)

*N*,*N*-Diisopropylethylamine (147 mL, 844 mmol) was added to a solution of *tert*-butyl (2*R*)-2-ethynyl-2-methyl-pyrrolidine-1-carboxylate (70.6 g, 337 mmol, **64**) and 2-(3-bromo-4-pyridyl)-3-(3-chloro-2-methoxy-anilino)-1,5,6,7-tetrahydropyrrolo[3,2-*c*]pyridin-4-one (126 g, 281 mmol, **60**) in DMF (630 mL) under a N_2_ atmosphere. The resultant reaction mixture was sparged with N_2_ for 10 min before copper iodide (2.68 g, 14.0 mmol) and [1,1′-bis(diphenylphosphino)ferrocene]dichloropalladium(II), complex with dichloromethane (22.9 g, 28.1 mmol) was introduced and the resulting mixture was stirred at 90 °C. After 16 h, ice-cold water (3.2 L) was introduced and the resulting mixture was extracted with EtOAc (2 × 3.2 L). The combined organic layers were washed with H_2_O (2 × 6.4 L) and brine (6.4 L), dried over anhydrous Na_2_SO_4_, filtered and concentrated under reduced pressure. The residue was filtered through a silica gel plug (eluent: 100% EtOAc) and the filtrate was concentrated under reduced pressure. The residue was resuspended in EtOAc (300 mL) and stirred at 90 °C. After 1 h, the mixture was allowed to cool to rt and then placed in a refrigerator at 4 °C. After 16 h, the mixture was filtered and the filter cake was washed with ice-cold EtOAc (100 mL) and cyclohexane (2 × 100 mL) to afford *tert*-butyl(2*R*)-2-[2-[4-[3-(3-chloro-2-methoxy-anilino)-4-oxo-1,5,6,7-tetrahydropyrr-olo[3,2-*c*]pyridin-2-yl]-3-pyridyl]ethynyl]-2-methyl-pyrrolidine-1-carboxylate (82.5 g, 49% yield) as a yellow solid. ^1^H NMR (400 MHz, DMSO-*d*_6_) δ = 11.55–11.23 (m, 1H), 8.55 (s, 1H), 8.42–8.15 (m, 1H), 7.62 (s, 1H), 7.33 (d, *J* = 11.2 Hz, 1H), 7.26–7.05 (m, 1H), 6.73–6.50 (m, 2H), 6.12 (d, *J* = 6.0 Hz, 1H), 3.96–3.76 (m, 3H), 3.44 (d, *J* = 6.0 Hz, 4H), 2.98 (s, 1H), 2.81 (s, 1H), 2.38 (s, 1H), 2.12–2.01 (m, 1H), 1.95–1.75 (m, 2H), 1.65 (s, 3H), 1.45–1.27 (m, 9H). *m*/*z* (ESI, +ve ion): 576.4 (M + H)^+^.

#### 3-(3-Chloro-2-methoxy-anilino)-2-[3-[2-[(2*R*)-2-methylpyrrolidin-2-yl]ethynyl]-4-pyridyl]-1,5,6,7-tetrahydropyrrolo[3,2-*c*]pyridin-4-one (**66**)

Hydrochloric acid (12.0 M, 141 mL, 1700 mmol) was added dropwise to a solution of *tert*-butyl (2*R*)-2-[2-[4-[3-(3-chloro-2-methoxy-anilino)-4-oxo-1,5,6,7-tetrahydropyrrolo[3,2-*c*]pyridin-2-yl]-3-pyridyl]ethynyl]-2-methyl-pyrrolidine-1-carboxylate (98.0 g, 170 mmol, **65**) in 2-methyltetrahydrofuran (980 mL) at rt. The resulting mixture was stirred at 50 °C for 2 h, allowed to cool to rt, then concentrated under reduced pressure. The residue was diluted with MeOH (500 mL) and H_2_O (1 L) and cooled to 0 °C before the pH of the mixture was adjusted to pH = 8–9 with an aqueous solution of NaHCO_3_. The resulting mixture was diluted with DCM (2000 mL) and MeOH (500 mL). The layers were separated, and the aqueous layer was extracted with DCM (2 × 2 L). The combined organic layers were washed with brine (6 L) and dried over anhydrous Na_2_SO_4_, filtered, and concentrated under reduced pressure to afford 3-(3-chloro-2-methoxy-anilino)-2-[3-[2-[(2*R*)-2-methylpyrrolidin-2-yl] ethynyl]-4-pyridyl]-1,5,6,7-tetrahydropyrrolo[3,2-*c*]pyridin-4-one (84 g, crude) as a yellow solid, which was used without further purification. *m*/*z* (ESI, +ve ion): 476.3 (M + H)^+^.

#### (*E*)-4-(Dimethylamino)but-2-enoyl Chloride Hydrochloride (**68**)

Oxalyl chloride (70.3 mL, 803 mmol) was added dropwise to a solution of (*E*)-4-(dimethylamino)but-2-enoic acid hydrochloride (100 g, 604 mmol, **67**) in THF (1000 mL) at 5 °C. DMF (1.0 mL) was introduced and the resultant reaction mixture was stirred at rt. After 3 h, the reaction mixture was cooled to 0 °C and filtered under a N_2_ atmosphere. The filter cake was washed with ice-cold THF (3 × 500 mL) and the filter cake was dried under reduced pressure to afford (*E*)-4-(dimethylamino)but-2-enoyl chloride hydrochloride (210 g, crude) as a yellow solid, which was used without further purification.

#### (*R*,*E*)-3-((3-Chloro-2-methoxyphenyl)amino)-2-(3-((1-(4-(dimethylamino)but-2-enoyl)-2-methylpyrrolidin-2-yl)ethynyl)pyridin-4-yl)-1,5,6,7-tetrahydro-4*H*-pyrrolo[3,2-*c*]pyridin-4-one (**STX-721**)

(*E*)-4-(Dimethylamino)but-2-enoyl chloride hydrochloride (37.7 g, 205 mmol, **68**) was added portionwise to a mixture of (*R*)-3-((3-chloro-2-methoxyphenyl)amino)-2-(3-((2-methylpyrrolidin-2-yl)ethynyl)pyridin-4-yl)-6,7-dihydro-1*H*-pyrrolo[3,2-*c*]pyridin-4(5*H*)-one (65.0 g, 137 mmol, **66**) and NaOH (10 M, 137 mL, 1370 mmol) in DCM (1.3 L) at 0 °C. The resultant reaction mixture was allowed to warm to ambient temperature over 2 h. Water was introduced (1.3 L) and the mixture was extracted with DCM (2 × 1.3 L). The combined organic layers were washed with brine (2.6 L) and dried over anhydrous Na_2_SO_4_, filtered, and concentrated under reduced pressure. The residue was resuspended in MeOH (75 mL) and stirred at rt. After 1 h, the precipitate was filtered, the filter cake was washed with ice-cold MeOH (50 mL) and cyclohexane (2 × 50 mL), and further dried under reduced pressure to afford (*R*,*E*)-3-((3-chloro-2-methoxyphenyl)amino)-2-(3-((1-(4-(dimethylamino)but-2-enoyl)-2-methylpyrrolidin-2-yl)ethynyl)pyridin-4-yl)-6,7-dihydro-1*H*-pyrrolo[3,2-*c*]pyridin-4(5*H*)-one (**STX-721**; 47.0 g, 58% yield) as an off-white solid. ^1^H NMR (400 MHz, CDCl_3_) δ = 11.52 (s, 1H), 8.52 (s, 1H), 8.14 (d, *J* = 5.6 Hz, 1H), 7.67 (s, 1H), 7.40 (d, *J* = 5.6 Hz, 1H), 7.01–6.83 (m, 1H), 6.75–6.68 (m, 1H), 6.67–6.56 (m, 1H), 6.32 (d, *J* = 15.2 Hz, 1H), 6.25 (dd, J = 8.0, 1.2 Hz, 1H), 5.37 (s, 1H), 4.08 (s, 3H), 3.87–3.68 (m, 2H), 3.65–3.56 (m, 2H), 3.37–3.15 (m, 2H), 3.14–3.05 (m, 2H), 2.58–2.43 (m, 1H), 2.27 (s, 6H), 2.21–2.06 (m, 3H), 1.77 (s, 3H). *m*/*z* (ESI, +ve ion): 587.5 (M + H)^+^.

## Supporting Information Available

Experimental details for in vitro and in vivo studies; additional in vivo data for compounds **30**, **32**, **33**, **34**, **35**, and **53**; crystallographic data for compounds **23**, **32**, **39**, and **53**; synthetic procedures and characterization for compounds **1**–**53** and **S1**–**S10**; computational models for compounds **1** and **23** (PDF)
Molecular formula strings and including some data (CSV)

## Accession Codes

Atomic coordinates for the X-ray structures of compound **23** (PDB 9FQP) and compound **39** (PDB 9FQS) bound to EGFR exon20insNPG protein and compound **23** (PDB 9FRD) bound to EGFR WT protein are available from the RCSB Protein Data Bank (www.rcsb.org). Authors will release the atomic coordinates upon article publication.

## Abbreviations Used

AcOH: acetic acid
Caco-2: human colon carcinoma cell line
ChEF: chelation enhanced fluorescence
Cl_b_: in vivo blood clearance
Cl_p_: in vivo plasma clearance
CTG: CellTiter-Glo
CYP: cytochrome P450
DBU: 1,8-diazabicyclo(5.4.0)undec-7-ene
DCM: dichloromethane
DIBAL-H: diisobutylaluminum hydride
DMAP: 4-(dimethylamino)pyridine
DMF: *N*,*N*-dimethylformamide
DMSO: dimethyl sulfoxide
dppf: 1,1′-bis(diphenylphosphino)ferrocene
EDCI: 1-ethyl-3-(3-(dimethylamino)propyl) carbodiimide
EGFR: epidermal growth factor receptor
*F*: bioavailability
FaSSGF: fasted-state simulated gastric fluid
FaSSIF: fasted-state simulated intestinal fluid
GSH: glutathione
HER2: receptor tyrosine-protein kinase ERBB2
hERG: human ether-à-go-go-related gene
HLM: human liver microsomes
HPMC: hydroxypropyl methylcellulose
IV: intravenous
MDCK: Madin–Darby canine kidney cell
MeCN: acetonitrile
MeOH: methanol
2-MeTHF: 2-methyltetrahydrofuran
MS: mass spectrometry
p-EGFR or p-ERK: phosphorylated extracellular signal-regulated kinase
PBS: phosphate-buffered saline
PEG: polyethylene glycol
PK: pharmacokinetic
PO: per os (oral)
Py: pyridine
PyBOP: benzotriazol-1-yloxytripyrrolidinophosphonium hexafluorophosphate
QD: quaque die (once daily)
*Q*_H_: hepatic blood flow rate
rt: room temperature
SAR: structure–activity relationship
*t*_1/2_: half-life
*V*_ss_: volume of distribution measured at steady-state
XRC: X-ray crystal
