# Supplementary material for: Discovery of STX-721, a Covalent, Potent, and Highly Mutant-Selective EGFR/HER2 Exon20 Insertion Inhibitor for the Treatment of Non-Small Cell Lung Cancer
Source: J Med Chem. 2025 Jan 17;68(3):2403–21. doi: 10.1021/acs.jmedchem.4c02377 (PMC11831596; doi:10.1021/acs.jmedchem.4c02377)
Supplement: Supplementary file 1 — jm4c02377_si_001.pdf [file jm4c02377_si_001.pdf]

Supplementary Information

Discovery of STX-721, a Covalent, Potent, and Highly Mutant-Selective EGFR/HER2 Exon20 Inhibitor for the Treatment of Non-Small Cell Lung Cancer

Benjamin C. Milgram,[*,†] Deanna R. Borrelli,[†] Natasja Brooijmans,[†] Jack A. Henderson,[†] Brendan J. Hilbert,[†] Michael R. Huff,[†] Takahiro Ito,[‡] Erica L. Jackson,[†] Philip Jonsson,[Δ] Brendon Ladd,[†] Erin L. O'Hearn,[†] Raymond A. Pagliarini,[†] Simon A. Roberts,[†] Sébastien Ronseaux,[†] Darrin D. Stuart,[†] Weixue Wang,[†] Angel Guzman-Perez[†]

[*]corresponding author

[†]Scorpion Therapeutics, 1 Winthrop Square, Boston, Massachusetts 02110, United States of America

[‡]Current address: Odyssey Therapeutics, San Diego, California 92121, United States of America

[Δ]Current address: Loxo Oncology at Lilly, Lilly Corporate Center, Indianapolis, Indiana 46285, United States of America

Corresponding Author
Benjamin C. Milgram — *Scorpion Therapeutics, Boston, Massachusetts 02110, United States of America*
ORCID iD 0009-0008-5824-4645
Email: ben@scorpiontx.com

Table of Contents

Methods 3
Recombinant proteins 3
Cell lines 4
Cell proliferation assays 4
k_{inact}/K_I determination 4
MDCK permeability assay 5
Caco-2 permeability assay 5
HT solubility assay 6
Plasma protein binding assay 7
Whole blood portioning ratio 7
Whole blood stability assay 8
GSH stability assay 8
Hepatocyte stability assay 8
Animal studies 8
Ba/F3 allograft and NCI-H2073 xenograft studies 9

Pharmacokinetic and tumor pharmacodynamic assays 9
Protein crystallization 9
Structure determination and refinement 10
Crystallography references 10
Procedures for the syntheses of STX-721 and related analogs 11
General synthetic procedures 11
Compound 1 11
Compound S1 12
Compound 2 12
Compound 3 13
Compound 4 13
Compound 5 14
Compound 6 14
Compound 7 15
Compound 8 16
Compound 9 17
Compound 10 18
Compound 11 18
Compound 12 18
Compound 13 18
Compound 14 20
Compound 15 20
Compound 16 20
Compound 17 20
Compound 18 20
Compound 19 20
Compounds 20 and 21 21
Compounds 22 and 23 22
Compounds 24 and 25 23
Compound 26 23
Compounds 27 and S2 24
Compounds S3 and 28, S4, and S5 24
Compounds 29 and S6 27
Compounds 30 and S7 29
Compounds 31 and S8 30
Compounds 32 and S9 33
Compound 33 34
Compound 34 35
Compound 35 37

Compounds 36 and 37 39
Compound 38........ 41
Compound 39........ 43
Compound 40........ 43
Compounds 41 and 42 43
Compounds 43 and S10 44
Compound 44........ 44
Compound 45........ 46
Compound 46........ 47
Compound 47........ 47
Compound 48........ 47
Compound 49........ 47
Compound 50........ 47
Compound 51........ 48
Compound 52........ 49
Additional data for compounds with arbitrarily defined stereochemistry 49
Biochemical activity and selectivity data for compounds 38 & 53 (STX-721)........ 51
Comprehensive Ba/F3, NCI-H2073, and NCI-H1975 cell antiproliferative activity and selectivity data........ 51
Additional mouse in vivo Ba/F3 EGFR exon20insASV allograft data 60
HPLC purity data 61
Docking calculations for compound 1 62
Compound 23 WaterMap with EGFR WT and ex20insNPG protein........ 63
Analytical data for key compounds 64
X-ray crystallographic data........ 78
X-ray co-crystal structures of compounds 23 & 39........ 78
X-ray structure determination of compound 32........ 79
X-ray structure determination of STX-721........ 80
References........ 82

Methods

Recombinant proteins

The NPG EGFR (695-770_N771insNPG-1022, V948R) construct with a C-terminal hexahistidine tag was expressed in *Sf21* cells and purified by Ni^{2+}-affinity chromatography, dephosphorylation with lambda phosphatase and size-exclusion chromatography. The WT kinase EGFR kinase domain (696-1022) was fused to an N-terminal Thrombin-cleavable GST fusion tag. The protein was produced as previously published.[1] In brief, the gene was expressed in baculovirus-infected insect cells, and the protein was purified by a three-step procedure comprising affinity and size exclusion chromatography steps. Protein for crystallization was concentrated to 4-8 mg/mL in crystallization buffer (0.1 mM Benzamidine, 4 mM DTT, 20 mM TRIS pH 8). NPG V948R mutant protein (V951R in D770_N771 insNPG mutant numbering) was prepared and employed to facilitate crystallization efforts.

Cell lines

Ba/F3 cells were purchased from Cobioer (Nanjing, China). Ba/F3 cells were grown in RPMI media supplemented with 10% fetal bovine serum (Fisher) and 10 ng/mL recombinant mouse IL-3 (R&D Systems), and grown at 37 °C with 5% CO_2 in a humidified incubator. EGFR and HER2 construct engineering was performed by transducing parental Ba/F3 with retroviral constructs expressing the noted EGFR or HER2 variants, and selecting in the absence of IL-3; and additionally for the EGFR wild-type-transduced cell line only, in the presence of 50 ng/mL recombinant human EGF (Gibco). The human NSCLC cancer cell lines NCI-H1975 and NCI-H2073 (ATCC) were grown in RPMI media supplemented with 10% FBS (Fisher Scientific). Parental cell identity was verified by STR profiling, and by directed Sanger sequencing of inserted EGFR constructs.

Cell proliferation assays

Cells were plated into 384 well plates in a 50 μL culture volume and incubated at 37°C with 5% CO_2 in a tissue culture incubator overnight. The following day cells were treated with compounds via acoustic liquid dispensing (Echo 550), to generate a 10-point dose response curve with a top final concentration of 10 μM and 1:3 serial dilutions. Following dosing, the compound-treated cell plates were incubated at 37°C with 5% CO_2 in a tissue culture incubator for 72 hours. Following 72 hours of compound treatment, CellTiter-Glo (Promega) reagent was equilibrated to room temperature and added at a volume of 25 μL/well. Plates were placed on an orbital shaker at 300-500 rpm for 10 minutes in the dark at room temperature, followed by an additional 20 minutes not shaking, before reading luminescence on an Envision using the USLum-US LUM 384 (cps) DC filter. Data was processed using Scinamic software, normalized to positive (media only) and negative (cells with DMSO treatment only) controls. A four-paramater logistic fit was used to generate a fitted dose response curve and calculate the concentration required for 50% inhibition (IC_{50}). On graphed data, selectivity for a given exon 20 mutant versus wild-type EGFR was calculated according to the following equation:

$$Fold\ mutant\ selectivity = \frac{[WT\ IC50\ geometric\ mean]}{[MUT\ IC50\ geometric\ mean]}$$

Upper and lower 95% confidence intervals were calculated using the below equations:

$$Upper\ confidence\ interval =\ 10^{(log_{10}(Fold\ mutant\ selectivity)\ +1.96(log_{10}SE))}$$

$$Lower\ confidence\ interval =\ 10^{(log_{10}(Fold\ mutant\ selectivity) -1.96(log_{10}SE))}$$

where,

$$log_{10}\ (Fold\ mutant\ selectivity) =\ log_{10}\ (WT\ IC50\ geometric\ mean) -\ log_{10}\ (MUT\ IC50\ geometric\ mean)$$

and

$$log_{10}SE = \sqrt{\frac{(log_{10}(WT\ IC50\ geometric\ std.dev))^{\ 2}}{\#\ WT\ experimental\ repeats} + \frac{(log_{10}(MUT\ IC50\ geometric\ std.dev))^{\ 2}}{\#\ MUT\ experimental\ repeats}}$$

k_{inact}/K_I determination

Biochemical potency k_{inact}/K_I was determined using chelation-enhanced fluorescence (ChEF) detection.[2, 3] AQT0001 peptide substrate was obtained from AssayQuant technologies, Inc. Proteins were obtained from SignalChem: GST-tagged wild-type EGFR (668-end) (catalog number E10-112G) and GST-tagged EGFR D770_N771 insNPG mutant (668-end) (catalog number E10-132GG. Briefly, compound stock solutions in DMSO were dispensed onto the assay plate by an HP D300 Digital Dispenser. 10μL/well 2X peptide substrate solution and 10μL/well 2X solution with enzyme and ATP were then sequentially added by Multidrop Combi. Both solutions were prepared in the assay buffer contained 50mM HEPES pH7.5, 10mM $MgCl_2$, 0.01% Brij-35, 0.5mM EGTA and 1mM DTT. The final peptide substrate concentration was 10μM; the final enzyme concentration was 10nM for wild-type EGFR and 2nM for the D770_N771 insNPG mutant; the final ATP concentration was 200μM for the wild-type and 800μM for the mutant. The assay plate was sealed and centrifuged for 1 minutes at 1000rpm. Fluorescence intensity (360nm excitation and 485nm emission) was recorded every 15 seconds for 1 hour by a PHERAstar plate reader.

Data were analyzed by the Dynafit software.[4,5] To determine the k_{inact}/K_I value, enzyme reaction time course data at varying compound concentrations were globally fit into differential equations that describes a rapid-equilibrium two-step enzyme inactivation mechanism.[4] This procedure yielded k_{inact}/K_I values at ATP concentrations used in the experiments. For ATP-competitive compounds, k_{inact}/K_I values at any given ATP concentration can be related to $(k_{inact}/K_I)_{true}$ using the following equation: $k_{inact}/K_I = (k_{inact}/K_I)_{true} / (1+[ATP]/K_{M,ATP})$, where $(k_{inact}/K_I)_{true}$ is the k_{inact}/K_I value in the absence of ATP, and $K_{M, ATP}$ was 18 μM for the recombinant wild-type EGFR, and 83 μM for the recombinant D770_N771 insNPG mutant EGFR used in the study. Using this equation, k_{inact}/K_I values in the absence of ATP and in the presence of 1 mM ATP were calculated from the k_{inact}/K_I values determined from experiments.

MDCK permeability assay

50 μL and 25 mL of cell culture medium were added to a transwell insert and reservoir, respectively. The HTS transwell plates were incubated at 37 °C, 5% CO_2 for 1 h before cell seeding. MDCK cells were diluted to 1.56×10^6 cells/mL with culture medium and 50 μL of cell suspension were dispensed into the filter well of a 96-well HTS transwell plate. Cells were cultivated for 4-8 days in a cell culture incubator at 37 °C, 5% CO_2, 95% relative humidity. Cell culture medium was replaced every other day, beginning no later than 24 h after initial plating. 10 mM stock solutions of test compounds and controls were prepared in DMSO. Metoprolol, prazosin and imatinib were used as control compounds in this assay. The MDCK plate was removed from the incubator and washed twice with pre-warmed HBSS (10 mM HEPES, pH 7.4), and then incubated at 37 °C for 30 minutes. The stock solutions of test compounds were diluted in DMSO to get 0.2 mM solutions and then diluted with HBSS (10 mM HEPES, pH 7.4 with 3%BSA) with 5 μM PSC833 to get 1 μM working solutions. The stock solutions of controls were diluted in DMSO to get 0.2 mM solutions and then diluted with HBSS (10 mM HEPES, pH 7.4) with 5 μM PSC833 to get 1 μM working solutions. The final concentration of DMSO in the incubation system was 0.5%. To determine the rate of drug transport in the apical to basolateral direction. 125 μL of 1 μM working solution of control compounds were added to the transwell insert (apical compartment) and a 50 μL sample (Donor T=0 min sample) was transferred immediately from the apical compartment to a new 96-well plate. The wells of the receiver plate (basolateral compartment) were filled with 235 μL of HBSS (10 mM HEPES, pH 7.4) and 5 μM PSC833. 125 μL of 1 μM working solution of test compounds was added to the transwell insert (apical compartment) and a 50 μL sample (D0 sample) was transferred immediately from the apical compartment to a new 96-well plate. The wells in the receiver plate (basolateral compartment) were filled with 235 μL of HBSS (10 mM HEPES, pH 7.4 with 3%BSA) with 5 μM PSC833. To determine the rate of drug transport in the basolateral to apical direction. 285 μL of 1 μM working solution of control compounds were added to the receiver plate wells (basolateral compartment) and a 50 μL sample (D0 sample) was transferred immediately from the basolateral compartment to a new 96-well plate. The wells in the transwell insert (apical compartment) were filled with 75 μL of HBSS (10 mM HEPES, pH 7.4) and 5 μM PSC833. 285 μL of 1 μM working solution of test compounds were added to the receiver plate wells (basolateral compartment) and a 50 μL sample (D0 sample) was transferred immediately from the basolateral compartment to a new 96-well plate. The wells in the transwell insert (apical compartment) were filled with 75 μL of HBSS (10 mM HEPES, pH 7.4 with 3%BSA) and 5 μM PSC833.The assay was performed in duplicate. The plates were incubated at 37 °C for 2 h. At the end of the incubation, 50 μL samples from donor sides (apical compartment for Ap→Bl flux, and basolateral compartment for Bl→Ap) and receiver sides (basolateral compartment for Ap→Bl flux, and apical compartment for Bl→Ap) were transferred to wells of a new 96-well plate, followed by the addition of 4 volume of cold methanol containing appropriate internal standards (IS). Samples were vortexed for 5 min and then centrifuged at 3,220 g for 40 min. An aliquot of 100 μL of the supernatant was mixed with an appropriate volume of ultra-pure water before LC-MS/MS analysis. The apparent permeability coefficient (P_{app}), in units of centimeter per second, was calculated using the following equation: $P_{app} = (V_A \times [drug]_{reciever})/(Area \times Time \times [drug]_{initial,\ donor})$, where V_A is the volume (in mL) in the reciever well, Area is the surface area of the membrane (0.143 cm^2 for Transwell-96 Well Permeable Supports), and time is the total transport time in seconds.

Caco-2 permeability assay

Caco-2 cells were prepared in cell seeding medium consisting of DMEM with high glucose (4.5 g/L) and L-glutamine supplemented with: 10% FBS, 0.1 mg/mL of streptomycin, 100 units/mL of penicillin and 1% non-essential amino acids (100× NEAA). The cells were cultivated in a cell culture incubator set at 37 °C, 5% CO_2, 95% relative humidity to reach 70-90% confluence before detaching and splitting. Before seeding, 50 μL of culture medium was added to each well of the transwell insert and 25 mL of culture medium in

reservoir. The plate was then incubated at 37 °C, 5% CO_2 for 1 h. After the cell dissociation, the cell suspension was removed to a conical tube and the cells were pelleted by centrifugation at 120 g for 5 min. The cells were then resuspended in seeding medium at a density of 6.86 × 10^5 cells/mL. An aliquot of the cell suspension (50 μL) was added to each well of a previously prepared transwell plate to yield a final cell monolayer density of 2.4×10^5 cells/cm^2. The plate was then incubated for 14-18 days. The medium was replaced every other day, beginning no sooner than 24 hours after initial plating. The medium was then aspirated from the reservoir and each transwell insert. Culture medium (75 μL) was added to each well of the transwell insert and 25 mL of culture medium was added to the reservoir tray. The transport buffer (HBSS (Hank's Balanced Salt Solution) with 10 mM HEPES (4-(2-hydroxyethyl)-1-piperazineethanesulfonic acid), pH 7.4) was prepared by adding 2.38 g of HEPES and 0.35 g $NaHCO_3$ to 900 mL of pure water followed by sonication to dissolve the content. Next, 100 mL of HBSS (10x) was added and the resultant solution was stirred, and the pH slowly adjusted with NaOH to 6.5 and 7.4 and filtered to give HBSS solution (with 10 mM HEPES, pH 6.5 and pH 7.4). Bulk donor and receiver solutions (pH 6.5 and 7.4) were prepared by combining 497.5 mL of the respective transport buffer with 2.5 μL of compound solution to reach a total volume of 500 μL. Specifically test article stock solutions were prepared at 50, 20, 10, and 1 mM in DMSO, then diluted with transport buffer to test concentrations of 250, 100, 50, and 5 μM. 1 mM control compound (digoxin, propranolol and minoxidil) solutions in DMSO were prepared. The final test concentration of the control compounds was 5 μM. The content of organic reagent (DMSO) was 0.5%. The Caco-2 plate was removed from the incubator and the monolayer was washed twice using pre-warmed HBSS solution (10 mM HEPES, pH 7.4). The plate was then incubated at 37 °C for 30 min. The drug transport in the apical to basolateral direction was determined by adding 125 μL of each donor solution (pH 6.5) to the transwell insert (apical compartment) and an aliquot (235 μL) of receiver solutions (pH 7.4) were added into the appropriate wells of a 96-well basolateral receiver plate. The drug transport in the basolateral to apical direction was determined by adding 285 μL of each donor solution (pH 7.4) to the appropriate well of a 96-well basolateral receiver plate and an aliquot (75 μL) of receiver solutions (pH 6.5) was added to the transwell insert (apical compartment). The multiwell insert plate was inserted into the basolateral plate, transferred into the incubator, and incubated at 37 °C for 2 h. An aliquot (50 μL) of both apical and basolateral wells (T=120 min) was transferred into two new 96-well plates and 200 μL quench solution (acetonitrile with 100 nM alprazolam, 200 nM labetalol, 200 nM caffeine and 2 μM ketoprofen) was then added. The samples were vortexed for 10 min and then centrifuged at 3220 g, 4 °C for 30 min. 100 μL of the supernatant was transferred to a new 96-well plate, diluted with 100 μL of pure water, and analyzed by UPLC-MS/MS. All incubations were performed in duplicate. Apparent permeability (P_{app}, × 10^{-6} cm/s) was calculated for drug transport assays using the following equation: $P_{app} = (V_A \times [drug]_{reciever})/(Area \times Time \times [drug]_{initial, donor})$, where V_A is the volume (in mL) in the receiver well (0.235 mL for A to B flux and 0.075 mL for B to A flux), Area is the surface area of the membrane (0.143 cm^2 for HTS Transwell-96 Well Permeable Supports), and Time is the total transport time in seconds (7200s). Efflux ratio was determined using the following equation: Efflux Ratio = $P_{app (B-A)}/P_{app (A-B)}$, where $P_{app (B-A)}$ is the apparent permeability coefficient in basolateral to apical direction and $P_{app (A-B)}$ is the apparent permeability coefficient in apical to basolateral direction.

HT solubility assay

FeSSIF was prepared according to the following procedure: 1) 4.04 g of NaOH, 8.65 g of glacial acetic acid and 11.87 g of NaCl were dissolved in 900 mL ultrapure water and the pH of the solution was adjusted to 5.0 with 1 N NaOH or 1 N HCl, 2) the buffer solution was diluted with ultrapure water to 1000 mL at room temperature, 3) 11.2 g of FeSSIF powder (FFF-1219-A from Biorelevant) was added to 500 mL of the buffer solution and the resultant mixture was stirred until the powder was completely dissolved, and 4) the solution was further diluted with buffer solution to a final volume 1000 mL. SGF was prepared according to the following procedure: 1) 234 mL concentrated HCl was added to a 1000 mL volumetric flask and diluted with ultrapure water to reach a final volume of 1000 mL and 2) 16.4 mL of the dilute HCl solution, 800 mL of ultrapure water, and 10.0 g of pepsin were added a 1000 mL volumetric flask, mixed, and diluted with ultrapure water to reach a final volume of 1000 mL. 100 mM sodium phosphate (PBS) solution was prepared according to the following procedure: 1) a basic solution of 14.2 g/L Na_2HPO_4 in deionized water was prepared, 2) an acidic solution of 12.0 g/L NaH_2PO_4 in deionized water was prepared, and 3) the basic solution was titrated with the acidic solution to reach a final pH of 7.4. Compounds were prepared as 10 mM stock solutions in DMSO and added (30 μL/vial) to vials contained within a 96-well cap-less solubility sample plate, followed by 970 μL of PBS (pH 7.4), FeSSIF (fed state simulated intestinal fluid), or SGF (simulated gastrointestinal fluid). The vials were then sealed using a molded PTFE/silicone plug. The assay was performed in duplicate. Then the solubility sample plate was transferred to an Eppendorf Thermomixer Comfort plate shaker and shaken at 25 °C

at 1100 RPM for 2 h. Next the plugs were removed and the stir sticks were removed using a magnet, the samples from the solubility sample plate were transferred into a filter plate. Samples were filtered and a 5 μL aliquot was transferred to a 96-well plate and diluted with 5 μL DMSO and 490 μL (1:1) acetonitrile/water. The plates were then vortexed for 5 min and centrifuged at 3,220 g at 4 °C for 10 min. The samples were evaluated by LC-MS/MS analysis. The filtrate was analyzed and quantified against a standard of known concentration using LC, coupled with mass spectral peak identification and quantitation. Solubility values of the test compound and control compound were calculated as follows: $[Sample] = AREA_{Sample} \times Injection\ Volume_{Standard} \times Dilution\ Factor_{Sample} \times [Standard]\ /\ AREA_{Standard} \times Injection\ Volume_{Sample}$.

Plasma protein binding assay

Compound and control (ketoconazole) solutions were prepared in DMSO at 1 mM. Frozen plasma (stored at −80 °C) was thawed immediately in a room temperature water bath (37 °C), centrifuged at 3,220 g for 10 min to remove clots, and the supernatant was collected into a fresh tube. The pH of the plasma was checked, and only plasma within the range of pH 7 to pH 9 was used. Plasma was then pre-incubated in a 37 °C water bath for 5 minutes. Plasma protein binding of test compound was determined by diluting 3 μL of 1 mM compound solution with 2997 μL of the pre-warmed plasma to obtain the final concentration of 1 μM. Tubes were then gently votexed. A 1 mL plasma sample was transferred to two ultracentrifuge tubes (n=2). The ultracentrifuge tubes were balanced into pre-warmed rotor, and incubated at 37 °C, 5% CO_2 for 30 min. After incubation, the ultracentrifuge tubes were centrifuged at 600,000 g for 5.5 h at 37 °C. 50 μL of spiked plasma sample was transferred to six 0.6 mL tubes. Samples were incubated at 37 °C, 5% CO_2 for 0, 0.5, and 6 h. At designated time points, 50 μL of PBS was added, mixed thoroughly and then 400 μL of RT quench solution (acetonitrile containing internal standards (IS, 500 nM Labetalol, 100 nM Alprazolam and 2 μM Ketoprofen)) was added to precipitate protein. The samples were vortexed for 5 min and centrifuged for 15 min at 20,000 g. The 0.5 hour samples were non-spun controls. After ultracentrifugation, 50 μL from the center of the ultracentrifuge tubes was removed as the post-ultracentrifugation sample and 50 μL of plasma was added to the samples. The samples were then treated the same as non-spun control samples. 100 μL of the supernatant was transfered to a new plate. The supernatant was diluted with 100 μL or 200 μL water according to the LC-MS signal response and peak shape, mixed well, and analyzed by LC-MS/MS. Unbound fraction and bound fraction are calculated using the following equations: Unbound fraction = $\text{Conc.}_{\text{Post Ultracentrifugation}}$ / $\text{Conc.}_{\text{Non-Spun Control}}$ and Bound fraction = 1–Unbound, where $\text{Conc.}_{\text{post-ultracentrifugation}}$ is the concentration in post-ultracentrifugation samples and $\text{Conc.}_{\text{non-spun control}}$ is the concentration in non-spun control sample.

Whole blood portioning ratio

Working solutions of test compounds were prepared in DMSO at the concentration of 1 mM and the control compound chloroquine was prepared in ultrapure water at 1 mM. A volume of plasma sufficient for the assay was obtained from a portion of the whole blood by centrifugation (10,000 g, 4°C and 10 minutes). 2 μL of working solutions were added to 398 μL of reference blood and blood to achieve a final concentration of 5 μM. The assay was performed in duplicate. The final percent volume of organic solvents was no more than 0.5%. Tubes were incubated at 37 °C with shaking for 60 min. Next, 50 μL of the reference blood samples was removed into new tubes. These samples (the 50 uL samples transferred from reference blood samples) were then spun for 10 minutes at 10,000 g (37 °C) together with the blood samples. Aliquots (50 μL) of the spiked reference blood samples (before incubation) were removed into new tubes containing 50 μL of blank plasma and mixed well. 400 μL of RT quench solution (acetonitrile containing internal standards: 500 nM Labetalol, 100 nM Alprazolam and 2 μM Ketoprofen) was added to precipitate protein then vortexed for 5 minutes. The samples were then stored in a freezer before centrifugation. 50 μL of plasma from centrifuged whole blood samples was removed into new tubes. 50 μL of blank plasma was added to the 50 μL of centrifuged reference blood samples, and an equal volume of blank whole blood was added to the 50 μL of collected plasma samples. The samples were mixed well and then 400 μL of RT quench solution (acetonitrile containing internal standard: 500 nM Labetalol, 100 nM Alprazolam and 2 μM Ketoprofen) was added to precipitate protein. The samples were then vortexed for 5 min and centrifuged for 15 minutes at 20,000 g at 4 °C. 100 μL of the supernatant was transferred to a new plate. The supernatant was diluted with 100 μL water. The samples were mixed well and analyzed using LC-MS/MS. The concentrations of test compound and control compound in the plasma/blood were determined from peak area ratios. Blood to plasma ratios ($K_{b/p}$) of test compound and control compound were calculated by the following equation $K_{b/p} = I_{REF\ BL}\ /\ I_{PL}$, where % remaining = $I_{REF\ BL}\ /\ I_{T0\ *\ 100}$, $I_{REF\ BL}$ is peak area ratio of analyte in the reference blood sample, and I_{PL} is the peak area ratio of analyte in the plasma sample separated from whole blood.

Whole blood stability assay

Compounds were prepared 1 mM in DMSO and added (4 µL/well) to 796 µL of pre-incubated whole blood to reach a final concentration of 5 µM and resulting in a final solvent concentration of 0.5%. Time 0 samples were prepared by adding 50 µL of the spiked whole blood to new microfuge tubes of a 96-well plate containing 300 µL of RT quench solution (acetonitrile containing internal standards (100 nM alprazolam and 500 nM labetalol). Aliquots of 50 µL of the spiked whole blood samples were transferred into new microfuge tubes and incubated at 37 °C with shaking at 60 rpm for 15, 30, 60, and 120 min. The assay was performed in duplicate. The reaction was stopped by adding 300 µL of RT quench solution to the spiked whole blood samples at the aforementioned time points. Propantheline bromide was used as the positive control for beagle dog, cynomolgus monkey, and human whole blood, and mevinolin was used as the positive control for Sprague-Dawley rat whole blood. The samples were vortex mixed for 5 minutes. Samples in the plate were centrifuged at 3,220 × g for 30 min at 4 °C to precipitate proteins, and 100 µL of the supernatant was transferred to a new 96-well plate with 100 µL ultrapure water for UPLC MS/MS analysis. The slope value was determined by linear regression of the natural logarithm of the remaining percentage of the parent drug vs. incubation time curve. The *in vitro* half-life ($t_{1/2}$) was determined from the slope value ($t_{1/2}$ = 0.693/k, where k = -slope value). Maximal depletion of the parent molecule able to be accurately measured was determined to be 15%. Consequently, the maximal $t_{1/2}$ for the 120 min incubation was assumed to be 512 min.

GSH stability assay

Solutions of compounds and control compounds (*N*-(4-nitrophenyl)acrylamide and *N*-phenylacrylamide) were prepared in DMSO at 500 µM. 2 µL of the 500 µM solutions and 198 µL of PBS pH 7.4 with 5 mM GSH were added into glass vials to achieve a final compound concentration of 5 µM. The assay was performed in duplicate. Vials were incubated at 37°C at 60 rpm in a water bath and taken at designated time points including 0, 15, 30, 60, 120 and 180 minutes. For each time point, the initiation of the reaction was staggered so all time points were terminated with 1000 µL cold acetonitrile containing internal standards (IS, 100 nM Alprazolam, 500 nM Labetalol and 2 µM Ketoprofen) at the same time. Samples were vortexed for 1 minute then centrifuged at RT at 2,500 g for 10 minutes. Aliquots of 200 µL of the supernatant were used for LC-MS/MS analysis. The slope value was determined by linear regression of the natural logarithm of the remaining percentage of the parent drug vs. incubation time curve. The *in vitro* half-life ($t_{1/2}$) was determined from the slope value ($t_{1/2}$ = 0.693/k, where k = -slope value). Maximal depletion of the parent molecule able to be accurately measured was determined to be 15%. Consequently, the maximal $t_{1/2}$ for the 180 min incubation was assumed to be 768 min.

Hepatocyte stability assay

Compounds were prepared 100 µM in (1:1) acetonitrile/water and added (2 µL/well) to thawed cryopreserved rat, dog, monkey, and human hepatocytes (0.5 × 10^6 cells/mL; 198 µL/well) in 96-well non-coated plates. Incubations were carried out at a final test concentration of 1 µM over a total incubation period of 120 minutes (37 °C, 5% CO_2). 25 µL aliquots were taken at time points of 0, 15, 30, 60, 90 and 120 minutes. The aliquots were mixed with 6 volumes (150 µL) of acetonitrile containing internal standards (IS: 100 nM alprazolam, 200 nM labetalol, 200 nM caffeine and 2 µM ketoprofen) to terminate the reaction. The plates were centrifuged for 20 minutes at 3,220 g. 100 µL aliquots of the supernatant were mixed with 100 µL of ultra-pure H_2O for LC-MS/MS analysis to determine the concentration of test compound and the percentage remaining to calculate the intrinsic clearance (*in vitro* CL_{int}) and half-life values using the substrate depletion method. All incubations were performed in triplicate.

Animal studies

All animal handling and treatment procedures were performed according to the approved Institutional Animal Care and Use Committee guidelines following the Association for Assessment and Accreditation of Laboratory Animal Care guidance. All studies utilized 6-8 week old athymic nude BALB/C mice (Jackson Labs, Beijing HFK Bio-Science Co, Ltd, or GemPharmatech Co, Ltd). All animals were kept in an environmentally monitored, well-ventilated room at a relative humidity of 40–70%, at 3-5 mice/cage and a 12-hour light/dark cycle.

Ba/F3 allograft and NCI-H2073 xenograft studies

Mouse Ba/F3 EGFR V769_D770 insASV cells (2×10^6 cells in 0.1 mL of RPMI-1640 Medium), human NCI-H2073 NSCLC cells, or derivatized NCI-H2073 cells bearing a homozygous knockin of the p.D770_N771insSVD insertion into the endogenous *EGFR* locus[6] (1×10^7 cells in 0.1 mL of PBS) were injected subcutaneously into the right flanks of female athymic nude mice. Treatments started when mean tumor volume reached a minimum of approximately 150-200 mm^3. Compound **32** and **53** (STX-721) were formulated in 1% Tween 80, 2% HPMC, and H_2O, mobocertinib was formulated in 0.1% Tween 80, 0.5% HPMC, and H_2O. Tumor volumes were calculated using TV = a × b2*.52, where "a" and "b" are long and short diameters of a tumor, respectively. Body weight change was calculated as BW change (%) = BW (Day X) / BW (Day 0) × 100. The tumor volume and percent body weight change were reported as mean +/- SEM. At the end of each study, statistical significance was assessed using two-way RM ANOVA followed by Tukeys post hoc comparisons of the meansusing GraphPad Prism 9.4.1. Percent Tumor Growth Inhibition (TGI) was calculated from measured tumor volumes (TV) as follows: %TGI = [1 – (Treated final TV –Treated initial TV)/(Vehicle final TV – Vehicle initial TV)] × 100. Percent regression was calculated by the formula: % Regression = [1 – (Treated final TV – Treated initial TV)] × 100. Fold change in tumor volume as calculated as (TV final – TV initial) /TV initial × 100. Data represents the fold change of each tumor +/- SEM.

Pharmacokinetic and tumor pharmacodynamic assays

For single dose PK/PD studies of Compound **53** (STX-721) and **32** in Ba/F3 EGFR V769_D770 insASV allografts, subcutaneous injection was performed as noted above, with the exception that treatment began at a tumor volume of roughly 500-1000 mm^3. After dosing, at the noted timepoints blood was collected to determine exposure levels and tumor was collected to determine pharmacodynamic modulation of pEGFR (Y1068) and pERK (ERK1: T202/Y204; ERK2: T185/Y187). Tumor samples were snap frozen until processing in Phosphosafe Extraction buffer (Novagen) and homogenization via TissueLyser (Qiagen) for 2 min × 30 amplitude. Samples were then centrifuged at 12,000 g at 4 °C for 10 min and supernatants were collected. Total protein concentration was determined by BCA according to manufacturer's instructions (Thermo), and pEGFR and pERK levels were determined using pEGFR and pERK AlphaLISA kits according to manufacter's instructions (Perkin Elmer), reading on Pherastar FSX (BMG Labtech) using the AlphaLISA module.

Blood pharmacokinetic analysis for test articles was performed as follows. The desired serial concentrations of working solutions were achieved by diluting stock solution of analyte with 50% acetonitrile in water solution. 5 μL of working solutions (2, 4, 20, 100, 200, 1000, 2000, 4000 ng/mL) were added to 10 μL of the blank Balb/c nude Mice plasma to achieve calibration standards of 1~2000 ng/mL (1, 2, 10, 50, 100, 500, 1000, 2000 ng/mL) in a total volume of 15 μL. Five quality control samples at 2 ng/mL, 5 ng/mL, 50 ng/mL, 800 ng/mLand 1600 ng/mL for plasma were prepared independently of those used for the calibration curves. These QC samples were prepared on the day of analysis in the same way as calibration standards. 15 μL standards, 15 μL QC samples and 15 μL unknown samples (10 μL plasma with 5 μL blank solution) were added to 200 μL of acetonitrile containing IS mixture for precipitating protein respectively. Then the samples were vortexed for 30 s. After centrifugation at 4 °C, 3900 rpm for 15 min, the supernatant was diluted 3 times with water. 5 μL of diluted supernatant was injected into the LC/MS/MS system for quantitative analysis, and resulting values were corrected for plasma protein binding.

Protein crystallization

NPG protein in complex with the compound was crystallized by sitting-drop vapor-diffusion at 20°C. NPG protein (7.3 mg/mL in 300 mM NaCl, 25 mM HEPES-NaOH pH 8.0, 4 mM TCEP, 10% v/v Glycerol) was incubated with 1 mM compound for 1 hour at 4°C. For Compound **39** 0.10 μL of the sample was mixed with 0.18 μL of crystallization solution (0.2 M Ammonium Sulfate, 100 mM HEPES-NaOH pH 6.8, 20% v/v PEG 5000 MME) and 0.02 μL seed stock and equilibrated against a reservoir containing 0.07 mL crystallization solution. The crystals were mounted after two weeks. Crystals were cryo-protected in crystallization solution containing 25% v/v Ethylene Glycol and cooled in liquid nitrogen. Data were collected at beamline P14 of the Deutsches Elektronen-Synchrotron (Hamburg, Germany). For Compound **23** 0.10 μL of the sample was mixed with 0.03 μL of crystallization solution (0.3 M Sodium Chloride, 50 mM TRIS-HCl pH 7.9, 9% v/v PEG 400) and 0.02 μL seed stock and equilibrated against a reservoir containing 0.07 mL crystallization solution. The crystals were mounted after three weeks. Crystals were cryo-protected in Paratone-

n and cooled in liquid nitrogen. Data were collected at beamline ID30A1 of the European Synchrotron Radiation Facility (Grenoble, France).

Crystals of EGFR kinase wildtype in complex with Compound **23** were grown by mixing protein solution (4 mg/ mL + 2 mM compound) with reservoir solution containing sodium tartrate, sodium acetate and ammonium chloride in a 1:1 ratio using the sitting drop vapor diffusion method at 293 K. Before flash freezing in liquid nitrogen, crystals were cryo-protected by immersing them in reservoir solution supplemented with 25% (v/v) glycerol. Diffraction data of the complex were collected at the Swiss Light Source (SLS, Villigen, Switzerland).

Structure determination and refinement

NPG co-structure diffraction data were integrated, analyzed and scaled with *XDS*, *POINTLESS*, *AIMLESS* and *STARANISO* in *AUTOPROC*. The structures were determined by molecular replacement with PHASER using a previously determined model (without any ligands) as a starting model. The model was improved through manual rebuilding of the model in COOT and restrained refinement with BUSTER. Atomic displacement factors were modelled with a single TLS group per chain and a single isotropic B-factor per atom. The backbone geometry was analyzed with MOLPROBITY. The restraints for the modelled compounds were generated with JIGAND (Compound **39)** or GRADE (Compound **23)**. The WT Compound **23** co-structure was solved by molecular replacement using a previously solved structure of EGFR kinase domain as a search model. Subsequent model building and refinement was performed according to standard protocols with CCP4 and COOT. Ligand parametrization and generation was carried out with CORINA. The water model was built with the "find waters2" algorithm of COOT, followed by refinement with REFMAC and checking all waters with the validation tool of COOT. The NPG complex with Compound **23**, NPG with Compound **39**, and WT with Compound **23** were deposited in the RCSB with codes 9FQP, 9FQS, and 9FRD, respectively.

Crystallography references

XDS – Kabsch W (2010) - Acta Crystallographica **D66** p125-132. *XDS.*
AIMLESS and POINTLESS - Evans P (2006) Acta Crystallographica **D62** p72-72. *Scaling and assessment of data quality.*
AUTOPROC - Vonrhein C, et al (2011) - *Data processing and analysis with the autoPROC toolbox.* Acta Crystallographica **D67** p293
BUSTER – Bricogne G, Blanc E, Brandl M, Flensburg C, Keller P, Paciorek W, Roversi P, Sharff A, Smart OS, Vonrhein C, Womack TO (2017) - BUSTER. Cambridge, United Kingdom, Global Phasing Ltd.
COOT – Emsley P, Lohkamp B, Scott WG, Cowtan K (2010) – Acta Crystallographica **D66** p486-501. *Features and development of Coot.*
JLIGAND – Lebedev A, Young P, Isupov MN, Moroz OV, Vagin AA, Murshudov GN (2012) – Acta Crystallographica **D68** p431. *JLigand: a graphical tool for the CCP4 template-restraint library.*
MOLPROBITY - Williams CJ, et al (2018) - Protein Science **27** p29. *MolProbity: More and better reference data for improved all-atom structure validation.*
GRADE - Smart OS, Womack TO, Sharff A, Flensburg C, Keller P, Paciorek W, Vonrhein C and Bricogne G (2011) - Grade. Cambridge, United Kingdom, Global Phasing Ltd.

CCP4 - CCP4 suite: J. Agirre et al. Acta. Cryst. D79, 449-461 (2023) "The CCP4 suite: integrative software for macromolecular crystallography" [doi:10.1107/S2059798323003595].

CORINA - Molecular Networks GmbH, Germany, and Altamira, LLC, USA. Corina Classic

REFMAC - Murshudov, G. N.; Skubak, P.; Lebedev, A. A.; Pannu, N. S.; Steiner, R. A.; Nicholls, R. A.; Winn, M. D.; Long, F.; Vagin, A. A. REFMAC5 for the Refinement of Macromolecular Crystal Structures. Acta Crystallogr., Sect. D: Biol. Crystallogr. 2011, 67, 355–367.

Procedures for the syntheses of STX-721 and related analogs

General synthetic procedures

All materials were obtained from commercial suppliers and used without further purification unless otherwise noted. Anhydrous solvents were obtained from Sigma-Aldrich or WuXi-EHS and used directly. Reactions involving air- or moisture sensitive reagents were performed under a nitrogen or argon atmosphere. Silica gel chromatography was performed using prepacked silica gel cartridges (SANPONT® or Agela-CS). NMR spectra were acquired on Bruker Avance or QUANTUM-1 PLUS 400 MHz spectrometer equipped with 5 mm BBFO probes. Chemical shifts are reported in parts per million (ppm, δ units). All final compounds were purified to >95% purity as determined by liquid chromatography–mass spectrometry (LC–MS) using several instruments and methods: A) Shimadzu LCMS-2020 system using an ESI model mass spectrometer utilizing ESI ionization fitted with an Advanced Materials Technology HALO C18 column (30 mm × 3.0 mm, 2.0 μm) at 40 °C with a 1.5 mL/min flow rate using a 5–100% gradient of acetonitrile/water with 0.1% formic acid over 1.5 min; B) Shimadzu LCMS-2020 system using a SCAN model mass spectrometer utilizing ES-API ionization fitted with an Advanced Materials Technology Halo 90A C18 column (30 mm × 3.0 mm, 5.0 μm) at 50 °C with a 1.5 mL/min flow rate using a 5–95% gradient of acetonitrile/water with 0.04% trifluoracetic acid over 1.0 min; C) Agilent 1260-6025 Halo 90A C18 column (30 mm × 3.0 mm, 5.0 μm) at 50 °C with a 1.5 mL/min flow rate using a 5–95% gradient of acetonitrile/water with 0.04% trifluoracetic acid over 1.0 min; D) Shimadzu LCMS-2020 Kinetex EVO C18 column (30 mm × 2.1 mm, 5.0 μm) at 50 °C with a 1.5 mL/min flow rate using a 5–95% gradient of acetonitrile/water with 0.025% $NH_3{\cdot}H_2O$ over 1.5 min.

Compound 1

3-[(3-Chloro-2-methoxyphenyl)amino]-2-[3-[(2*S*)-oxolan-2-ylmethoxy]pyridin-4-yl]-1*H*,5*H*,6*H*,7*H*-pyrrolo[3,2-c]pyridin-4-one.[7]

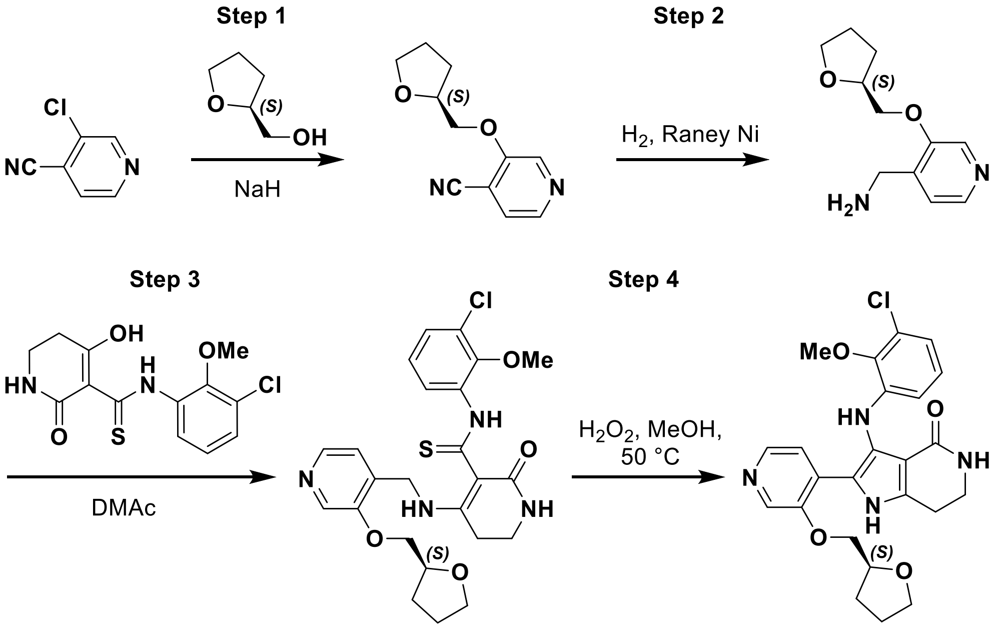

Compound 1

Step 1: 3-[(2*S*)-Oxolan-2-ylmethoxy]pyridine-4-carbonitrile. To a solution of (2*S*)-oxolan-2-ylmethanol (50 mg, 0.49 mmol) in DMF (0.5 mL) was added sodium hydride (60% in oil, 17.6 mg) at 0 °C. After stirring for 15 min, 3-chloropyridine-4-carbonitrile (81.4 mg, 0.588 mmol, 1.2 equiv) was added. The mixture was allowed to warm to ambient temperature and stirred for an additional

4 h. The resulting mixture was extracted with EtOAc (3 × 20 mL). The combined organic layers were washed with brine (3 × 30 mL), dried over anhydrous sodium sulfate, filtered, and concentrated under reduced pressure. Reverse-phase preparative chromatography (C18 silica gel; 10–50% water/MeOH, gradient over 10 min) gave 3-[(2*S*)-oxolan-2-ylmethoxy]pyridine-4-carbonitrile (51.0 mg, 69% yield) as a white oil. *m/z* (ESI, +ve ion): 205.09 $(M + H)^+$.

Step 2: (*S*)-(3-((Tetrahydrofuran-2-yl)methoxy)pyridin-4-yl)methanamine. 3-[(2*S*)-oxolan-2-ylmethoxy]pyridine-4-carbonitrile (1.81 g, 8.70 mmol) was added to a mixture of Raney Nickel (2.50 g) in MeOH (20.0 mL) and stirred overnight at rt under a H_2 atmosphere. The resultant black mixture was filtered and the filter cake was washed with MeOH (200mL). The filtrate was concentrated under reduced pressure to afford (*S*)-(3-((tetrahydrofuran-2-yl)methoxy)pyridin-4-yl)methanamine (1.10 g, 59% yield) as a light-green oil, which was used directly in next step without further purification. *m/z* (ESI, +ve ion): 208.10 $(M + H)^+$.

Step 3: *N*-(3-Chloro-2-methoxyphenyl)-2-oxo-4-[([3-[(2*S*)-oxolan-2-ylmethoxy]pyridin-4-yl]methyl)amino]-5,6-dihydro-1*H*-pyridine-3-carbothioamide. 1-[3-[(2*S*)-oxolan-2-ylmethoxy]pyridin-4-yl]methanamine (200 mg, 0.960 mmol) and *N*-(3-chloro-2-methoxyphenyl)-4-hydroxy-2-oxo-5,6-dihydro-1*H*-pyridine-3-carbothioamide (240 mg, 0.768 mmol, see main text for synthesis procedure) were added to *N*,*N*'-dimethyl acetamide (5 mL) and the resultant mixture stirred at 110 °C under a N_2 atmosphere for 2 h. The resulting mixture was extracted with EtOAc (3 × 100 mL). The combined organic layers were washed with brine (3 × 50 mL), dried over anhydrous sodium sulfate, filtered, and concentrated under reduced pressure. Prep-TLC purifcation (silica gel; 15/1 DCM/MeOH) afforded *N*-(3-chloro-2-methoxyphenyl)-2-oxo-4-[([3-[(2*S*)-oxolan-2-ylmethoxy]pyridin-4-yl]methyl)amino]-5,6-dihydro-1H-pyridine-3-carbothioamide (199 mg, 42% yield) as a light-yellow solid. *m/z* (ESI, +ve ion): 503.60 $(M + H)^+$.

Step 4: 3-[(3-Chloro-2-methoxyphenyl)amino]-2-[3-[(2*S*)-oxolan-2-ylmethoxy]pyridin-4-yl]-1*H*,5*H*,6*H*,7*H*-pyrrolo[3,2-c]pyridin-4-one. H_2O_2 (40.0 µL, 0.35 mmol, 30% w/v in H_2O) was added to a solution of *N*-(3-chloro-2-methoxyphenyl)-2-oxo-4-[([3-[(2*S*)-oxolan-2-ylmethoxy]pyridin-4-yl]methyl)amino]-5,6-dihydro-1H-pyridine-3-carbothioamide (100 mg, 0.178 mmol) in MeOH (2.50 mL) and the resultant mixture was stirred overnight at 50 °C under a N_2 atmosphere. The reaction mixture was allowed to cool to RT and concentrated under reduced pressure. The residue was purified by Prep-HPLC (Column: CHIRAL ART Cellulose-SC, 2 × 25 cm, 5 µm; Mobile Phase A: 3/1 Hex/DCM (0.5% 2 M NH_3-MeOH), Mobile Phase B: EtOH; Flow rate: 20 mL/min; Gradient: 30% B to 30% B in 10 min; Rt: 6.96 min) to afford 3-[(3-chloro-2-methoxyphenyl)amino]-2-[3-[(2*S*)-oxolan-2-ylmethoxy]pyridin-4-yl]-1*H*,5*H*,6*H*,7*H*-pyrrolo[3,2-c]pyridin-4-one (8.90 mg, 9.2% yield) as a light yellow solid. ^{1}H NMR (400 MHz, DMSO-d_6) δ = 11.41 (s, 1H), 10.76 (d, *J* = 5.9 Hz, 1H), 8.49 (s, 1H), 8.13 (d, *J* = 5.0 Hz, 1H), 7.45 (d, *J* = 5.0 Hz, 1H), 7.38 (s, 1H), 7.05 (dd, *J* = 7.1, 5.8 Hz, 1H), 6.76–6.59 (m, 2H), 6.37 (d, *J* = 7.0 Hz, 1H), 6.17 (dd, *J* = 7.5, 2.2 Hz, 1H), 4.32 (ddt, *J* = 13.7, 6.9, 3.5 Hz, 2H), 4.10 (dd, *J* = 9.8, 6.8 Hz, 1H), 3.98–3.61 (m, 5H), 2.01 (dq, *J* = 12.5, 7.2 Hz, 1H), 1.92– .78 (m, 2H), 1.77–1.61 (m, 1H). *m/z* (ESI, +ve ion): 469.16 $(M + H)^+$.

Compound S1

3-[(3-Chloro-2-methoxyphenyl)amino]-2-[3-[(2*R*)-oxolan-2-ylmethoxy]pyridin-4-yl]-1*H*,5*H*,6*H*,7*H*-pyrrolo[3,2-c]pyridin-4-one[7] was prepared according to the procedure described for Compound **1**, using (2*S*)-oxolan-2-ylmethanol in Step 1 (59% yield). Next, Step 2 (67% yield), Step 3 (50% yield), and Step 4 afforded the title compound (34.3 mg, 23% yield) as a light yellow solid. ^{1}H NMR (400 MHz, DMSO-d_6) δ = 11.25 (s, 1H), 8.43 (s, 1H), 8.04 (d, *J* = 5.1 Hz, 1H), 7.48 (s, 1H), 7.28 (d, *J* =5.1 Hz, 1H), 7.13 (d, *J* = 2.4 Hz, 1H), 6.73–6.64 (m, 2H), 6.22–6.13 (m, 1H), 4.41–4.27 (m, 2H), 4.04 (dd, *J* = 9.8, 7.4 Hz, 1H), 3.92–3.82 (m, 4H), 3.78 (dt, *J* = 8.0, 6.6 Hz, 1H), 3.43 (td, *J* = 6.9, 2.5 Hz, 2H), 2.89–2.81 (m, 2H), 2.09–1.97 (m, 1H), 1.95–1.83 (m, 2H), 1.75–1.62 (m, 1H). *m/z* (ESI, +ve ion): 469.10 $(M + H)^+$.

Compound 2

3-[(3-Chloro-2-methoxyphenyl)amino]-2-(pyridin-4-yl)-1*H*,5*H*,6*H*,7*H*-pyrrolo[3,2-c]pyridin-4-one was prepared was prepared according to the procedure described for Compound **1**, using 4-pyridinemethaneamine in Step 3 heating at 120 °C for 2 h (35% yield). Cyclization Step 4 proceeded at 80 °C in 2 h to yield the title compound (20.4 mg, 22% yield) after purification as a yellow solid. ^{1}H NMR (300 MHz, DMSO-d_6) δ = 11.84 (s, 1H), δ 8.45–8.37 (m, 2H), 7.40 (dd, *J* = 6.7, 2.1 Hz, 3H), 7.12 (s, 1H), 6.79–6.67 (m, 2H), 6.14 (dd, *J* = 5.5, 4.1 Hz, 1H), 3.91 (s, 3H), 3.41 (td, *J* = 6.8, 2.6 Hz, 2H), 2.86 (t, *J* = 6.8 Hz, 2H). *m/z* (ESI, +ve ion): 369.0 $(M + H)^+$.

Compound 3

3-[(3-Chloro-2-methoxyphenyl)amino]-2-(3-methoxypyridin-4-yl)-1*H*,5*H*,6*H*,7*H*-pyrrolo[3,2-c]pyridin-4-one prepared was prepared according to the procedure described for Compound **1**, using 1-(3-methoxypyridin-4-yl)methanamine in Step 3 heating at 80 °C for 3 h (62% yield). Cyclization Step 4 proceeded in 2 h at RT to yield the title compound (14.4 mg, 17% yield) after purification as a yellow solid. ^{1}H NMR (300 MHz, DMSO-d_6) δ = 11.26 (s, 1H), 8.38 (s, 1H), 8.04 (d, *J* = 5.0 Hz, 1H), 7.50 (s, 1H), 7.31 (d, *J* = 5.0 Hz, 1H), 7.10 (s, 1H), 6.71–6.63 (m, 2H), 6.18–6.11 (m, 1H), 3.96 (s, 3H), 3.89 (s, 3H), 3.47–3.38 (m, 2H), 2.88 (t, *J* = 6.8 Hz, 2H). *m/z* (ESI, +ve ion): 399.10 $(M + H)^+$.

Compound 4

3-[(3-Chloro-2-methoxyphenyl)amino]-2-(3-fluoropyridin-4-yl)-1*H*,5*H*,6*H*,7*H*-pyrrolo[3,2-c]pyridin-4-one.

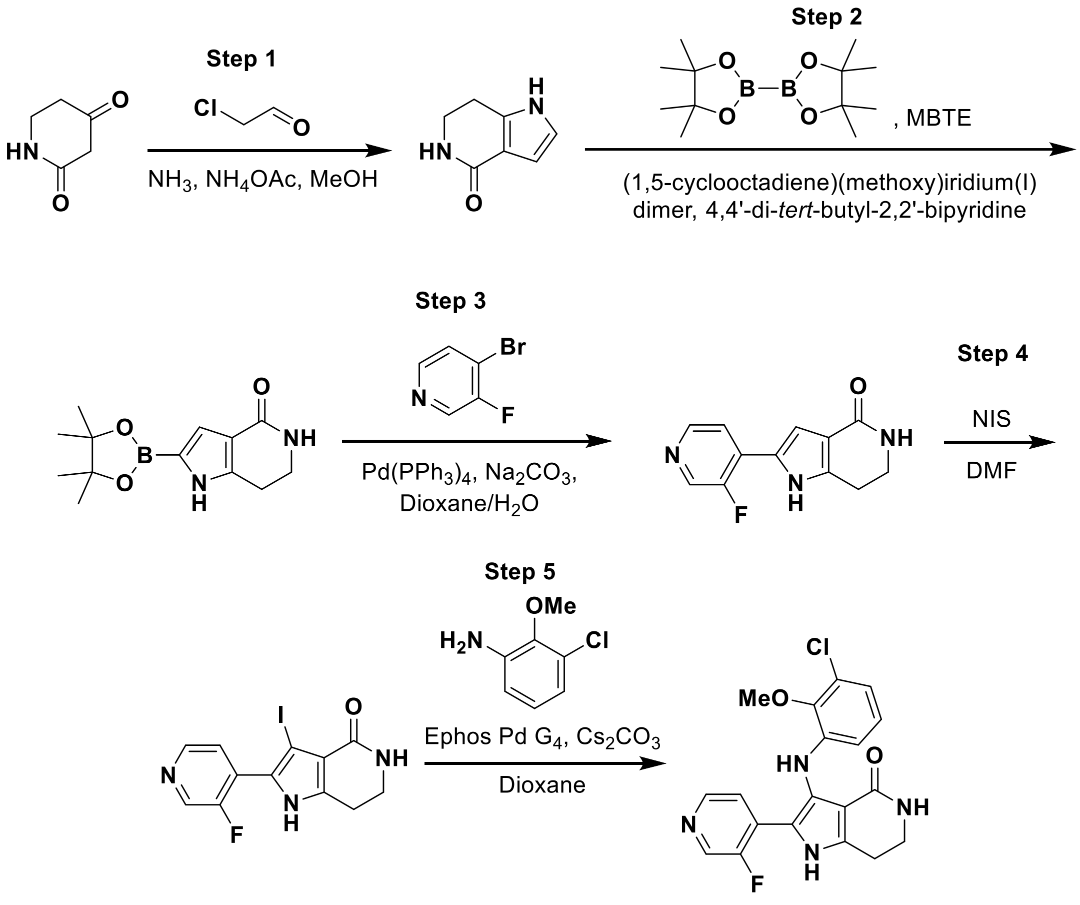

Compound 4

Step 1: 1*H*,5*H*,6*H*,7*H*-Pyrrolo[3,2-c]pyridin-4-one. To a stirred solution of piperidine-2,4-dione (10.0 g, 88.4 mmol) in NH_3 in MeOH (67 mL, 2 M solution) were added NH_4OAc (27.3 g, 354 mmol) and chloroacetaldehyde (13.9 g, 177 mmol) in portions at RT under a N_2 atmosphere. The resulting mixture was stirred for 2 h at 50 °C. The resulting oil was dried under reduced pressure and the residue purified by silica gel column chromatography, eluted with DCM / MeOH (10:1) to afford 1*H*,5*H*,6*H*,7*H*-pyrrolo[3,2-c]pyridin-4-one (6.0 g, 50% yield) as a yellow solid. *m/z* (ESI, +ve ion): 137.3 $(M + H)^+$.

Step 2: 2-(4,4,5,5-Tetramethyl-1,3,2-dioxaborolan-2-yl)-1*H*,5*H*,6*H*,7*H*-pyrrolo[3,2-c]pyridin-4-one. To a stirred solution of 1*H*,5*H*,6*H*,7*H*-pyrrolo[3,2-c]pyridin-4-one (100 mg, 0.734 mmol) and bis(pinacolato)diboron (224 mg, 0.881 mmol) in MTBE

(7.5 mL) was added (1,5-cyclooctadiene)(methoxy)iridium(I) dimer (29.2 mg, 0.044 mmol) and 4,4'-di-*tert*-butyl-2,2'-bipyridine (19.7 mg, 0.073 mmol) in portions at RT under a argon atmosphere. The resulting mixture was stirred for 1 h at 50 °C and the solid precipitate was collected by filtration, washed with MTBE (3 × 20 mL), and dried under reduced pressure to afford 2-(4,4,5,5-tetramethyl-1,3,2-dioxaborolan-2-yl)-1*H*,5*H*,6*H*,7*H*-pyrrolo[3,2-c]pyridin-4-one (80 mg, 42% yield) as a light yellow solid that was used directly without further purification. *m/z* (ESI, +ve ion): 262.95 $(M + H)^+$.

Step 3: 2-(3-Fluoropyridin-4-yl)-1*H*,5*H*,6*H*,7*H* -pyrrolo[3,2-c] pyridin-4-one. A mixture of 4-bromo-3-fluoropyridine (280 mg, 1.59 mmol), 2-(4,4,5,5-tetramethyl-1,3,2-dioxaborolan-2-yl)-1*H*,5*H*,6*H*,7*H*-pyrrolo[3,2-c] pyridin-4-one (625 mg, 2.38 mmol) and Na_2CO_3 (505 mg, 4.77 mmol, 3.00 equiv) in 1,4-dioxane (15 mL) and H_2O (3.0 mL) was prepared at RT under a N_2 atmosphere. To the above mixture was added $Pd(PPh_3)_4$ (367 mg, 0.31 mmol) and the resulting mixture was warmed to 50 °C and stirred for 12 h. The reaction mixture was cooled to RT, diluted with H_2O (25 mL) and extracted with DCM (3 × 60 mL). The combined organic layers were concentrated under reduced pressure. The residue was purified by Prep-TLC (CH_2Cl_2 / MeOH 10:1) to afford 2-(3-fluoropyridin-4-yl)-1*H*,5*H*,6*H*,7*H*-pyrrolo[3,2-c] pyridin-4-one (190 mg, 45%) as a light brown solid. *m/z* (ESI, +ve ion): 232.15 $(M + H)^+$.

Step 4: 2-(3-Fluoropyridin-4-yl)-3-iodo-1*H*,5*H*,6*H*,7*H*-pyrrolo[3,2-c]pyridin-4-one. To a stirred mixture of 2-(3-fluoropyridin-4-yl)-1*H*,5*H*,6*H*,7*H* -pyrrolo[3,2-c]pyridin-4-one (190 mg, 0.820 mmol) in DMF (8.0 mL) was added NIS (221 mg, 0.98 mmol) in three portions at RT under a N_2 atmosphere. The resulting mixture was stirred for additional 2 h at RT. The solid precipitate was collected by filtration and washed with water (3 × 3 mL) to afford 2-(3-fluoropyridin-4-yl)-3-iodo-1*H*,5*H*,6*H*,7*H*-pyrrolo[3,2-c]pyridin-4-one (110 mg, 36%) as a yellow solid. *m/z* (ESI, +ve ion): 357.85 $(M + H)^+$.

Step 5: 3-[(3-Chloro-2-methoxyphenyl)amino]-2-(3-fluoropyridin-4-yl)-1*H*,5*H*,6*H*,7*H*-pyrrolo[3,2-c]pyridin-4-one (Compound 4). 3-Chloro-2-methoxyaniline (44.0 mg, 0.280 mmol), Ephos Pd G4 (51.0 mg, 0.050 mmol), and Cs_2CO_3 (182 mg, 0.560 mmol) were added to a solution of 2-(3-fluoropyridin-4-yl)-3-iodo-1*H*,5*H*,6*H*,7*H*-pyrrolo[3,2-c]pyridin-4-one (100 mg, 0.28 mmol) in 1,4-dioxane (3.0 mL) under an argon atmosphere. The resulting mixture was warmed to 50 °C and stirred for 12 h. The resulting mixture was concentrated under reduced pressure. The residue was purified by silica gel column chromatography, eluted with CH_2Cl_2 / MeOH (10:1) to afford crude product. The crude product (80 mg) was purified by Prep-HPLC (Column: X-select CSH OBD Column 30 × 150 mm, 5 μm; Mobile Phase A: Water (0.05% TFA), Mobile Phase B: MeCN; Flow rate: 60 mL/min; Gradient: 14% B to 34% B in 8 min; Rt: 7 min) to afford 3-[(3-chloro-2-methoxyphenyl)amino]-2-(3-fluoropyridin-4-yl)-1*H*,5*H*,6*H*,7*H*-pyrrolo[3,2-c]pyridin-4-one trifluoroacetic acid salt (12.5 mg, 8.9% yield) as an orange solid. ^{1}H NMR (300 MHz, DMSO-d_6) δ = 11.66 (s, 1H), 8.60 (d, *J* = 3.5 Hz, 1H), 8.32 (d, *J* = 5.3 Hz, 1H), 7.68 (s, 1H), 7.47 (dd, *J* = 7.0, 5.3 Hz, 1H), 7.26 (d, *J* = 7.5 Hz, 1H), 6.78–6.57 (m, 2H), 6.16 (dd, *J* = 7.3, 2.3 Hz, 1H), 3.88 (s, 3H), 3.43 (t, *J* = 7.0 Hz, 2H), 2.89 (t, *J* = 6.8 Hz, 2H). *m/z* (ESI, +ve ion): 387.0 $(M + H)^+$.

Compound 5

3-[(3-Chloro-2-methoxyphenyl)amino]-2-(3-chloropyridin-4-yl)-1*H*,5*H*,6*H*,7*H*-pyrrolo[3,2-c]pyridin-4-one was prepared following the same steps used to synthesize Compound **4**. Step 3 employed 4-bromo-3-chloropyridine, K_3PO_4, and $Pd(dppf)Cl_2$ at 80 °C for 12 h (59% yield). Next, Step 4 (52% yield) followed by Step 5 afforded the title compound (19.5 mg, 18% yield) as a white solid. ^{1}H NMR (400 MHz, DMSO-d_6) δ = 11.60 (s, 1H), 8.58 (s, 1H), 8.42 (d, *J* = 5.1 Hz, 1H), 7.56 (s, 1H), 7.44 (d, *J* = 5.1 Hz, 1H), 7.18 (d, *J* = 2.6 Hz, 1H), 6.68–6.48 (m, 2H), 6.09–6.07 (m, 1H), 3.83 (s, 3H), 3.44 (t, *J* = 6.9, 2.5 Hz, 2H), 2.86 (t, *J* = 6.8 Hz, 2H). *m/z* (ESI, +ve ion): 402.95 $(M + H)^+$.

Compound 6

3-[(3-Chloro-2-methoxyphenyl)amino]-2-[3-(trifluoromethyl)pyridin-4-yl]-1*H*,5*H*,6*H*,7*H*-pyrrolo[3,2-c]pyridin-4-one was prepared following the same steps used to synthesize Compound **4**. Step 3 employed 4-chloro-3-(trifluoromethyl)pyridine hydrochloride, Na_2CO_3 and Xphos Pd G3 at 80 °C for 2 h (93% yield). Next, Step 4 (75% yield) followed by Step 5 afforded the title compound (26.9 mg, 36% yield) as a white solid. ^{1}H NMR (400 MHz, DMSO-d_6) δ = 11.30 (s, 1H), 8.95 (s, 1H), 8.74 (d, *J* = 5.2 Hz, 1H), 7.58 (d, *J* = 5.1 Hz, 1H), 7.26–7.02 (m, 2H), 6.63–6.49 (m, 2H), 6.14 (dd, *J* = 7.2, 2.4 Hz, 1H), 3.78 (s, 3H), 3.42 (td, *J* = 6.9, 2.6 Hz, 2H), 2.85 (t, *J* = 6.8 Hz, 2H). *m/z* (ESI, +ve ion): 437.20 $(M + H)^+$.

Compound 7

3-[(3-Chloro-2-methoxyphenyl)amino]-2-(pyrimidin-4-yl)-1*H*,5*H*,6*H*,7*H*-pyrrolo[3,2-c]pyridin-4-one.

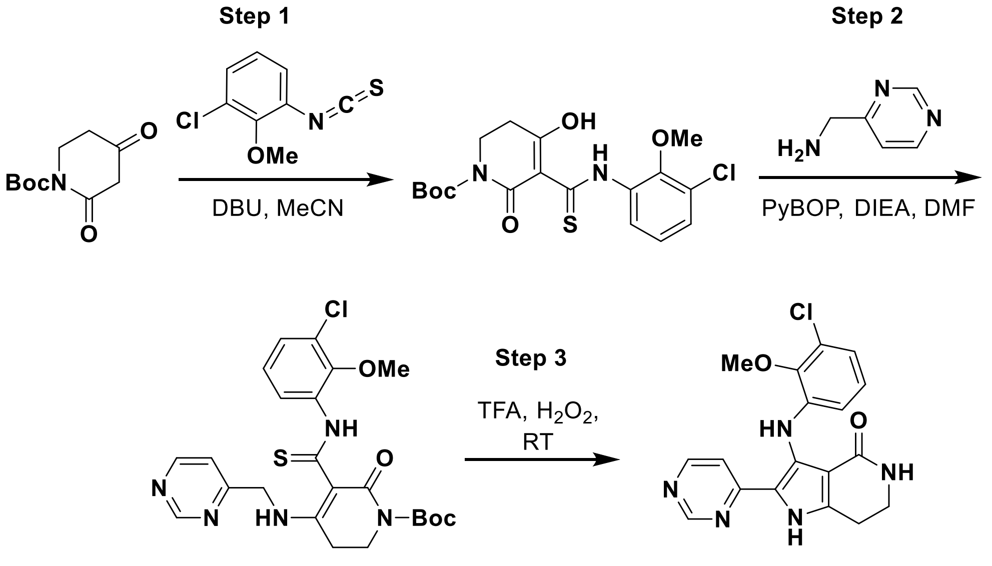

Step 1: *tert*-Butyl 3-[(3-chloro-2-methoxyphenyl)carbamothioyl]-4-hydroxy-2-oxo-5,6-dihydropyridine-1-carboxylate. To a stirred solution of *tert*-butyl 2,4-dioxopiperidine-1-carboxylate (1.00 g, 4.69 mmol) and 1-chloro-3- -2-methoxybenzene (936 mg, 4.69 mmol) in MeCN (6 mL) was added DBU (1.43 g, 9.38 mmol) at RT under a N_2 nitrogen atmosphere. After 3 h, the reaction mixture was concentrated under reduced pressure and the residue was purified by silica gel column chromatography (PE-EtOAc (1:3)) to afford *tert*-butyl 3-[(3-chloro-2-methoxyphenyl)carbamothioyl]-4-hydroxy-2-oxo-5,6-dihydropyridine-1-carboxylate (2.10 g, 92% yield) as a white solid. *m/z* (ESI, +ve ion): 313.0 $(M + H)^+$.

Step 2: *tert*-Butyl 3-[(3-chloro-2-methoxyphenyl)carbamothioyl]-2-oxo-4-[(pyrimidin-4-ylmethyl)amino]-5,6-dihydropyridine-1-carboxylate. PyBOP (8.58 g, 16.5 mmol) and *N*,*N*-diisopropylethylamine (4.26 g, 33.0 mmol) were added to a stirred solution of 1-(pyrimidin-4-yl)methanamine (1.20 g, 11.0 mmol) and *tert*-butyl 3-[(3-chloro-2-methoxyphenyl)carbamothioyl]-4-hydroxy-2-oxo-5,6-dihydropyridine-1-carboxylate (4.54 g, 11.0 mmol) in DMF (40 mL) at RT under a N_2 atmosphere. The resultant reaction mixture was stirred for 12 h, concentrated under reduced pressure and the residue purified by reverse-phase preparative chromatography (C_{18} silica gel; mobile phase, 10–50% water/MeCN, gradient in 10 min; detector, UV 254 nm) afforded *tert*-butyl 3-[(3-chloro-2-methoxyphenyl)carbamothioyl]-2-oxo-4-[(pyrimidin-4-ylmethyl)amino]-5,6-dihydropyridine-1-carboxylate (830 mg, 15% yield) as a red oil. *m/z* (ESI, +ve ion): 504.0 $(M + H)^+$.

Step 3: 3-[(3-Chloro-2-methoxyphenyl)amino]-2-(pyrimidin-4-yl)-1*H*,5*H*,6*H*,7*H*-pyrrolo[3,2-c]pyridin-4-one (Compound 7). TFA (139 mg, 1.22 mmol) and H_2O_2 (0.18 mL, 1.63 mmol, 30% w/v in H_2O) were added to a stirred mixture of *tert*-butyl 3-[(3-chloro-2-methoxyphenyl)carbamothioyl]-2-oxo-4-[(pyrimidin-4-ylmethyl)amino]-5,6-dihydropyridine-1-carboxylate (410 mg, 0.813 mmol) in MeOH (8.0 mL) at RT under a N_2 atmosphere. The resulting mixture was stirred for 12 h then warmed to 80 °C for 2 h. The reaction mixture was concentrated under reduced pressure and the residue was by Prep-HPLC (Column: XBridge Shield RP18 OBD Column, 30 × 150 mm, 5 μm; Mobile Phase A: Water (10 mmol/L NH_4HCO_3), Mobile Phase B: MeCN; Flow rate: 60 mL/min; Gradient: 18% B to 40% B in 10 min; Rt: 10.7 min) to afford 3-[(3-chloro-2-methoxyphenyl)amino]-2-(pyrimidin-4-yl)-1*H*,5*H*,6*H*,7*H*-pyrrolo[3,2-c]pyridin-4-one (31.6 mg, 10% yield) as a light yellow solid. ^{1}H NMR (400 MHz, DMSO-d_6) δ = 12.13 (s, 1H), 9.04 (d, *J* = 1.4 Hz, 1H), 8.55 (d, *J* = 5.6 Hz, 1H), 7.93 (s, 1H), 7.34–7.11 (m, 2H), 6.94–6.74 (m, 2H), 6.33 (dd, *J* = 7.0, 2.6 Hz, 1H), 3.93 (s, 3H), 3.41 (td, *J* = 6.8, 2.6 Hz, 2H), 2.87 (t, *J* = 6.8 Hz, 2H). *m/z* (ESI, +ve ion): 370.0 $(M + H)^+$.

Compound 8

2-(2-Aminopyrimidin-4-yl)-3-[(3-chloro-2-methoxyphenyl)amino]-1*H*,5*H*,6*H*,7*H*-pyrrolo[3,2-c]pyridin-4-one.

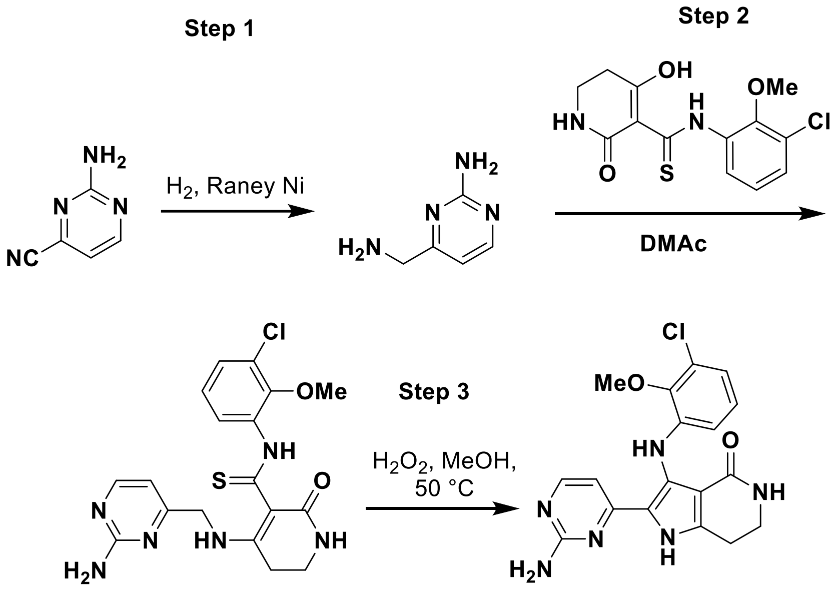

Compound 8

Step 1: 4-(Aminomethyl)pyrimidin-2-amine. To a stirred mixture of 2-aminopyrimidine-4-carbonitrile (1.00 g, 8.33 mmol) in 7 M NH_3 in MeOH (40 mL) was added Raney Ni (4.00 g, 46.7 mmol) at RT under a N_2 atmosphere. The reaction vessel was charged with H_2 (3×) and stirred for 12 h at RT, filtered, and the filtrate concentrated under reduced pressure. The residue was washed with MeOH (3 x 20 mL) and the resulting solid was dried under reduced pressure to afford 4-(aminomethyl)pyrimidin-2-amine (1.00 g, 97% yield) as a purple solid. The product was used in the next step directly without further purification. *m/z* (ESI, +ve ion): 125.0 $(M + H)^+$.

Step 2: 4-[[(2-Aminopyrimidin-4-yl)methyl]amino]-*N*-(3-chloro-2-methoxyphenyl)-2-oxo-5,6-dihydro-1*H*-pyridine-3-carbothioamide. To a stirred mixture of 4-(aminomethyl)pyrimidin-2-amine (1.00 g, 8.06 mmol) and 4 Å MS (1.00 g) in DMA (15 mL) was added *N*-(3-chloro-2-methoxyphenyl)-4-hydroxy-2-oxo-5,6-dihydro-1*H*-pyridine-3-carbothioamide (2.77 g, 8.86 mmol) in portions. The resulting mixture was stirred for 3 h at 120 °C under a N_2 atmosphere. The reaction mixture was allowed to cool RT and filtered. The filter cake was washed with CH_2Cl_2/MeOH (10:1) (3 x 10 mL). The filtrate was concentrated under reduced pressure. The residue was diluted with water (40 mL) and extracted with CH_2Cl_2/MeOH (10:1) (3 x 50 mL). The combined organic layers were washed with brine (3 x 150 mL), dried over anhydrous sodium sulfate, filtered, and the filtrate was concentrated under reduced pressure. The residue was diluted with MeOH (10 mL), the precipitated solids were collected by filtration, and washed with MeOH (3 x 5 mL). The resulting solid was dried under reduced pressure to afford 4-[[(2-aminopyrimidin-4-yl)methyl]amino]-*N*-(3-chloro-2-methoxyphenyl)-2-oxo-5,6-dihydro-1*H*-pyridine-3-carbothioamide (320 mg) as an off-white solid that was used directly without further purification. *m/z* (ESI, +ve ion): 418.95 $(M + H)^+$.

Step 3: 2-(2-Aminopyrimidin-4-yl)-3-[(3-chloro-2-methoxyphenyl)amino]-1*H*,5*H*,6*H*,7*H*-pyrrolo[3,2-c]pyridin-4-one (Compound 8). To a stirred mixture of 4-[[(2-aminopyrimidin-4-yl)methyl]amino]-*N*-(3-chloro-2-methoxyphenyl)-2-oxo-5,6-dihydro-1*H*-pyridine-3-carbothioamide (300 mg, 0.72 mmol) in MeOH (10 mL) was added H_2O_2 (0.37 mL, 3.24 mmol, 30% w/v in H_2O) dropwise. The resulting mixture was stirred for 12 h at 50 °C under a N_2 atmosphere. The resulting mixture was concentrated under reduced pressure. The residue product was purified by Prep-HPLC (Column: XBridge Prep Phenyl OBD Column, 5 µm, 19 x 250mm; Mobile Phase A:Water (10 mmol/L NH_4HCO_3), Mobile Phase B: MeCN; Flow rate: 50 mL/min; Gradient: 10% B to 30% B in 10 min; 254 nm; Rt: 6.0 min) to afford 2-(2-aminopyrimidin-4-yl)-3-[(3-chloro-2-methoxyphenyl)amino]-1*H*,5*H*,6*H*,7*H*-pyrrolo[3,2-c]pyridin-4-one (57.7 mg, 20%) as a light yellow solid. ^{1}H NMR (400 MHz, DMSO-d_6) δ = 11.64 (s, 1H), 8.16–7.91 (m,

2H), 7.13 (s, 1H), 6.89–6.71 (m, 2H), 6.52–6.30 (m, 2H), 6.19 (s, 2H), 3.90 (s, 3H), 3.46–3.38 (m, 2H), 2.84 (t, J = 6.7 Hz, 2H), 2.08 (s, 1H). *m/z* (ESI, +ve ion): 385.10 $(M + H)^+$.

Compound 9

tert-Butyl *N*-(*tert*-butoxycarbonyl)-*N*-[4-[4-oxo-3-(phenylamino)-1*H*,5*H*,6*H*,7*H*-pyrrolo[3,2-c]pyridin-2-yl]pyrimidin-2-yl]carbamate.

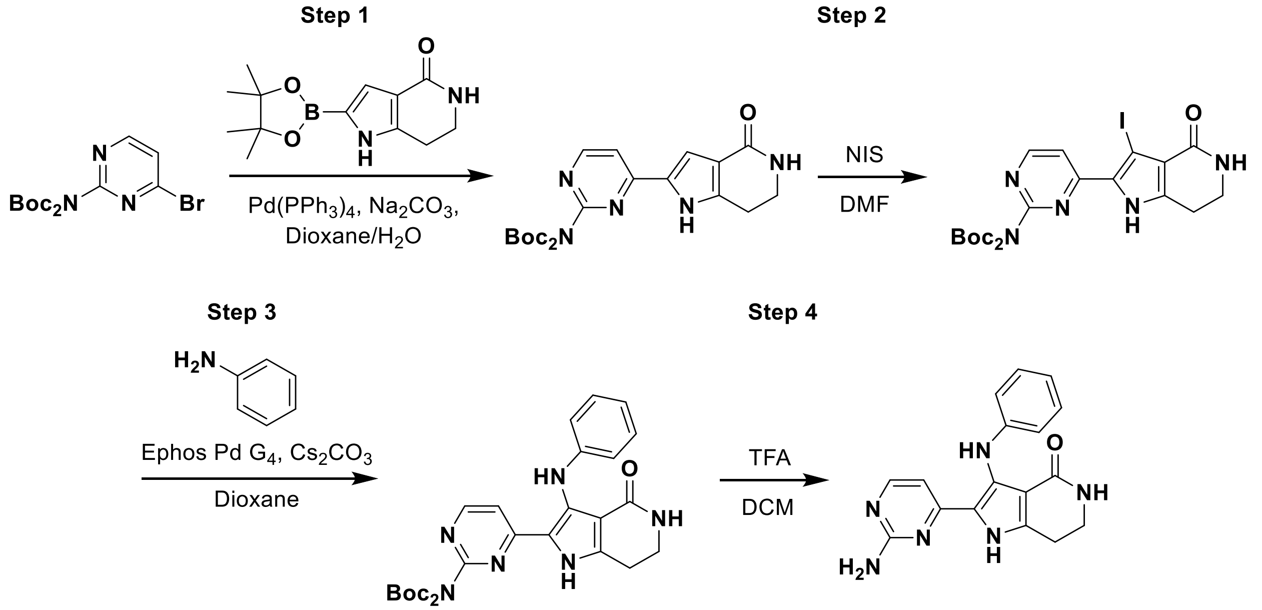

Compound 9

Step 1: *tert*-Butyl *N*-(*tert*-butoxycarbonyl)-*N*-(4-(4-oxo-1*H*,5*H*,6*H*,7*H*-pyrrolo[3,2-c]pyridin-2-yl)pyrimidin-2-yl)carbamate. To a stirred solution of *tert*-butyl *N*-(4-bromopyrimidin-2-yl)-*N*-(*tert*-butoxycarbonyl)carbamate (5.00 g, 13.4 mmol) and 2-(4,4,5,5-tetramethyl-1,3,2-dioxaborolan-2-yl)-1*H*,5*H*,6*H*,7*H*-pyrrolo[3,2-c]pyridin-4-one (4.20 g, 16.0 mmol) in 1,4-dioxane (50 mL) and H_2O (10 mL) were added Pd(PPh3)4 (1.54 g, 1.34 mmol) and Na_2CO_3 (4.29 g, 40.2 mmol) and the resulting mixture was stirred for 10 h at 50 °C under N_2 atmosphere. The resulting mixture was concentrated under reduced pressure and the residue was purified by silica gel column chromatography, eluted with DCM / MeOH (20:1) to *tert*-Butyl *N*-(*tert*-butoxycarbonyl)-*N*-(4-(4-oxo-1*H*,5*H*,6*H*,7*H*-pyrrolo[3,2-c]pyridin-2-yl)pyrimidin-2-yl)carbamate (4.90 g, 85% yield) as a yellow solid. *m/z* (ESI, +ve ion): 430.10 $(M + H)^+$.

Step 2: *tert*-butyl *N*-(*tert*-butoxycarbonyl)-*N*-(4-(3-iodo-4-oxo-1*H*,5*H*,6*H*,7*H*-pyrrolo[3,2-c]pyridin-2-yl)pyrimidin-2-yl)carbamate. A solution of *tert*-Butyl *N*-(*tert*-butoxycarbonyl)-*N*-(4-(4-oxo-1*H*,5*H*,6*H*,7*H*-pyrrolo[3,2-c]pyridin-2-yl)pyrimidin-2-yl)carbamate (500 mg, 1.16 mmol) and NIS (393 mg, 1.75 mmol) in DMF (5.0 mL) was stirred for 4 h at RT under a N_2 atmosphere. The reaction mixture was subsequently concentrated under reduced pressure and the residue was purified by reverse-phase column chromatography (0.1%NH_4HCO_3; 30% MeCN to 80% MeCN in 25 min) to afford *tert*-butyl *N*-(*tert*-butoxycarbonyl)-*N*-(4-(3-iodo-4-oxo-1*H*,5*H*,6*H*,7*H*-pyrrolo[3,2-c]pyridin-2-yl)pyrimidin-2-yl)carbamate (550 mg, 85% yield) as a yellow solid. *m/z* (ESI, +ve ion): 556.00 $(M + H)^+$.

Step 3: *tert*-Butyl *N*-(*tert*-butoxycarbonyl)-N-[4-[4-oxo-3-(phenylamino)-1*H*,5*H*,6*H*,7*H*-pyrrolo[3,2-c]pyridin-2-yl]pyrimidin-2-yl]carbamate. A solution of *tert*-butyl *N*-(*tert*-butoxycarbonyl)-*N*-(4-[3-iodo-4-oxo-1*H*,5*H*,6*H*,7*H*-pyrrolo[3,2-c]pyridin-2-yl]pyrimidin-2-yl)carbamate (200 mg, 0.360 mmol) and aniline (33.5 mg, 0.360 mmol) and Ephos Pd G4 (165 mg, 0.180 mmol) and EPhos (96.3 mg, 0.180 mmol) in dioxane (4.0 mL) was stirred at 50 °C for 12 h under an argon atmosphere. The resultant mixture was concentrated under reduced pressure and diluted with water (10 mL).The aqueous layer was extracted with EtOAc (3 × 10 mL). The combined organic layers were concentrated under reduced pressure and the residue was purified by Prep-TLC (PE /

EA 1:1) to afford *tert*-butyl *N*-(*tert*-butoxycarbonyl)-*N*-[4-[4-oxo-3-(phenylamino)-1*H*,5*H*,6*H*,7*H*-pyrrolo[3,2-c]pyridin-2-yl]pyrimidin-2-yl]carbamate (87.0 mg, 34%) as an orange solid. *m/z* (ESI, +ve ion): 521.15 $(M + H)^+$.

Step 3: 2-(2-Aminopyrimidin-4-yl)-3-(phenylamino)-1*H*,5*H*,6*H*,7*H*-pyrrolo[3,2-c]pyridin-4-one (Compound 9). A solution of *tert*-butyl *N*-(*tert*-butoxycarbonyl)-*N*-[4-[4-oxo-3-(phenylamino)-1*H*,5*H*,6*H*,7*H*-pyrrolo[3,2-c]pyridin-2-yl]pyrimidin-2-yl]carbamate (220 mg, 0.423 mmol) in TFA (1.0 mL) and was stirred for 1 h at RT under a N_2 atmosphere. The resultant mixture was concentrated under reduced pressure and the residue was purified by Prep-HPLC (Column: XBridge Prep OBD C18 Column, 30 × 150 mm, 5 μm; Mobile Phase A: Water (10 mmol/L NH_4HCO_3), Mobile Phase B: MeCN; Flow rate: 60 mL/min; Gradient: 10% B to 40% B in 7 min; Rt: 6.5 min) to afford 2-(2-aminopyrimidin-4-yl)-3-(phenylamino)-1*H*,5*H*,6*H*,7*H*-pyrrolo[3,2-c]pyridin-4-one (61.1 mg, 43%) as a yellow solid. ^{1}H NMR (300 MHz, DMSO-d_6) δ = 11.58 (s, 1H), 8.34 (s, 1H), 8.04 (d, *J* = 5.3 Hz, 1H), 7.17–7.01 (m, 3H), 6.79 (d, *J* = 8.0 Hz, 2H), 6.69 (t, *J* = 7.3 Hz, 1H), 6.56 (d, *J* = 5.4 Hz, 1H), 6.39 (s, 2H), 3.41 (dd, *J* = 6.7, 2.5 Hz, 2H), 2.84 (t, *J* = 6.7 Hz, 2H), 2.08 (s, 1H). *m/z* (ESI, +ve ion): 321.30 $(M + H)^+$.

Compound 10

2-(2-Aminopyrimidin-4-yl)-3-[(2-chlorophenyl)amino]-1*H*,5*H*,6*H*,7*H*-pyrrolo[3,2-c]pyridin-4-one was prepared according to the procedure described for Compound **9**, using 2-chloroaniline in Step 3 heating at 50 °C for 12 h (quantitative yield). Boc deprotection Step 4 proceeded at RT in 3 h to afford the TFA salt of the title compound (48.7 mg, 52% yield) as a yellow solid. ^{1}H NMR (400 MHz, DMSO-d_6) δ = 12.11 (s, 1H), 8.93 (s, 1H), 8.07 (d, *J* = 6.7 Hz, 1H), 7.55 (s, 2H), 7.40 (dd, *J* = 7.9, 1.4 Hz, 1H), 7.30 (s, 1H), 7.13–7.06 (m, 1H), 6.97–6.81 (m, 2H), 6.74 (d, *J* = 6.7 Hz, 1H), 3.46–3.37 (m, 2H), 2.90 (t, *J* = 6.6 Hz, 2H). ^{19}F NMR (400 MHz, DMSO-d_6) δ = –73.99. *m/z* (ESI, +ve ion): 355.00 $(M + H)^+$.

Compound 11

2-(2-Aminopyrimidin-4-yl)-3-[(3-chlorophenyl)amino]-1*H*,5*H*,6*H*,7*H*-pyrrolo[3,2-c]pyridin-4-one was prepared according to the procedure described for Compound **9**, using 3-chloroaniline in Step 3 heating at 50 °C for 2 h (25% yield). Boc deprotection Step 4 proceeded at RT in 3 h to afford the TFA salt of the title compound (28.6 mg, 66% yield) as a yellow solid. ^{1}H NMR (300 MHz, DMSO-d_6) δ = 12.19 (s, 1H), 9.26 (s, 1H), 8.15–7.91 (m, 3H), 7.30 (s, 1H), 7.15 (t, *J* = 8.1 Hz, 1H), 7.03 (d, *J* = 2.1 Hz, 1H), 6.96–6.67 (m, 3H), 3.46–3.38 (m, 2H), 2.95–2.85 (m, 2H). ^{19}F NMR (300 MHz, DMSO-d_6) δ = –73.97. *m/z* (ESI, +ve ion): 355.0 $(M + H)^+$.

Compound 12

2-(2-Aminopyrimidin-4-yl)-3-[(4-chlorophenyl)amino]-1*H*,5*H*,6*H*,7*H*-pyrrolo[3,2-c]pyridin-4-one was prepared according to the procedure described for Compound **9**, using 4-chloroaniline in Step 3 heating at 50 °C for 3 h (50% yield). Boc deprotection Step 4 proceeded at RT in 1 h to afford the TFA salt of the title compound (28.6 mg, 66% yield) as a yellow solid. ^{1}H NMR (400 MHz, DMSO-d_6) δ = 12.10 (s, 1H), 9.21 (s, 1H), 8.05 (d, 1H), 7.89 (s, 2H), 7.21–7.16 (m, 3H), 6.98–6.95 (m, 2H), 6.84 (d, *J* = 6.8 Hz, 1H), 3.43–3.39 (m, 2H), 2.88 (t, *J* = 6.6 Hz, 2H). ^{19}F NMR (300 MHz, DMSO-d_6) δ = –73.83. *m/z* (ESI, +ve ion): 355.05 $(M + H)^+$.

Compound 13

2-(2-Aminopyrimidin-4-yl)-3-[(3-fluoro-2-methoxyphenyl)amino]-1*H*,5*H*,6*H*,7*H*-pyrrolo[3,2-c]pyridin-4-one.

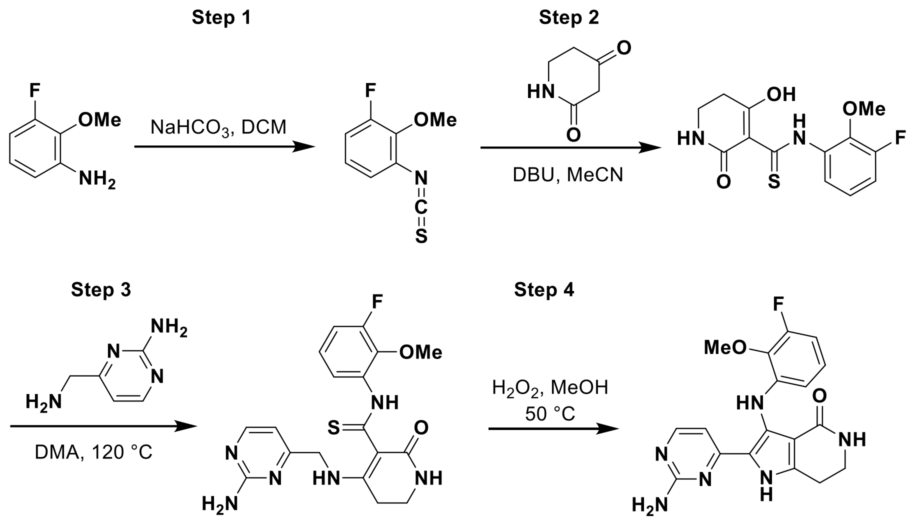

Compound 13

Step 1: 1-Fluoro-3-isothiocyanato-2-methoxybenzene. To a stirred solution of 3-fluoro-2-methoxyaniline (7.00 g, 49.6 mmol) and $NaHCO_3$ (5.22 g, 49.6 mmol) in DCM (42 mL) and H_2O (42 mL) was added thiophosgene (5.70 g, 49.6 mmol) dropwise at 0 °C under a N_2 atmosphere. The resulting mixture was stirred for 1 h at RT under a N_2 atmosphere. The reaction mixture was then neutralized to pH 7 with sat. aq. $NaHCO_3$. The layers were separated and the aqueous layer was extracted with DCM (3 × 25 mL). The combined organic layers were washed with brine and dried over anhydrous magnesium sulfate, filtered, and concentrated under reduced pressure. The residue was purified by silica gel column chromatography, eluted with PE/EtOAc (12:1) to afford 1-fluoro-3-isothiocyanato-2-methoxybenzene (7.90 g, 87% yield) as a dark yellow solid.

Step 2: *N*-(3-Fluoro-2-methoxyphenyl)-4-hydroxy-2-oxo-5,6-dihydro-1*H*-pyridine-3-carbothioamide. To a stirred mixture of 1-fluoro-3-isothiocyanato-2-methoxybenzene (6.00 g, 32.8 mmol) and piperidine-2,4-dione (3.70 g, 32.8 mmol) in MeCN (60 mL) was added DBU (7.48 g, 49.1 mmol) dropwise at 0 °C under a N_2 atmosphere. The resulting mixture was stirred for 2 h at RT. The mixture was acidified to pH 6 with aq. 1M HCl. The precipitated solids were collected by filtration and washed with MeCN (10 mL) to afford *N*-(3-Fluoro-2-methoxyphenyl)-4-hydroxy-2-oxo-5,6-dihydro-1*H*-pyridine-3-carbothioamide (6.20 g, 64% yield) as a light yellow solid. *m/z* (ESI, +ve ion): 296.95 $(M + H)^+$.

Step 3: 4-[[(2-Aminopyrimidin-4-yl) methyl]amino]-*N*-(3-fluoro-2-methoxyphenyl)-2-oxo-5,6-dihydro-1*H*-pyridine-3-carbothioamide. To a stirred solution of 4-(aminomethyl)pyrimidin-2-amine (500 mg, 4.03 mmol) in DMA (5.00 mL) was added *N*-(3-Fluoro-2-methoxyphenyl)-4-hydroxy-2-oxo-5,6-dihydro-1*H*-pyridine-3-carbothioamide (1.31 g, 4.43 mmol) at RT under a N_2 atmosphere. The resulting mixture was stirred for 2 h at 120 °C and the reaction mixture was concentrated under reduced pressure. The residue was purified by silica gel column chromatography, eluted with PE/EA=1:3 to afford 4-[[(2-aminopyrimidin-4-yl) methyl]amino]-*N*-(3-fluoro-2-methoxyphenyl)-2-oxo-5,6-dihydro-1*H*-pyridine-3-carbothioamide (850 mg, 47% yield) as a white solid. *m/z* (ESI, +ve ion): 403.05 $(M + H)^+$.

Step 4: 2-(2-Aminopyrimidin-4-yl)-3-[(3-fluoro-2-methoxyphenyl)amino]-1*H*,5*H*,6*H*,7*H*-pyrrolo[3,2-c]pyridin-4-one (Compound 13). To a stirred solution of 4-[[(2-aminopyrimidin-4-yl) methyl]amino]-*N*-(3-fluoro-2-methoxyphenyl)-2-oxo-5,6-dihydro-1*H*-pyridine-3-carbothioamide (50.0 mg, 0.124 mmol) in MeOH (1.00 mL) was added H_2O_2 (14.2 µL, 0.124 mmol, 30% w/v in H_2O) at RT under a N_2 atmosphere. The resulting mixture was stirred for 2 h at 80 °C. The reaction mixture was allowed to cool to RT and was concentrated under reduced pressure. The residue was purified by silica gel column chromatography, eluted with PE/EA=1:3 to afford 2-(2-aminopyrimidin-4-yl)-3-[(3-fluoro-2-methoxyphenyl)amino]-1*H*,5*H*,6*H*,7*H*-pyrrolo[3,2-c]pyridin-4-one (18.0 mg, 38% yield) as a white solid. ^{1}H NMR (400 MHz, DMSO-d_6) δ = 11.64 (s, 1H), 8.16 (s, 1H), 7.91 (s, 1H), 7.23 (s, 1H), 6.69 (s, 1H), 6.52–6.29 (m, 2H), 6.15 (s, 3H), 3.94 (s, 3H), 3.45 (s, 2H), 2.89 (s, 2H). *m/z* (ESI, +ve ion): 369.10 $(M + H)^+$.

Compound 14

2-(2-Aminopyrimidin-4-yl)-3-[(2-methoxy-3-methylphenyl)amino]-1*H*,5*H*,6*H*,7*H*-pyrrolo[3,2-c]pyridin-4-one was prepared according to the procedure described for Compound **9**, using 2-methoxy-3-methylaniline in Step 3 heating at 50 °C for 12 h (25% yield). Boc deprotection Step 4 proceeded at RT in 3 h to afford the formic acid salt of the title compound (28.6 mg, 66% yield) as a yellow solid after purification. ^{1}H NMR (400 MHz, DMSO-d_6) δ = 11.56 (s, 1H), 8.21 (s, 1H), 8.01 (d, *J* = 5.4 Hz, 1H), 7.78 (s, 1H), 7.16 (t, *J* = 2.6 Hz, 1H), 6.70 (t, *J* = 7.8 Hz, 1H), 6.59–6.52 (m, 1H), 6.38 (d, *J* = 5.3 Hz, 1H), 6.31–6.07 (m, 3H), 3.81 (s, 3H), 3.39–3.36 (m, 2H), 2.84 (t, *J* = 6.8 Hz, 2H), 2.24 (s, 3H). *m/z* (ESI, +ve ion): 365.10 $(M + H)^+$.

Compound 15

2-(2-Aminopyrimidin-4-yl)-3-[[2-methoxy-3-(trifluoromethyl)phenyl]amino]-1*H*,5*H*,6*H*,7*H*-pyrrolo[3,2-c]pyridin-4-one was prepared according to the procedure described for Compound **9**, using 2-methoxy-3-(trifluoromethyl) aniline in Step 3 heating at 40 °C for 12 h (16% yield). Boc deprotection Step 4 proceeded at RT in 2 h to afford the title compound (38.3 mg, 90% yield) as a white solid after purification. ^{1}H NMR (400 MHz, DMSO-d_6) δ = 11.70 (s, 1H), 8.18 (s, 1H), 8.08 (d, *J* = 5.4 Hz, 1H), 7.15 (d, *J* = 2.7 Hz, 1H), 7.05–6.89 (m, 2H), 6.75 (dd, *J* = 7.5, 2.3 Hz, 1H), 6.52 (d, *J* = 5.3 Hz, 1H), 6.19 (s, 2H), 3.92 (s, 3H), 3.40 (td, *J* = 6.7, 2.5 Hz, 2H), 2.86 (t, *J* = 6.7 Hz, 2H). *m/z* (ESI, +ve ion): 419.00 $(M + H)^+$.

Compound 16

2-(2-Aminopyrimidin-4-yl)-3-[(3-chloro-2-fluorophenyl)amino]-1*H*,5*H*,6*H*,7*H*-pyrrolo[3,2-c]pyridin-4-one was prepared according to the procedure described for Compound **9**, using 3-chloro-2-fluoroaniline in Step 3 heating at 50 °C for 2 h (63% yield). Boc deprotection Step 4 proceeded at RT in 1 h to afford the title compound (7.90 mg, 18% yield) as a white solid after purification. ^{1}H NMR (400 MHz, DMSO-d_6) δ = 11.71 (s, 1H), 8.25 (s, 1H), 8.10 (d, *J* = 5.4 Hz, 1H), 7.11 (s, 1H), 6.89–6.82 (m, 2H), 6.63 (d, *J* = 5.4 Hz, 1H), 6.57–6.52 (m, 1H), 6.29 (s, 2H), 3.41–3.34 (m, 2H), 2.84 (t, *J* = 6.7 Hz, 2H). *m/z* (ESI, +ve ion): 373.00 $(M + H)^+$.

Compound 17

2-(2-Aminopyrimidin-4-yl)-3-[(3-chloro-2-methylphenyl)amino]-1*H*,5*H*,6*H*,7*H*-pyrrolo[3,2-c]pyridin-4-one was prepared according to the procedure described for Compound **9**, using 2-methyl-3-chloroaniline in Step 3 heating at 50 °C for 2 h (49% yield). Boc deprotection Step 4 proceeded at 50 °C in 2 h to afford the trifluoroacetic acid salt of the title compound (13.2 mg, 31% yield) as a yellow solid after purification. ^{1}H NMR (400 MHz, DMSO-d_6) δ = 11.97 (s, 1H), 8.90 (s, 1H), 8.00 (d, *J* = 8.0 Hz, 1H), 7.67 (s, 2H), 7.11 (s, 1H), 7.00–6.98 (m, 2H), 6.77 (s, 2H), 3.38–3.35 (m, 2H), 2.85 (t, *J* = 8.0 Hz, 2H), 2.36 (s, 3H). ^{19}F NMR (400 MHz, DMSO-d_6) δ = –73.92. *m/z* (ESI, +ve ion): 369.05 $(M + H)^+$.

Compound 18

2-(2-Aminopyrimidin-4-yl)-3-[(3-chloro-2-ethylphenyl)amino]-1*H*,5*H*,6*H*,7*H*-pyrrolo[3,2-c]pyridin-4-one was prepared according to the procedure described for Compound **9**, using 3-chloro-2-ethylaniline in Step 3 heating at 50 °C for 2 h (66% yield). Boc deprotection Step 4 proceeded at 50 °C in 2 h to afford the trifluoroacetic acid salt of the title compound (64.1 mg, 55% yield) as a yellow solid after purification. ^{1}H NMR (400 MHz, DMSO-d_6) δ = *δ* 11.93 (s, 1H), 8.71 (s, 1H), 7.99 (d, *J* = 6.8 Hz, 1H), 7.62 (s, 2H), 7. 11 (s, 1H), 7.01–6.95 (m, 2H), 6.73–6.67 (m, 2H), 3.39–3.36 (m, 2H), 2.88–2.83 (m, 4H), 1.17 (t, *J* = 8.0 Hz, 3H). ^{19}F NMR (400 MHz, DMSO-d_6) δ = –73.93. *m/z* (ESI, +ve ion): 383.05 $(M + H)^+$.

Compound 19

2-(2-Aminopyrimidin-4-yl)-3-[(3-chloro-2-ethoxyphenyl)amino]-1*H*,5*H*,6*H*,7*H*-pyrrolo[3,2-c]pyridin-4-one was prepared according to the procedure described for Compound **9**, using 3-chloro-2-ethoxyaniline in Step 3 heating at 50 °C for 2 h (65% yield). Boc deprotection Step 4 proceeded at RT in 1 h to afford the title compound (25.2 mg, 55% yield) as a yellow solid after purification. ^{1}H NMR (400 MHz, DMSO-d_6) δ = *δ* 11.94 (s, 1H), 8.46 (s, 1H), 8.03 (d, *J* = 6.4 Hz, 1H), 7.30 (s, 2H), 7.26 (s, 1H), 6.91–6.84 (m,

2H), 6.62–6.53 (m, 2H), 6.11–6.06 (m, 2H), 3.42–3.39 (m, 2H), 2.87 (t, *J* = 6.6 Hz, 2H), 1.38 (t, *J* = 3.5 Hz, 3H). ^{19}F NMR (400 MHz, DMSO-d_6) δ = −73.75. *m/z* (ESI, +ve ion): 399.30 (M + H)$^+$.

Compounds 20 and 21

(*S*)-3-((3-Chloro-2-methoxyphenyl)amino)-2-(3-fluoropyridin-4-yl)-7-(methoxymethyl)-1,5,6,7-tetrahydro-4*H*-pyrrolo[3,2-c]pyridin-4-one (Compound **21**) and (*R*)-3-((3-chloro-2-methoxyphenyl)amino)-2-(3-fluoropyridin-4-yl)-7-(methoxymethyl)-1,5,6,7-tetrahydro-4*H*-pyrrolo[3,2-c]pyridin-4-one (Compound **20**).

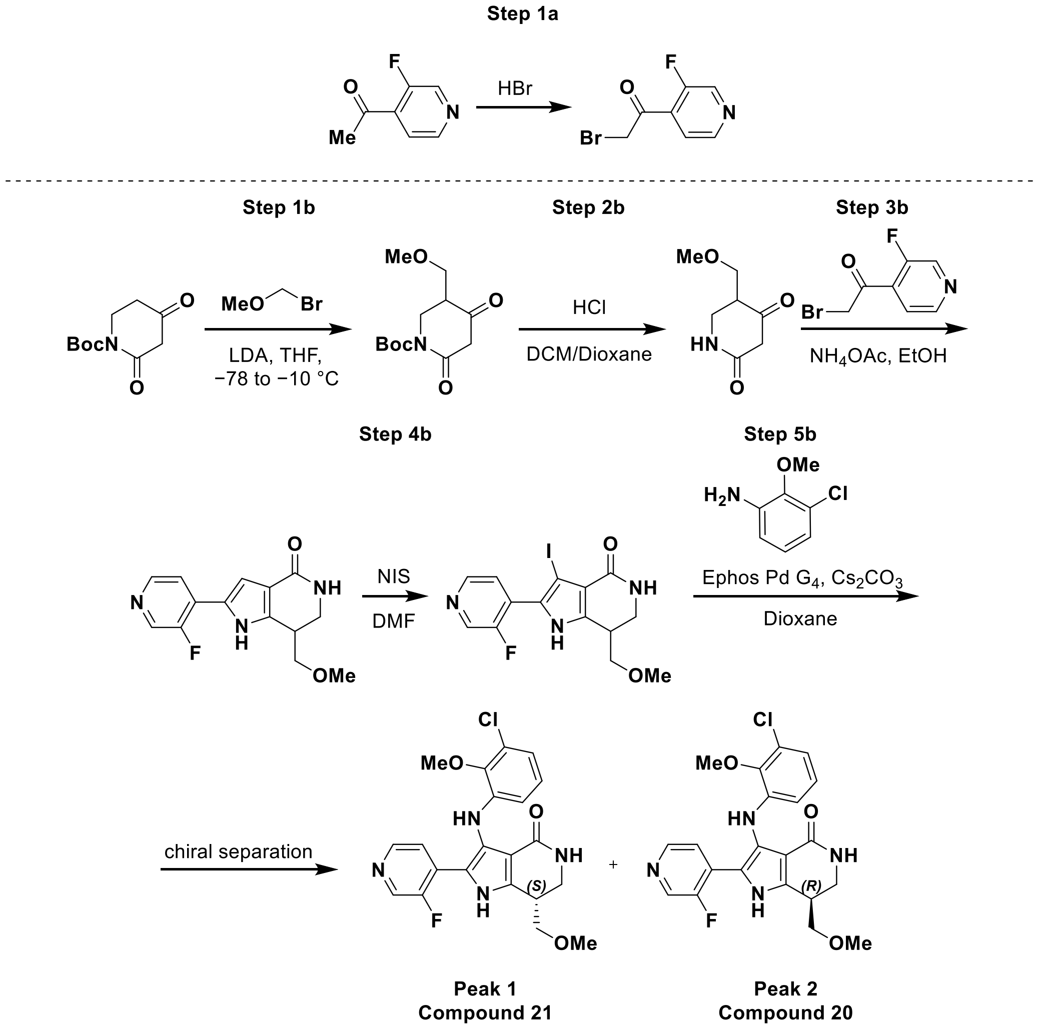

Step 1a: 2-Bromo-1-(3-fluoropyridin-4-yl)ethenone. To a stirred solution of 1-(3-fluoropyridin-4-yl)ethanone (15.0 g, 108 mmol) in AcOH (45 mL) and HBr (15 mL, 48 wt. % in H_2O) was added Br_2 (17.23 g, 108 mmol) in AcOH (15 mL) dropwise at RT. The resulting mixture was stirred for 3 h at 65 °C, cooled to RT and diluted with EtOAc (20 mL). After 20 min stirring, the precipitated solids were collected by filtration to afford 2-bromo-1-(3-fluoropyridin-4-yl)ethanone (6.00 g, 26% yield) as an off-white solid. *m/z* (ESI, +ve ion): 217.80 (M + H)$^+$.

Step 1b: *tert*-Butyl 5-(methoxymethyl)-2,4-dioxopiperidine-1-carboxylate. To a stirred solution of *tert*-butyl 2,4-dioxopiperidine-1-carboxylate (1.0 g, 4.69 mmol) in THF (30 mL) was added LDA (1.51 g, 14.1 mmol, 2 M THF) dropwise at −78 °C under a N_2 atmosphere. After 30 min, bromo(methoxy)methane (0.70 g, 5.63 mmol) was added dropwise over 30 sec at −78 °C.

The resulting reaction mixture was gradually allowed to warm to 10 °C. The reaction stirred for 1 h at 10 °C before it was acidified to pH 4 with aq. HCl (3M). The resulting mixture was extracted with EtOAc (3 × 20 mL). The combined organic layers were washed with brine (3 × 30 mL), dried over anhydrous sodium sulfate, filtered, and concentrated under reduced pressure. The residue was purified by Prep-TLC (PE: EA=1:1) to afford *tert*-butyl 5-(methoxymethyl)-2,4-dioxopiperidine-1-carboxylate (260 mg, 22% yield) as a yellow oil. *m/z* (ESI, +ve ion): 258.15 $(M + H)^+$.

Step 2b: 5-(Methoxymethyl) piperidine-2,4-dione. To a stirred solution of *tert*-butyl 5-(methoxymethyl)-2,4-dioxopiperidine-1-carboxylate (260 mg, 1.01 mmol) in DCM (3 mL) was added HCl in 1,4-dioxane (1.5 mL, 1.50 mmol, 1 M) dropwise at RT. After 2 h, the reaction mixture was concentrated under reduced pressure to afford 5-(methoxymethyl) piperidine-2,4-dione (150 mg, 94% yield) as a yellow solid which was used without further purification. *m/z* (ESI, +ve ion): 158.15 $(M + H)^+$.

Step 3b: 2-(3-Fluoropyridin-4-yl)-7-(methoxymethyl)-1*H*,5*H*,6*H*,7*H*-pyrrolo[3,2-c]pyridin-4-one. To a stirred solution of 5-(methoxymethyl)piperidine-2,4-dione (150 mg, 0.954 mmol) and 2-bromo-1-(3-fluoropyridin-4-yl)ethanone (250 mg, 1.15 mmol) in EtOH (2 mL) was added NH_4OAc (368 mg, 4.77 mmol) in portions at RT. The resultant solution was stirred at 50 °C under a N_2 atmosphere for 16 h. The resulting mixture was concentrated under reduced pressure and the residue was purified by Prep-TLC (DCM:MeOH=15:1) to afford 2-(3-fluoropyridin-4-yl)-7-(methoxymethyl)-1*H*,5*H*,6*H*,7*H*-pyrrolo[3,2-c]pyridin-4-one (75.0 mg, 29% yield) as a yellow solid. *m/z* (ESI, +ve ion): 276.15 $(M + H)^+$.

Step 4b: 2-(3-Fluoropyridin-4-yl)-3-iodo-7-(methoxymethyl)-1*H*,5*H*,6*H*,7*H*-pyrrolo[3,2-c]pyridin-4-one. To a stirred solution of 2-(3-fluoropyridin-4-yl)-7-(methoxymethyl)-1*H*,5*H*,6*H*,7*H*-pyrrolo[3,2-c]pyridin-4-one (75 mg, 0.272 mmol) in DMF (1.0 mL) was added *N*-iodosuccinimide (61.3 mg, 0.354 mmol) in portions at RT. The resulting solution stirred at RT for 2 h and was then concentrated under reduced pressure. The residue was purified by Prep-TLC (DCM:MeOH=15:1) to afford 2-(3-fluoropyridin-4-yl)-3-iodo-7-(methoxymethyl)-1*H*,5*H*,6*H*,7*H*-pyrrolo[3,2-c]pyridin-4-one (90.0 mg, 82% yield) as a yellow solid. *m/z* (ESI, +ve ion): 402.00 $(M + H)^+$.

Step 5b: (*S*)-3-((3-Chloro-2-methoxyphenyl)amino)-2-(3-fluoropyridin-4-yl)-7-(methoxymethyl)-1,5,6,7-tetrahydro-4*H*-pyrrolo[3,2-c]pyridin-4-one (Compound 21) and (*R*)-3-((3-chloro-2-methoxyphenyl)amino)-2-(3-fluoropyridin-4-yl)-7-(methoxymethyl)-1,5,6,7-tetrahydro-4*H*-pyrrolo[3,2-c]pyridin-4-one (Compound 20). To a stirred solution of 2-(3-fluoropyridin-4-yl)-3-iodo-7-(methoxymethyl)-1*H*,5*H*,6*H*,7*H*-pyrrolo[3,2-c]pyridin-4-one (90.0 mg, 0.224 mmol) and 3-chloro-2-methoxyaniline (70.7 mg, 0.448 mmol) in DMF (1.5 mL) was added EPhos Pd G4 (30.9 mg, 0.034 mmol) and Cs_2CO_3 (146 mg, 0.448 mmol) at RT under a N_2 atmosphere. The resultant reaction mixture was warmed 50 °C and stirred for 3 h. The resultant reaction mixture was concentrated under reduced pressure and the residue was purified by Prep-TLC (DCM:MeOH=15:1) to afford crude product (120 mg) that was further purified by Prep-HPLC (Column: YMC-Actus Triart C18, 30 ×150 mm, 5 μm; Mobile Phase A: Water (10 mmol/L NH_4HCO_3+0.1%NH_3 in H_2O), Mobile Phase B: MeCN; Flow rate: 60 mL/min; Gradient: 32% B to 62% B in 8 min; Rt: 6.78 min) to afford *rac*-3-[(3-chloro-2-methoxyphenyl)amino]-2-(3-fluoropyridin-4-yl)-7-(methoxymethyl)-1*H*,5*H*,6*H*,7*H*-pyrrolo[3,2-c]pyridin-4-one (50 mg, 52% yield) as a yellow solid. The solid was further purified by Prep-Chiral-HPLC (Column: CHIRALPAK IG, 2 × 25 cm, 5 μm; Mobile Phase A: hexanes (0.5% 2 M NH_3-MeOH), Mobile Phase B: EtOH; Flow rate: 20 mL/min; Gradient: 30% B in 37 min) to afford (*S*)-3-((3-chloro-2-methoxyphenyl)amino)-2-(3-fluoropyridin-4-yl)-7-(methoxymethyl)-1,5,6,7-tetrahydro-4*H*-pyrrolo[3,2-c]pyridin-4-one (Rt = 3.27 min, peak 1, 10.2 mg, 20% yield) as the first-eluting peak as a white solid and (*R*)-3-((3-chloro-2-methoxyphenyl)amino)-2-(3-fluoropyridin-4-yl)-7-(methoxymethyl)-1,5,6,7-tetrahydro-4*H*-pyrrolo[3,2-c]pyridin-4-one was afforded as the second-eluting peak one (Rt = 5.30 min, peak 2, 13.7 mg, 27% yield) as a white solid. ^{1}H NMR (400 MHz, DMSO-d_6) δ = 11.57 (s, 1H), 8.51 (d, *J* = 2.8 Hz, 1H), 8.29 (dd, *J* = 5.0, 1.2 Hz, 1H), 7.58 (s, 1H), 7.44 (dd, *J* = 6.8, 5.1 Hz, 1H), 7.19–7.04 (m, 1H), 6.73–6.59 (m, 2H), 6.12 (dd, *J* = 7.3, 2.4 Hz, 1H), 3.86 (s, 3H), 3.54–3.47 (m, 3H), 3.36 (dt, *J* = 12.5, 3.8 Hz, 4H), 3.25 (ddd, *J* = 9.1, 5.6, 2.9 Hz, 1H). *m/z* (ESI, +ve ion): 430.95 $(M + H)^+$. Absolute stereochemistry (*R* vs. *S*) was assigned based on biological activity analogy to Compound **23** and **22** whose stereochemistry is assigned crystallographically.

Compounds 22 and 23

(*S*)-3-((3-Chloro-2-methoxyphenyl)amino)-2-(3-fluoropyridin-4-yl)-7-(2-methoxyethyl)-1,5,6,7-tetrahydro-4*H*-pyrrolo[3,2-c]pyridin-4-one (Compound **23**) and **(*R*)-3-((3-chloro-2-methoxyphenyl)amino)-2-(3-fluoropyridin-4-yl)-7-(2-methoxyethyl)-1,5,6,7-tetrahydro-4*H*-pyrrolo[3,2-c]pyridin-4-one** (Compound **22**) were prepared according to the

procedure described for Compounds **20** and **21**, using 2-bromoethyl methyl ether as the electrophile and LiHMDS as a base in Step 1b at −70 to −10 °C over 3 h (74% yield), Step 2b (quantitative yield), Step 3b (63% yield), Step 4b (77% yield), and Step 5b afforded the racemate of the title compounds (600 mg, 80% yield) as a yellow solid after Prep-TLC (DCM:MeOH=0 to 10:1). Chiral separation was then conducted via Prep-Chiral-HPLC (Column: CHIRALPAK IG-3, 4.6 × 50 mm, 3 μm; Mobile Phase: (Hex: DCM=3: 1)(0.1% DEA:IPA=70:30; Flow rate: 1 mL/min) to afford (*S*)-3-((3-chloro-2-methoxyphenyl)amino)-2-(3-fluoropyridin-4-yl)-7-(2-methoxyethyl)-1,5,6,7-tetrahydro-4*H*-pyrrolo[3,2-c]pyridin-4-one (Rt = 2.90 min, peak 1, 47.8 mg, 28% yield) as the first-eluting peak as a white solid and (*R*)-3-((3-chloro-2-methoxyphenyl)amino)-2-(3-fluoropyridin-4-yl)-7-(2-methoxyethyl)-1,5,6,7-tetrahydro-4*H*-pyrrolo[3,2-c]pyridin-4-one (Rt = 4.23 min, peak 2, 55.1 mg, 32% yield) as the second-eluting peak as a white solid. ^{1}H NMR (400 MHz, DMSO-d_6) δ = 11.49 (s, 1H), 8.51 (d, *J* = 3.0 Hz, 1H), 8.29 (dd, *J* = 5.1, 1.2 Hz, 1H), 7.59 (s, 1H), 7.44 (dd, *J* = 6.8, 5.1 Hz, 1H), 7.18 (t, *J* = 2.7 Hz, 1H), 6.73–6.60 (m, 2H), 6.13 (dd, *J* = 7.3, 2.3 Hz, 1H), 3.86 (s, 3H), 3.57–3.41 (m, 3H), 3.30 (s, 3H), 3.24 (ddd, *J* = 12.5, 6.1, 3.1 Hz, 1H), 3.09 (dq, *J* = 11.0, 5.5 Hz, 1H), 2.07–1.95 (m, 1H), 1.78 (td, *J* = 13.9, 5.6 Hz, 1H). *m/z* (ESI, +ve ion): 445.00 (M + H)$^+$. Absolute stereochemistry (*R* vs. *S*) assigned crystallographically.

Compounds 24 and 25

(*S*)-3-((3-Chloro-2-methoxyphenyl)amino)-2-(3-fluoropyridin-4-yl)-7-(3-methoxypropyl)-1,5,6,7-tetrahydro-4*H*-pyrrolo[3,2-c]pyridin-4-one (Compound **25**) and **(*R*)-3-((3-chloro-2-methoxyphenyl)amino)-2-(3-fluoropyridin-4-yl)-7-(3-methoxypropyl)-1,5,6,7-tetrahydro-4*H*-pyrrolo[3,2-c]pyridin-4-one** (Compound **24**) were prepared according to the procedure described for Compounds **20** and **21**, using 1-bromo-3-methoxypropane as the electrophile and LDA as a base in Step 1b at −78 °C to RT over 30 min (27% yield), Step 2b (quantitative yield), Step 3b (46% yield), Step 4b (70% yield), and Step 5b afforded the racemate of the title compounds (165 mg, 62% yield) as a light yellow solid after Prep-TLC (DCM:MeOH = 15:1). Chiral separation was then conducted via Prep-Chiral-HPLC (Column: CHIRALPAK IG-3, 4.6 × 50 mm, 3 μm; Mobile Phase: (Hex: DCM=3: 1)(0.1%DEA): EtOH=80:20; Flow rate: 1 mL/min) to afford (*S*)-3-((3-chloro-2-methoxyphenyl)amino)-2-(3-fluoropyridin-4-yl)-7-(3-methoxypropyl)-1,5,6,7-tetrahydro-4*H*-pyrrolo[3,2-c]pyridin-4-one (Rt = 3.21 min, peak 1, 18.6 mg, 37% yield) as the first-eluting peak as a white solid and (*R*)-3-((3-chloro-2-methoxyphenyl)amino)-2-(3-fluoropyridin-4-yl)-7-(3-methoxypropyl)-1,5,6,7-tetrahydro-4*H*-pyrrolo[3,2-c]pyridin-4-one (Rt = 4.27 min, peak 2, 15.1 mg, 30% yield) as the second-eluting peak as a white solid. ^{1}H NMR (300 MHz, $CDCl_3$) δ 10.34 (d, *J* = 6.7 Hz, 1H), 8.46 (d, *J* = 4.4 Hz, 1H), 8.13 (d, *J* = 5.5 Hz, 1H), 7.69 (s, 1H), 7.36 (dd, *J* = 7.3, 5.4 Hz, 1H), 6.78 (dd, *J* = 8.1, 1.5 Hz, 1H), 6.66 (t, *J* = 8.1 Hz, 1H), 6.20 (dd, *J* = 8.1, 1.5 Hz, 1H), 5.25 (d, *J* = 3.9 Hz, 1H), 4.08 (s, 3H), 3.88–3.76 (m, 1H), 3.69 (t, *J* = 7.0 Hz, 1H), 3.51 (s, 4H), 3.32–3.17 (m, 2H), 2.10 (td, *J* = 9.9, 8.3, 4.3 Hz, 1H), 1.75–1.59 (m, 3H). *m/z* (ESI, +ve ion): 459.00 (M + H)$^+$. Absolute stereochemistry (*R* vs. *S*) was assigned based on biological activity analogy to Compound **23** and **22** whose stereochemistry is assigned crystallographically.

Compound 26

3-[(3-chloro-2-methoxyphenyl)amino]-2-(3-fluoropyridin-4-yl)-7-(2-methoxyethyl)-1*H*,5*H*-pyrrolo[3,2-c]pyridin-4-one.

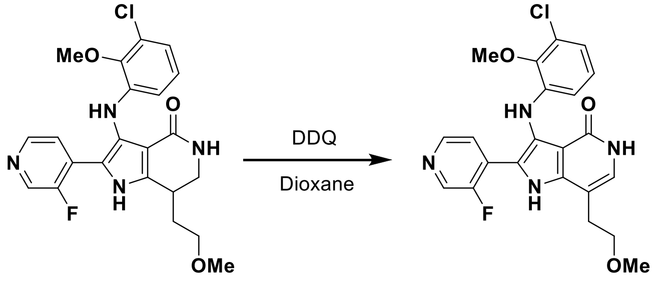

Compound 26

To a stirred solution of racemic 3-[(3-chloro-2-methoxyphenyl) amino]-2-(3-fluoropyridin-4-yl)-7-(2-methoxyethyl)-1*H*,5*H*,6*H*,7*H*-pyrrolo[3,2-c] pyridin-4-one (70 mg, 0.141 mmol) in 1,4-dioxane (1.0 mL) was added DDQ (38.5 mg, 0.169 mmol) in portions at RT. The resulting mixture was stirred for 6 h at 50 °C. The resultant mixture was allowed to cool to RT and was concentrated under reduced pressure. The residue was initially purified by Prep-TLC (DCM/MeOH = 15:1) the repurified by

reverse-phase flash chromatography (Column: YMC-Actus Triart C18, 30 × 150 mm, 5 μm; Mobile Phase A: Water (10 mmol/L NH_4HCO_3+0.1%NH_3·H_2O), Mobile Phase B: MeCN; Flow rate: 60 mL/min; Gradient: 30% B to 60% B in 8 min; Rt: 7.48 min) to afford 3-[(3-chloro-2-methoxyphenyl)amino]-2-(3-fluoropyridin-4-yl)-7-(2-methoxyethyl)-1*H*,5*H*-pyrrolo[3,2-c]pyridin-4-one (3.70 mg, 5.9% yield) as a yellow solid. ^{1}H NMR (400 MHz, DMSO-d_6) δ = 11.54 (s, 1H), 10.74 (d, *J* = 5.9 Hz, 1H), 8.57 (d, *J* = 2.6 Hz, 1H), 8.41 (d, *J* = 5.0 Hz, 1H), 7.61 (dd, *J* = 6.6, 5.0 Hz, 1H), 7.55 (s, 1H), 6.90 (d, *J* = 5.9 Hz, 1H), 6.71–6.60 (m, 2H), 6.14 (dd, *J* = 6.4, 3.2 Hz, 1H), 3.86 (s, 3H), 3.57 (t, *J* = 6.6 Hz, 2H), 3.30 (s, 3H), 2.87 (t, *J* = 6.5 Hz, 2H). *m/z* (ESI, +ve ion): 442.95 $(M + H)^+$.

Compounds 27 and S2

(7*S*)-3-[(3-Chloro-2-methoxyphenyl)amino]-2-(3-fluoropyridin-4-yl)-7-(2-methoxyethyl)-7-methyl-1*H*,5*H*,6*H*-pyrrolo[3,2-c]pyridin-4-one (Compound **27**) and **(7*R*)-3-[(3-chloro-2-methoxyphenyl)amino]-2-(3-fluoropyridin-4-yl)-7-(2-methoxyethyl)-7-methyl-1*H*,5*H*,6*H*-pyrrolo[3,2-c]pyridin-4-one** (Compound **S2**) were prepared according to the procedure described for Compounds **25** and **24**, using *tert*-butyl 5-methyl-2,4-dioxopiperidine-1-carboxylate as the nucleophile, LDA as the base, and 2-bromoethyl methyl ether as the electrophile in Step 1b at −70 °C for 1 h (50% yield), Step 2b-4b (59% yield over 3 steps), and Step 5b afforded the racemate of the title compounds (275 mg, 54% yield) as a light yellow solid after Prep-TLC (DCM:MeOH = 20:1). Chiral separation was then conducted via Prep-Chiral-HPLC (Column: CHIRALPAK ID, 2 × 25 cm, 5 μm; Mobile Phase A: Hex:DCM = 3:1 (0.5% 2 M NH_3-MeOH), Mobile Phase B: IPA; Flow rate: 20 mL/min; 30% B in 13 min) to afford (7*R*)-3-[(3-chloro-2-methoxyphenyl)amino]-2-(3-fluoropyridin-4-yl)-7-(2-methoxyethyl)-7-methyl-1*H*,5*H*,6*H*-pyrrolo[3,2-c]pyridin-4-one (Rt = 7.62 min, peak 1, 117 mg, 39% yield) as the first-eluting peak as an off-white solid and (7*S*)-3-[(3-chloro-2-methoxyphenyl)amino]-2-(3-fluoropyridin-4-yl)-7-(2-methoxyethyl)-7-methyl-1*H*,5*H*,6*H*-pyrrolo[3,2-c]pyridin-4-one (Rt = 11.27 min, peak 2, 112 mg, 37% yield) as the second-eluting peak as an off-white solid. ^{1}H NMR (400 MHz, DMSO-d_6) δ = 11.37 (s, 1H), 8.50 (d, *J* = 2.8 Hz, 1H), 8.32 (dd, *J* = 5.0, 1.1 Hz, 1H), 7.65 (s, 1H), 7.45 (dd, *J* = 6.8, 5.1 Hz, 1H), 7.28 (d, *J* = 2.7 Hz, 1H), 6.70–6.59 (m, 2H), 6.11 (dd, *J* = 7.6, 2.1 Hz, 1H), 3.85 (s, 3H), 3.43 (td, *J* = 6.7, 2.8 Hz, 2H), 3.29–3.24 (m, 4H), 3.17 (dd, *J* = 12.5, 2.4 Hz, 1H), 1.99 (dt, *J* = 14.0, 7.0 Hz, 1H), 1.84 (dt, *J* = 13.6, 6.5 Hz, 1H), 1.33 (s, 3H). *m/z* (ESI, +ve ion): 459.00 $(M + H)^+$. Absolute stereochemistry (*R* vs. *S*) was assigned based on biological activity analogy to Compound **23** and **22** whose stereochemistry is assigned crystallographically.

Compounds S3 and 28, S4, and S5

(7*S*)-3-[(3-chloro-2-methoxyphenyl)amino]-2-(3-fluoropyridin-4-yl)-7-[(2*S*)-oxetan-2-ylmethyl]-1*H*,5*H*,6*H*,7*H*-pyrrolo[3,2-c]pyridin-4-one (Compound **S3**), (7*S*)-3-[(3-chloro-2-methoxyphenyl)amino]-2-(3-fluoropyridin-4-yl)-7-[(2*R*)-oxetan-2-ylmethyl]-1*H*,5*H*,6*H*,7*H*-pyrrolo[3,2-c]pyridin-4-one (Compound **28**), (7*R*)-3-[(3-chloro-2-methoxyphenyl)amino]-2-(3-fluoropyridin-4-yl)-7-[(2*S*)-oxetan-2-ylmethyl]-1*H*,5*H*,6*H*,7*H*-pyrrolo[3,2-c]pyridin-4-one (Compound **S4**), and (7*R*)-3-[(3-chloro-2-methoxyphenyl)amino]-2-(3-fluoropyridin-4-yl)-7-[(2*R*)-oxetan-2-ylmethyl]-1*H*,5*H*,6*H*,7*H*-pyrrolo[3,2-c]pyridin-4-one (Compound **S5**).

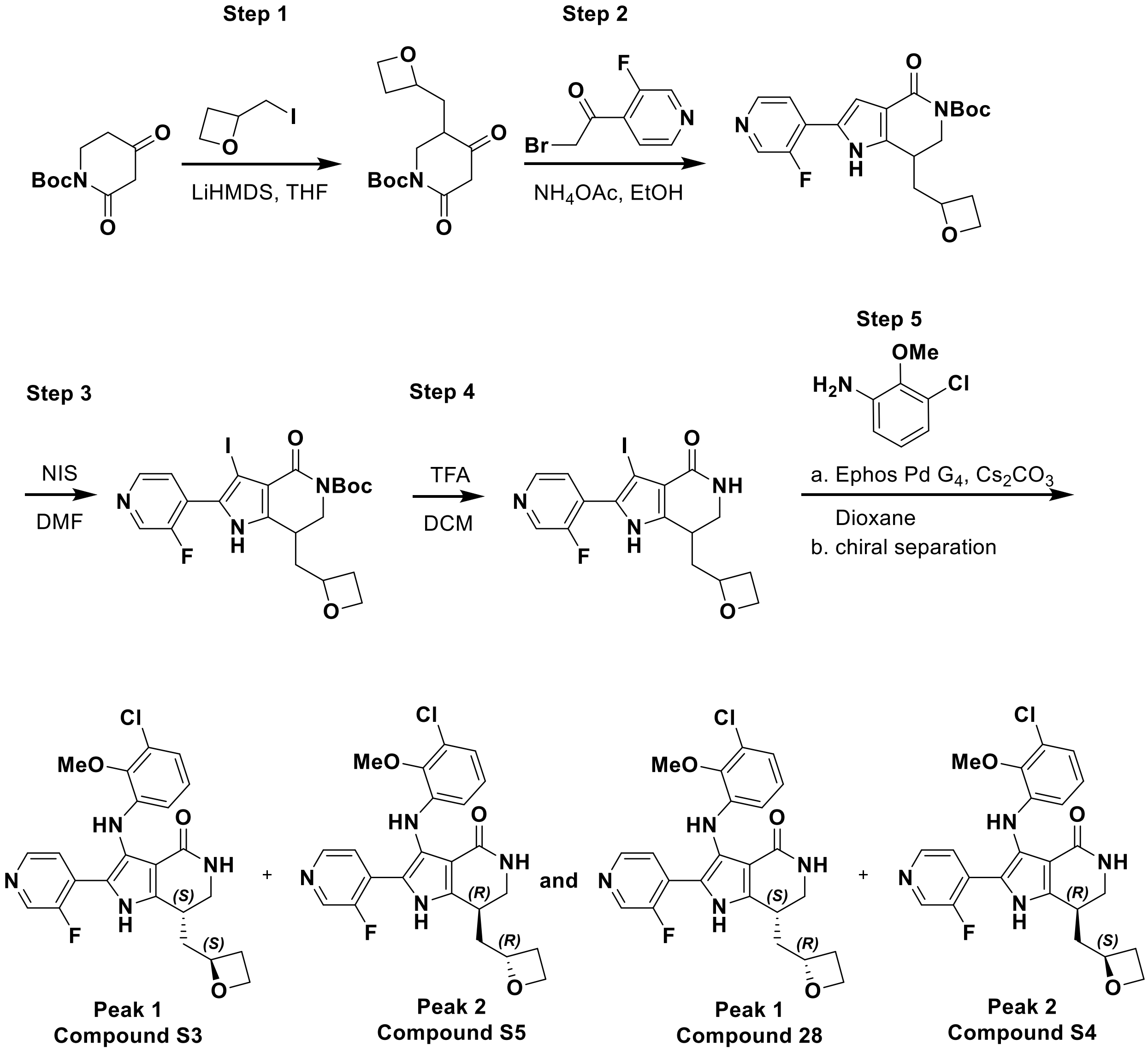

Step 1: *tert*-Butyl 5-(oxetan-2-ylmethyl)-2,4-dioxopiperidine-1-carboxylate. To a stirred solution of *tert*-butyl 2,4-dioxopiperidine-1-carboxylate (3.59 g, 16.8 mmol) in THF (50 mL) was added LiHMDS (42.1 mL, 42.1 mmol, 1 M in THF) dropwise at -20 degrees C under nitrogen atmosphere. To the above mixture was added 2-(iodomethyl)oxetane (5.00 g, 25.254 mmol, 1.5 equiv) dropwise at −20 °C. The resulting mixture was stirred for additional 3 h at −20 °C. The mixture was acidified to pH 5 with 5% $KHSO_4$ (aq.). The resulting mixture was extracted with EtOAc (3 × 100 mL). The combined organic layers were washed with brine (2 × 100 mL), dried over anhydrous Na_2SO_4, filtered, and concentrated under reduced pressure. The residue was purified by silica gel column chromatography, eluted with PE / EtOAc (2:1) to afford *tert*-butyl 5-(oxetan-2-ylmethyl)-2,4-dioxopiperidine-1-carboxylate (2.50 g, 52% yield) as a light yellow solid. *m/z* (ESI, +ve ion): 284.00 $(M + H)^+$.

Step 2: *tert*-Butyl 2-(3-fluoropyridin-4-yl)-7-(oxetan-2-ylmethyl)-4-oxo-1*H*,6*H*,7*H*-pyrrolo[3,2-c]pyridine-5-carboxylate. A solution of *tert*-butyl 5-(oxetan-2-ylmethyl)-2,4-dioxopiperidine-1-carboxylate (2.00 g, 7.06 mmol) in EtOH (30 mL) was treated with 2-bromo-1-(3-fluoropyridin-4-yl)ethanone (1.85 g, 8.47 mmol) for 2 min at RT under a N_2 atmosphere followed by the addition of NH_4OAc (3.26 g, 42.4 mmol) in portions at 60 °C. The residue was purified by reverse-phase flash chromatography (column: silica

gel; mobile phase, MeCN in water, 10% to 50% gradient in 10 min; detector, UV 220 nm) to afford *tert*-butyl 2-(3-fluoropyridin-4-yl)-7-(oxetan-2-ylmethyl)-4-oxo-1*H*,6*H*,7*H*-pyrrolo[3,2-c]pyridine-5-carboxylate (1.00 g, 35% yield) as a yellow oil. *m/z* (ESI, +ve ion): 402.30 $(M + H)^+$.

Step 3: *tert*-Butyl 2-(3-fluoropyridin-4-yl)-3-iodo-7-(oxetan-2-ylmethyl)-4-oxo-1*H*,6*H*,7*H*-pyrrolo[3,2-c]pyridine-5-carboxylate. A solution of *tert*-butyl 2-(3-fluoropyridin-4-yl)-7-(oxetan-2-ylmethyl)-4-oxo-1*H*,6*H*,7*H*-pyrrolo[3,2-c]pyridine-5-carboxylate (1.00 g, 2.49 mmol) in DMF (15 mL) was treated with NIS (0.56 g, 2.49 mmol) for 30 min at 0 °C under a N_2 atmosphere. The resulting mixture was stirred for 3 h at RT and the resulting mixture was extracted with EtOAc (3 × 50 mL). The combined organic layers were washed with brine (2 × 30 mL), dried over anhydrous Na_2SO_4, filtered, and concentrated reduced pressure. The residue was purified by silica gel column chromatography, eluted with DCM / MeOH (12:1) to afford *tert*-butyl 2-(3-fluoropyridin-4-yl)-3-iodo-7-(oxetan-2-ylmethyl)-4-oxo-1*H*,6*H*,7*H*-pyrrolo[3,2-c]pyridine-5-carboxylate (900 mg, 69% yield) as a yellow solid. *m/z* (ESI, +ve ion): 527.95 $(M + H)^+$.

Step 4: 2-(3-Fluoropyridin-4-yl)-3-iodo-7-(oxetan-2-ylmethyl)-1*H*,5*H*,6*H*,7*H*-pyrrolo[3,2-c]pyridin-4-one. A solution of *tert*-butyl 2-(3-fluoropyridin-4-yl)-3-iodo-7-(oxetan-2-ylmethyl)-4-oxo-1*H*,6*H*,7*H*-pyrrolo[3,2-c]pyridine-5-carboxylate (900 mg, 1.71 mmol) in DCM (7 mL) was treated with TFA (6 mL) at 0 °C under a N_2 atmosphere. followed by the addition of TFA (3 mL) dropwise at 0 degrees C. The resulting mixture was stirred for 2 h at RT and was then extracted with DCM (3 × 50 mL). The combined organic layers were washed with brine (2 × 30 mL), dried over anhydrous Na_2SO_4, filtered, and concentrated under reduced pressure. The residue was purified by silica gel column chromatography, eluted with DCM / MeOH (12:1) to afford 2-(3-fluoropyridin-4-yl)-3-iodo-7-(oxetan-2-ylmethyl)-1*H*,5*H*,6*H*,7*H*-pyrrolo[3,2-c]pyridin-4-one (580 mg, 80% yield) as a yellow solid. *m/z* (ESI, +ve ion): 428.00 $(M + H)^+$.

Step 5: (7*S*)-3-[(3-chloro-2-methoxyphenyl)amino]-2-(3-fluoropyridin-4-yl)-7-[(2*S*)-oxetan-2-ylmethyl]-1*H*,5*H*,6*H*,7*H*-pyrrolo[3,2-c]pyridin-4-one (Compound S3), (7*S*)-3-[(3-chloro-2-methoxyphenyl)amino]-2-(3-fluoropyridin-4-yl)-7-[(2*R*)-oxetan-2-ylmethyl]-1*H*,5*H*,6*H*,7*H*-pyrrolo[3,2-c]pyridin-4-one (Compound 28), (7*R*)-3-[(3-chloro-2-methoxyphenyl)amino]-2-(3-fluoropyridin-4-yl)-7-[(2*S*)-oxetan-2-ylmethyl]-1*H*,5*H*,6*H*,7*H*-pyrrolo[3,2-c]pyridin-4-one (Compound S4), and (7*R*)-3-[(3-chloro-2-methoxyphenyl)amino]-2-(3-fluoropyridin-4-yl)-7-[(2*R*)-oxetan-2-ylmethyl]-1*H*,5*H*,6*H*,7*H*-pyrrolo[3,2-c]pyridin-4-one (Compound S5). To a stirred mixture of 2-(3-fluoropyridin-4-yl)-3-iodo-7-(oxetan-2-ylmethyl)-1*H*,5*H*,6*H*,7*H*-pyrrolo[3,2-c]pyridin-4-one (405 mg, 0.948 mmol) and 3-chloro-2-methoxyaniline (374 mg, 2.37 mmol) in 1,4-dioxane (10 mL) were added Ephos Pd G4 (87.1 mg, 0.095 mmol) and Ephos (101 mg, 0.190 mmol) and Cs_2CO_3 (618 mg, 1.90 mmol) in portions at RT under a N_2 atmosphere. The resulting mixture was stirred for 1 h at 50 °C. The reaction mixture was then cooled to RT and concentrated under reduced pressure. The residue was purified by silica gel column chromatography, eluted with DCM / MeOH (10:1) to afford racemic 3-((3-chloro-2-methoxyphenyl)amino)-2-(3-fluoropyridin-4-yl)-7-(oxetan-2-ylmethyl)-1,5,6,7-tetrahydro-4*H*-pyrrolo[3,2-c]pyridin-4-one (300 mg, 69% yield) as a yellow solid. 300 mg of the racemate was purified by Prep-HPLC (Column: Xselect CSH C18 OBD Column 30 × 150 mm, 5 μmn; Mobile Phase A: Water (0.1% Formic Acid), Mobile Phase B: MeCN; Flow rate: 60 mL/min; Gradient: 29% B to 45% B in 10 min) to afford peak 1 (Rt: 8.0 min, 100 mg) and peak 2 (Rt = 9.0 min, 100 mg) as mixtures of two isomers. 100 mg of peak 1 was further purified by Prep-Chiral-HPLC (Column: CHIRAL ART Amylose-SA, 2 × 25 cm, 5 μm; Mobile Phase A: MBTE (0.5% 2 M NH_3-MeOH), Mobile Phase B: EtOH; Flow rate: 20 mL/min; 30% B in 18 min) to afford (7*S*)-3-[(3-chloro-2-methoxyphenyl)amino]-2-(3-fluoropyridin-4-yl)-7-[(2*S*)-oxetan-2-ylmethyl]-1*H*,5*H*,6*H*,7*H*-pyrrolo[3,2-c]pyridin-4-one (Compound **S3**) (Rt = 7.11 min, peak 1, 5.6 mg, 5.2% yield) as the first-eluting peak. ^1H NMR (400 MHz, DMSO-d_6) δ = 11.56 (s, 1H), 8.52 (d, *J* = 3.1 Hz, 1H), 8.29 (d, *J* = 5.2 Hz, 1H), 7.62 (s, 1H), 7.44 (dd, *J* = 6.8, 5.1 Hz, 1H), 7.24 (d, *J* = 2.5 Hz, 1H), 6.74–6.63 (m, 2H), 6.14 (dd, *J* = 7.2, 2.4 Hz, 1H), 5.02–4.91 (m, 1H), 4.62 (td, *J* = 7.9, 5.8 Hz, 1H), 4.52 (dt, *J* = 9.1, 5.8 Hz, 1H), 3.87 (s, 3H), 3.48–3.39 (m, 1H), 3.27–3.07 (m, 2H), 2.81–2.70 (m, 1H), 2.44–2.33 (m, 1H), 2.23 (m, 1H), 1.91 (m, 1H). *m/z* (ESI, +ve ion): 457.00 $(M + H)^+$. (7*R*)-3-[(3-Chloro-2-methoxyphenyl)amino]-2-(3-fluoropyridin-4-yl)-7-[(2*R*)-oxetan-2-ylmethyl]-1H,5H,6H,7H-pyrrolo[3,2-c]pyridin-4-one (Compound **S5**) (Rt = 13.35 min, peak 2, 6.1 mg, 6.1% yield) was isolated as the second-eluting peak. ^1H NMR (400 MHz, DMSO-d_6) δ = 11.56 (s, 1H), 8.52 (d, *J* = 3.0 Hz, 1H), 8.29 (d, *J* = 5.2 Hz, 1H), 7.62 (s, 1H), 7.44 (dd, *J* = 6.9, 5.1 Hz, 1H), 7.24 (d, *J* = 2.6 Hz, 1H), 6.74–6.63 (m, 2H), 6.14 (dd, *J* = 7.2, 2.4 Hz, 1H), 4.96 (m, 1H), 4.62 (td, *J* = 8.0, 5.8 Hz, 1H), 4.52 (dt, *J* = 9.0, 5.8 Hz, 1H), 3.88 (s, 3H), 3.44 (m, 1H), 3.27–3.07 (m, 2H), 2.81–2.69 (m, 1H), 2.44–2.33 (m, 1H), 2.23 (m, 1H), 1.91 (m, 1H). *m/z* (ESI, +ve ion): 457.00 $(M + H)^+$. 100 mg of peak 2 from Prep-HPLC was further purified by Prep-Chiral-HPLC (Column: CHIRAL ART Amylose-SA, 2 × 25 cm, 5 μm; Mobile Phase A: MBTE (0.5% 2 M NH_3-MeOH), Mobile Phase B: EtOH; Flow rate: 20 mL/min; 30% B in 18 min) to afford (7*S*)-3-[(3-chloro-

2-methoxyphenyl)amino]-2-(3-fluoropyridin-4-yl)-7-[(2*R*)-oxetan-2-ylmethyl]-1*H*,5*H*,6*H*,7*H*-pyrrolo[3,2-c]pyridin-4-one (Compound **28**) (Rt = 6.87 min, peak 1, 23.7 mg, 23.7% yield) as the first-eluting peak. ^{1}H NMR (400 MHz, DMSO-d_6) δ = 11.55 (s, 1H), 8.51 (d, *J* = 2.9 Hz, 1H), 8.30 (dd, *J* = 5.1, 1.1 Hz, 1H), 7.60 (s, 1H), 7.45 (dd, *J* = 6.8, 5.1 Hz, 1H), 7.22 (s, 1H), 6.72–6.61 (m, 2H), 6.13 (dd, *J* = 7.3, 2.4 Hz, 1H), 4.93 (p, *J* = 6.8 Hz, 1H), 4.55 (td, *J* = 8.0, 5.7 Hz, 1H), 4.42 (dt, *J* = 9.0, 5.7 Hz, 1H), 3.86 (s, 3H), 3.50 (m, 1H), 3.30–3.20 (m, 1H), 3.05 (m, 1H), 2.68–2.58 (m, 1H), 2.33–2.24 (m, 1H), 2.16–1.97 (m, 2H). *m/z* (ESI, +ve ion): 457.00 (M + H)$^+$. (7*R*)-3-[(3-Chloro-2-methoxyphenyl)amino]-2-(3-fluoropyridin-4-yl)-7-[(2*S*)-oxetan-2-ylmethyl]-1H,5H,6H,7H-pyrrolo[3,2-c]pyridin-4-one (Compound **S4**) (Rt = 15.10 min, peak 2, 20.7 mg, 20.7% yield) was isolated as the second-eluting peak. ^{1}H NMR (400 MHz, DMSO-d_6) δ = 11.55 (s, 1H), 8.51 (d, *J* = 2.9 Hz, 1H), 8.30 (d, *J* = 5.1 Hz, 1H), 7.60 (s, 1H), 7.45 (dd, *J* = 6.8, 5.1 Hz, 1H), 7.22 (d, *J* = 2.7 Hz, 1H), 6.72–6.61 (m, 2H), 6.13 (dd, *J* = 7.3, 2.4 Hz, 1H), 4.93 (p, *J* = 6.7 Hz, 1H), 4.55 (td, *J* = 8.0, 5.8 Hz, 1H), 4.42 (dt, *J* = 9.1, 5.7 Hz, 1H), 3.86 (s, 3H), 3.50 (ddd, *J* = 12.7, 5.3, 1.9 Hz, 1H), 3.25 (ddd, *J* = 12.6, 5.6, 3.5 Hz, 1H), 3.05 (dd, *J* = 7.7, 5.2 Hz, 1H), 2.68–2.58 (m, 1H), 2.30 (dd, *J* = 13.5, 6.2 Hz, 1H), 2.07 (ddt, *J* = 26.5, 13.9, 6.4 Hz, 2H). *m/z* (ESI, +ve ion): 457.00 (M + H)$^+$. Absolute stereochemistry of the lactam stereocenter (*R* vs. *S*) was assigned based on biological activity analogy to Compound **23** and **22** whose stereochemistry is assigned crystallographically. Absolute stereochemistry of the oxetane stereocenter was arbitrarily assigned.

Compounds 29 and S6

(*S*)-7-(((*S*)-1,4-Dioxan-2-yl)methyl)-3-((3-chloro-2-methoxyphenyl)amino)-2-(3-fluoropyridin-4-yl)-1,5,6,7-tetrahydro-4*H*-pyrrolo[3,2-c]pyridin-4-one (Compound **29**) and (*R*)-7-(((*S*)-1,4-dioxan-2-yl)methyl)-3-((3-chloro-2-methoxyphenyl)amino)-2-(3-fluoropyridin-4-yl)-1,5,6,7-tetrahydro-4*H*-pyrrolo[3,2-c]pyridin-4-one (Compound **S6**)

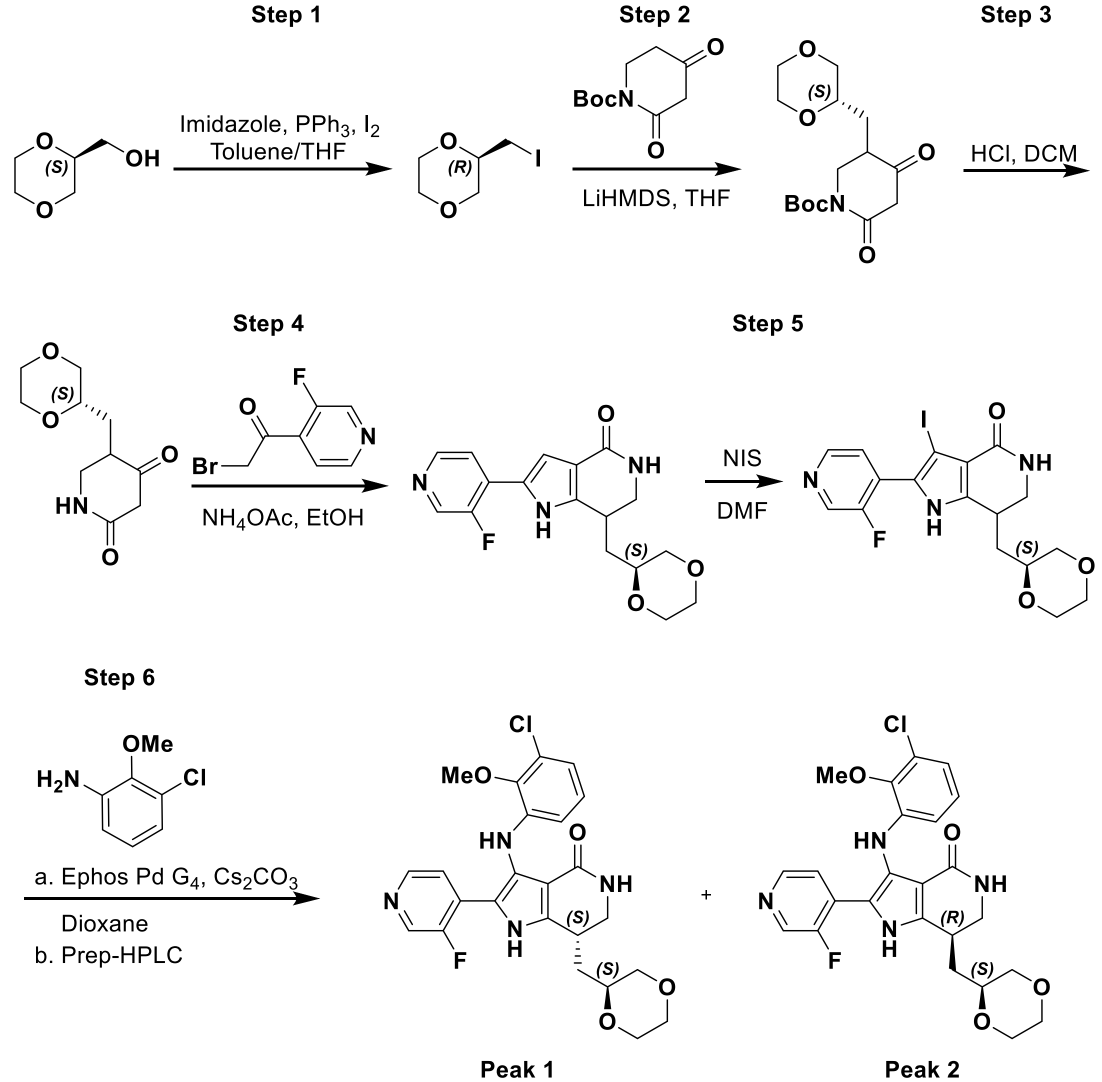

Step 1: (2*R*)-2-(Iodomethyl)-1,4-dioxane. To a stirred mixture of (2*S*)-1,4-dioxan-2-ylmethanol (100 g, 847 mmol) and imidazole (115 g, 1.69 mol) in toluene (100 mL) was added I_2 (215 g, 847 mmol) and PPh_3 (222 g, 847 mmol) in THF (50 mL) at 0 °C under a N_2 atmosphere. The resulting mixture was stirred for 3 h at RT before the reaction was quenched by the addition of sat. aq. Na_2SO_4 (100 mL). The precipitated solids were collected by filtration and washed with PE (2 × 100 mL) and the solids were purified by silica gel column chromatography, eluted with PE / EA (8:1) to afford (2*R*)-2-(iodomethyl)-1,4-dioxane (180 g, 89% yield) as a colorless oil.

Step 2: *tert*-Butyl 5-(((*S*)-1,4-dioxan-2-yl)methyl)-2,4-dioxopiperidine-1-carboxylate. To a stirred mixture of *tert*-butyl 2,4-dioxopiperidine-1-carboxylate (125 g, 586 mmol) and (2*R*)-2-(iodomethyl)-1,4-dioxane (174 g, 762 mmol) in THF (2.00 L) was added LiHMDS (1.76 L, 1 M in THF) dropwise at −70 °C under a N_2 atmosphere. The resulting mixture was stirred for 1 h at 0 °C and then acidified to pH 2 with HCl (aq.). The aqueous layer was extracted with EtOAc (2 × 250 mL), dried over anhydrous sodium sulfate, filtered and concentrated under reduced pressure. The residue was purified by silica gel column chromatography, eluted with PE / EA (3:1) to afford *tert*-butyl 5-(((*R*)-1,4-dioxan-2-yl)methyl)-2,4-dioxopiperidine-1-carboxylate (76.0 g, 41% yield) as a yellow green oil. *m/z* (ESI, +ve ion): 314.05 $(M + H)^+$.

Step 3: 5-(((*R*)-1,4-Dioxan-2-yl)methyl)piperidine-2,4-dione. To a stirred solution of *tert*-butyl 5-(((*R*)-1,4-dioxan-2-yl)methyl)-2,4-dioxopiperidine-1-carboxylate (70 g, 223 mmol) in DCM (1.5 L) was added HCl (300 mL, 300 mmol, 1 M in 1,4-dioxane) at RT under a N_2 atmosphere. The resultant mixture stirred at RT for 2 h and was then concentrated under reduced pressure to afford 5-(((*R*)-1,4-dioxan-2-yl)methyl)piperidine-2,4-dione (40 g, 84% yield) as a yellow oil that was used without further purification. *m/z* (ESI, +ve ion): 210.0 $(M + H)^+$.

Step 4: 7-(((*S*)-1,4-Dioxan-2-yl)methyl)-2-(3-fluoropyridin-4-yl)-1,5,6,7-tetrahydro-4*H*-pyrrolo[3,2-c]pyridin-4-one. To a stirred solution of 5-(((*R*)-1,4-dioxan-2-yl)methyl)piperidine-2,4-dione (55.0 g, 258 mmol) and NH_4OAc (99.4 g, 1.29 mol) in EtOH (1.45 L) were added 2-bromo-1-(3-fluoropyridin-4-yl)ethanone (56.2 g, 258 mmol) at RT under a N_2 atmosphere. Then, the solution was stirred at 50 °C for 16 h. The resulting mixture was concentrated under reduced pressure and purified by silica gel column chromatography, eluted with DCM / MeOH (12:1) to afford 7-(((*S*)-1,4-dioxan-2-yl)methyl)-2-(3-fluoropyridin-4-yl)-1,5,6,7-tetrahydro-4*H*-pyrrolo[3,2-c]pyridin-4-one (40.0 g, 46.8% yield) as a pink solid. *m/z* (ESI, +ve ion): 332.05 $(M + H)^+$.

Step 5: 7-(((*S*)-1,4-Dioxan-2-yl)methyl)-2-(3-fluoropyridin-4-yl)-3-iodo-1,5,6,7-tetrahydro-4*H*-pyrrolo[3,2-c]pyridin-4-one. To a stirred solution of 7-(((*S*)-1,4-dioxan-2-yl)methyl)-2-(3-fluoropyridin-4-yl)-1,5,6,7-tetrahydro-4*H*-pyrrolo[3,2-c]pyridin-4-one (40.0 g, 121 mmol) in DMF (1.50 L) was added NIS (32.6 g, 145 mmol) at 0 °C under a N_2 atmosphere. The resultant reaction mixture stirred for 1 h at RT. The resulting mixture was concentrated under reduced pressure and purified by silica gel column chromatography, eluted with DCM / MeOH (15:1) to afford to afford 7-(((*S*)-1,4-dioxan-2-yl)methyl)-2-(3-fluoropyridin-4-yl)-3-iodo-1,5,6,7-tetrahydro-4*H*-pyrrolo[3,2-c]pyridin-4-one (40.0 g, 72% yield) as a brown yellow solid. *m/z* (ESI, +ve ion): 457.90 $(M + H)^+$.

Step 6: (*S*)-7-(((*S*)-1,4-Dioxan-2-yl)methyl)-3-((3-chloro-2-methoxyphenyl)amino)-2-(3-fluoropyridin-4-yl)-1,5,6,7-tetrahydro-4*H*-pyrrolo[3,2-c]pyridin-4-one (Compound 29) and (*R*)-7-(((S)-1,4-dioxan-2-yl)methyl)-3-((3-chloro-2-methoxyphenyl)amino)-2-(3-fluoropyridin-4-yl)-1,5,6,7-tetrahydro-4*H*-pyrrolo[3,2-c]pyridin-4-one (Compound S6). To a stirred mixture of 7-(((*S*)-1,4-dioxan-2-yl)methyl)-2-(3-fluoropyridin-4-yl)-3-iodo-1,5,6,7-tetrahydro-4*H*-pyrrolo[3,2-c]pyridin-4-one (10.0 g, 21.9 mmol) and 3-chloro-2-methoxyaniline (5.17 g, 32.8 mmol) in DMF (100 mL) were added EPhos Pd G4 (2.01 g, 2.19 mmol), EPhos (2.34 g, 4.37 mmol), and Cs_2CO_3 (14.3 g, 43.7 mmol) in portions at RT under a N_2 atmosphere. The resulting mixture was stirred for 2 h at 50 °C, then cooled to RT, and extracted with EtOAc (2 × 500 mL). The combined organic layers were dried over anhydrous Na_2SO_4, filtered, and concentrated under reduced pressure. The residue was purified by silica gel column chromatography, eluted with DCM / MeOH (10:1) to afford 7-(((*S*)-1,4-dioxan-2-yl)methyl)-3-((3-chloro-2-methoxyphenyl)amino)-2-(3-fluoropyridin-4-yl)-1,5,6,7-tetrahydro-4*H*-pyrrolo[3,2-c]pyridin-4-one as a diastereomeric mixture (10 g, 93% yield) as a brown solid. Separation of diastereomers was then conducted via Prep-HPLC (Column: XBridge Prep OBD C18 Column, 30 × 150 mm, 5 μm; Mobile Phase A: Water (10 mmol/L NH_4HCO_3+0.1%NH_3-H2O), Mobile Phase B: MeCN; Flow rate: 60 mL/min; Gradient: 34% B to 49% B in 8 min) to afford (*S*)-7-(((*S*)-1,4-dioxan-2-yl)methyl)-3-((3-chloro-2-methoxyphenyl)amino)-2-(3-fluoropyridin-4-yl)-1,5,6,7-tetrahydro-4H-pyrrolo[3,2-c]pyridin-4-one (Rt = 6.13 min, peak 1, 1.50 g, 14% yield) as the first-eluting peak as a light yellow solid. ^{1}H NMR (400 MHz, DMSO-d_6) δ = 11.44 (s, 1H) 8.51 (d, *J* = 2.8 Hz, 1H), 8.30 (dd, *J* = 5.0, 1.1 Hz, 1H), 7.57 (s, 1H), 7.44 (dd, *J* = 6.8, 5.1 Hz, 1H), 7.18 (t, *J* = 2.6 Hz, 1H), 6.60–6.70 (m, 2H), 6.13 (dd, *J* = 7.1, 2.6 Hz, 1H), 3.85 (s, 3H), 3.76 (dd, *J* = 11.2, 2.8 Hz, 1H), 3.52–3.72 (m, 4H), 3.47 (td, *J* = 12.1, 2.9 Hz, 1H), 3.05–3.31 (m, 3H), 1.71–1.81 (m, 1H), 1.53–1.66 (m, 1H). *m/z* (ESI, +ve ion): 486.90 $(M + H)^+$. ((7*R*)-3-[(3-(*R*)-7-(((*S*)-1,4-dioxan-2-yl)methyl)-3-((3-chloro-2-methoxyphenyl)amino)-2-(3-fluoropyridin-4-yl)-1,5,6,7-tetrahydro-4H-pyrrolo[3,2-c]pyridin-4-one (Rt = 7.13 min, peak 2, 1.89 g, 19% yield) was isolated as the second-eluting peak as a yellow solid. ^{1}H NMR (400 MHz, DMSO-d_6) δ = 11.45 (s, 1H), 8.51 (d, *J* = 2.8 Hz, 1H), 8.30 (d, *J* = 5.1 Hz, 1H), 7.57 (s, 1H), 7.49–7.41 (m, 1H), 7.18 (s, 1H), 6.72–6.62 (m, 2H), 6.13 (dd, *J* = 7.1, 2.6 Hz, 1H), 3.81–3.66 (m, 3H), 3.66–3.33 (m, 7H), 3.30–3.09 (m, 3H), 1.77 (dd, *J* = 14.2, 5.3 Hz, 1H), 1.60 (dt, *J* = 15.0, 8.1 Hz, 1H). *m/z* (ESI, +ve ion): 486.95 $(M + H)^+$. Lactam absolute stereochemistry (*R* vs. *S*) was assigned based on biological activity analogy to Compound **32** and **S9** whose lactam stereochemistry is assigned crystallographically. Dioxane absolute stereochemistry (*R* vs. *S*) defined based on known starting material stereochemistry.

Compounds 30 and S7

((7*S*)-3-[(3-Chloro-2-methoxyphenyl)amino]-7-[(2*R*)-1,4-dioxan-2-ylmethyl]-2-(3-fluoropyridin-4-yl)-1*H*,5*H*,6*H*,7*H*-pyrrolo[3,2-c]pyridin-4-one (Compound **30**) and ((7*R*)-3-[(3-chloro-2-methoxyphenyl)amino]-7-[(2*R*)-1,4-dioxan-2-ylmethyl]-

2-(3-fluoropyridin-4-yl)-1*H*,5*H*,6*H*,7*H*-pyrrolo[3,2-c]pyridin-4-one (Compound **S7**) were prepared according to the procedure described for Compounds **29** and **S6**, using (2*R*)-1,4-dioxan-2-ylmethanol in Step 1 (67% yield), followed by Step 2 (46% yield), Step 3 and 4 (42% yield over 2 steps), Step 5 (80% yield), and Step 6 to afford 7-(((*R*)-1,4-dioxan-2-yl)methyl)-3-((3-chloro-2-methoxyphenyl)amino)-2-(3-fluoropyridin-4-yl)-1,5,6,7-tetrahydro-4*H*-pyrrolo[3,2-c]pyridin-4-one (4.50 g) as a brown solid after silica gel column chromatography, eluted with PE/EtOAc (1:3). Separation of diastereomers was then conducted via Prep-HPLC (Column: XBridge Prep OBD C18 Column, 30 × 150 mm, 5μm; Mobile Phase A: Water (10 mmol/L NH_4HCO_3), Mobile Phase B: MeCN; Flow rate: 60 mL/min; Gradient: 30% B to 60% B in 7 min) to afford ((7*R*)-3-[(3-chloro-2-methoxyphenyl)amino]-7-[(2*R*)-1,4-dioxan-2-ylmethyl]-2-(3-fluoropyridin-4-yl)-1*H*,5*H*,6*H*,7*H*-pyrrolo[3,2-c]pyridin-4-one (Compound **S7**) (Rt = 6.92 min, peak 1, 2.65 g, 15% yield) as the first-eluting peak as a light yellow solid. ^{1}H NMR (400 MHz, $CDCl_3$) δ = 10.20 (d, *J* = 7.8 Hz, 1H), 8.48 (d, *J* = 4.3 Hz, 1H), 8.17 (dd, *J* = 5.3, 1.2 Hz, 1H), 7.56 (s, 1H), 7.37 (dd, *J* = 7.3, 5.3 Hz, 1H), 6.78 (dd, *J* = 8.1, 1.5 Hz, 1H), 6.68 (t, *J* = 8.1 Hz, 1H), 6.23 (dd, *J* = 8.1, 1.5 Hz, 1H), 5.61 (s, 1H), 4.07 (s, 4H), 3.93–3.59 (m, 6H), 3.54–3.45 (m, 1H), 3.36–3.25 (m, 2H), 2.11–1.97 (m, 1H), 1.74-1.64 (m, 1H). *m/z* (ESI, +ve ion): 487.10 $(M + H)^+$. ((7*S*)-3-[(3-Chloro-2-methoxyphenyl)amino]-7-[(2*R*)-1,4-dioxan-2-ylmethyl]-2-(3-fluoropyridin-4-yl)-1*H*,5*H*,6*H*,7*H*-pyrrolo[3,2-c]pyridin-4-one (Compound **30**) (Rt = 7.85 min, peak 2, 3.16 g, 18% yield) was isolated as the second-eluting peak as a yellow solid. ^{1}H NMR (400 MHz, $CDCl_3$) δ = 11.01 (d, *J* = 6.6 Hz, 1H), 8.46 (d, *J* = 4.3 Hz, 1H), 8.14 (dd, *J* = 5.3, 1.2 Hz, 1H), 7.57 (s, 1H), 7.38 (dd, *J* = 7.3, 5.3 Hz, 1H), 6.77 (dd, *J* = 8.1, 1.5 Hz, 1H), 6.66 (t, *J* = 8.1 Hz, 1H), 6.22 (dd, *J* = 8.1, 1.5 Hz, 1H), 5.64 (d, *J* = 3.9 Hz, 1H), 4.11 (d, *J* = 11.6 Hz, 1H), 4.07 (s, 3H), 3.99–3.70 (m, 5H), 3.46–3.27 (m, 4H), 1.83–1.72 (m, 1H), 1.56–1.45 (m, 1H). *m/z* (ESI, +ve ion): 487.00 $(M + H)^+$. Lactam absolute stereochemistry (*R* vs. *S*) was assigned based on biological activity analogy to Compound **32** and **S9** whose lactam stereochemistry is assigned crystallographically. Dioxane absolute stereochemistry (*R* vs. *S*) defined based on known starting material stereochemistry.

Compounds 31 and S8

(7*S*)-3-[(3-Chloro-2-methoxyphenyl)amino]-7-[(2*R*)-1,4-dioxan-2-ylmethyl]-2-(pyrimidin-4-yl)- 1*H*,5*H*,6*H*,7*H* -pyrrolo[3,2-c]pyridin-4-one (Compound **31**) and (7*R*)-3-[(3-chloro-2-methoxyphenyl)amino]-7-[(2*R*)-1,4-dioxan-2-ylmethyl]-2-(pyrimidin-4-yl)- 1*H*,5*H*,6*H*,7*H* -pyrrolo[3,2-c]pyridin-4-one (Compound **S8**)

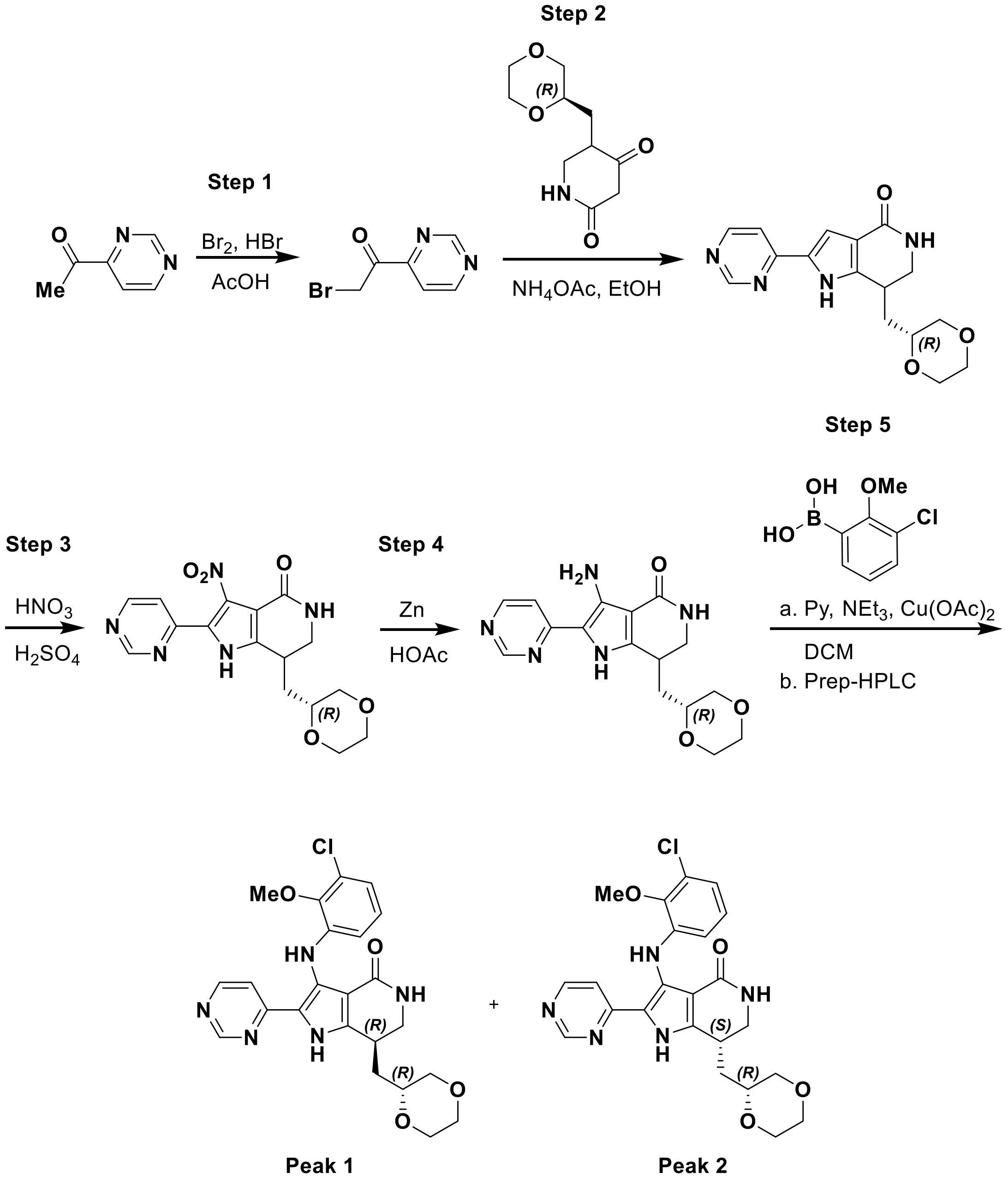

Peak 1 Compound S8 + Peak 2 Compound 31

Step 1: 2-Bromo-1-(pyrimidin-4-yl)ethenone. To a stirred solution of 1-(pyrimidin-4-yl)ethanone (1.00 g, 8.19 mmol) and HBr (1.45 mL, 49.6 mmol) in AcOH (3.0 mL) was added Br_2 (1.00 g, 6.26 mmol) dropwise at RT. The resultant mixture was allowed to stir at 60 °C. After 3 h, the resultant mixture was diluted with EtOAc (25 mL) and the mixture was stirred at RT. After 12 h, the solid precipitate was filtrated and dried under reduced pressure to afford 2-bromo-1-(pyrimidin-4-yl)ethenone (1.30 g, 79% yield) as a brown solid. *m/z* (ESI, +ve ion): 200.95 $(M + H)^+$.

Step 2: 7-[(2*R*)-1,4-Dioxan-2-ylmethyl]-2-(pyrimidin-4-yl)-1*H*,5*H*,6*H*,7*H*-pyrrolo[3,2-c]pyridin-4-one. A solution of 2-bromo-1-(pyrimidin-4-yl)ethanone (3 g, 10.6 mmol) in EtOH (50 mL) was treated with 5-[(2*R*)-1,4-dioxan-2-ylmethyl]piperidine-

2,4-dione (2.27 g, 10.6 mmol) at RT under a N_2 atmosphere. NH_4OAc (8.20 g, 106 mmol) was then introduced in portions at RT. The resulting mixture was stirred for 2 h at 60 °C. The resulting mixture was extracted with EtOAc (3 × 100 mL). The combined organic layers were dried over anhydrous Na_2SO_4, filtered, and concentrated under reduced pressure. The residue was purified by silica gel column chromatography, eluted with DCM / MeOH (10:1) to afford 7-[(2*R*)-1,4-dioxan-2-ylmethyl]-2-(pyrimidin-4-yl)-1*H*,5*H*,6*H*,7*H*-pyrrolo[3,2-c]pyridin-4-one (1.50 g, 45% yield) as a yellow solid. *m/z* (ESI, +ve ion): 315.10 $(M + H)^+$.

Step 3: 7-[(2*R*)-1,4-Dioxan-2-ylmethyl]-3-nitro-2-(pyrimidin-4-yl)-1*H*,5*H*,6*H*,7*H*-pyrrolo[3,2-c]pyridin-4-one. A solution of 7-[(2*R*)-1,4-dioxan-2-ylmethyl]-2-(pyrimidin-4-yl)-1*H*,5*H*,6*H*,7*H*-pyrrolo[3,2-c]pyridin-4-one (700 mg, 2.23 mmol) in H_2SO_4 (10 mL) was treated with HNO_3 (93.0 µL, 2.23 mmol) at −5 °C under a N_2 atmosphere. The resulting mixture was stirred for 1 h at 0 °C. The resulting mixture was diluted with water (40 mL). The mixture was basified to pH 9 with NaOH (aq.). The resulting mixture was extracted with DCM (3 × 30 mL). The combined organic layers were dried over anhydrous Na_2SO_4, filtered, and concentrated under reduced pressure to afford 7-[(2*R*)-1,4-dioxan-2-ylmethyl]-3-nitro-2-(pyrimidin-4-yl)-1*H*,5*H*,6*H*,7*H*-pyrrolo[3,2-c]pyridin-4-one (800 mg) as a brown solid that was used immediately in the next step without further purification. *m/z* (ESI, +ve ion): 360.25 $(M + H)^+$.

Step 4: 3-Amino-7-[(2*R*)-1,4-dioxan-2-ylmethyl]-2-(pyrimidin-4-yl)-1*H*,5*H*,6*H*,7*H*-pyrrolo[3,2-c]pyridin-4-one. A solution of 7-[(2*R*)-1,4-dioxan-2-ylmethyl]-3-nitro-2-(pyrimidin-4-yl)-1*H*,5*H*,6*H*,7*H*-pyrrolo[3,2-c]pyridin-4-one (800 mg, 2.23 mmol) in HOAc (10 mL) was treated with Zn (728 mg, 11.1 mmol) for 1 h at RT under a N_2 atmosphere. The resulting mixture was diluted with water (30 mL). The mixture was basified to pH 8 with NaOH (aq.). The resulting mixture was extracted with DCM (3 × 30 mL). The combined organic layers were dried over anhydrous Na_2SO_4, filtered, and concentrated under reduced pressure to afford 3-amino-7-[(2*R*)-1,4-dioxan-2-ylmethyl]-2-(pyrimidin-4-yl)-1*H*,5*H*,6*H*,7*H*-pyrrolo[3,2-c]pyridin-4-one (500 mg) as a yellow solid that was used immediately in the next step without further purification. *m/z* (ESI, +ve ion): 330.30 $(M + H)^+$.

Step 5: (7*S*)-3-[(3-Chloro-2-methoxyphenyl)amino]-7-[(2*R*)-1,4-dioxan-2-ylmethyl]-2-(pyrimidin-4-yl)- 1*H*,5*H*,6*H*,7*H* -pyrrolo[3,2-c]pyridin-4-one (Compound 31) and (7*R*)-3-[(3-chloro-2-methoxyphenyl)amino]-7-[(2*R*)-1,4-dioxan-2-ylmethyl]-2-(pyrimidin-4-yl)- 1*H*,5*H*,6*H*,7*H* -pyrrolo[3,2-c]pyridin-4-one (Compound S8). A solution of 3-amino-7-[(2*R*)-1,4-dioxan-2-ylmethyl]-2-(pyrimidin-4-yl)-1*H*,5*H*,6*H*,7*H*-pyrrolo[3,2-c]pyridin-4-one (500 mg, 1.52 mmol) and 3-chloro-2-methoxyphenylboronic acid (566 mg, 3.04 mmol) in DCM (10 mL) was treated with pyridine (245 µL, 3.04 mmol) and NEt_3 (423 µL, 3.036 mmol) at 0 °C under a N_2 atmosphere. $Cu(OAc)_2$ (276 mg, 1.52 mmol) was introduced in portions at RT. The resulting mixture was stirred for 2 h and the resulting mixture was extracted with DCM (3 × 50 mL). The combined organic layers were washed with brine (2 × 50 mL), dried over anhydrous Na_2SO_4, filtered, and concentrated under reduced pressure to 7-(((*R*)-1,4-dioxan-2-yl)methyl)-3-((3-chloro-2-methoxyphenyl)amino)-2-(pyrimidin-4-yl)-1,5,6,7-tetrahydro-4*H*-pyrrolo[3,2-c]pyridin-4-one (150 mg) as a yellow solid. *m/z* (ESI, +ve ion): 470.40 $(M + H)^+$. Separation of diastereomers was then conducted via Prep-HPLC (Column: Xselect CSH C18 OBD Column 30 × 150 mm, 5µm; Mobile Phase A: Water (0.05% TFA), Mobile Phase B: MeCN; Flow rate: 60 mL/min; Gradient: 20% B to 31% B in 11 min) to afford (7*R*)-3-[(3-chloro-2-methoxyphenyl)amino]-7-[(2*R*)-1,4-dioxan-2-ylmethyl]-2-(pyrimidin-4-yl)-1*H*,5*H*,6*H*,7*H*-pyrrolo[3,2-c]pyridin-4-one (Compound **S8**) (Rt = 9.00 min, peak 1, 7.6 mg, 0.7% yield over 3 steps) as the first-eluting peak as a yellow solid. ^1H NMR (400 MHz, DMSO-d_6) δ =12.00 (s, 1H), 9.05 (d, *J* = 1.4 Hz, 1H), 8.56 (d, *J* = 5.6 Hz, 1H), 7.96 (s, 1H), 7.20 (m, 2H), 6.86–6.76 (m, 2H), 6.31 (dd, *J* = 6.8, 2.8 Hz, 1H), 3.93 (s, 3H), 3.83–3.39 (m, 8H), 3.19 (dd, *J* = 11.3, 9.7 Hz, 1H), 3.09 (dd, *J* = 8.8, 4.6 Hz, 1H), 1.78 (dt, *J* = 14.1, 4.7 Hz, 1H), 1.67–1.55 (m, 1H). *m/z* (ESI, +ve ion): 470.35 $(M + H)^+$. (7*S*)-3-[(3-Chloro-2-methoxyphenyl)amino]-7-[(2*R*)-1,4-dioxan-2-ylmethyl]-2-(pyrimidin-4-yl)-1*H*,5*H*,6*H*,7*H*-pyrrolo[3,2-c]pyridin-4-one (Compound **31**) (Rt = 10.00 min, peak 1, 11.5 mg, 1.1% yield over 3 steps) was isolated as the second-eluting peak as a yellow solid. ^1H NMR (400 MHz, DMSO-d_6) δ = 12.02 (s, 1H), 9.03 (d, *J* = 1.4 Hz, 1H), 8.56 (d, *J* = 5.6 Hz, 1H), 8.06 (s, 1H), 7.26–7.16 (m, 2H), 6.87–6.76 (m, 2H), 6.34 (dd, *J* = 7.0, 2.7 Hz, 1H), 3.93 (s, 3H), 3.86–3.56 (m, 5H), 3.50 (td, *J* = 10.6, 10.1, 3.6 Hz, 2H), 3.29–3.14 (m, 3H), 1.84 (ddd, *J* = 14.3, 9.2, 5.3 Hz, 1H), 1.65–1.54 (m, 1H). *m/z* (ESI, +ve ion): 470.35 $(M + H)^+$. Lactam absolute stereochemistry (*R* vs. *S*) was assigned based on biological activity analogy to Compound **32** and **S9** whose lactam stereochemistry is assigned crystallographically. Dioxane absolute stereochemistry (*R* vs. *S*) defined based on known starting material stereochemistry.

Compounds 32 and S9

(7*S*)-3-[(3-Chloro-2-methoxyphenyl)amino]-7-[(2*R*)-1,4-dioxan-2-ylmethyl]-2-(2-methylpyrimidin-4-yl)- 1*H*,5*H*,6*H*,7*H*-pyrrolo[3,2-c]pyridin-4-one (Compound **32**) and (7*R*)-3-[(3-chloro-2-methoxyphenyl)amino]-7 [(2*R*)-1,4-dioxan-2-ylmethyl]-2-(2-methylpyrimidin-4-yl)-1*H*,5*H*,6*H*,7*H*-pyrrolo[3,2-c]pyridin-4-one (Compound **S9**).

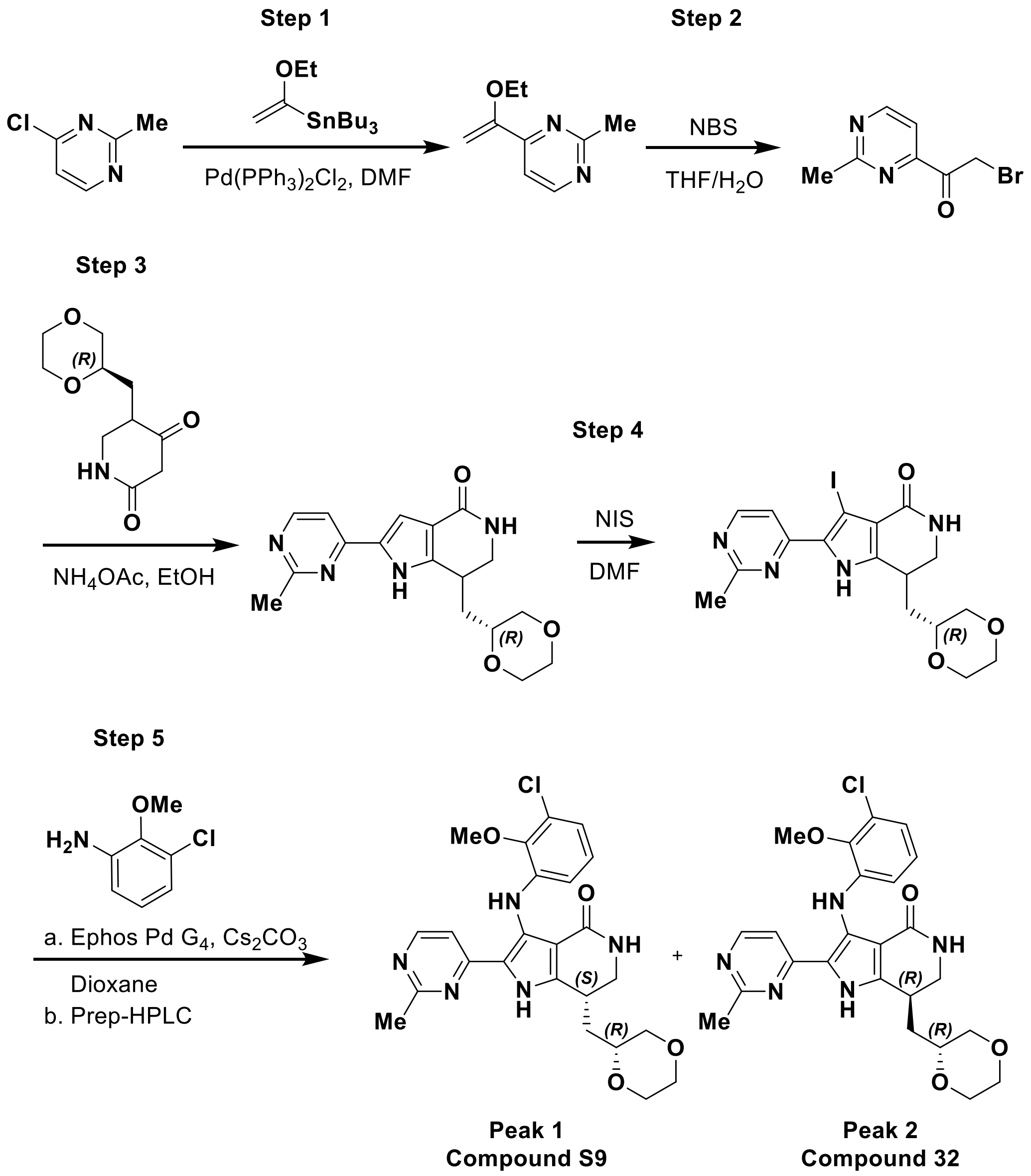

Step 1: 4-(1-Ethoxyethenyl)-2-methylpyrimidine. To a stirred mixture of 4-chloro-2-methylpyrimidine (10 g, 77.8 mmol) and tributyl(1-ethoxyethenyl)stannane (42.1 g, 117 mmol) in DMF (100 mL) was added $Pd(PPh_3)_2Cl_2$ (5.46 g, 7.78 mmol) at RT under a N_2 atmosphere. The resulting mixture was stirred for 12 h at 100 °C then cooled to RT before brine (1 L) was introduced. The aqueous layer was extracted with EtOAc (3 × 500 mL). The combined organic layers were dried over anhydrous Na_2SO_4, filtered, and

concentrated under reduced pressure. The residue was purified by silica gel column chromatography, eluted with PE / EA (10:1) to afford 4-(1-ethoxyethenyl)-2-methylpyrimidine (8.0 g, 63% yield) as a brown yellow liquid. *m/z* (ESI, +ve ion): 165.05 (M + H)$^+$.

Step 2: 2-Bromo-1-(2-methylpyrimidin-4-yl)ethenone. To a stirred mixture of 4-(1-ethoxyethenyl)-2-methylpyrimidine (4.00 g, 24.4 mmol) in THF (40 mL) and H_2O (4 mL) was added NBS (5.20 g, 29.2 mmol) in portions at RT under a N_2 atmosphere. The resulting mixture was stirred for 30 min at RT before brine (100 mL) was introduced. The aqueous layer was extracted with EtOAc (3 × 50 mL). The combined organic layers were dried over anhydrous Na_2SO_4, filtered, and concentrated under reduced pressure to afford 2-bromo-1-(2-methylpyrimidin-4-yl)ethanone (6.65 g) as a brown solid which was used in the next step directly without further purification. *m/z* (ESI, +ve ion): 215.95 (M + H)$^+$.

Step 3: 7-[(2*R*)-1,4-Dioxan-2-ylmethyl]-2-(2-methylpyrimidin-4-yl)-1*H*,5*H*,6*H*,7*H*-pyrrolo[3,2-c]pyridin-4-one. To a stirred mixture of 5-[(2*R*)-1,4-dioxan-2-ylmethyl]piperidine-2,4-dione (3.00 g, 14.1 mmol) and 2-bromo-1-(2-methylpyrimidin-4-yl)ethanone (4.54 g, 21.1 mmol) in EtOH (20 mL) was added NH_4OAc (10.8 g, 141 mmol) in portions at RT under a N_2 atmosphere. The resulting mixture was stirred for 30 min at 60 °C and was cooled to RT. Brine (50 mL) was introduced and the mixture was extracted with EtOAc (3 × 50 mL). The combined organic layers were dried over anhydrous Na_2SO_4, filtered, and concentrated under reduced pressure. The residue was purified by silica gel column chromatography, eluted with DCM / MeOH (20:1) to afford 7-[(2*R*)-1,4-dioxan-2-ylmethyl]-2-(2-methylpyrimidin-4-yl)-1*H*,5*H*,6*H*,7*H*-pyrrolo[3,2-c]pyridin-4-one (700 mg, 15% yield) as a yellow solid. *m/z* (ESI, +ve ion): 329.2 (M + H)$^+$.

Step 4: 7-[(2*R*)-1,4-dioxan-2-ylmethyl]-3-iodo-2-(2-methylpyrimidin-4-yl)-1*H*,5*H*,6*H*,7*H*-pyrrolo[3,2-c]pyridin-4-one. To a stirred solution of 7-[(2*R*)-1,4-dioxan-2-ylmethyl]-2-(2-methylpyrimidin-4-yl)-1*H*,5*H*,6*H*,7*H*-pyrrolo[3,2-c]pyridin-4-one (700 mg, 2.13 mmol) in DMF (10 mL) was added NIS (576 mg, 2.56 mmol) in portions at 0 °C under a N_2 atmosphere. The resulting mixture was stirred for 1 h at RT before brine (50 mL) was introduced. The aqueous layer was extracted with EtOAc (3 × 25 mL). The combined organic layers were dried over anhydrous Na_2SO_4, filtered, and concentrated under reduced pressure to afford 7-[(2*R*)-1,4-dioxan-2-ylmethyl]-3-iodo-2-(2-methylpyrimidin-4-yl)-1*H*,5*H*,6*H*,7*H*-pyrrolo[3,2-c]pyridin-4-one (700 mg) as a yellow oil which was used in the next step directly without further purification. *m/z* (ESI, +ve ion): 455.0 (M + H)$^+$.

Step 5: (7*S*)-3-[(3-Chloro-2-methoxyphenyl)amino]-7-[(2*R*)-1,4-dioxan-2-ylmethyl]-2-(2-methylpyrimidin-4-yl)-1*H*,5*H*,6*H*,7*H*-pyrrolo[3,2-c]pyridin-4-one (Compound 32) and (7*R*)-3-[(3-chloro-2-methoxyphenyl)amino]-7 [(2*R*)-1,4-dioxan-2-ylmethyl]-2-(2-methylpyrimidin-4-yl)-1*H*,5*H*,6*H*,7*H*-pyrrolo[3,2-c]pyridin-4-one (Compound S9). To a stirred mixture of 7-[(2*R*)-1,4-dioxan-2-ylmethyl]-3-iodo-2-(2-methylpyrimidin-4-yl)-1*H*,5*H*,6*H*,7*H*-pyrrolo[3,2-c]pyridin-4-one (700 mg, 1.54 mmol) and 3-chloro-2-methoxyaniline (729 mg, 4.62 mmol) in DMF (10 mL) was added Cs_2CO_3 (1.00 g, 3.08 mmol) and Ephos Pd G4 (283 mg, 0.308 mmol) in portions at RT under a N_2 atmosphere. The resulting mixture was stirred for 1 h at 60 °C then cooled to RT and concentrated under reduced pressure. The residue was purified by silica gel column chromatography, eluted with DCM / MeOH (20:1) to afford 7-(((*R*)-1,4-dioxan-2-yl)methyl)-3-((3-chloro-2-methoxyphenyl)amino)-2-(2-methylpyrimidin-4-yl)-1,5,6,7-tetrahydro-4*H*-pyrrolo[3,2-c]pyridin-4-one (325 mg, 44% yield) as a brown solid. Separation of diastereomers was then conducted via Prep-HPLC on 120 mg (0.248 mmol) (Column: CHIRALPAK IG, 2 × 25 cm, 5 μm; Mobile Phase A: Hex:DCM=3:1 (0.5% 2 M NH_3-MeOH), Mobile Phase B: IPA; Flow rate: 20 mL/min; Isocratic gradient: 20% B) to afford (7*R*)-3-[(3-chloro-2-methoxyphenyl)amino]-7-[(2*R*)-1,4-dioxan-2-ylmethyl]-2-(2-methylpyrimidin-4-yl)-1*H*,5*H*,6*H*,7*H*-pyrrolo[3,2-c]pyridin-4-one (Compound **S9**) (Rt = 7.92, peak 1, 24.8 mg, 21% yield) as the first-eluting peak as a yellow solid and (7*S*)-3-[(3-chloro-2-methoxyphenyl)amino]-7-[(2*R*)-1,4-dioxan-2-ylmethyl]-2-(2-methylpyrimidin-4-yl)-1*H*,5*H*,6*H*,7*H*-pyrrolo[3,2-c]pyridin-4-one (Compound **32**) (Rt = 9.81, peak 2, 18.2 mg, 15% yield) as the second-eluting peak as a yellow solid. ^{1}H NMR (400 MHz, DMSO-d_6) δ = 10.42 (s, 1H), 8.36 (d, *J* = 5.5 Hz, 1H), 7.56 (s, 1H), 6.85–6.70 (m, 3H), 6.32 (dd, *J* = 8.1, 1.5 Hz, 1H), 5.36 (s, 1H), 4.07 (s, 3H), 4.04 (d, *J* = 2.6 Hz, 1H), 3.91 (td, *J* = 11.4, 2.7 Hz, 1H), 3.79 (dd, *J* = 11.8, 2.6 Hz, 1H), 3.76–3.64 (m, 3H), 3.61 (d, *J* = 9.4 Hz, 1H), 3.44 (dd, *J* = 11.4, 9.9 Hz, 1H), 3.35–3.28 (m, 2H), 2.70 (s, 3H), 1.98 (td, *J* = 11.3, 9.4, 3.2 Hz, 1H), 1.75–1.64 (m, 1H). *m/z* (ESI, +ve ion): 484.25 (M + H)$^+$. Absolute stereochemistry of Compound **32** was assigned crystallographically.

Compound 33

3-[(3-Chloro-2-methoxyphenyl) amino]-2-(3-[2-[1-(difluoromethyl)cyclopropyl]ethynyl]pyridin-4-yl)- 1*H*,5*H*,6*H*,7*H*-pyrrolo[3,2-c]pyridin-4-one

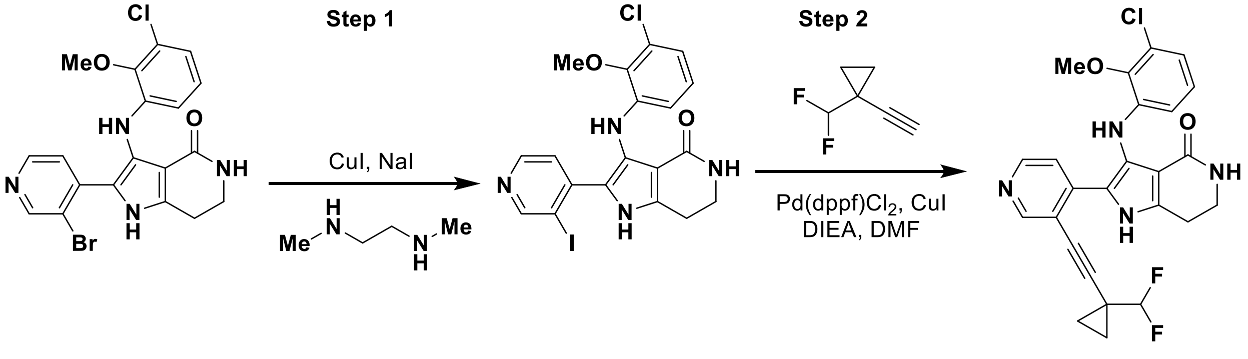

Compound 33

Step 1: 3-[(3-Chloro-2-methoxyphenyl)amino]-2-(3-iodopyridin-4-yl)-1*H*,5*H*,6*H*,7*H*-pyrrolo[3,2-c]pyridin-4-one. NaI (670 mg, 4.48 mmol), CuI (43.0 mg, 0.224 mmol), and methyl[2-(methylamino)ethyl]amine (98 mg, 1.12 mmol) were added to a solution of 2-(3-bromopyridin-4-yl)-3-[(3-chloro-2-methoxyphenyl)amino]-1*H*,5*H*,6*H*,7*H*-pyrrolo[3,2-c] pyridin-4-one (1.00 g, 2.24 mmol) in 1,4-dioxane (10 mL). The resulting mixture was stirred for 12 h at 110 °C under an argon atmosphere. The mixture was allowed to cool to RT, concentrated under reduced pressure, and purified by reverse-phase flash chromatography (Column: C18; mobile phase, MeCN in water, 20% to 60% gradient in 30 min; detector, UV 254 nm) to afford 3-[(3-chloro-2-methoxyphenyl)amino]-2-(3-iodopyridin-4-yl)-1*H*,5*H*,6*H*,7*H*-pyrrolo[3,2-c]pyridin-4-one (610 mg, 40% yield) as a yellow solid. *m/z* (ESI, +ve ion): 496.0 $(M + H)^+$.

Step 2: 3-[(3-Chloro-2-methoxyphenyl)amino]-2-(3-[2-[1-(difluoromethyl)cyclopropyl]ethynyl]pyridin-4-yl)-1*H*,5*H*,6*H*,7*H*-pyrrolo[3,2-c]pyridin-4-one (Compound 33). To a stirred mixture of 3-[(3-chloro-2-methoxyphenyl)amino]-2-(3-iodopyridin-4-yl)-1*H*,5*H*,6*H*,7*H*-pyrrolo[3,2-c] pyridin-4-one (300 mg, 0.61 mmol) and 1-(difluoromethyl)-1-ethynylcyclopropane (211 mg, 1.82 mmol) in DMF (3.0 mL) were added *N*,*N*-diisopropylethylamine (392 mg, 3.03 mmol), CuI (12 mg, 0.061 mmol) and Pd(dppf)Cl_2·CH_2Cl_2 (49 mg, 0.06 mmol) at RT under an argon atmosphere. The resulting mixture was stirred for 2 h at 50 °C. The mixture was allowed to cool to RT, concentrated under reduced pressure, and purified by reverse-phase flash chromatography (Column: C18 silica gel; mobile phase, MeCN in water (5 mM NH_4HCO_3), 30% to 70% gradient in 10 min; detector, UV 254 nm) to afford 3-[(3-chloro-2-methoxyphenyl)amino]-2-(3-[2-[1-(difluoromethyl)cyclopropyl]ethynyl]pyridin-4-yl)-1*H*,5*H*,6*H*,7*H*-pyrrolo[3,2-c]pyridin-4-one (142 mg, 47% yield) as a yellow solid. ^{1}H NMR (300 MHz, DMSO-d_6) δ = 11.40 (s, 1H), 8.55 (s, 1H), 8.36 (s, 1H), 7.31 (d, *J* = 7.9 Hz, 2H), 7.10 (d, *J* = 2.6 Hz, 1H), 6.65–6.59 (m, 2H), 6.11–5.99 (m, 1H), 6.02–5.58 (m, 1H), 3.83 (s, 3H), 3.45–3.35 (m, 2H), 2.82 (t, *J* = 6.8 Hz, 2H), 1.30–1.15 (m, 4H). *m/z* (ESI, +ve ion): 483.00 $(M + H)^+$.

Compound 34

3-[(3-Chloro-2-methoxyphenyl)amino]-2-[furo[3,2-b]pyridin-7-yl]-1*H*,5*H*,6*H*,7*H*-pyrrolo[3,2-c]pyridin-4-one

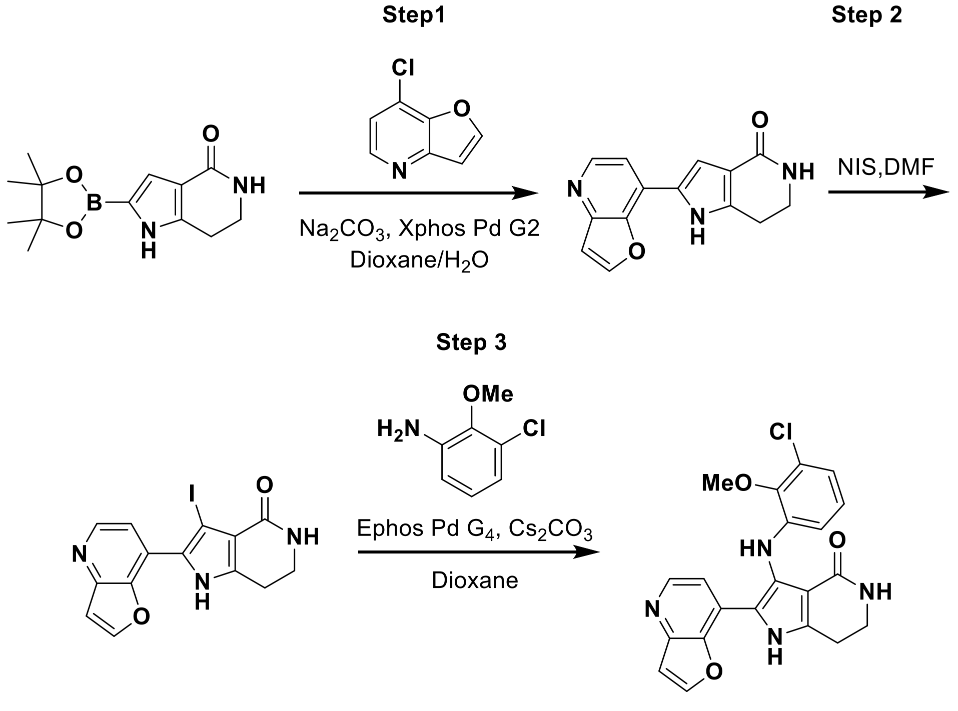

Compound 34

Step 1: 2-[Furo[3,2-b]pyridin-7-yl]-1*H*,5*H*,6*H*,7*H*-pyrrolo[3,2-c]pyridin-4-one. XPhos Pd G2 (232 mg, 0.31 mmol) was added to a mixture of 7-chlorofuro[3,2-b]pyridine (400 mg, 2.60 mmol), 2-(4,4,5,5-tetramethyl-1,3,2-dioxaborolan-2-yl)-1*H*,5*H*,6*H*,7*H*-pyrrolo[3,2-c]pyridin-4-one (1.02 g, 3.91 mmol), and Na_2CO_3 (323 mg, 3.05 mmol) in 1,4-dioxane (15.00 mL) and water (3.00 mL) at RT under a N_2 atmosphere. The resulting mixture was stirred 12 h at 50 °C. The resulting mixture was cooled to RT and extracted with DCM (3 × 300 mL). The combined organic layers were washed with water (3 × 50 mL), dried over anhydrous Na_2SO_4, filtered, and concentrated under reduced pressure. The residue was purified by Prep-TLC (CH_2Cl_2 / MeOH = 10:1) to afford 2-[furo[3,2-b]pyridin-7-yl]-1*H*,5*H*,6*H*,7*H*-pyrrolo[3,2-c]pyridin-4-one (430 mg, 77% yield) as a brown solid. *m/z* (ESI, +ve ion): 254.15 $(M + H)^+$.

Step 2: 2-[Furo[3,2-b]pyridin-7-yl]-3-iodo-1*H*,5*H*,6*H*,7*H*-pyrrolo[3,2-c]pyridin-4-one. To a stirred mixture of 2-[furo[3,2-b]pyridin-7-yl]-1*H*,5*H*,6*H*,7*H*-pyrrolo[3,2-c]pyridin-4-one (150 mg, 0.590 mmol) in DMF (6.00 mL) was added NIS (160 mg, 0.710 mmol) in three portions at RT under a N_2 atmosphere. The resulting mixture was stirred for 1 h at RT before the reaction mixture was filtered and the filter cake was washed with water (3 × 5 mL) to afford 2-[furo[3,2-b]pyridin-7-yl]-3-iodo-1*H*,5*H*,6*H*,7*H*-pyrrolo[3,2-c]pyridin-4-one (220 mg, 91% yield) as a yellow solid. *m/z* (ESI, +ve ion): 379.95 $(M + H)^+$.

Step 3: 3-[(3-Chloro-2-methoxyphenyl)amino]-2-[furo[3,2-b]pyridin-7-yl]-1*H*,5*H*,6*H*,7*H*-pyrrolo[3,2-c]pyridin-4-one (Compound 34). 3-Chloro-2-methoxyaniline (42.0 mg, 0.260 mmol), Ephos Pd G4 (48.0 mg, 0.050 mmol), and Cs_2CO_3 (172 mg, 0.530 mmol) were added to a solution of 2-[furo[3,2-b]pyridin-7-yl]-3-iodo-1*H*,5*H*,6*H*,7*H*-pyrrolo[3,2-c]pyridin-4-one (100 mg, 0.26 mmol) in 1,4-dioxane (3.00 mL) under an argon atmosphere at RT. The resulting mixture was stirred for 12 h at 50 °C. The resulting mixture was cooled to RT, concentrated under reduced pressure, and purified by Prep-TLC (CH_2Cl_2 / MeOH, 10:1) to afford impure product (80 mg), which was purified by Prep-HPLC (Column: XBridge Prep C18 OBD Column, 30 × 100 mm, 5 μm; Mobile Phase A: Water (10 mmol/L NH_4HCO_3), Mobile Phase B: MeCN; Flow rate: 60 mL/min; Gradient: 23% B to 35% B in 12 min; Rt: 11.65 min) to afford 3-[(3-chloro-2-methoxyphenyl)amino]-2-[furo[3,2-b]pyridin-7-yl]-1*H*,5*H*,6*H*,7*H*-pyrrolo[3,2-c]pyridin-4-one (50.6 mg, 47% yield) as a yellow solid. ^1H NMR (300 MHz, DMSO-d_6) δ = 11.64 (s, 1H), 8.39–8.27 (m, 2H), 7.58

(s, 1H), 7.30–7.18 (m, 2H), 7.12 (d, *J* = 2.3 Hz, 1H), 6.70–6.57 (m, 2H), 6.14 (dd, *J* = 7.8, 1.9 Hz, 1H), 3.91 (s, 3H), 3.44 (td, *J* = 6.8, 2.4 Hz, 2H), 2.93 (t, *J* = 6.8 Hz, 2H). *m/z* (ESI, +ve ion): 408.95 $(M + H)^+$.

Compound 35

(7*S*)-3-[(3-fluoro-2-methoxyphenyl)amino]-7-methyl-2-([1,2]thiazolo[4,5-b]pyridin-7-yl)-5*H*,6*H*,7*H*-pyrazolo[1,5-a]pyrazin-4-one.

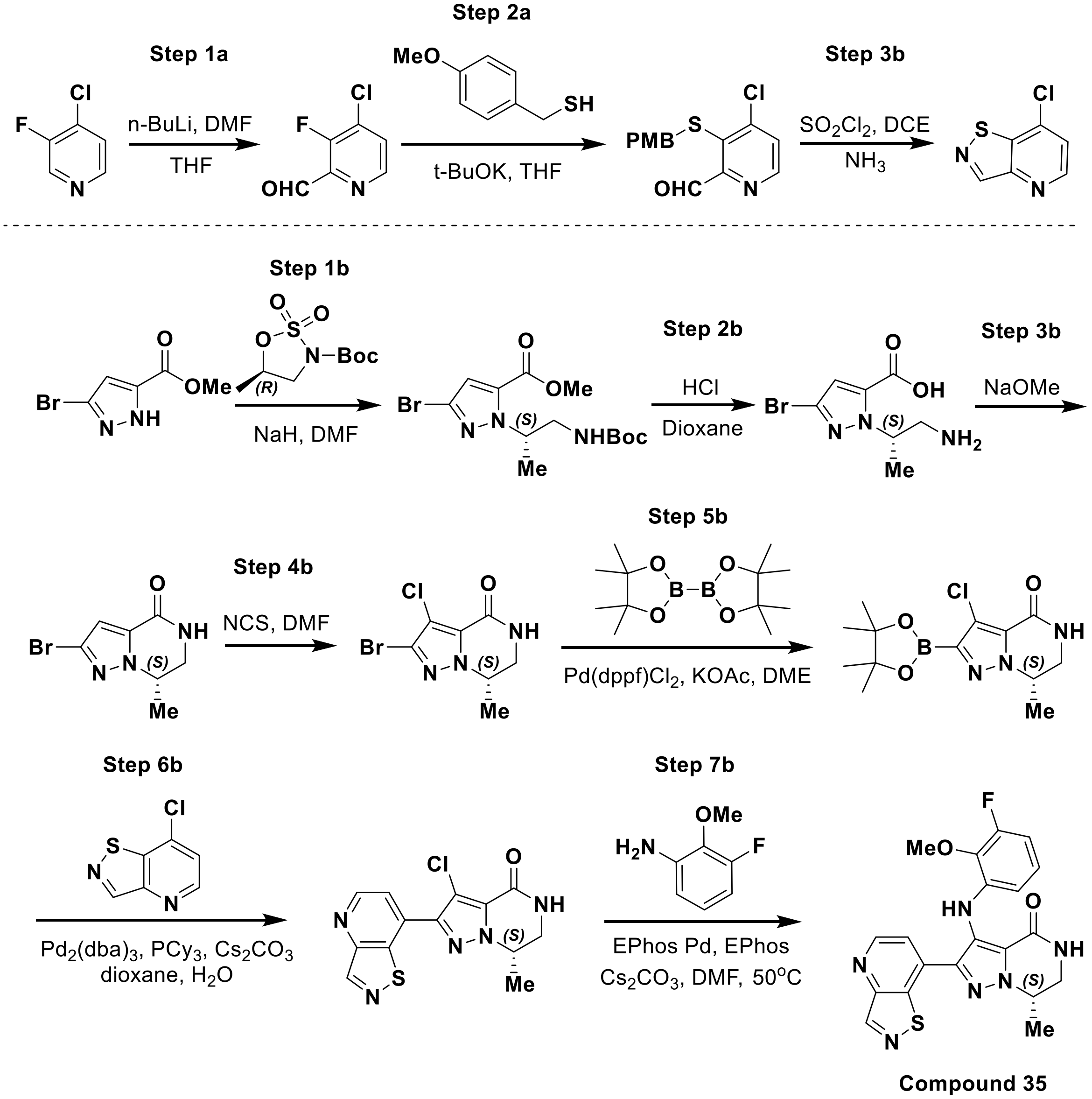

Step 1a: 4-Chloro-3-fluoropyridine-2-carbaldehyde. To a stirred solution of 2,2,6,6-tetramethylpiperidine (5.91 g, 41.8 mmol) and in THF (70 mL) was added 2.5 M *n*-BuLi in hexanes (16.0 mL, 39.9 mmol) dropwise at 0 °C under an argon atmosphere. The resulting mixture was stirred for 1 h at −5 °C. The mixture was then cooled to −78 °C before a solution of 4-chloro-3-fluoropyridine

(5.00 g, 38.0 mmol) in THF (40 mL) was added dropwise. The resultant reaction mixture stirred for 2 h at −78 °C before a solution of DMF (2.92 g, 39.9 mmol) in THF (20 mL) was introduced. The resulting mixture was stirred for additional 1 h at −78 °C. Next, sat. aq. NH_4Cl (50 mL) was introduced at at 0 °C. The resulting mixture was extracted with EtOAc (3 × 200 mL). The combined organic layers were dried over anhydrous Na_2SO_4, filtered, and concentrated under reduced pressure. The residue was purified by silica gel column chromatography, eluted with PE / EA (2:1) to afford 4-chloro-3-fluoropyridine-2-carbaldehyde (5.5 g, 91% yield) as a yellow solid. *m/z* (ESI, +ve ion): 160.00 $(M + H)^+$.

Step 2a: 4-Chloro-3-([(4-methoxyphenyl)methyl]sulfanyl)pyridine-2-carbaldehyde. To a stirred solution of 4-chloro-3-fluoropyridine-2-carbaldehyde (2.00 g, 11.5 mmol) and (4-methoxyphenyl)methanethiol (1.78 g, 11.5 mmol) in THF (50 mL) was added t-BuOK (1.29 g, 11.5 mmol) in portions at 0 °C under an argon atmosphere. After 1 h, the resulting mixture was filtered, the filter cake was washed with ethyl acetate (3 × 10 mL), and the filtrate was concentrated under reduced pressure. The residue was purified by silica gel column chromatography, eluted with CH_2Cl_2/MeOH (50:1) to afford 4-chloro-3-([(4-methoxyphenyl)methyl]sulfanyl)pyridine-2-carbaldehyde (2.00 g, 59% yield) as a yellow solid. *m/z* (ESI, +ve ion): 294.1 $(M + H)^+$.

Step 3a: 7-Chloro-[1,2]thiazolo[4,5-b]pyridine. To a stirred solution of 4-chloro-3-([(4-methoxyphenyl)methyl]sulfanyl)pyridine-2-carbaldehyde (2.2 g, 7.49 mmol, 1.00 equiv) in DCE (50 mL) was added SO_2Cl_2 (1.21 mL, 15.0 mmol) dropwise at 0 °C under an argon atmosphere. The resulting mixture was stirred for 1 h at RT before 7M NH_3 in MeOH (5.35 mL, 37.4 mmol) was added dropwise over 5 min at RT. The resulting mixture was stirred for 1 h at RT before the reaction mixture was concentrated under reduced pressure. The residue was purified by silica gel column chromatography, eluted with CH_2Cl_2/MeOH (10:1) to afford 7-chloro-[1,2]thiazolo[4,5-b]pyridine (400 mg, 28% yield) as a yellow solid. *m/z* (ESI, +ve ion): 170.95 $(M + H)^+$.

Step 1b: Methyl 5-bromo-2-[(2*S*)-1-[(*tert*-butoxycarbonyl)amino]propan-2-yl]pyrazole-3-carboxylate. To a stirred solution of *tert*-butyl (*R*)-5-methyl-1,2,3-oxathiazolidine-3-carboxylate 2,2-dioxide (6.94 g, 29.3 mmol) and methyl 5-bromo-2*H*-pyrazole-3-carboxylate (5.00 g, 24.4 mmol) in DMF (50 mL) was added NaH (1.46 g, 36.6 mmol, 60% in mineral oil) in portions at 0 °C under a N_2 atmosphere. After 1 h, H_2O (300 mL) and the resulting mixture was extracted with EtOAc (3 × 120 mL). The combined organic layers were washed with brine (2 × 120 mL), dried over anhydrous Na_2SO_4, filtered, and concentrated under reduced pressure. The residue was purified by silica gel column chromatography, eluted with DCM:MeOH (15:1) to afford methyl 5-bromo-2-[(2*S*)-1-[(*tert*-butoxycarbonyl)amino]propan-2-yl]pyrazole-3-carboxylate (3.20 g, 29% yield) as a white solid. *m/z* (ESI, +ve ion): 361.95 $(M + H)^+$.

Step 2b: 2-[(2*S*)-1-Aminopropan-2-yl]-5-bromopyrazole-3-carboxylate. HCl in 1,4-dioxane (50 mL, 1 M) was added to a stirred solution of methyl 5-bromo-2-[(2*S*)-1-[(*tert*-butoxycarbonyl)amino]propan-2-yl]pyrazole-3-carboxylate (3.50 g, 9.66 mmol) in 1,4-dioxane (50 mL) at RT under a N_2 atmosphere. After 1 h, the resulting mixture was concentrated under reduced pressure to afford methyl 2-[(2*S*)-1-aminopropan-2-yl]-5-bromopyrazole-3-carboxylate (2.60 g, 82% yield) as a white solid which was used without further purification. *m/z* (ESI, +ve ion): 263.95 $(M + H)^+$.

Step 3b: (7*S*)-2-Bromo-7-methyl-5*H*,6*H*,7*H*-pyrazolo[1,5-a]pyrazin-4-one. To a stirred solution of methyl 2-[(2*S*)-1-aminopropan-2-yl]-5-bromopyrazole-3-carboxylate (2.5 g, 9.54 mmol) in MeOH (50 mL) was added NaOMe (2.32 g, 42.9 mmol, 1 M in MeOH) at −78 °C under a N_2 atmosphere. After 3 h, the resulting mixture was extracted with EtOAc (2 × 50 mL). The combined organic layers were washed with brine (2 × 40 mL), dried over anhydrous Na_2SO_4, filtered, and concentrated under reduced pressure to afford (7*S*)-2-Bromo-7-methyl-5*H*,6*H*,7*H*-pyrazolo[1,5-a]pyrazin-4-one (2.10 g, 74% yield) as a white solid which was used without further purification. *m/z* (ESI, +ve ion): 229.95 $(M + H)^+$.

Step 4b: (7*S*)-2-Bromo-3-chloro-7-methyl-5*H*,6*H*,7*H*-pyrazolo[1,5-a]pyrazin-4-one. To a stirred solution of (7*S*)-2-Bromo-7-methyl-5*H*,6*H*,7*H*-pyrazolo[1,5-a]pyrazin-4-one (2.00 g, 8.69 mmol) in DMF (30 mL) was added NCS (1.39 g, 10.4 mmol) in portions at 0 °C. The reaction mixture was stirred for 2 h at 50 °C, then cooled to RT diluted with H_2O (100 mL). The resulting mixture was filtered, the filter cake was washed with H_2O (20 mL), and the filter cake was dried under reduced pressure to afford (7*S*)-2-bromo-3-chloro-7-methyl-5*H*,6*H*,7*H*-pyrazolo[1,5-a]pyrazin-4-one (1.90 g, 79% yield) as a white solid which was used without further purification. *m/z* (ESI, +ve ion): 265.90 $(M + H)^+$.

Step 5b: (7*S*)-3-Chloro-7-methyl-2-(4,4,5,5-tetramethyl-1,3,2-dioxaborolan-2-yl)-5*H*,6*H*,7*H*-pyrazolo[1,5-a]pyrazin-4-one. To a solution of (7*S*)-2-bromo-3-chloro-7-methyl-5*H*,6*H*,7*H*-pyrazolo[1,5-a]pyrazin-4-one (2.30 g, 8.70 mmol) and bis(pinacolato)diboron (4.42 g, 17.4 mmol) in DME (20 mL) were added KOAc (1.71 g, 17.4 mmol) and Pd(dppf)Cl_2·CH_2Cl_2 (0.71

g, 0.870 mmol) under a N_2 atmosphere. After stirring for 16 h at 100 °C the resulting mixture was filtered, the filter cake was washed with DCM (100 mL), the filtrate was concentrated under reduced pressure, and diluted with EtOAc (50 mL) leading to solid precipitation. The resulting precipitate was filtered, the filter cake was washed with EtOAc (25 mL), and dried under reduced pressure to afford (7*S*)-3-chloro-7-methyl-2-(4,4,5,5-tetramethyl-1,3,2-dioxaborolan-2-yl)-5*H*,6*H*,7*H*-pyrazolo[1,5-a]pyrazin-4-one (7 g) as a brown solid which was used without further purification. *m/z* (ESI, +ve ion): 230.00 $(M + H)^+$.

Step 6b: (*S*)-3-chloro-2-(isothiazolo[4,5-b]pyridin-7-yl)-7-methyl-6,7-dihydropyrazolo[1,5-a]pyrazin-4(5*H*)-one. A solution of 7-chloro-[1,2]thiazolo[4,5-b]pyridine (350 mg, 2.05 mmol) and (7*S*)-3-chloro-7-methyl-2-(4,4,5,5-tetramethyl-1,3,2-dioxaborolan-2-yl)-5*H*,6*H*,7*H*-pyrazolo[1,5-a]pyrazin-4-one (2.56 g, 8.20 mmol) in 1,4-diaxone/H_2O (3 mL; 5:1) was charged with $Pd_2(dba)_3$ (188 mg, 0.205 mmol), PCy_3 (57.5 mg, 0.205 mmol), and Cs_2CO_3 (2.01 g, 6.15 mmol). The resulting solution was stirred and warmed to at 60 °C. After 2 h, the resultant mixture was allowed to cool to RT and the solid precipitate was collected by filtration and washed with MeOH (3 × 5 mL) to afford (*S*)-3-chloro-2-(isothiazolo[4,5-b]pyridin-7-yl)-7-methyl-6,7-dihydropyrazolo[1,5-a]pyrazin-4(5*H*)-one (300 mg, 46% yield) as a grey solid which was used without further purification. *m/z* (ESI, +ve ion): 320.10 $(M + H)^+$.

Step 7b: (*S*)-3-((3-fluoro-2-methoxyphenyl)amino)-2-(isothiazolo[4,5-b]pyridin-7-yl)-7-methyl-6,7-dihydropyrazolo[1,5-a]pyrazin-4(5*H*)-one (Compound 35). To a solution of (*S*)-3-chloro-2-(isothiazolo[4,5-b]pyridin-7-yl)-7-methyl-6,7-dihydropyrazolo[1,5-a]pyrazin-4(5*H*)-one (150 mg, 0.469 mmol) and 3-fluoro-2-methoxyaniline (199 mg, 1.41 mmol) in DMF (2 mL) was added EPhos Pd G4 (129 mg, 0.141 mmol) and Cs_2CO_3 (459 mg, 1.41 mmol). The resulting reaction mixture was stirred and warmed to 50 °C. After 2 h, the resulting reaction mixture was concentrated under reduced pressure and the residue was purified by Prep-TLC with DCM/MeOH (25:1). The crude product was purified by Prep-Flash-HPLC (Column: YMC-Actus Triart C18, 30 × 150 mm, 5 μm; Mobile Phase A: Water (10 mmol/L NH_4HCO_3+0.1%NH_3-H_2O), Mobile Phase B: MeCN; Flow rate: 60 mL/min; Gradient: 46% B to 56% B in 8 min; Rt: 6.60 min) afforded (*S*)-3-((3-fluoro-2-methoxyphenyl)amino)-2-(isothiazolo[4,5-b]pyridin-7-yl)-7-methyl-6,7-dihydropyrazolo[1,5-a]pyrazin-4(5*H*)-one (67.8 mg, 33% yield) as a white solid. ^{1}H NMR (300 MHz, DMSO-d_6) δ = 9.31 (d, *J* = 1.4 Hz, 1H), 8.79 (d, *J* = 4.8 Hz, 1H), 8.42 (d, *J* = 3.2 Hz, 1H), 7.67 (d, *J* = 4.8 Hz, 1H), 7.48 (s, 1H), 6.78–6.68 (m, 1H), 6.66–6.57 (m, 1H), 6.05 (d, *J* = 8.1 Hz, 1H), 4.97–4.51 (m, 1H), 3.98 (s, 3H), 3.81–3.67 (m, 1H), 3.56–3.40 (m, 1H), 1.71 (d, *J* = 6.4 Hz, 3H). *m/z* (ESI, +ve ion): 425.10 $(M + H)^+$.

Compounds 36 and 37

3-[(3-Chloro-2-methoxyphenyl)amino]-2-(3-(2-[(2*S*)-1-(prop-2-enoyl)azetidin-2-yl]ethynyl)pyridin-4-yl)-1*H*,5*H*,6*H*,7*H*-pyrrolo[3,2-c]pyridin-4-one (Compound 36) and 3-[(3-chloro-2-methoxyphenyl)amino]-2-(3-(2-[(2*R*)-1-(prop-2-enoyl)azetidin-2-yl]ethynyl)pyridin-4-yl)-1*H*,5*H*,6*H*,7*H*-pyrrolo[3,2-c]pyridin-4-one (Compound 37).

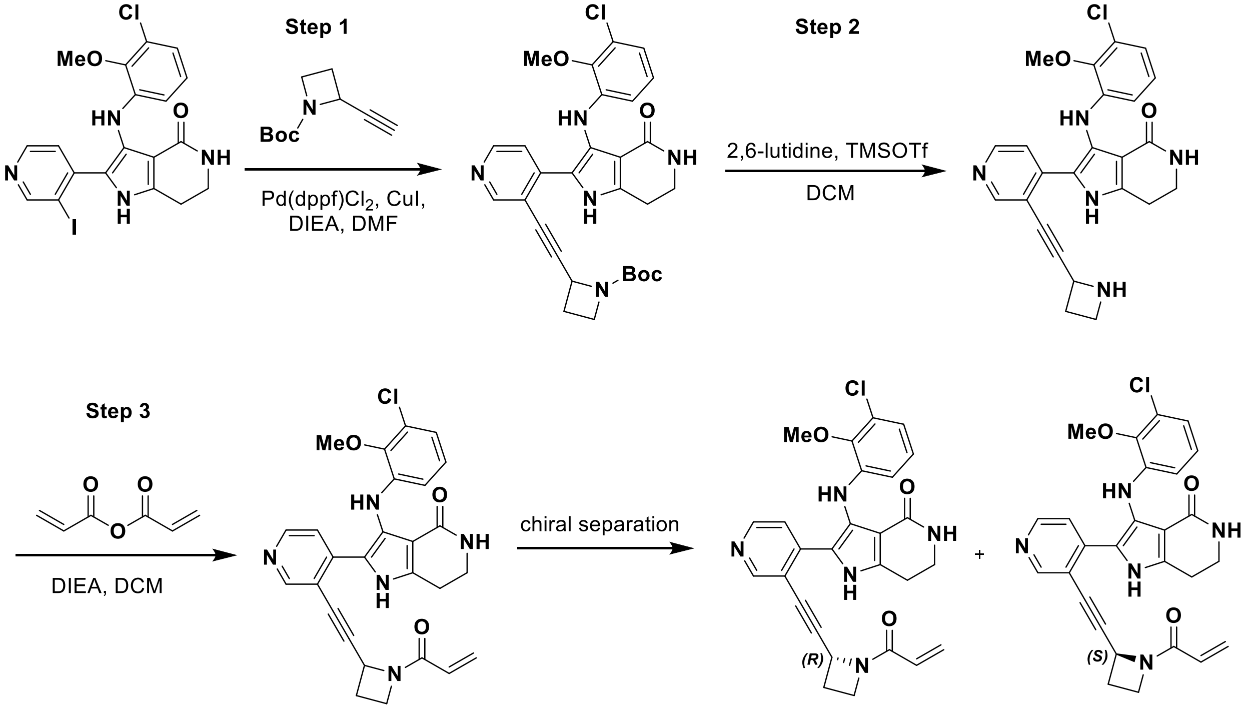

Step 1: *tert*-Butyl 2-[2-(4-(3-[(3-chloro-2-methoxyphenyl)amino]-4-oxo-1*H*,5*H*,6*H*,7*H*-pyrrolo[3,2-c]pyridin-2-yl)pyridin-3-yl)ethynyl]azetidine-1-carboxylate. To a stirred solution of 3-[(3-chloro-2-methoxyphenyl)amino]-2-(3-iodopyridin-4-yl)-1*H*,5*H*,6*H*,7*H*-pyrrolo[3,2-c]pyridin-4-one (200 mg, 0.404 mmol) and *tert*-butyl 2-ethynylazetidine-1-carboxylate (183 mg, 1.01 mmol) in DMF (4.0 mL) was added Pd(dppf)Cl_2·CH_2Cl_2 (82.3 mg, 0.101 mmol), CuI (38.5 mg, 0.202 mmol) and *N*,*N*-diisopropylethylamine (157 mg, 1.21 mmol) at RT under a argon atmosphere. The resulting mixture was stirred for 12 h at 50 °C then cooled to RT and concentrated under reduced pressure. The residue was purified by silica gel column chromatography, eluted with CH_2Cl_2/MeOH (10:1) to afford *tert*-butyl 2-[2-(4-(3-[(3-chloro-2-methoxyphenyl)amino]-4-oxo-1*H*,5*H*,6*H*,7*H*-pyrrolo[3,2-c]pyridin-2-yl)pyridin-3-yl)ethynyl]azetidine-1-carboxylate (220 mg, 99% yield) as a brown solid. *m/z* (ESI, +ve ion): 548.10 $(M + H)^+$.

Step 2: 2-(3-[2-(Azetidin-2-yl)ethynyl]pyridin-4-yl)-3-[(3-chloro-2-methoxyphenyl)amino]-1*H*,5*H*,6*H*,7*H*-pyrrolo[3,2-c]pyridin-4-one. To a stirred solution of *tert*-butyl 2-[2-(4-(3-[(3-chloro-2-methoxyphenyl)amino]-4-oxo-1*H*,5*H*,6*H*,7*H*-pyrrolo[3,2-c]pyridin-2-yl)pyridin-3-yl)ethynyl]azetidine-1-carboxylate (170 mg, 0.310 mmol) and 2,6-lutidine (199 mg, 1.86 mmol) in DCM (3.0 mL) was added TMSOTf (283 mL, 345 mg, 1.55 mmol) dropwise at RT under a N_2 atmosphere. The resulting mixture was stirred for 1 h at RT and was then concentrated under reduced pressure. The residue was purified by reverse-phase flash chromatography (Column: C18 silica gel; mobile phase, MeCN in Water (10mmol/L NH_4HCO_3), 10% to 50% gradient in 10 min; detector, UV 254 nm) to afford 2-(3-[2-(Azetidin-2-yl)ethynyl]pyridin-4-yl)-3-[(3-chloro-2-methoxyphenyl)amino]-1*H*,5*H*,6*H*,7*H*-pyrrolo[3,2-c]pyridin-4-one (100 mg, 72% yield) as a light yellow solid. *m/z* (ESI, +ve ion): 448.00 $(M + H)^+$.

Step 3: 3-[(3-Chloro-2-methoxyphenyl)amino]-2-(3-(2-[(2*S*)-1-(prop-2-enoyl)azetidin-2-yl]ethynyl)pyridin-4-yl)-1*H*,5*H*,6*H*,7*H*-pyrrolo[3,2-c]pyridin-4-one (Compound 36) and 3-[(3-chloro-2-methoxyphenyl)amino]-2-(3-(2-[(2*R*)-1-(prop-2-enoyl)azetidin-2-yl]ethynyl)pyridin-4-yl)-1*H*,5*H*,6*H*,7*H*-pyrrolo[3,2-c]pyridin-4-one (Compound 37). To a stirred solution of 2-(3-[2-(Azetidin-2-yl)ethynyl]pyridin-4-yl)-3-[(3-chloro-2-methoxyphenyl)amino]-1*H*,5*H*,6*H*,7*H*-pyrrolo[3,2-c]pyridin-4-one (120 mg, 0.268 mmol) and prop-2-enoyl prop-2-enoate (67.6 mg, 0.536 mmol) in DCM (2.0 mL) was added *N*,*N*-diisopropylethylamine (233 mL, 173.13 mg, 1.34 mmol) dropwise at 0 °C under a N_2 atmosphere. The resulting mixture was stirred

for 1 h at RT before brine (25 mL) was introduced. The resulting mixture was extracted with EtOAc (3 × 10 mL). The combined organic layers were dried over anhydrous Na_2SO_4, filtered, and the filtrate was concentrated under reduced pressure. The residue (150 mg) was purified by Prep-HPLC (Column: Xselect CSH C18 OBD Column 30 × 150 mm 5 μm; Mobile Phase A: Water (0.1%FA), Mobile Phase B: MeCN; Flow rate: 60 mL/min; Gradient: 18% B to 38% B in 10 min) to afford racemic 2-(3-((1-acryloylazetidin-2-yl)ethynyl)pyridin-4-yl)-3-((3-chloro-2-methoxyphenyl)amino)-1,5,6,7-tetrahydro-4*H*-pyrrolo[3,2-c]pyridin-4-one (70 mg, 52% yield). Chiral separation was conducted (Column: CHIRALPAK IE, 2 × 25 cm, 5 μm; Mobile Phase A: Hex:DCM=3:1 (0.5% 2 M NH_3-MeOH), Mobile Phase B: IPA; Flow rate: 17 mL/min; Isocratic gradient = 15% B) to afford 3-[(3-chloro-2-methoxyphenyl)amino]-2-(3-(2-[(2*R*)-1-(prop-2-enoyl)azetidin-2-yl]ethynyl)pyridin-4-yl)-1*H*,5*H*,6*H*,7*H*-pyrrolo[3,2-c]pyridin-4-one (Compound **37**) (Rt = 42.83, peak 1, 17.5 mg, 25% yield) as the first-eluting peak as a yellow solid and 3-[(3-chloro-2-methoxyphenyl)amino]-2-(3-(2-[(2*S*)-1-(prop-2-enoyl)azetidin-2-yl]ethynyl)pyridin-4-yl)-1*H*,5*H*,6*H*,7*H*-pyrrolo[3,2-c]pyridin-4-one (Compound **36**) (Rt: 55.23, peak 3, 14.3 mg, 20% yield) as the second-eluting peak as a yellow solid. ^{1}H NMR (400 MHz, DMSO-d_6) δ = 10.91 (s, 1H), 8.61 (s, 1H), 8.15 (d, *J* = 5.8 Hz, 1H), 7.84 (s, 1H), 7.43 (d, *J* = 5.7 Hz, 1H), 6.78–6.71 (m, 1H), 6.63 (t, *J* = 8.1 Hz, 1H), 6.45–6.33 (m, 1H), 6.30–6.19 (m, 2H), 5.82–5.75 (m, 1H), 5.36 (s, 1H), 5.30–5.20 (m, 1H), 4.43 (td, *J* = 8.9, 5.7 Hz, 1H), 4.34–4.26 (m, 1H), 4.09 (s, 3H), 3.69–3.55 (m, 2H), 3.29–3.18 (m, 2H), 2.85–2.74 (m, 1H), 2.71–2.60 (m, 1H). *m/z* (ESI, +ve ion): 502.00 $(M + H)^+$. Absolute stereochemistry arbitrarily assigned.

Compound 38

3-[(3-Chloro-2-methoxyphenyl)amino]-2-(3-(2-[(2*R*)-1-(prop-2-enoyl)pyrrolidin-2-yl]ethynyl)pyridin-4-yl)-1*H*,5*H*,6*H*,7*H*-pyrrolo[3,2-c]pyridin-4-one.

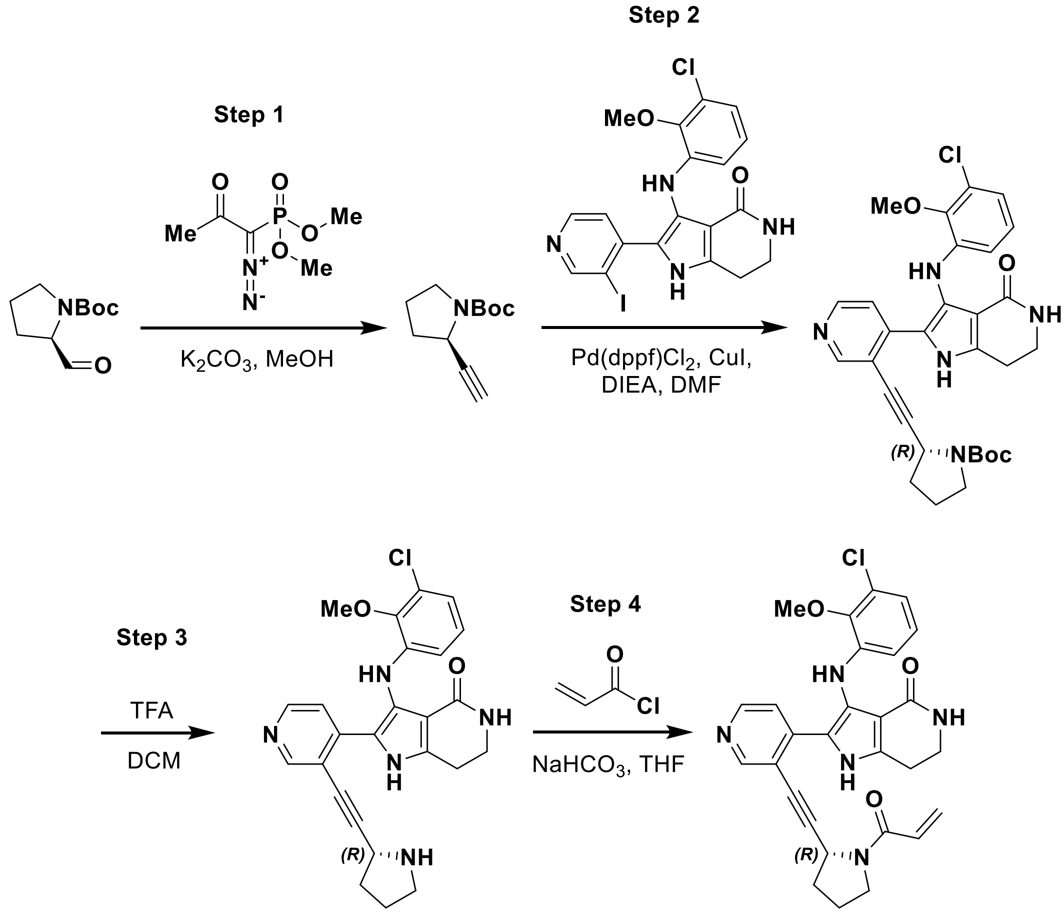

Compound 38

Step 1: 2-Ethynylpyrrolidine-1-carboxylate. To a stirred mixture of *tert*-butyl (2*R*)-2-formylpyrrolidine-1-carboxylate (2.00 g, 10.0 mmol) and K_2CO_3 (2.77 g, 20.1 mmol) in MeOH (15 mL) was added dimethyl (1-diazo-2-oxopropyl)phosphonate (2.31 g, 12.1 mmol) in portions at 0 °C under a N_2 atmosphere. The resulting mixture was stirred for 1 h at 0 °C before sat. aq. potassium sodium tartrate was introduced at 0 °C. The aqueous layer was extracted with EtOAc (2 × 100 mL). The combined organic layers were dried over anhydrous Na_2SO_4, filtered, and concentrated under reduced pressure. The residue was purified by silica gel column chromatograph, eluted with PE / EA (10:1) to afford *tert*-butyl (2*R*)-2-ethynylpyrrolidine-1-carboxylate (1.70 g, 87% yield) as a light yellow oil.

Step 2: *tert*-butyl (*R*)-2-((4-(3-((3-chloro-2-methoxyphenyl)amino)-4-oxo-4,5,6,7-tetrahydro-1*H*-pyrrolo[3,2-c]pyridin-2-yl)pyridin-3-yl)ethynyl)pyrrolidine-1-carboxylate. To a stirred mixture of 3-[(3-chloro-2-methoxyphenyl)amino]-2-(3-iodopyridin-4-yl)-1*H*,5*H*,6*H*,7*H*-pyrrolo[3,2-c]pyridin-4-one (200 mg, 0.40 mmol) and *tert*-butyl (2*R*)-2-ethynylpyrrolidine-1-carboxylate (395 mg, 2.02 mmol) in DMF (3 mL) was added $Pd(dppf)Cl_2{\cdot}CH_2Cl_2$ (165 mg, 0.20 mmol) and *N,N*-diisopropylethylamine (0.5 mL) dropwise at room temperature under an argon atmosphere. The resulting mixture was stirred for 2 h at 50 °C. The resultant reaction mixture was cooled to RT and concentrated under reduced pressure. The residue was purified by reverse-phase flash chromatography (Column: C18 spherical column; mobile phase: MeCN in water, 10% to 50% gradient in 30 min) afford *tert*-butyl (*R*)-2-((4-(3-((3-chloro-2-methoxyphenyl)amino)-4-oxo-4,5,6,7-tetrahydro-1*H*-pyrrolo[3,2-c]pyridin-2-yl)pyridin-3-yl)ethynyl)pyrrolidine-1-carboxylate (230 mg, 91% yield) as a yellow solid. *m/z* (ESI, +ve ion): 562.15 $(M + H)^+$.

Step 3: (*R*)-3-((3-Chloro-2-methoxyphenyl)amino)-2-(3-(pyrrolidin-2-ylethynyl)pyridin-4-yl)-1,5,6,7-tetrahydro-4*H*-pyrrolo[3,2-c]pyridin-4-one. To a solution of *tert*-butyl (*R*)-2-((4-(3-((3-chloro-2-methoxyphenyl)amino)-4-oxo-4,5,6,7-tetrahydro-1*H*-pyrrolo[3,2-c]pyridin-2-yl)pyridin-3-yl)ethynyl)pyrrolidine-1-carboxylate (200 mg, 0.36 mmol) in DCM (3 mL) was added TFA (1 mL) at RT under a N_2 atmosphere. After 1 h, the resulting mixture was concentrated under reduced pressure to afford (*R*)-3-((3-chloro-2-methoxyphenyl)amino)-2-(3-(pyrrolidin-2-ylethynyl)pyridin-4-yl)-1,5,6,7-tetrahydro-4*H*-pyrrolo[3,2-c]pyridin-4-one (460 mg) as a red oil which was used in the next step directly without further purification. *m/z* (ESI, +ve ion): 462.05 $(M + H)^+$.

Step 4: (*R*)-2-(3-((1-Acryloylpyrrolidin-2-yl)ethynyl)pyridin-4-yl)-3-((3-chloro-2-methoxyphenyl)amino)-1,5,6,7-tetrahydro-4*H*-pyrrolo[3,2-c]pyridin-4-one (Compound 38). A solution of (*R*)-3-((3-chloro-2-methoxyphenyl)amino)-2-(3-(pyrrolidin-2-ylethynyl)pyridin-4-yl)-1,5,6,7-tetrahydro-4*H*-pyrrolo[3,2-c]pyridin-4-one (101 mg, 0.220 mmol) in THF (2.0 mL) was treated with $NaHCO_3$(sat. aq.) (2.0 mL) for 5 min at −30 °C under a N_2 atmosphere followed by the addition of acryloyl chloride (18 mg, 0.19 mmol) dropwise at 0 °C. The resulting mixture was stirred for 30 min at RT before MeOH (1 mL) was introduced at 0 °C. The resulting mixture was concentrated under reduced pressure. The residue was purified by silica gel column chromatography, eluted with DCM / MeOH (25:1) to afford 80 mg of impure material which was purified by Prep-HPLC (Column: XBridge Prep OBD C18 Column, 30 × 150 mm, 5 μm; Mobile Phase A: Water (10 mmol/L NH_4HCO_3), Mobile Phase B: MeCN; Flow rate: 60 mL/min; Gradient: 27% B to 47% B in 10 min; Rt: 8.95 min) to afford (*R*)-2-(3-((1-acryloylpyrrolidin-2-yl)ethynyl)pyridin-4-yl)-3-((3-chloro-2-methoxyphenyl)amino)-1,5,6,7-tetrahydro-4*H*-pyrrolo[3,2-c]pyridin-4-one (52.7 mg, 47% yield) as a yellow solid. ^{1}H NMR (400 MHz, DMSO-d_6) δ = 11.14 (s, 1H), 8.50 (d, *J* = 7.5 Hz, 1H), 8.26 (s, 1H), 7.31 (t, *J* = 6.9 Hz, 2H), 6.74 (s, 1H), 6.67–6.49 (m, 3H), 6.27–6.21 (m, 1H), 6.13– 6.06 (m, 1H), 5.64 (s, 1H), 4.94 (s, 1H), 3.87 (d, *J* = 7.5 Hz, 3H), 3.75–3.48 (m, 3H), 3.48–3.40 (m, 2H), 2.95–2.93 (m, 2H), 2.22 (s, 1H), 2.08 (s, 3H). *m/z* (ESI, +ve ion): 515.95 $(M + H)^+$.

Compound 39

(*R*)-2-(3-((1-Acryloylpyrrolidin-2-yl)ethynyl)pyridin-4-yl)-3-((3-fluoro-2-methoxyphenyl)amino)-1,5,6,7-tetrahydro-4*H*-pyrrolo[3,2-c]pyridin-4-one was prepared according to the procedure described for Compound **38**, using 3-[(3-fluoro-2-methoxyphenyl)amino]-2-(3-iodopyridin-4-yl)-1*H*,5*H*,6*H*,7*H*-pyrrolo[3,2-c]pyridin-4-one in Step 2 heating at 50 °C for 2 h (56% yield). Next, Step 3 (97% yield) and Step 4 afforded the title compound (5.60 mg, 8.2% yield) after Prep-HPLC (Column: XBridge Prep OBD C18 Column, 30 × 150 mm, 5μm; Mobile Phase A: Water (10 mmol/L NH_4HCO_3), Mobile Phase B: MeCN; Flow rate: 60 mL/min; Gradient: 25% B to 45% B in 8 min; Rt: 7.1 min) as an off-white solid. ^{1}H NMR (400 MHz, DMSO-d_6) δ = 11.50–11.30 (s, 1H), 8.54 (d, *J* = 5.2 Hz, 1H), 8.37–8.27 (m, 1H), 7.53 (s, 1H), 7.39–7.28 (m, 1H), 7.18–7.10 (m, 1H), 6.71–6.36 (m, 3H), 6.25–6.09 (m, 1H), 5.96–5.89 (m, 1H), 5.77–5.58 (m, 1H), 5.07–4.93 (m, 1H), 3.88 (d, *J* = 16 Hz, 3H), 3.77–3.68 (m, 1H), 3.62–3.42 (m, 3H), 2.99–2.81 (m, 2H), 2.27–1.88 (m, 4H). *m/z* (ESI, +ve ion): 500.20 $(M + H)^+$.

Compound 40

(*S*)-2-(3-((1-Acryloylpyrrolidin-2-yl)ethynyl)pyridin-4-yl)-3-((3-chloro-2-methoxyphenyl)amino)-1,5,6,7-tetrahydro-4*H*-pyrrolo[3,2-c]pyridin-4-one was prepared according to the procedure described for Compound **38**, using *tert*-butyl (2S)-2-formylpyrrolidine-1-carboxylate in Step 1 (75% yield). Next, Step 2 (80% yield), Step 3 (41% yield) and Step 4 afforded the title compound after Prep-TLC in DCM / MeOH (20:1) (20.6 mg, 31% yield) as a yellow solid. ^{1}H NMR (400 MHz, $CDCl_3$) δ = 11.13 (s, 1H), 8.53 (s, 1H), 8.11 (s, 1H), 7.83 (s, 1H), 7.41 (d, *J* = 5.7 Hz, 1H), 6.78–6.68 (m, 1H), 6.66–6.33 (m, 3H), 6.29–6.06 (m, 1H), 5.83–5.64 (m, 1H), 5.30 (d, *J* = 3.2 Hz, 1H), 5.01–4.72 (m, 1H), 4.07 (s, 3H), 3.85–3.76 (m, 1H), 3.74–3.54 (m, 3H), 3.38–3.15 (m, 2H), 2.46–2.16 (m, 3H), 2.16–1.89 (m, 1H). *m/z* (ESI, +ve ion): 516.00 $(M + H)^+$.

Compounds 41 and 42

(*R*)-2-(3-((1-Acryloylpiperidin-2-yl)ethynyl)pyridin-4-yl)-3-((3-chloro-2-methoxyphenyl)amino)-1,5,6,7-tetrahydro-4*H*-pyrrolo[3,2-c]pyridin-4-one (Compound 41) and **(*S*)-2-(3-((1-acryloylpiperidin-2-yl)ethynyl)pyridin-4-yl)-3-((3-chloro-2-methoxyphenyl)amino)-1,5,6,7-tetrahydro-4*H*-pyrrolo[3,2-c]pyridin-4-one (Compound 42)** were prepared according to the procedure described for Compound ST-0001211, using racemic *tert*-butyl 2-ethynylpiperidine-1-carboxylate in Step 2 heating at 50 °C for 2 h (65% yield). Next, Step 3 and Step 4 afforded the racemic mixture of the title compounds after silica gel column

chromatography, eluted with CH_2Cl_2 / MeOH (10:1) (60.0 mg, 37% yield over two steps) as a red solid. Prep-CHIRAL-HPLC (Column: CHIRALPAK IH-3, 4.6 × 50 mm, 3μm; Mobile Phase A: hexanes (0.1%DEA): EtOH = 60: 40; Flow rate: 1 mL/min) afforded (*R*)-2-(3-((1-acryloylpiperidin-2-yl)ethynyl)pyridin-4-yl)-3-((3-chloro-2-methoxyphenyl)amino)-1,5,6,7-tetrahydro-4*H*-pyrrolo[3,2-c]pyridin-4-one (Compound **41**) (Rt = 1.81 min, peak 1, 4.50 mg, 6.5% yield) as the first-eluting peak as a light yellow solid. (*S*)-2-(3-((1-Acryloylpiperidin-2-yl)ethynyl)pyridin-4-yl)-3-((3-chloro-2-methoxyphenyl)amino)-1,5,6,7-tetrahydro-4*H*-pyrrolo[3,2-c]pyridin-4-one (Compound **42**) (Rt = 2.60 min, peak 2, 6.80 mg, 10% yield) was isolated as the second-eluting peak as a light yellow solid. ^{1}H NMR (400 MHz, DMSO-d_6) δ = 10.49 (s, 1H), 8.60 (s, 1H), 8.13 (d, *J* = 5.8 Hz, 1H), 7.87 (s, 1H), 7.37 (d, *J* = 5.9 Hz, 1H), 6.78 (dd, *J* = 8.1, 1.5 Hz, 1H), 6.68–6.58 (m, 2H), 6.34–6.28 (m, 1H), 6.21–6.17 (m, 1H), 5.80–5.73 (m, 1H), 5.66 (s, 1H), 5.29 (s, 1H), 4.09 (s, 3H), 3.99–3.89 (m,1H), 3.69–3.55 (m, 2H), 3.42–3.09 (m, 3H), 2.12–2.02 (m, 1H), 1.92 (d, *J* = 6.5 Hz, 5H). *m/z* (ESI, +ve ion): 530.40 $(M + H)^+$. Absolute stereochemistry was arbitrarily.

Compounds 43 and S10

2-(3-(((1*R*,5*S*)-2-Acryloyl-2-azabicyclo[3.1.0]hexan-1-yl)ethynyl)pyridin-4-yl)-3-((3-chloro-2-methoxyphenyl)amino)-1,5,6,7-tetrahydro-4*H*-pyrrolo[3,2-c]pyridin-4-one (Compound 43) and **2-(3-(((1*S*,5*R*)-2-acryloyl-2-azabicyclo[3.1.0]hexan-1-yl)ethynyl)pyridin-4-yl)-3-((3-chloro-2-methoxyphenyl)amino)-1,5,6,7-tetrahydro-4*H*-pyrrolo[3,2-c]pyridin-4-one (Compound S10)** were prepared according to the procedure described for Compound **38**, using racemic *tert*-butyl 1-formyl-2-azabicyclo[3.1.0]hexane-2-carboxylate in Step 1 (87% yield). Next, Step 2 (90% yield), Step 3 and Step 4 afforded the racemic mixture of the title compounds after silica gel column chromatography, eluted with CH_2Cl_2 / MeOH (15:1) (50.0 mg, 21% yield over two steps) as a yellow solid. Prep-CHIRAL-HPLC (Column: CHIRAL ART Amylose-SA, 2 × 25 cm, 5 μm; Mobile Phase A: hexanes (0.5% 2 M NH_3-MeOH), Mobile Phase B: EtOH; Flow rate: 20 mL/min; Gradient: 30% B in 17 min) afforded 2-(3-(((1*R*,5*S*)-2-acryloyl-2-azabicyclo[3.1.0]hexan-1-yl)ethynyl)pyridin-4-yl)-3-((3-chloro-2-methoxyphenyl)amino)-1,5,6,7-tetrahydro-4*H*-pyrrolo[3,2-c]pyridin-4-one (**43**) (Rt = 8.30 min, peak 1, 12.0 mg, 24% yield) as the first-eluting peak as a yellow solid. 2-(3-(((1*S*,5*R*)-2-Acryloyl-2-azabicyclo[3.1.0]hexan-1-yl)ethynyl)pyridin-4-yl)-3-((3-chloro-2-methoxyphenyl)amino)-1,5,6,7-tetrahydro-4*H*-pyrrolo[3,2-c]pyridin-4-one (Compound **S10**) (Rt = 12.49 min, peak 2, 11.5 mg, 22% yield) was isolated as the second-eluting peak as a yellow solid. ^{1}H NMR (400 MHz, DMSO-d_6) δ = 11.54 (s, 1H), 8.43 (s, 1H), 8.25 (d, *J* = 5.2 Hz, 1H), 7.35 (d, *J* = 5.2 Hz, 1H), 6.73–6.59 (m, 5H), 6.16–6.10 (m, 1H), 5.66–5.39 (m, 1H), 4.09–3.84 (m, 2H), 3.46–3.42 (m, 2H), 3.25 (s, 3H), 2.90–2.87 (m, 3H), 2.40–2.28 (m, 1H), 2.15–2.04 (m, 1H), 1.68–1.66 (m, 1H), 0.93 (t, *J* = 5.2 Hz, 1H). *m/z* (ESI, +ve ion): 528.30 $(M + H)^+$. Absolute stereochemistry (*R* vs. *S*) was arbitrarily assigned.

Compound 44

2-(3-(((1*R*,2*R*,5*S*)-3-Acryloyl-3-azabicyclo[3.1.0]hexan-2-yl)ethynyl)pyridin-4-yl)-3-((3-chloro-2-methoxyphenyl)amino)-1,5,6,7-tetrahydro-4*H*-pyrrolo[3,2-c]pyridin-4-one.

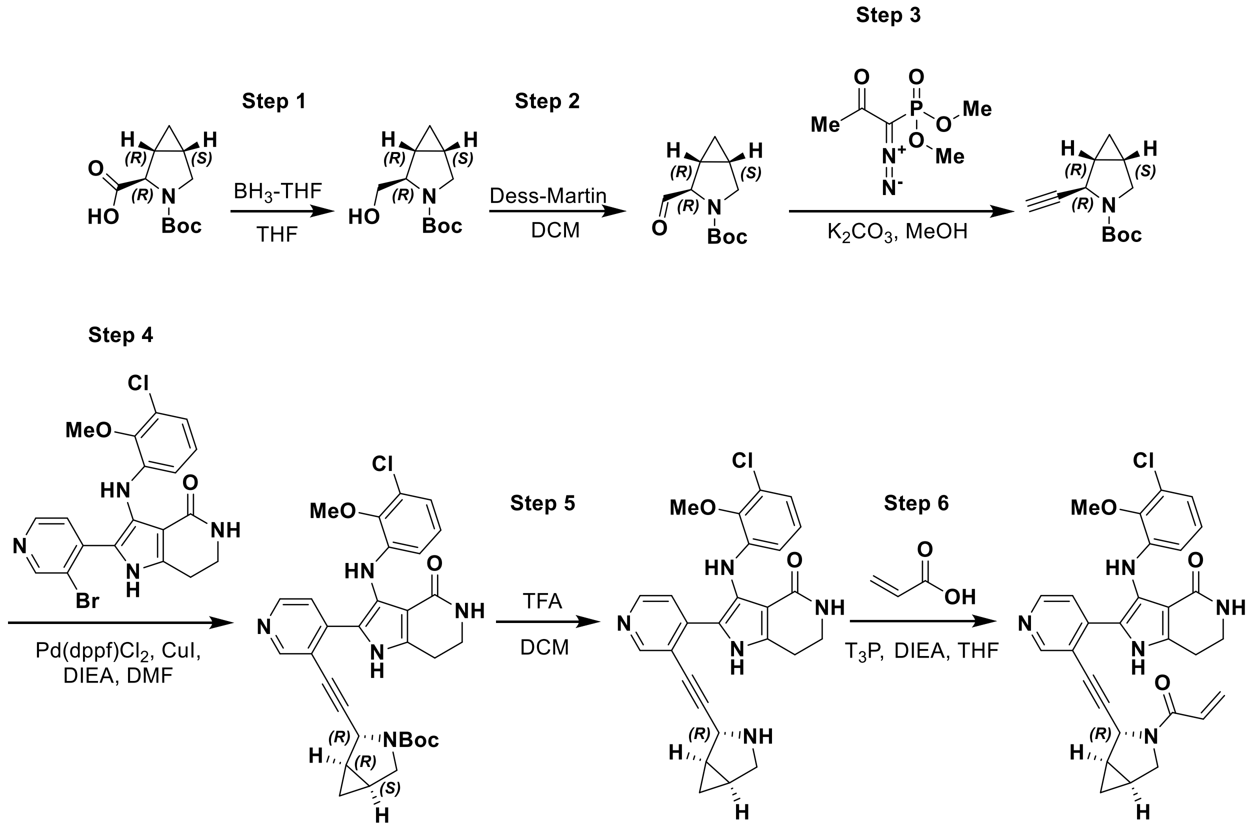

Compound 44

Step 1: *tert*-Butyl (1*R*,2*R*,5*S*)-2-(hydroxymethyl)-3-azabicyclo[3.1.0]hexane-3-carboxylate. To a stirred solution of (1*R*,2*R*,5*S*)-3-(*tert*-butoxycarbonyl)-3-azabicyclo[3.1.0]hexane-2-carboxylic acid (1.50 g, 6.60 mmol) in tetrahydrofuran (10.0 mL) was added BH_3-THF (13.2 mL, 13.2 mmol, 1 M in THF) dropwise at 0 °C. The resulting mixture was allowed to warm to RT. After 1 h, the reaction mixture was cooled to 0 °C and MeOH (10 mL) was introduced. The resulting mixture was concentrated under reduced pressure and purified by silica gel column chromatography, eluted with PE/EtOAc (3:1) to afford *tert*-butyl (1*R*,2*R*,5*S*)-2-(hydroxymethyl)-3-azabicyclo[3.1.0]hexane-3-carboxylate (1.30 g, 92% yield) as a light yellow oil. ^{1}H NMR (400 MHz, $CDCl_3$) δ = 4.05 (s, 1H), 3.83–3.56 (m, 2H), 3.42 (dd, *J* = 10.8, 4.0 Hz, 1H), 1.47 (s, 10H), 1.38–1.27 (m, 1H), 0.80–0.68 (m, 1H), 0.23–0.13 (m, 1H). *m/z* (ESI, +ve ion): 214.10 $(M + H)^+$.

Step 2: *tert*-Butyl (1*R*,2*R*,5*S*)-2-formyl-3-azabicyclo[3.1.0]hexane-3-carboxylate. To a stirred solution of *tert*-butyl (1*R*,2*R*,5*S*)-2-(hydroxymethyl)-3-azabicyclo[3.1.0]hexane-3-carboxylate (1.10 g, 5.15 mmol) in DCM (20.0 mL) was added Dess–Martin periodinane (2.63 mg, 6.19 mmol) in portions at 0 °C. The resulting mixture was allowed to warm to RT. After 1 h, the reaction mixture was cooled to 0 °C and MeOH (10 mL) was introduced. The residue was purified by silica gel column chromatography, eluted with PE/EtOAc (6:1) to afford *tert*-butyl (1*R*,2*R*,5*S*)-2-formyl-3-azabicyclo[3.1.0]hexane-3-carboxylate (770 mg, 71% yield). ^{1}H NMR (400 MHz, $CDCl_3$) δ = 9.63 (dd, *J* = 30.4, 1.5 Hz, 1H), 4.43–4.20 (m, 1H), 3.75–3.43 (m, 2H), 1.69–1.53 (m, 2H), 1.46 (d, *J* = 18.1 Hz, 9H), 0.94–0.75 (m, 1H), 0.43–0.30 (m, 1H). *m/z* (ESI, +ve ion): not observed.

Step 3: *tert*-Butyl (1*R*,2*R*,5*S*)-2-ethynyl-3-azabicyclo[3.1.0]hexane-3-carboxylate. To a stirred solution of *tert*-butyl (1*R*,2*R*,5*S*)-2-formyl-3-azabicyclo[3.1.0]hexane-3-carboxylate (770 mg, 3.64 mmol) in MeOH (10.0 mL) was added K_2CO_3 (1.00 g, 7.29 mmol) in portions at 0 °C. The resulting mixture was stirred for 30 min at 0 °C. Sat aq. potassium sodium tartrate was introduced at 0 °C. The resulting mixture was extracted with EtOAc (3 × 5 mL). The combined organic layers were washed with brine, dried over anhydrous Na_2SO_4, filted, and the filtrate was concentrated under reduced pressure. The residue was purified by silica gel column chromatography, eluted with PE/ EtOAc (15:1) to afford *tert*-butyl (1*R*,2*R*,5*S*)-2-ethynyl-3-

azabicyclo[3.1.0]hexane-3-carboxylate (450 mg, 60% yield) as a yellow oil. ^{1}H NMR (400 MHz, $CDCl_3$) δ = 4.51 (dd, *J* = 58.2, 2.1 Hz, 1H), 3.60 (dd, *J* = 22.0, 10.6 Hz, 1H), 3.50–3.42 (m, 1H), 2.29 (dd, *J* = 12.8, 2.1 Hz, 1H), 1.68–1.63 (m, 1H), 1.60–1.53 (m, 1H), 1.48 (d, *J* = 4.1 Hz, 9H), 0.70–0.65 (m, 1H), 0.29–0.23 (m, 1H). *m/z* (ESI, +ve ion): not observed.

Step 4: *tert*-Butyl (1*R*,2*R*,5*S*)-2-((4-(3-((3-chloro-2-methoxyphenyl)amino)-4-oxo-4,5,6,7-tetrahydro-1*H*-pyrrolo[3,2-c]pyridin-2-yl)pyridin-3-yl)ethynyl)-3-azabicyclo[3.1.0]hexane-3-carboxylate. To a stirred solution of 2-(3-bromopyridin-4-yl)-3-((3-chloro-2-methoxyphenyl)amino)-1,5,6,7-tetrahydro-4H-pyrrolo[3,2-c]pyridin-4-one (495 mg, 1.10 mmol) and *tert*-butyl (1*R*,2*R*,5*S*)-2-ethynyl-3-azabicyclo[3.1.0]hexane-3-carboxylate (458 mg, 2.20 mmol) in DMF (5.00 mL) was added $Pd(dppf)Cl_2 \cdot CH_2Cl_2$ (225 mg, 0.27 mmol), CuI (105 mg, 0.55 mmol), and *N*,*N*-diisopropylethylamine (962 µL, 5.53 mmol) at RT under a N_2 atmosphere. The resulting mixture was stirred for overnight at 50 °C and then concentrated under reduced pressure. The residue was purified by reversed-phase flash chromatography (Column: C18 silica gel; Mobile phase: MeCN in Water (0.1% FA), 10% to 50% gradient in 10 min) to afford esulted *tert*-butyl (1*R*,2*R*,5*S*)-2-((4-(3-((3-chloro-2-methoxyphenyl)amino)-4-oxo-4,5,6,7-tetrahydro-1*H*-pyrrolo[3,2-c]pyridin-2-yl)pyridin-3-yl)ethynyl)-3-azabicyclo[3.1.0]hexane-3-carboxylate (460 mg, 72% yield) as a red solid. *m/z* (ESI, +ve ion): 574.10 $(M + H)^+$.

Step 5: 2-(3-(((1*R*,2*R*,5*S*)-3-Azabicyclo[3.1.0]hexan-2-yl)ethynyl)pyridin-4-yl)-3-((3-chloro-2-methoxyphenyl)amino)-1,5,6,7-tetrahydro-4*H*-pyrrolo[3,2-c]pyridin-4-one. To a stirred solution of *tert*-butyl (1*R*,2*R*,5*S*)-2-((4-(3-((3-chloro-2-methoxyphenyl)amino)-4-oxo-4,5,6,7-tetrahydro-1*H*-pyrrolo[3,2-c]pyridin-2-yl)pyridin-3-yl)ethynyl)-3-azabicyclo[3.1.0]hexane-3-carboxylate (460 mg, 0.80 mmol) in DCM (5.00 mL) was added TFA (2.00 mL) dropwise at 0 °C. The resulting mixture was stirred for 1 h at RT. The resulting mixture was concentrated under reduced pressure. The residue was purified by reversed-phase flash chromatography (Column: C18 silica gel; Mobile phase: MeCN in Water (10 mmol/L NH_4HCO_3), 10% to 50% gradient in 10 min) to afford 2-(3-(((1*R*,2*R*,5*S*)-3-azabicyclo[3.1.0]hexan-2-yl)ethynyl)pyridin-4-yl)-3-((3-chloro-2-methoxyphenyl)amino)-1,5,6,7-tetrahydro-4*H*-pyrrolo[3,2-c]pyridin-4-one (500 mg, quantitative yield) as a red oil. *m/z* (ESI, +ve ion): 474.10 $(M + H)^+$.

Step 6: 2-(3-(((1*R*,2*R*,5*S*)-3-Acryloyl-3-azabicyclo[3.1.0]hexan-2-yl)ethynyl)pyridin-4-yl)-3-((3-chloro-2-methoxyphenyl)amino)-1,5,6,7-tetrahydro-4*H*-pyrrolo[3,2-c]pyridin-4-one (Compound 44). To a stirred mixture of 2-(3-(((1*R*,2*R*,5*S*)-3-azabicyclo[3.1.0]hexan-2-yl)ethynyl)pyridin-4-yl)-3-((3-chloro-2-methoxyphenyl)amino)-1,5,6,7-tetrahydro-4*H*-pyrrolo[3,2-c]pyridin-4-one (110 mg, 0.230 mmol) and prop-2-enoic acid (50.0 mg, 0.690 mmol), *N*,*N*-diisopropylethylamine (121 µL, 0.690 mmol) in THF (2.00 mL) was added propanephosphonic acid anhydride (829 µL, 0.690 mmol, 50 wt.% in EtOAc) dropwise at 0 °C. The resulting mixture was stirred for 1 h at RT and then cooled to 0 °C before sat. aq. $NaHCO_3$ was introduced. The resulting mixture was extracted with EtOAc (3 × 10 mL). The combined organic layers were washed with brine, dried over anhydrous Na_2SO_4, filtered, and concentrated under reduced pressure. The residue was purified by Prep-HPLC (Column: XBridge Prep OBD C18 Column, 30 × 150 mm, 5 µm; Mobile Phase A: Water (10 mmol/L NH_4HCO_3), Mobile Phase B: MeCN; Flow rate: 60 mL/min; Gradient: 32% B to 36% B in 7 min; Rt: 7.17 min) to afford 2-(3-(((1*R*,2*R*,5*S*)-3-Acryloyl-3-azabicyclo[3.1.0]hexan-2-yl)ethynyl)pyridin-4-yl)-3-((3-chloro-2-methoxyphenyl)amino)-1,5,6,7-tetrahydro-4*H*-pyrrolo[3,2-c]pyridin-4-one (27.7 mg, 22% yield) as a yellow solid. ^{1}H NMR (400 MHz, $CDCl_3$) δ = 10.85 (s, 1H), 8.61 (s, 1H), 8.18 (d, *J* = 5.6 Hz, 1H), 7.76 (s, 1H), 7.43 (d, *J* = 5.6 Hz, 1H), 6.74 (dd, *J* = 8.1, 1.5 Hz, 1H), 6.63 (t, *J* = 8.1 Hz, 1H), 6.52–6.34 (m, 2H), 6.25 (dd, *J* = 8.1, 1.5 Hz, 1H), 5.78 (dd, *J* = 8.6, 3.6 Hz, 1H), 5.27 (d, *J* = 2.6 Hz, 1H), 5.05 (s, 1H), 4.10 (s, 3H), 3.94 (dd, *J* = 9.8, 3.9 Hz, 1H), 3.84 (d, *J* = 9.9 Hz, 1H), 3.64–3.60 (m, 2H), 3.31–3.27 (m, 2H), 1.95–1.90 (m, 2H), 0.96–0.95 (m, 1H), 0.41–0.39 (m, 1H). *m/z* (ESI, +ve ion): 595.15 $(M + H)^+$.

Compound 45

2-(3-(((1*S*,3*R*,5*S*)-2-Acryloyl-2-azabicyclo[3.1.0]hexan-3-yl)ethynyl)pyridin-4-yl)-3-((3-chloro-2-methoxyphenyl)amino)-1,5,6,7-tetrahydro-4*H*-pyrrolo[3,2-c]pyridin-4-one was prepared according to the procedure described for Compound **38**, using *tert*-butyl (1*S*,3*R*,5*S*)-3-formyl-2-azabicyclo[3.1.0]hexane-2-carboxylate in Step 1 (92% yield). Next, Step 2 (98% yield), Step 3 and Step 4 afforded the title compound by Prep-HPLC (Column: XBridge Prep C18 OBD Column, 30 × 100 mm, 5µm; Mobile Phase A: Water (10 mmol/L NH_4HCO_3), Mobile Phase B: MeCN; Flow rate: 60 mL/min; Gradient: 28% B to 50% B in 9 min) (10.8 mg, 10% yield over two steps) as a yellow solid. ^{1}H NMR (400 MHz, $CDCl_3$) δ = 11.00 (s, 1H), 8.59 (s, 1H), 8.16 (s, 1H), 7.74 (s, 1H), 7.44 (s, 1H), 6.84–6.65 (m, 2H), 6.60 (t, *J* = 8.1 Hz, 1H), 6.48–6.33 (m, 1H), 6.24–6.14 (m, 1H), 5.88–5.69 (m, 1H), 5.22 (s, 1H), 4.89–4.73 (m, 1H), 4.07 (s, 3H), 3.67–3.52 (m, 3H), 3.30–3.20 (m, 2H), 2.66–2.45 (m, 2H), 2.06–1.95 (m, 1H), 1.13–1.01 (m, 1H), 0.70–0.60 (m, 1H). *m/z* (ESI, +ve ion): 527.90 $(M + H)^+$.

Compound 46

2-(3-((2-Acryloyl-2-azabicyclo[2.1.1]hexan-1-yl)ethynyl)pyridin-4-yl)-3-((3-chloro-2-methoxyphenyl)amino)-1,5,6,7-tetrahydro-4*H*-pyrrolo[3,2-c]pyridin-4-one was prepared according to the procedure described for Compound **38**, using *tert*-butyl 1-formyl-2-azabicyclo[2.1.1]hexane-2-carboxylate in Step 2 (68% yield). Next, Step 3 (93% yield) and Step 4 afforded the title compound (25.1 mg, 20% yield) after tritration with MeCN as a yellow solid. ^{1}H NMR (300 MHz, $CDCl_3$) δ = 11.53 (s, 1H), 8.61 (s, 1H), 8.14 (d, *J* = 5.7 Hz, 1H), 7.74 (s, 1H), 7.42 (d, *J* = 5.7 Hz, 1H), 6.76–6.67 (m, 1H), 6.65–6.53 (m, 1H), 6.53–6.37 (m, 2H), 6.29–6.18 (m, 1H), 5.86–5.75 (m, 1H), 5.24 (s, 1H), 4.07 (s, 3H), 3.68 (s, 2H), 3.62–3.51 (m, 2H), 3.07 (t, *J* = 6.8 Hz, 2H), 2.96 (t, *J* = 3.2 Hz, 1H), 2.46–2.32 (m, 2H), 2.00–1.92 (m, 2H). *m/z* (ESI, +ve ion): 528.05 $(M + H)^+$.

Compound 47

(*R*)-2-(3-((1-Acryloyl-2-methylpyrrolidin-2-yl)ethynyl)pyridin-4-yl)-3-((3-chloro-2-methoxyphenyl)amino)-1,5,6,7-tetrahydro-4*H*-pyrrolo[3,2-c]pyridin-4-one was prepared according to the procedure described for Compound **38**, using *tert*-butyl (2*R*)-2-formyl-2-methylpyrrolidine-1-carboxylate in Step 1 (87% yield). Next, Step 2 (92% yield), Step 3 and Step 4 afforded the title compound (17.0 mg, 15% yield over two steps) after Prep-HPLC (Column: XBridge Prep C18 OBD Column, 30 × 100 mm, 5 μm; Mobile Phase A: Water (10 mmol/L NH_4HCO_3), Mobile Phase B: MeCN; Flow rate: 60 mL/min; Gradient: 35% B to 55% B in 10 min) as a yellow solid. ^{1}H NMR (400 MHz, DMSO-d_6) δ = 11.35 (s, 1H), 8.50 (s, 1H), 8.20 (d, *J* = 5.2 Hz, 2H), 7.46–7.29 (m, 2H), 6.82–6.54 (m, 4H), 6.22–6.10 (m, 2H), 5.65 (d, *J* = 9.2 Hz, 1H), 3.89 (s, 3H), 3.68 (d, 2H), 3.47–3.40 (m, 2H), 3.10–3.02 (m, 2H), 2.44–2.35 (m, 1H), 2.19–2.09 (m, 1H), 2.05–1.93 (m, 2H), 1.69 (s, 3H). *m/z* (ESI, +ve ion): 530.30 $(M + H)^+$.

Compound 48

(*R*)-3-((3-Chloro-2-methoxyphenyl)amino)-2-(3-((1-(2-fluoroacryloyl)pyrrolidin-2-yl)ethynyl)pyridin-4-yl)-1,5,6,7-tetrahydro-4*H*-pyrrolo[3,2-c]pyridin-4-one was prepared according to the procedure described for Compound **38**, using 2-fluoroprop-2-enoic acid as the acrylamide equivalent, *N*,*N*-diisopropylethylamine as the base, and propanephosphonic acid anhydride (T_3P) as the amide coupling reagent, in THF at 70 °C for 10 h in Step 4 to afford the title compound (12.2 mg, 13% yield) as a light yellow solid after Prep-HPLC (Column: XBridge Prep C18 OBD Column, 30 × 100 mm, 5μm; Mobile Phase A: Water (10 mmol/L NH_4HCO_3), Mobile Phase B: MeCN; Flow rate: 60 mL/min; Gradient: 35% B to 62% B in 7 min; Rt: 6.32 min). ^{1}H NMR (400 MHz, DMSO-d_6) δ = 11.50–11.20 (m, 1H), 8.56 (s, 1H), 8.40–8.20 (m, 1H), 7.47 (s, 1H), 7.38–7.23 (m, 1H), 7.20–7.05 (m, 1H), 6.71–6.56 (m, 2H), 6.20–6.00 (m, 1H), 5.62–5.04 (m, 2H), 4.95 (d, *J* = 6.8 Hz, 1H), 3.84 (d, *J* = 8.9 Hz, 3H), 3.76–3.64 (m, 1H), 3.42 (dt, *J* = 6.8, 3.4 Hz, 3H), 3.03–2.74 (m, 2H), 2.29–1.86 (m, 4H). *m/z* (ESI, +ve ion): 534.05 $(M + H)^+$.

Compound 49

(*R*)-2-(3-((1-(but-2-ynoyl)pyrrolidin-2-yl)ethynyl)pyridin-4-yl)-3-((3-chloro-2-methoxyphenyl)amino)-1,5,6,7-tetrahydro-4*H*-pyrrolo[3,2-c]pyridin-4-one was prepared according to the procedure described for Compound **38**, using 2-butynoic acid as the acrylamide equivalent, *N*,*N*-diisopropylethylamine as the base, and propanephosphonic acid anhydride (T_3P) as the amide coupling reagent, in THF at RT for 1 h in Step 4 to afford the title compound (56.0 mg, 72% yield) as a yellow solid after Prep-HPLC (Column: Xselect CSH C18 OBD Column 30 × 150 mm, 5 μm; Mobile Phase A: Water (0.05%TFA), Mobile Phase B: MeCN; Flow rate: 60 mL/min; Gradient: 13% B to 43% B in 7 min; Rt: 6.07 min). ^{1}H NMR (400 MHz, DMSO-d_6) δ = 10.96 (s, 1H), 8.49 (d, *J* = 11.2 Hz, 1H), 8.25 (s, 1H), 7.28 (q, *J* = 5.6 Hz, 2H), 6.61 (d, *J* = 22.5 Hz, 3H), 6.14 (m, 1H), 4.85 (s, 1H), 3.85 (d, *J* = 12.3 Hz, 3H), 3.64 (s, 1H), 3.44 (q, *J* = 7.1 Hz, 3H), 2.78 (s, 2H), 2.26 (d, *J* = 26.0 Hz, 1H), 2.13–2.00 (m, 2H), 1.95 (s, 4H). *m/z* (ESI, +ve ion): 528.20 $(M + H)^+$.

Compound 50

(*R*,*E*)-3-((3-Chloro-2-methoxyphenyl)amino)-2-(3-((1-(4-(dimethylamino)but-2-enoyl)pyrrolidin-2-yl)ethynyl)pyridin-4-yl)-1,5,6,7-tetrahydro-4*H*-pyrrolo[3,2-c]pyridin-4-one was prepared according to the procedure described for Compound **38**, using (2*E*)-4-(dimethylamino) but-2-enoyl chloride in Step 4 to afford the title compound (31.7 mg, 25% yield) as a light brown solid after Prep-HPLC (Column: Xselect CSH C18 OBD Column 30 × 150 mm, 5 μm; Mobile Phase A: Water (0.1% FA), Mobile Phase

B: MeCN; Flow rate: 60 mL/min; Gradient: 9% B to 25% B in 10 min; Rt: 9.3 min). ^{1}H NMR (300 MHz, $CDCl_3$) δ = 11.09 (s, 1H), 8.54 (s, 1H), 8.17 (t, *J* = 16.2 Hz, 2H), 7.69 (s, 1H), 7.42 (d, *J* = 5.5 Hz, 1H), 6.93–6.73 (m, 1H), 6.71–6.51 (m, 1H), 6.60 (t, *J* = 8.1 Hz, 1H), 6.47 (d, *J* = 15.2 Hz, 1H), 6.25–6.08 (m, 1H), 5.58 (s, 1H), 4.89 (t, *J* = 5.9 Hz, 1H), 4.06 (s, 3H), 3.90–3.70 (m, 1H), 3.75–3.55 (m, 4H), 3.40 (d, *J* = 6.5 Hz, 5H), 3.24 (t, *J* = 6.8 Hz, 2H), 2.49 (s, 6H), 2.39–2.21 (m, 3H), 2.15–2.05 (m, 1H). *m/z* (ESI, +ve ion): 573.35 $(M + H)^+$.

Compound 51

(*R*,*E*)-3-((3-chloro-2-methoxyphenyl)amino)-2-(3-((1-(4-(pyrrolidin-1-yl)but-2-enoyl)pyrrolidin-2-yl)ethynyl)pyridin-4-yl)-1,5,6,7-tetrahydro-4*H*-pyrrolo[3,2-c]pyridin-4-one.

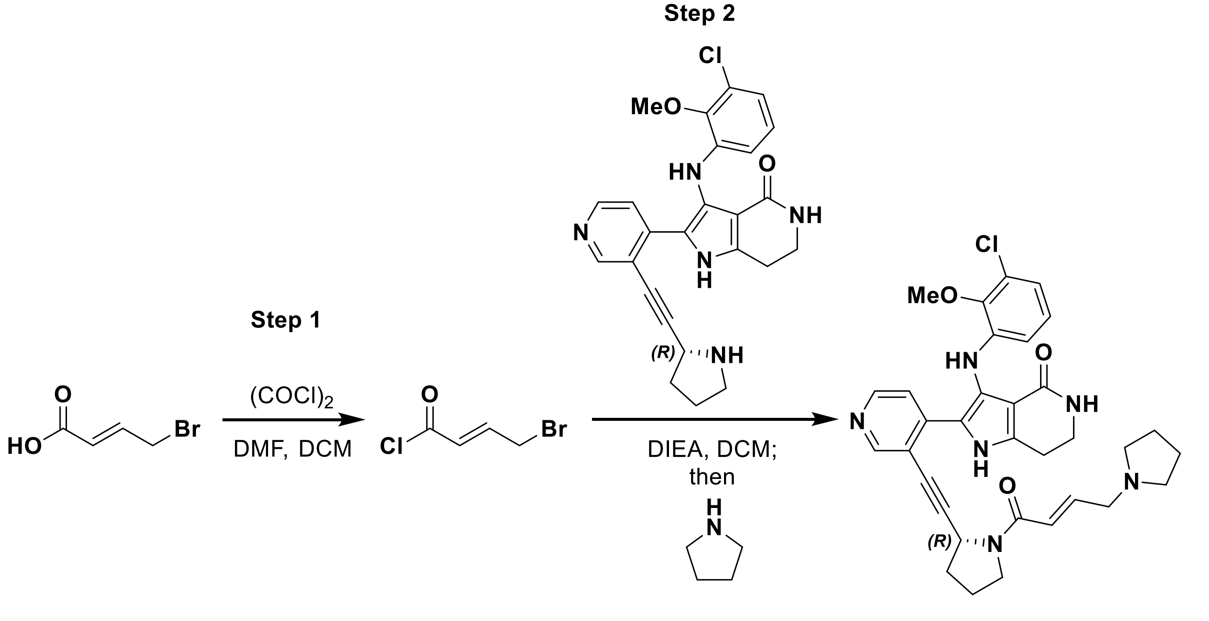

Compound 51

Step 1: (*E*)-4-bromobut-2-enoyl chloride. To a stirred solution of (*E*)-4-bromobut-2-enoic acid (200 mg, 1.21 mmol) and DMF (0.05 mL) in DCM (10 mL) was added oxalyl chloride (207 µL, 2.42 mmol) dropwise at 0 °C under a N_2 atmosphere. The resulting mixture was stirred for 1 h at 0 °C under nitrogen atmosphere. The resulting mixture was concentrated under reduced pressure to afford (*E*)-4-bromobut-2-enoyl chloride (200 mg, crude) as a yellow oil. The crude product was used in next step directly without purification.

Step 2: (*R*,*E*)-3-((3-chloro-2-methoxyphenyl)amino)-2-(3-((1-(4-(pyrrolidin-1-yl)but-2-enoyl)pyrrolidin-2-yl)ethynyl)pyridin-4-yl)-1,5,6,7-tetrahydro-4*H*-pyrrolo[3,2-c]pyridin-4-one (Compound 51). To a stirred solution of (*R*)-3-((3-chloro-2-methoxyphenyl)amino)-2-(3-(pyrrolidin-2-ylethynyl)pyridin-4-yl)-1,5,6,7-tetrahydro-4*H*-pyrrolo[3,2-c]pyridin-4-one (40.0 mg, 0.080 mmol) and *N*,*N*-diisopropylethylamine (46 µL, 0.26 mmol) in DCM (2.5 mL) was added (*E*)-4-bromobut-2-enoyl chloride (19.0 mg, 0.100 mmol) at 0 °C under a N_2 atmosphere. The resulting mixture was stirred for 0.5 h at 0 °C. Next, pyrrolidine (22.3 µL, 0.26 mmol) was added dropwise at 0 °C. The resulting mixture stirred at RT. After 48 h, brine (10 mL) was introduced and the resultant mixture was extracted with DCM:MeOH (10:1, 3 × 10 mL). The combined organic layers were dried over anhydrous Na_2SO_4, filtered, and concentrated under reduced pressure. The residue was purified by Prep-HPLC (Column: Xselect CSH C18 OBD Column 30 × 150 mm, 5 µm; Mobile Phase A: Water (0.1% FA), Mobile Phase B: MeCN; Flow rate: 60 mL/min; Gradient: 10% B to 28% B in 8 min; Rt: 8.00 min) to afford (*R*,*E*)-3-((3-chloro-2-methoxyphenyl)amino)-2-(3-((1-(4-(pyrrolidin-1-yl)but-2-enoyl)pyrrolidin-2-yl)ethynyl)pyridin-4-yl)-1,5,6,7-tetrahydro-4*H*-pyrrolo[3,2-c]pyridin-4-one (12.6 mg, 24% yield) as a yellow solid. ^{1}H NMR (400 MHz, $CDCl_3$) δ = 11.12 (s, 1H), 8.56 (s, 1H), 8.39 (s, 1H), 8.17 (d, *J* = 5.6 Hz, 1H), 7.72

(s, 1H), 7.44 (d, *J* = 5.6 Hz, 1H), 6.97 (m, *J* = 15.2, 6.4 Hz, 1H), 6.73 (m, *J* = 8.0, 1.5 Hz, 1H), 6.62 (t, *J* = 8.1 Hz, 1H), 6.52 (m, *J* = 15.0, 1.5 Hz, 1H), 6.24 (m, *J* = 8.2, 1.5 Hz, 1H), 5.52 (s, 1H), 4.91 (m, *J* = 7.3, 4.6 Hz, 1H), 4.09 (s, 3H), 3.82 (m, *J* = 10.0, 6.4 Hz, 1H), 3.73–3.54 (m, 5H), 3.27 (t, *J* = 6.5 Hz, 2H), 2.91 (d, *J* = 6.4 Hz, 4H), 2.40–2.21 (m, 3H), 2.17–2.05 (m, 1H), 1.97 (d, *J* = 3.7 Hz, 4H). *m/z* (ESI, +ve ion): 599.55 $(M + H)^+$.

Compound 52

(*R,E*)-3-((3-chloro-2-methoxyphenyl)amino)-2-(3-((1-(4-morpholinobut-2-enoyl)pyrrolidin-2-yl)ethynyl)pyridin-4-yl)-1,5,6,7-tetrahydro-4*H*-pyrrolo[3,2-c]pyridin-4-one was prepared according to the procedure described for Compound **51**, using morpholine in the second part of Step 2 as the nucleophile to afford the title compound (16.4 mg, 31% yield) as a yellow solid after Prep-HPLC (Column: Xselect CSH C18 OBD Column 30 × 150 mm, 5 μm; Mobile Phase A: Water (0.1% FA), Mobile Phase B: MeCN; Flow rate: 60 mL/min; Gradient: 10% B to 27% B in 8 min; Rt: 8.00 min). ^{1}H NMR (400 MHz, $CDCl_3$) δ = 11.14 (s, 1H), 8.56 (s, 1H), 8.22–8.13 (m, 1H), 7.72 (s, 1H), 7.44 (d, *J* = 5.6 Hz, 1H), 6.95 (m, *J* = 15.1, 6.3 Hz, 1H), 6.73 (m, *J* = 8.1, 1.5 Hz, 1H), 6.62 (t, *J* = 8.1 Hz, 1H), 6.40 (d, *J* = 15.1 Hz, 1H), 6.23 (m, *J* = 8.1, 1.5 Hz, 1H), 5.64 (t, *J* = 2.5 Hz, 1H), 4.92 (m, *J* = 7.2, 4.6 Hz, 1H), 4.09 (s, 3H), 3.89–3.52 (m, 9H), 3.34–3.17 (m, 4H), 2.55 (d, *J* = 4.8 Hz, 3H), 2.44–2.21 (m, 3H), 2.20–2.04 (m, 1H). *m/z* (ESI, +ve ion): 615.2 $(M + H)^+$.

Additional data for compounds with arbitrarily defined stereochemistry

Table S1. Antiproliferative Activity and Selectivity for Additional Isomers Included in Lactam Substituent Optimization of Rat and Dog PK

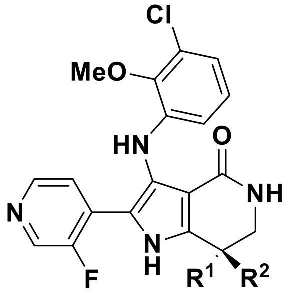

Cmpd	R¹	R²	EGFR ex20ins IC_{50} (nM) (ASV | SVD)[a]	Fold Selectivity vs. WT (ASV | SVD)[b]
**23**	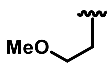	H	5.1 | 6.5	18 | 14
**22**	H	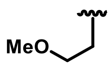	49 | 50	10 | 10
**27**[c]	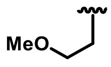	Me	18.7 | 17.9	9.0 | 9.4
**S2**[c]	Me	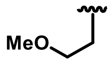	24.7 | 22.4	7.4 | 8.2

**28**[d]	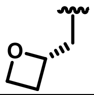	H	30.4 | 27.2	7.0 | 7.8
**S3**[d]	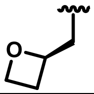	H	39.5 | 44.2	12.0 | 10.7
**S4**[d]	H	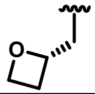	135.7 | 133.1	12.2 | 12.9
**S5**[d]	H	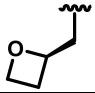	89.7 | 88.4	13.4 | 13.6
**29**[e]	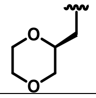	H	6.0 | 7.6	21 | 17
**30**[e]	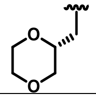	H	3.3 | 5.0	17 | 11
**S6**[e]	H	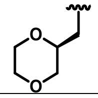	33.0 | 43.6	22.9 | 17.3
**S7**[e]	H	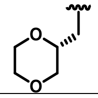	46.8 | 49.5	27.2 | 25.8

[a]Antiproliferative activity in Ba/F3 via CTG. [b]Fold selectivity against Ba/F3 EGFR WT. [c]The more potent lactam isomer was assigned the same stereochemistry as Compound **23** by analogy to Compound **23** (known absolute configuration based on X-ray crystallography) and Compound **22**. [d]The more potent lactam isomer was assigned the same stereochemistry as Compound **23**, the oxetane stereochemistry was arbitrarily assigned. [e]The more potent lactam isomer was assigned the same stereochemistry as Compound **32** by analogy to Compound **32** (known absolute configuration based on single crystal X-ray structure) and Compound **S9**. The dioxane stereochemistry was based on starting material identity. All data represents $n \geq 2$.

Table S2. Antiproliferative Activity and Selectivity for Additional Isomers Included in Hinge Binder Group Optimization

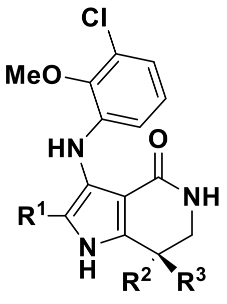

Cmpd	R[1]	R[2]	R[3]	EGFR ex20ins IC_{50} (nM) (ASV | SVD)[a]	Fold Selectivity vs. WT (ASV | SVD)[b]
**30**[c]	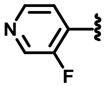	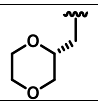	H	3.3 | 5.0	17 | 13

**S7**[c]	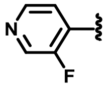	H	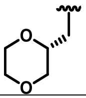	46.8 | 49.5	27.2 | 25.8
**31**[c]	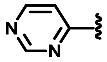	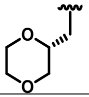	H	8.3 |7.8	27 | 28
**S8**[c]	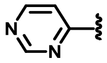	H	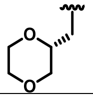	145 | 122	16.3 |19.3
**32**	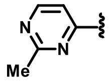	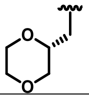	H	4.2 | 6.4	29 | 19
**S9**	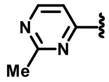	H	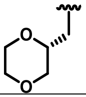	50.8 | 60.3	26.4 | 22.3

[a]Antiproliferative activity in Ba/F3 via CTG. [b]Fold selectivity against Ba/F3 EGFR WT. [c]The more potent lactam isomer was assigned the same stereochemistry as Compound **32** by analogy to Compound **32** (known absolute configuration based on single crystal X-ray structure) and Compound **S9**. All data represents $n \geq 2$.

Biochemical activity and selectivity data for compounds 38 & 53 (STX-721)

Table S3. Biochemical k_{inact}/K_I Activity and Selectivity via Chelation-Enhanced Fluorescence (ChEF) Detection

Cmpd	k_{inact}, s^{-1}, WT	k_{inact}, s^{-1}, insNPG	K_I (μM), 1mM ATP, WT	K_I (μM), 1mM ATP, insNPG	k_{inact}/K_I ($M^{-1}s^{-1}$), 1mM ATP, WT	k_{inact}/K_I ($M^{-1}s^{-1}$), 1mM ATP, insNPG
**38**	2.88E-04	2.71E-03	0.187	0.0267	1.54E+03	1.02E+05
**53**	4.68E-04	3.54E-03	0.144	0.0569	3.24E+03	6.22E+04

Comprehensive Ba/F3, NCI-H2073, and NCI-H1975 cell antiproliferative activity and selectivity data

Table S4. Ba/F3 EGFR WT Antiproliferative Activity

Cmpd	Ba/F3_EGFR [WT] CTGlo GMean IC50 (nM)	Ba/F3_EGFR [WT] CTGlo GStd Dev IC50 (nM)	Ba/F3_EGFR [WT] CTGlo IC50 Count	Cmpd	Ba/F3_EGFR [WT] CTGlo GMean IC50 (nM)	Ba/F3_EGFR [WT] CTGlo GStd Dev IC50 (nM)	Ba/F3_EGFR [WT] CTGlo IC50 Count
**1**	131.053	5.473	21	**33**	198.949	1.38	20
**2**	955.316	1.821	5	**34**	207.654	1.513	23
**3**	3205.784	2.645	11	**35**	164.766	1.816	16
**4**	3604.629	1.621	8	**36**	38.002	1.28	5
**5**	3976.576	1.119	2	**37**	26.803	1.361	5
**6**	>10000.000	1	2	**38**	319.904	1.514	22

**7**	4021.366	1.554	9	**39**	407.682	1.631	23
**8**	657.693	1.806	27	**40**	28.058	1.444	5
**9**	340.58	1.399	2	**41**	141.547	1.562	5
**10**	894.739	1.665	3	**42**	799.486	1.203	5
**11**	516.466	1.382	3	**43**	>10000.000	1	5
**12**	8998.664	1.101	3	**44**	67.503	1.056	3
**13**	1304.184	1.781	4	**45**	227.247	1.375	8
**14**	3648.134	1.279	3	**46**	411.413	3.065	4
**15**	4377.935	1.341	2	**47**	474.774	2.595	14
**16**	223.369	2.223	3	**48**	1164.058	1.294	6
**17**	983.147	1.842	3	**49**	1041.96	1.402	5
**18**	1326.947	1.232	2	**50**	1027.404	1.417	7
**19**	4891.775	1.136	3	**51**	1058.639	1.47	5
**20**	1355.703	1.205	6	**52**	4873.279	1.136	5
**21**	1177.282	1.335	6	**53**	127.365	1.439	140
**22**	505.334	1.377	4	**S1**	187.282	2.465	16
**23**	93.892	1.568	23	**S2**	182.868	1.233	4
**24**	1476.383	1.433	6	**S3**	473.233	1.236	9
**25**	411.215	1.409	6	**S4**	1712.759	1.383	9
**26**	1704.449	1.646	6	**S5**	1198.985	1.326	9
**27**	168.716	1.232	5	**S6**	754.154	1.321	9
**28**	211.287	1.364	9	**S7**	1275.19	1.426	11
**29**	127.357	1.345	25	**S8**	2364.201	1.448	4
**30**	56.398	1.312	31	**S9**	1342.923	1.645	18
**31**	219.877	1.401	12	**S10**	4769.139	1.224	5
**32**	119.7	1.608	49				

Table S5. Ba/F3 EGFR Exon 20 Insertion Mutant Antiproliferative Activity

Cmpd	Ba/F3_EGFR [V769_D770 InsASV] CTGlo GMean IC50 (nM)	Ba/F3_EGFR [V769_D770 InsASV] CTGlo GStd Dev IC50 (nM)	Ba/F3_EGFR [V769_D770 InsASV] CTGlo IC50 Count	Ba/F3_EGFR [D770_N771 InsSVD] CTGlo GMean IC50 (nM)	Ba/F3_EGFR [D770_N771 InsSVD] CTGlo GStd Dev IC50 (nM)	Ba/F3_EGFR [D770_N771 InsSVD] CTGlo IC50 Count
**1**	6.84	1.186	20	10.186	1.18	14
**2**	139.498	1.056	3	163.783	1.167	2
**3**	374.097	1.026	3	132.87	1.122	3
**4**	179.447	1.165	2	135.35	1.147	2

**5**	572.815	1.137	2	548.744	1.108	2
**6**	2077.388	1.464	2	1536.887	1.705	2
**7**	306.11	1.275	2	285.1	1.238	2
**8**	45.495	1.366	17	46.059	1.524	17
**9**	428.827	1.053	2	434.527	1.232	2
**10**	479.355	1.336	2	458.422	1.151	2
**11**	131.822	1.675	2	87.245	1.839	2
**12**	8451.179	No Summarized Data	2	6539	1.124	2
**13**	140.711	1.067	3	118.272	1.199	2
**14**	365.512	1.447	2	365.157	1.344	2
**15**	862.366	1.085	3	1116.551	1.153	2
**16**	95.092	1.004	2	91.096	1.066	2
**17**	162.928	1.15	2	222.549	1.345	2
**18**	90.332	1.031	3	93.012	1.208	2
**19**	1328.31	1.085	2	1198.352	1.184	2
**20**	221.24	1.075	2	108.919	1.052	2
**21**	113.25	1.214	2	69.861	1.025	2
**22**	48.597	1.181	3	49.515	1.101	3
**23**	5.113	1.209	15	6.514	1.25	15
**24**	151.447	1.081	2	67.091	1.08	2
**25**	74.681	1.066	2	35.049	1.027	2
**26**	130.657	1.337	6	138.984	1.399	6
**27**	18.672	1.062	5	17.856	1.099	5
**28**	30.395	1.203	8	27.172	1.237	8
**29**	5.957	1.295	22	7.615	1.29	22
**30**	3.256	1.459	28	4.987	1.257	28
**31**	8.284	1.221	10	7.847	1.235	10
**32**	4.176	1.492	43	6.446	1.373	43
**33**	11.449	1.411	20	13.505	1.443	20
**34**	14.927	1.283	21	12.252	1.46	21
**35**	11.347	1.412	9	7.276	1.445	9
**36**	5.458	1.282	5	8.682	1.352	5
**37**	3.312	1.564	5	4.111	1.821	5
**38**	17.608	1.593	19	19.228	1.297	19
**39**	17.057	1.394	16	16.497	1.425	19
**40**	5.82	1.198	3	12.84	1.356	3
**41**	12.507	1.472	3	18.306	1.148	3

**42**	45.778	1.272	3	49.457	1.31	3
**43**	6347.955	1.503	3	8096.139	No Summarized Data	3
**44**	21.925	1.043	3	13.947	1.003	3
**45**	11.28	1.351	8	16.167	1.182	8
**46**	14.582	1.42	4	22.647	1.441	4
**47**	24.201	2.8	14	27.14	2.972	14
**48**	99.585	1.717	6	152.261	1.214	6
**49**	73.209	1.448	5	68.473	1.552	5
**50**	49.466	1.343	7	46.601	1.372	7
**51**	68.99	1.348	5	57.1	1.541	5
**52**	603.154	1.332	5	588.474	1.422	5
**53**	5.376	1.414	141	5.812	1.477	137
**S1**	13.306	1.584	13	13.633	2.019	10
**S2**	24.712	1.19	4	22.407	1.044	4
**S3**	39.5	1.635	8	44.178	1.785	8
**S4**	135.683	1.139	8	133.148	1.126	8
**S5**	89.651	1.063	8	88.439	1.118	8
**S6**	32.996	1.287	6	43.623	1.524	6
**S7**	46.803	1.372	10	49.486	1.601	10
**S8**	144.789	1.034	4	122.356	1.24	4
**S9**	50.79	1.531	14	60.297	1.505	14
**S10**	1217.649	1.088	3	1462.636	1.268	3

Table S6. Ba/F3 EGFR Exon 20 Insertion Mutant vs. EGFR WT Antiproliferative Selectivity

Cmpd	EGFR ex20insASV Fold Selectivity vs. WT	EGFR ex20insASV Fold Selectivity vs. WT Upper Confidence Interval	EGFR ex20insASV Fold Selectivity vs. WT Lower Confidence Interval	EGFR ex20insSVD Fold Selectivity vs. WT	EGFR ex20insSVD Fold Selectivity vs. WT Upper Confidence Interval	EGFR ex20insSVD Fold Selectivity vs. WT Lower Confidence Interval
**1**	19.161	39.795	9.226	12.866	26.756	6.187
**2**	6.848	11.624	4.035	5.833	10.286	3.308
**3**	8.569	15.238	4.819	24.127	43.498	13.383
**4**	20.087	29.853	13.516	26.632	39.125	18.128
**5**	6.942	8.792	5.481	7.247	8.95	5.868
**6**	4.814	8.161	2.84	6.507	13.63	3.106

**7**	13.137	20.468	8.432	14.105	21.32	9.332
**8**	14.457	18.895	11.06	14.279	19.267	10.583
**9**	0.794	1.272	0.496	0.784	1.355	0.453
**10**	1.867	3.77	0.924	1.952	3.589	1.061
**11**	3.918	8.747	1.755	5.92	14.854	2.359
**12**	1.065	1.187	0.955	1.376	1.673	1.132
**13**	9.269	16.399	5.238	11.027	20.485	5.936
**14**	9.981	17.875	5.573	9.991	16.4	6.086
**15**	5.077	7.7	3.347	3.921	6.16	2.496
**16**	2.349	5.802	0.951	2.452	6.083	0.988
**17**	6.034	12.368	2.944	4.418	9.871	1.977
**18**	14.69	19.665	10.973	14.266	21.075	9.657
**19**	3.683	4.425	3.065	4.082	5.376	3.099
**20**	6.128	7.331	5.122	12.447	14.672	10.56
**21**	10.395	14.822	7.291	16.852	21.283	13.343
**22**	10.399	14.995	7.211	10.206	14.226	7.322
**23**	18.363	22.592	14.926	14.414	17.884	11.618
**24**	9.749	13.26	7.167	22.006	29.919	16.186
**25**	5.506	7.344	4.128	11.732	15.47	8.898
**26**	13.045	20.696	8.223	12.264	19.832	7.583
**27**	9.036	10.927	7.472	9.449	11.545	7.733
**28**	6.951	8.836	5.469	7.776	9.991	6.052
**29**	21.379	25.051	18.246	16.724	19.576	14.287
**30**	17.32	20.517	14.62	11.31	12.85	9.954
**31**	26.541	33.316	21.144	28.021	35.313	22.235
**32**	28.664	34.275	23.972	18.57	21.862	15.774
**33**	17.377	21.364	14.134	14.732	18.246	11.894
**34**	13.911	16.988	11.392	16.949	21.417	13.413
**35**	14.521	21.003	10.039	22.644	33.065	15.508
**36**	6.963	9.465	5.122	4.377	6.161	3.11
**37**	8.092	13.027	5.026	6.519	11.771	3.61
**38**	18.168	23.842	13.844	16.638	20.504	13.5
**39**	23.901	30.93	18.47	24.712	31.914	19.135
**40**	4.821	7.06	3.292	2.185	3.502	1.364
**41**	11.317	20.351	6.293	7.732	11.78	5.076
**42**	17.465	23.972	12.723	16.165	22.845	11.439
**43**	1.575	2.499	0.993	1.235	1.235	1.235

**44**	3.079	3.326	2.85	4.84	5.146	4.552
**45**	20.145	27.29	14.871	14.056	18.036	10.955
**46**	28.214	89.118	8.932	18.166	57.625	5.727
**47**	19.618	40.919	9.406	17.493	37.342	8.195
**48**	11.689	18.873	7.24	7.645	9.896	5.906
**49**	14.233	22.09	9.17	15.217	24.735	9.362
**50**	20.77	29.133	14.807	22.047	31.248	15.555
**51**	15.345	23.522	10.01	18.54	30.799	11.16
**52**	8.08	10.635	6.138	8.281	11.499	5.964
**53**	23.692	25.744	21.804	21.915	23.95	20.052
**S1**	14.075	23.39	8.469	13.738	25.549	7.386
**S2**	7.4	9.661	5.668	8.161	10.061	6.62
**S3**	11.981	17.303	8.295	10.712	16.378	7.006
**S4**	12.623	15.892	10.027	12.864	16.148	10.247
**S5**	13.374	16.157	11.07	13.557	16.555	11.102
**S6**	22.856	29.991	17.418	17.288	25.361	11.785
**S7**	27.246	36.31	20.444	25.769	36.91	17.991
**S8**	16.329	23.497	11.347	19.322	29.393	12.702
**S9**	26.441	36.429	19.191	22.272	30.497	16.265
**S10**	3.917	4.79	3.203	3.261	4.499	2.363

Table S7. Compound 23 Ba/F3 Antiproliferative Activity and WT Selectivity Panel

Ba/F3 EGFR [WT] CTGlo GMean IC50 (nM)	93.892
Ba/F3 EGFR [WT] CTGlo GStd Dev IC50 (nM)	1.568
Ba/F3 EGFR [WT] CTGlo IC50 Count	23
Ba/F3 EGFR [A763_V764 Ins FQEA] CTGlo GMean IC50 (nM)	9.611
Ba/F3 EGFR [A763_V764 Ins FQEA] CTGlo GStd Dev IC50 (nM)	1.575
Ba/F3 EGFR [A763_V764 Ins FQEA] CTGlo IC50 Count	12
Ba/F3 EGFR [V769_D770 Ins ASV] CTGlo GMean IC50 (nM)	5.113
Ba/F3 EGFR [V769_D770 Ins ASV] CTGlo GStd Dev IC50 (nM)	1.209
Ba/F3 EGFR [V769_D770 Ins ASV] CTGlo IC50 Count	15
Ba/F3 EGFR [D770_N771 Ins SVD] CTGlo GMean IC50 (nM)	6.514
Ba/F3 EGFR [D770_N771 Ins SVD] CTGlo GStd Dev IC50 (nM)	1.25
Ba/F3 EGFR [D770_N771 Ins SVD] CTGlo IC50 Count	15
Ba/F3 EGFR [D770_N771 Ins NPG] CTGlo GMean IC50 (nM)	5.394
Ba/F3 EGFR [D770_N771 Ins NPG] CTGlo GStd Dev IC50 (nM)	1.92

Ba/F3 EGFR [D770_N771 Ins NPG] CTGlo IC50 Count	13
Ba/F3 EGFR [N771_P772 Ins N] CTGlo GMean IC50 (nM)	3.192
Ba/F3 EGFR [N771_P772 Ins N] CTGlo GStd Dev IC50 (nM)	2.676
Ba/F3 EGFR [N771_P772 Ins N] CTGlo IC50 Count	5
Ba/F3 EGFR [H773_V774 Ins PH] CTGlo GMean IC50 (nM)	2.848
Ba/F3 EGFR [H773_V774 Ins PH] CTGlo GStd Dev IC50 (nM)	2.441
Ba/F3 EGFR [H773_V774 Ins PH] CTGlo IC50 Count	5
Ba/F3 EGFR [H773_V774 Ins NPH] CTGlo GMean IC50 (nM)	8.667
Ba/F3 EGFR [H773_V774 Ins NPH] CTGlo GStd Dev IC50 (nM)	1.931
Ba/F3 EGFR [H773_V774 Ins NPH] CTGlo IC50 Count	7
Ba/F3 EGFR [N773_V774 Ins AH] CTGlo GMean IC50 (nM)	12.054
Ba/F3 EGFR [N773_V774 Ins AH] CTGlo GStd Dev IC50 (nM)	2.329
Ba/F3 EGFR [N773_V774 Ins AH] CTGlo IC50 Count	5
Ba/F3 EGFR [H773_V774 Ins H] CTGlo GMean IC50 (nM)	689.257
Ba/F3 EGFR [H773_V774 Ins H] CTGlo GStd Dev IC50 (nM)	1.066
Ba/F3 EGFR [H773_V774 Ins H] CTGlo IC50 Count	5
Ba/F3 EGFR [V774_C775 Ins HV] CTGlo GMean IC50 (nM)	24.955
Ba/F3 EGFR [V774_C775 Ins HV] CTGlo GStd Dev IC50 (nM)	1.971
Ba/F3 EGFR [V774_C775 Ins HV] CTGlo IC50 Count	5
Ba/F3 Her2 [A775_G776 ins YVMA] CTGlo GMean IC50 (nM)	3.425
Ba/F3 Her2 [A775_G776 ins YVMA] CTGlo GStd Dev IC50 (nM)	1.41
Ba/F3 Her2 [A775_G776 ins YVMA] CTGlo IC50 Count	5
Ba/F3 EGFR [Del19] CTGlo GMean IC50 (nM)	0.099
Ba/F3 EGFR [Del19] CTGlo GStd Dev IC50 (nM)	1.469
Ba/F3 EGFR [Del19] CTGlo IC50 Count	9
Ba/F3 EGFR [L858R] CTGlo GMean IC50 (nM)	0.244
Ba/F3 EGFR [L858R] CTGlo GStd Dev IC50 (nM)	1.794
Ba/F3 EGFR [L858R] CTGlo IC50 Count	9
InsFQEA Fold Selectivity vs. WT	9.769
InsFQEA Fold Selectivity vs. WT Upper Confidence Interval	13.399
InsFQEA Fold Selectivity vs. WT Lower Confidence Interval	7.123
InsASV Fold Selectivity vs. WT	18.363
InsASV Fold Selectivity vs. WT Upper Confidence Interval	22.592
InsASV Fold Selectivity vs. WT Lower Confidence Interval	14.926
InsSVD Fold Selectivity vs. WT	14.414
InsSVD Fold Selectivity vs. WT Upper Confidence Interval	17.884
InsSVD Fold Selectivity vs. WT Lower Confidence Interval	11.618

InsNPG Fold Selectivity vs. WT	17.406
InsNPG Fold Selectivity vs. WT Upper Confidence Interval	25.948
InsNPG Fold Selectivity vs. WT Lower Confidence Interval	11.676
InsN Fold Selectivity vs. WT	29.418
InsN Fold Selectivity vs. WT Upper Confidence Interval	71.084
InsN Fold Selectivity vs. WT Lower Confidence Interval	12.175
InsPH Fold Selectivity vs. WT	32.965
InsPH Fold Selectivity vs. WT Upper Confidence Interval	73.619
InsPH Fold Selectivity vs. WT Lower Confidence Interval	14.761
InsNPH Fold Selectivity vs. WT	10.834
InsNPH Fold Selectivity vs. WT Upper Confidence Interval	18.241
InsNPH Fold Selectivity vs. WT Lower Confidence Interval	6.434
InsAH Fold Selectivity vs. WT	7.789
InsAH Fold Selectivity vs. WT Upper Confidence Interval	16.713
InsAH Fold Selectivity vs. WT Lower Confidence Interval	3.63
InsH Fold Selectivity vs. WT	0.136
InsH Fold Selectivity vs. WT Upper Confidence Interval	0.165
InsH Fold Selectivity vs. WT Lower Confidence Interval	0.165
InsHV Fold Selectivity vs. WT	3.762
InsHV Fold Selectivity vs. WT Upper Confidence Interval	7.012
InsHV Fold Selectivity vs. WT Lower Confidence Interval	2.019
Her2 InsYVMA Fold Selectivity vs. WT	27.414
Her2 InsYVMA Fold Selectivity vs. WT Upper Confidence Interval	39.017
Her2 InsYVMA Fold Selectivity vs. WT Lower Confidence Interval	19.261
Del19 Fold Selectivity vs. WT	952.394
Del19 Fold Selectivity vs. WT Upper Confidence Interval	1300.085
Del19 Fold Selectivity vs. WT Lower Confidence Interval	697.688
L858R Fold Selectivity vs. WT	385.445
L858R Fold Selectivity vs. WT Upper Confidence Interval	588.881
L858R Fold Selectivity vs. WT Lower Confidence Interval	252.288

Table S8. Osimertinib Ba/F3 L858R/T790M Antiproliferative Activity and WT Selectivity

Cmpd	Ba/F3_EGFR [WT] CTGlo GMean IC50 (nM)	Ba/F3_EGFR [WT] CTGlo GStd Dev IC50 (nM)	Ba/F3_EGFR [WT] CTGlo IC50 Count	Ba/F3_EGFR [L858R/T790M] CTGlo GMean IC50 (nM)	Ba/F3_EGFR [L858R/T790M] CTGlo GStd Dev IC50 (nM)	Ba/F3_EGFR [L858R/T790M] CTGlo IC50 Count	EGFR L858R/T790M Fold Selectivity vs. WT	EGFR L858R/T790M Fold Selectivity vs. WT Upper Confidence Interval	EGFR L858R/T790M Fold Selectivity vs. WT Lower Confidence Interval
osimertinib	132.419	1.495	752	4.517	1.398	55	29.318	32.177	26.714

Table S9. Osimertinib NCI-H2073 (EGFR WT) and NCI-H1975 EGFR LR/TM Antiproliferative Activity and WT Selectivity

Cmpd	NCI-H2073_EGFR [WT] CTGlo GMean IC50 (nM)	NCI-H2073_EGFR [WT] CTGlo GStd Dev IC50 (nM)	NCI-H2073_EGFR [WT] CTGlo IC50 Count	NCI-H1975_EGFR [L858R/T790M] CTGlo GMean IC50 (nM)	NCI-H1975_EGFR [L858R/T790M] CTGlo GStd Dev IC50 (nM)	NCI-H1975_EGFR [L858R/T790M] CTGlo IC50 Count	EGFR L858R/T790M Fold Selectivity vs. WT	EGFR L858R/T790M Fold Selectivity vs. WT Upper Confidence Interval	EGFR L858R/T790M Fold Selectivity vs. WT Lower Confidence Interval
osimertinib	55.852	2.043	19	8.962	1.261	3	6.232	9.436	4.116

Table S10. Compound 53 NCI-H2073 (EGFR WT) Antiproliferative Activity

Cmpd	NCI-H2073_EGFR [WT] CTGlo GMean IC50 (nM)	NCI-H2073_EGFR [WT] CTGlo GStd Dev IC50 (nM)	NCI-H2073_EGFR [WT] CTGlo IC50 Count
53	55.221	2.011	16

Table S11. Compound 53 NCI-H2073 EGFR ex20insASV Knock-in Antiproliferative Activity and WT Selectivity

Cmpd	NCI-H2073_EGFR [ASV KI] CTGlo GMean IC50 (nM)	NCI-H2073_EGFR [ASV KI] CTGlo GStd Dev IC50 (nM)	NCI-H2073_EGFR [ASV KI] CTGlo IC50 Count	EGFR ASV KI Fold Selectivity vs. WT	EGFR ASV KI Fold Selectivity vs. WT Upper Confidence Interval	EGFR ASV KI Fold Selectivity vs. WT Lower Confidence Interval
53	10.077	1.175	3	5.480	8.078	3.717

Table S12. Compound 53 NCI-H2073 EGFR ex20insSVD Knock-in Antiproliferative Activity and WT Selectivity

Cmpd	NCI-H2073 _EGFR [SVD KI] CTGlo GMean IC50 (nM)	NCI-H2073 _EGFR [SVD KI] CTGlo GStd Dev IC50 (nM)	NCI-H2073 _EGFR [SVD KI] CTGlo IC50 Count	EGFR SVD KI Fold Selectivity vs. WT	EGFR SVD KI Fold Selectivity vs. WT Upper Confidence Interval	EGFR SVD KI Fold Selectivity vs. WT Lower Confidence Interval
53	6.042	1.279	18	9.139	13.110	6.371

Additional mouse in vivo Ba/F3 EGFR exon20insASV allograft data

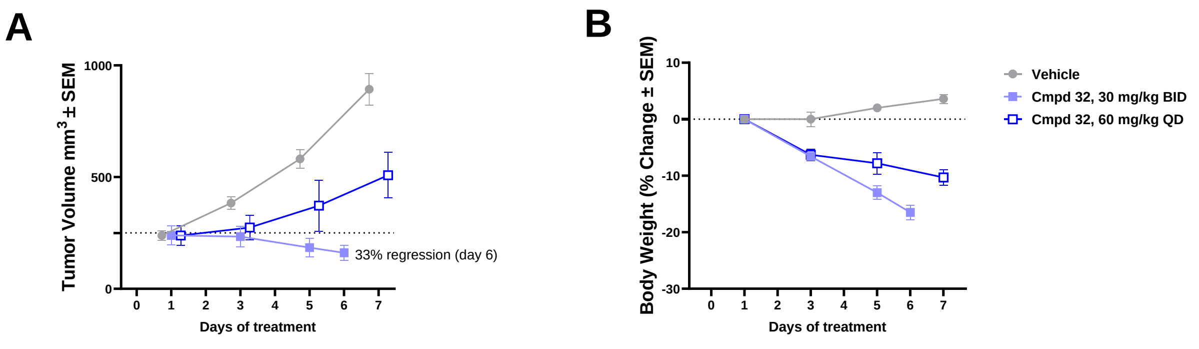

Figure S1. Reduced doses of Compound **32** dosed orally (relative to Figure 5) (A) Effect of compound **32** on Ba/F3 EGFR V769_D770 insASV tumor allografts growth in female BALB/c nude mice (QD or BID PO dosing). Data represent the mean tumor volume ± SEM (n= 6/group): the 30 mg/kg BID were $p < 0.0001$ and 60 mg/kg QD $p<0.01$ using a two-way RM ANOVA followed by Tukeys post hoc comparisons of the means. % regression calculations as noted in Supporting Information. (B) Tolerability, as reflected by body weight in female BALB/c nude mice.

Table S13. Mouse in vivo Ba/F3 EGFR exon20insASV allograft data for compounds 30, 32, 33, 34, and 35.

Cmpd	Dose (mg/kg)	% Regression	Tolerability (BWL %)
**30**	100 BID*	52%***	17%
**30**	30 BID*	20%***	12%
**32**	30 BID*	33%***	16%
**32**	60 QD	n/a (59% TGI**)	10%
**33**	200 QD	35%***	10%
**33**	100 BID	76%***	17%
**34**	100 BID*	66%***	25%
**34**	30 BID*	71%***	14%
**35**	100 BID	56%***	17%

Effect of compound **30**, **33**, **34**, and **35** on Ba/F3 EGFR V769_D770 insASV tumor allografts growth in female BALB/c nude mice (QD or BID PO dosing); *Study terminated before day 7 due to tolerability observations). % regression calculations as noted in Supporting

Information (n/a: not applicable for regression calculation, **p<0.01, ***p<0.0001 using a two-way RM ANOVA followed by Tukeys post hoc comparisons of the means). (B) Tolerability, as reflected by percent body weight loss relative to day 0 (BWL %) in female BALB/c nude mice. *Study terminated before day 7 due to tolerability observations.

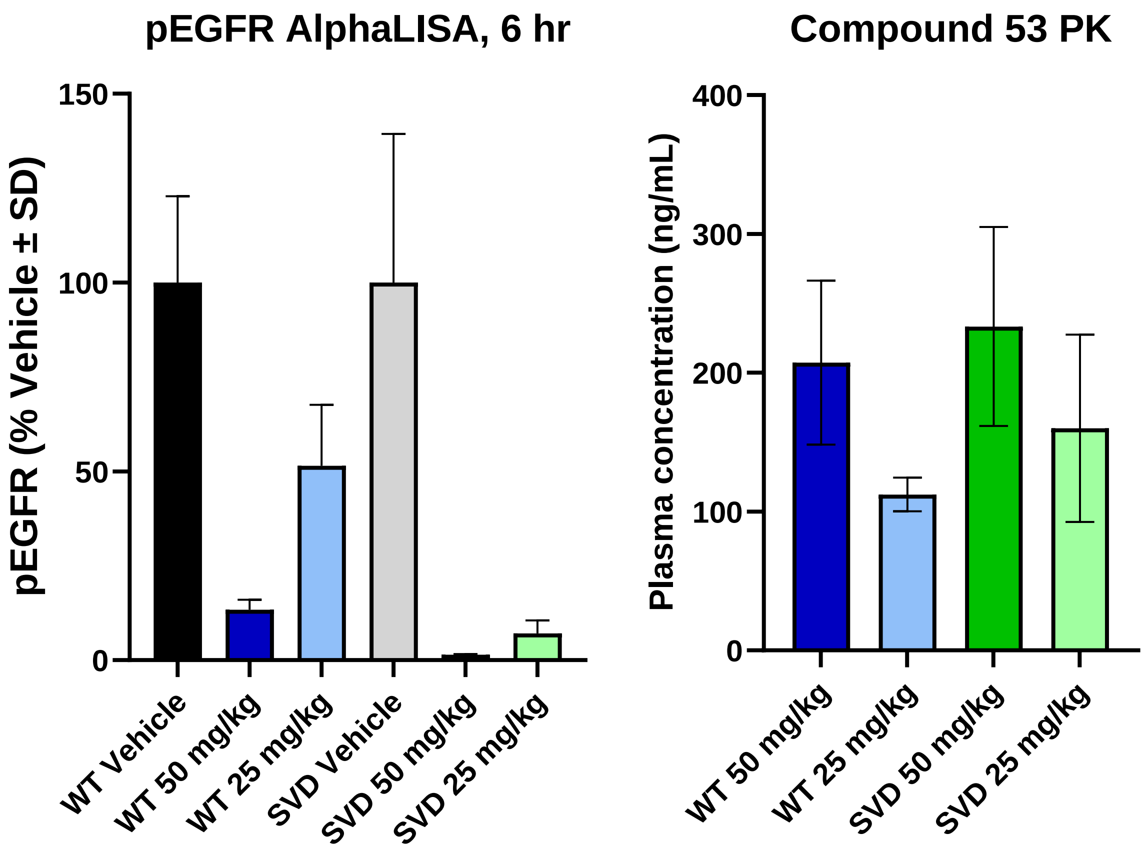

Figure S2. PK/PD analysis of Compound **53** dosed QD for 5 days and analyzed at 6 hours post-last dose in mice bearing NCI-H2073 EGFR ex20insSVD vs WT tumor xenografts. Left panel: PD determined by % of vehicle control ± SD. Right panel: Mean unbound plasma concentrations ± SD (n= 3/group).

HPLC purity data

Table S14. HPLC Purity Data for Tested Compounds

Cmpd	HPLC Purity (%)	Cmpd	HPLC Purity (%)	Cmpd	HPLC Purity (%)	Cmpd	HPLC Purity (%)
**1**	96.1	**17**	99.1	**33**	96.6	**49**	99.5
**2**	98.1	**18**	98.7	**34**	99.5	**50**	99.1

**3**	96.9	**19**	99.3	**35**	97.2	**51**	98.4
**4**	98.6	**20**	99.8	**36**	97.7	**52**	96.6
**5**	98.7	**21**	99.7	**37**	99.3	**53**	98.9
**6**	99.8	**22**	98.9	**38**	99.3	**S1**	95.3
7	97.9	**23**	99.6	**39**	98.2	**S2**	99.5
**8**	95.3	**24**	99.9	**40**	99.8	**S3**	95.4
**9**	95.2	**25**	99	**41**	95	**S4**	99
**10**	97.7	**26**	99.3	**42**	99.5	**S5**	96.8
**11**	97	**27**	99.5	**43**	98.8	**S6**	97.9
**12**	96.7	**28**	95.6	**44**	98.6	**S7**	99.2
**13**	98.5	**29**	99.2	**45**	98.5	**S8**	99.6
**14**	96	**30**	99.5	**46**	97.6	**S9**	99.4
**15**	98.4	**31**	99.4	**47**	98.2	**S10**	97.5
**16**	95	**32**	99.3	**48**	99.7		

Purity is reported as area percent at 254 nm UV absorption.

Docking calculations for compound 1

Glide docking generated binding poses for compound 1 using a αC-helix-in WT-EGFR XRC (PDB:1M17).[8] Before docking, the protein and compound **1** were prepared using the Schrodinger Maestro Suite 2020-2 Protein Preparation Wizard (Schrödinger Release 2020-2: Maestro and Protein Preparation Wizard; Epik, Schrödinger, LLC, New York, NY, 2020; Impact, Schrödinger, LLC, New York, NY; Prime, Schrödinger, LLC, New York, NY, 2020) and LigPrep default settings (Schrödinger Release 2020-2: LigPrep, Schrödinger, LLC, New York, NY, 2020).[9] This allowed for the addition of all hydrogens, assignment of residue protonation and ligand charge states with ProPKA and EPIK at pH 7.4, and optimization of protein hydrogen bond interactions.[10-12] All crystal waters were removed, and the alternate position of D855 was used in the protein preparation. Using default settings, the Glide (Schrödinger Release 2020-2: Glide, Schrödinger, LLC, New York, NY, 2020) grid was generated around the centroid of erlotinib present in the co-crystal structure. Glide docking calculations were performed with default standard precision (SP) protocol parameters but included a hydrogen bond constraint to the backbone amide nitrogen of M769 and enhanced ligand sampling.[13, 14] The protein and ligand were represented by OPLS4 forcefields for all calculations.[15]

Compound 23 WaterMap with EGFR WT and ex20insNPG protein

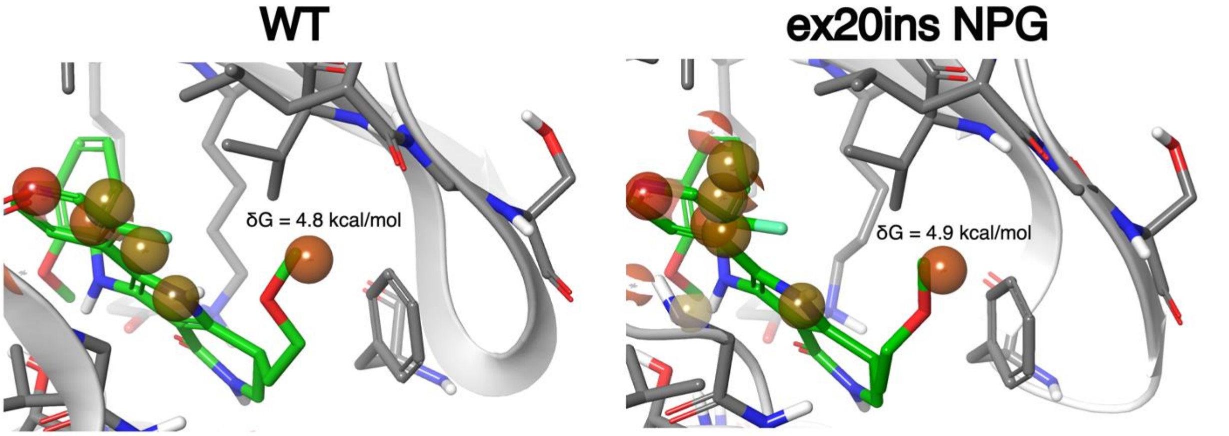

Figure S3. Water placements and free energies were predicted using the WaterMap tool in Schrödinger LLC's Maestro Suite (Schrödinger Release 2024-2: WaterMap, Schrödinger, LLC, New York, NY, 2024). The ligand was removed from the calculation but is shown in the figure to indicate where the high-energy gly-rich loop water overlays with compound **23**.[16, 17] X-ray structures used for WaterMap modeling; EGFR WT PDB: 9FRD and ex20insNPG PDB: 9FQP.

Analytical data for key compounds

Analytical Data for Compound **23**

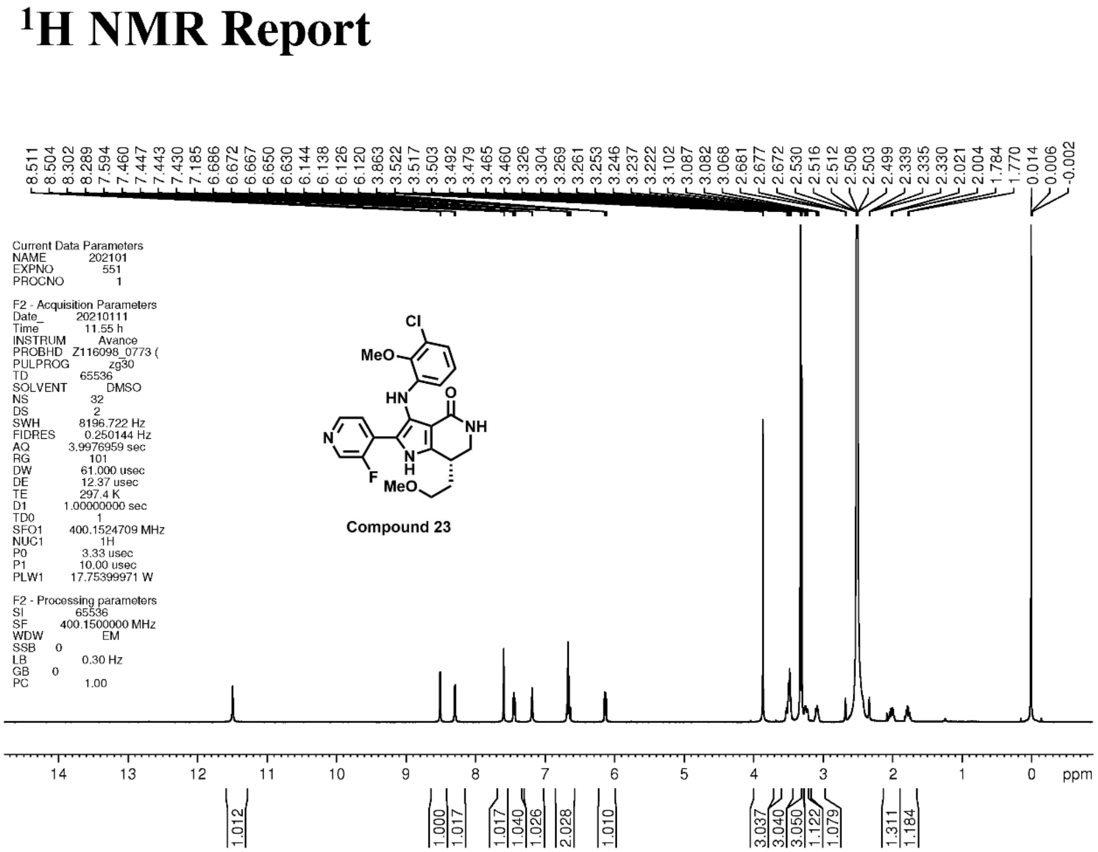

LCMS Report

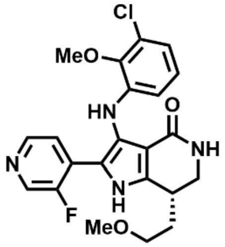

Compound 23

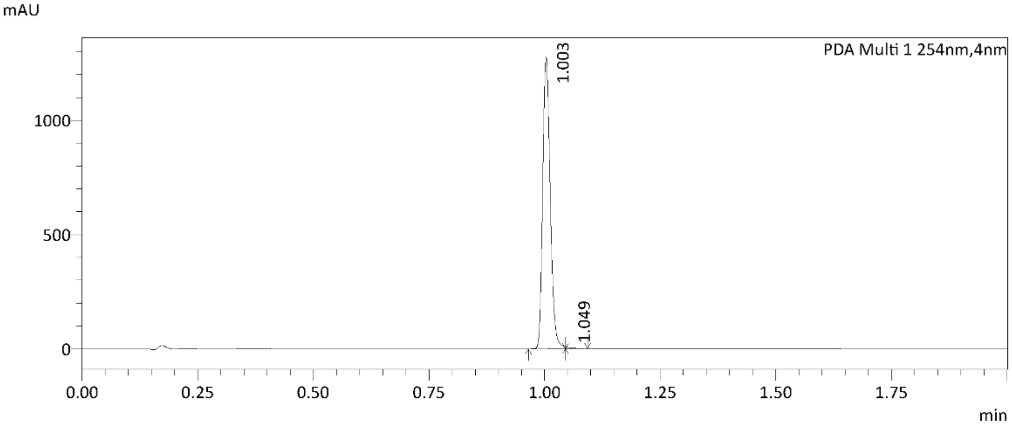

Peak Table

PDA Ch1 254nm

Peak#	Ret. Time	Height	Height%	Area	Area%
1	1.003	1253165	99.484	1429504	99.614
2	1.049	6504	0.516	5540	0.386
Total		1259669	100.000	1435044	100.000

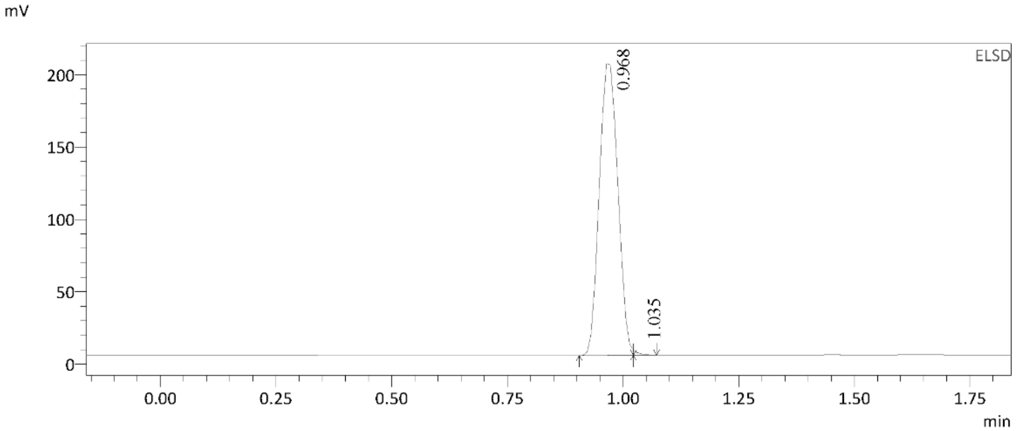

Peak Table

ELSD

Peak#	Ret. Time	Height	Height%	Area	Area%
1	0.968	201651	99.138	564576	99.712
2	1.035	1753	0.862	1632	0.288
Total		203404	100.000	566208	100.000

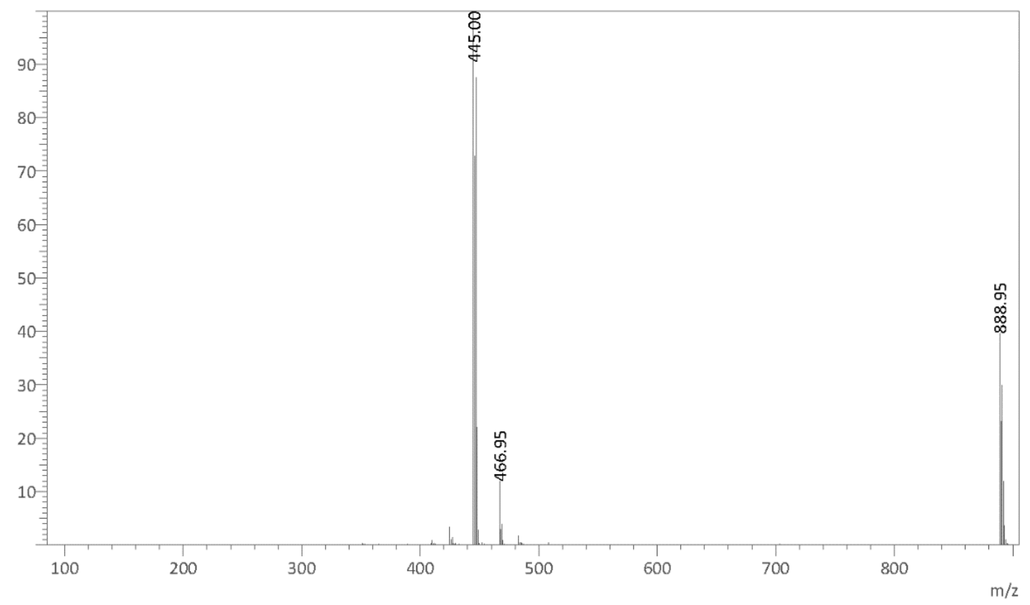
445.00
466.95
888.95
90
80
70
60
50
40
30
20
10
100
200
300
400
500
600
700
800
m/z

Analytical Data for Compound **32**

^{1}H NMR Report

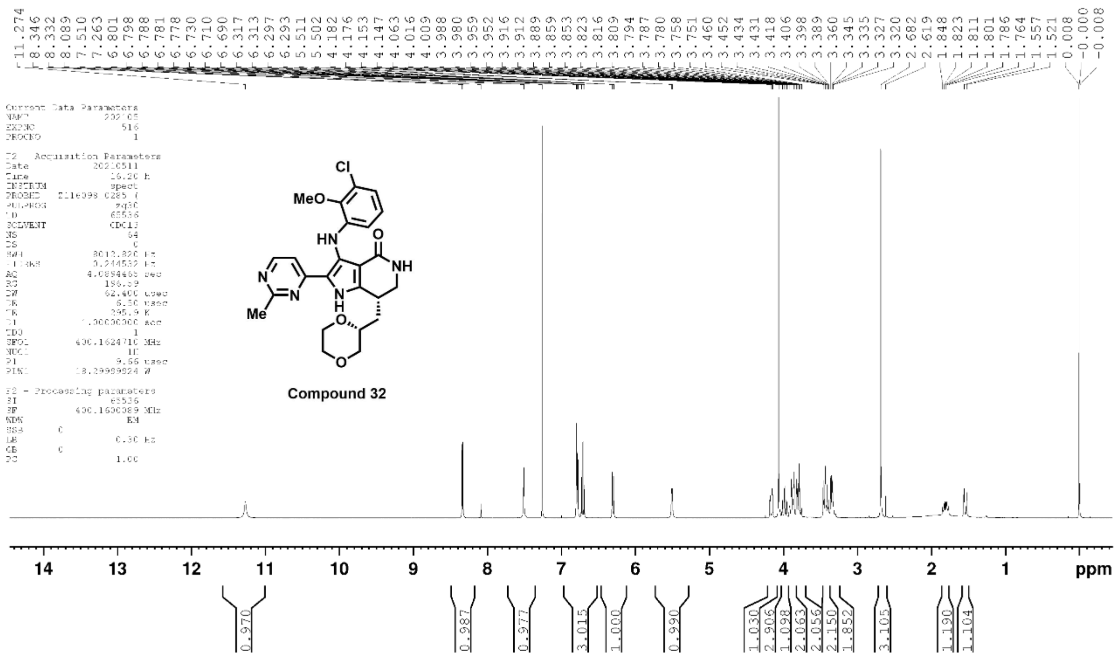

LCMS Report

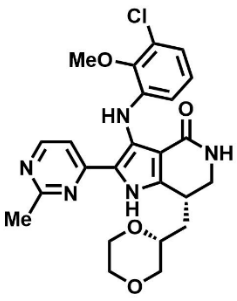

Compound 32

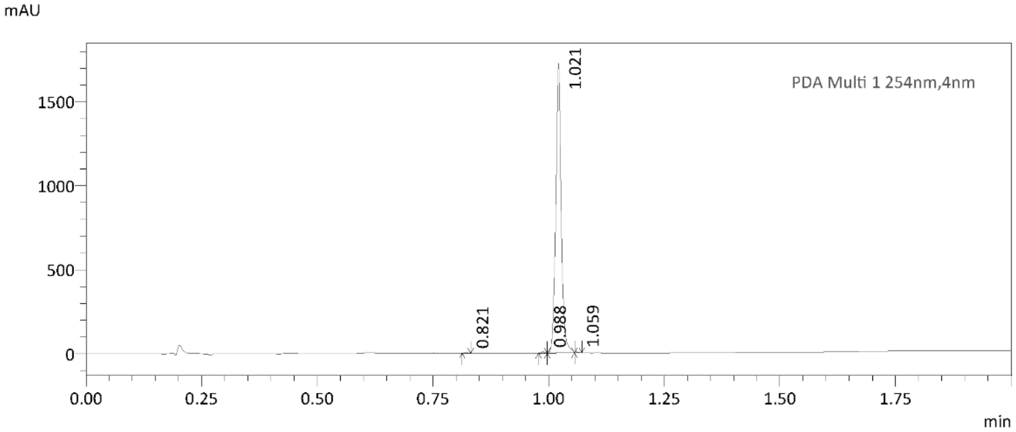

Peak Table

PDA Ch1 254nm

Peak#	Ret. Time	Height	Height%	Area	Area%
1	0.821	2686	0.158	1593	0.113
2	0.988	7622	0.450	5193	0.368
3	1.021	1678553	99.012	1403327	99.335
4	1.059	6436	0.380	2614	0.185
Total		1695297	100.000	1412727	100.000

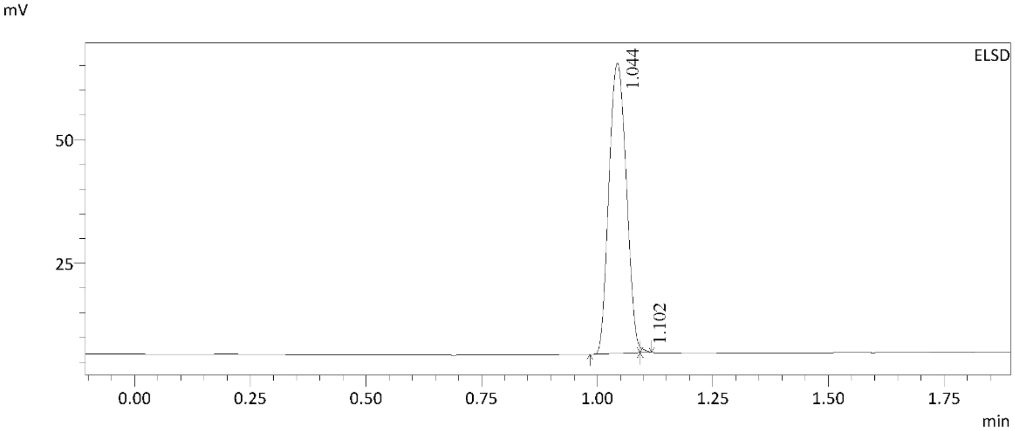

Peak Table

ELSD

Peak#	Ret. Time	Height	Height%	Area	Area%
1	1.044	56941	98.788	153474	99.656
2	1.102	699	1.212	529	0.344
Total		57640	100.000	154003	100.000

Mass Spectrum

Line#:1 R.Time: 0.995 (Scan#:15) MassPeaks: 391
Spectrum Mode: Single 0.995 (135) BasePeak: 484.30 (2346046)
BG Mode: Averaged 0.895 - 1.095 (123-147) Segment 1 - Event 1

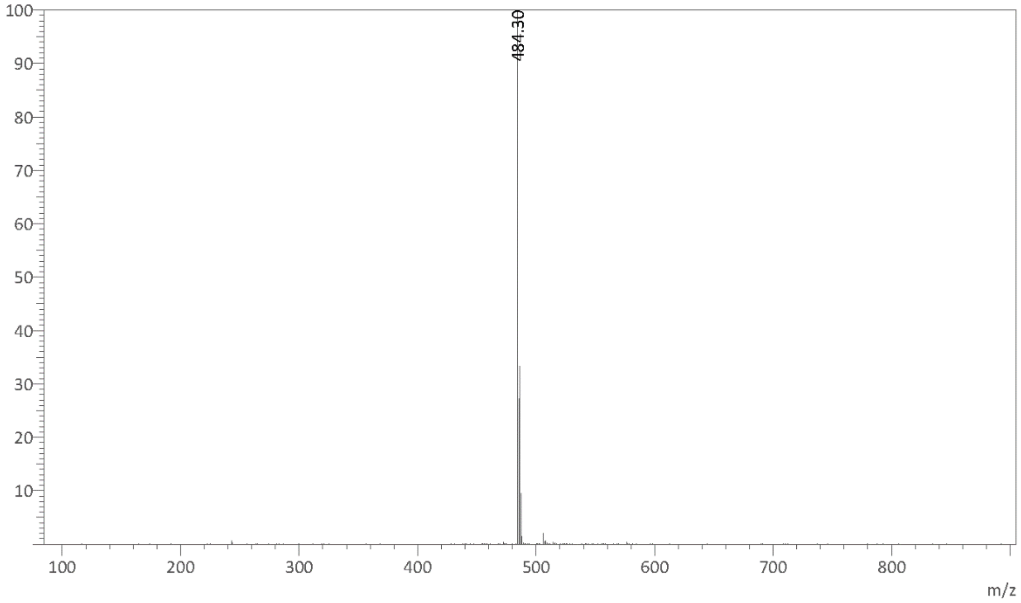

Analytical Data for Compound **39**

^{1}H NMR Report

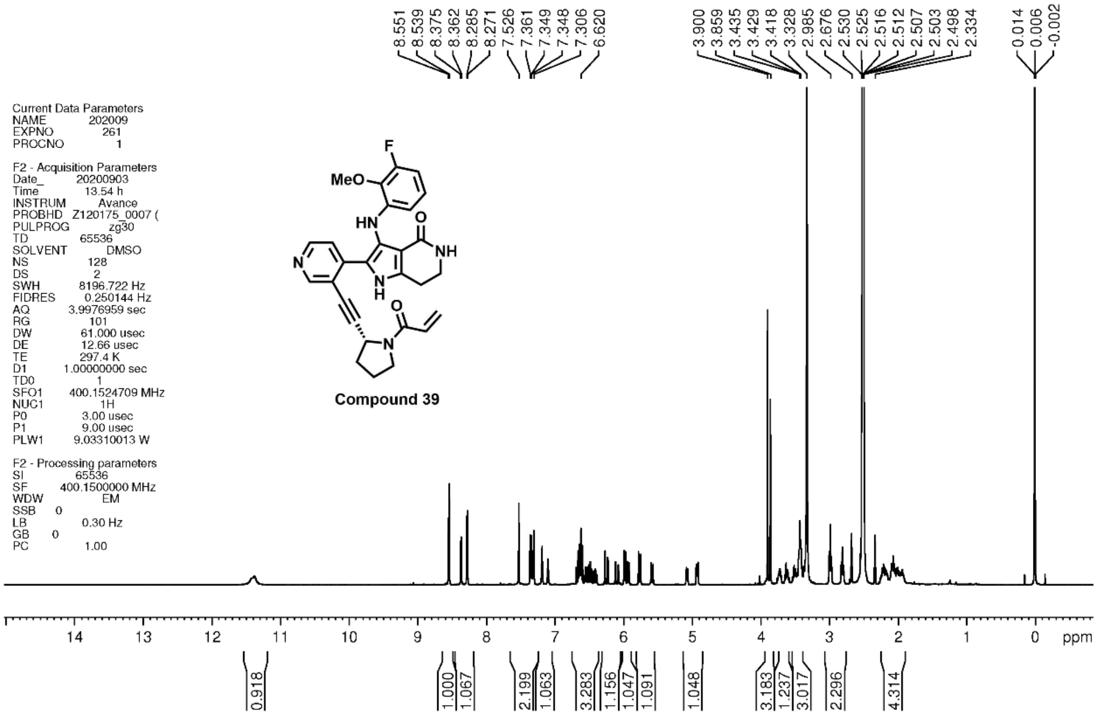

LCMS Report

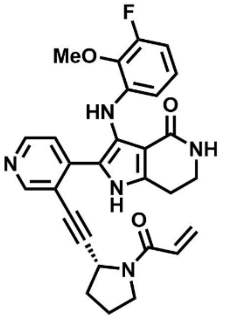

Compound 39

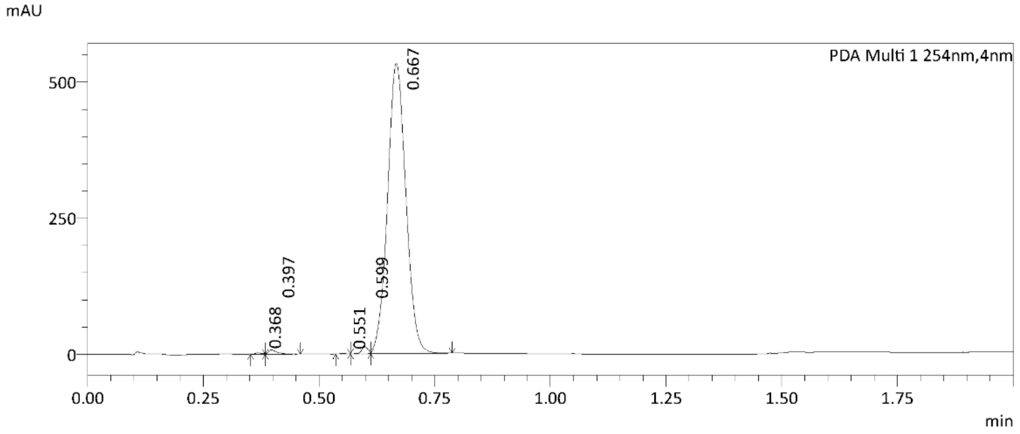

Peak Table

PDA Ch1 254nm

Peak#	Ret. Time	Height	Height%	Area	Area%
1	0.368	2281	0.411	2634	0.177
2	0.397	7718	1.392	10211	0.686
3	0.551	1158	0.209	981	0.066
4	0.599	11975	2.160	12767	0.858
5	0.667	531174	95.827	1462017	98.214
[illegible]		[illegible]	[illegible]	[illegible]	[illegible]

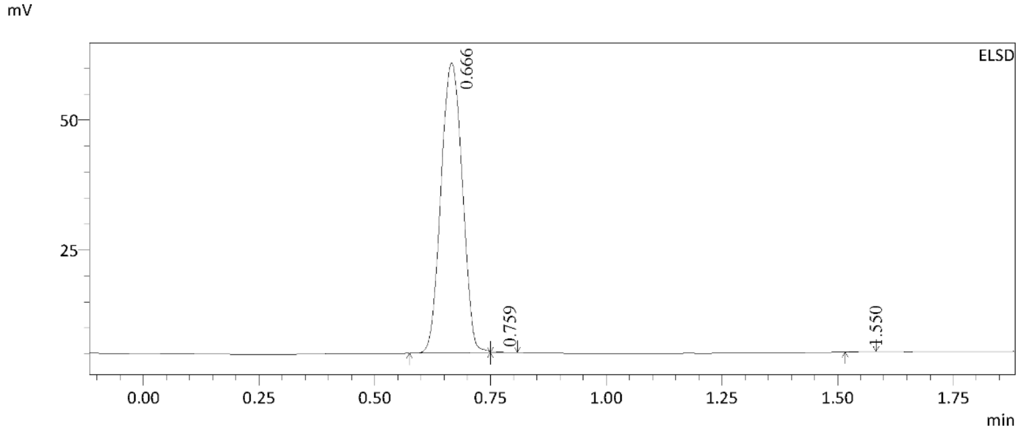

Peak Table

ELSD

Peak#	Ret. Time	Height	Height%	Area	Area%
1	0.666	55008	99.505	179300	99.761
2	0.759	200	0.362	250	0.139
3	1.550	74	0.133	179	0.099
Total		55282	100.000	179729	100.000

Mass Spectrum

Line#:1 R.Time: 0.670 (Scan#:80) MassPeaks: 395
Spectrum Mode: Single 0.670 (85) BasePeak: 500.20 (724286)
BG Mode: Peak Start 0.628(80) Segment 1 - Event 1

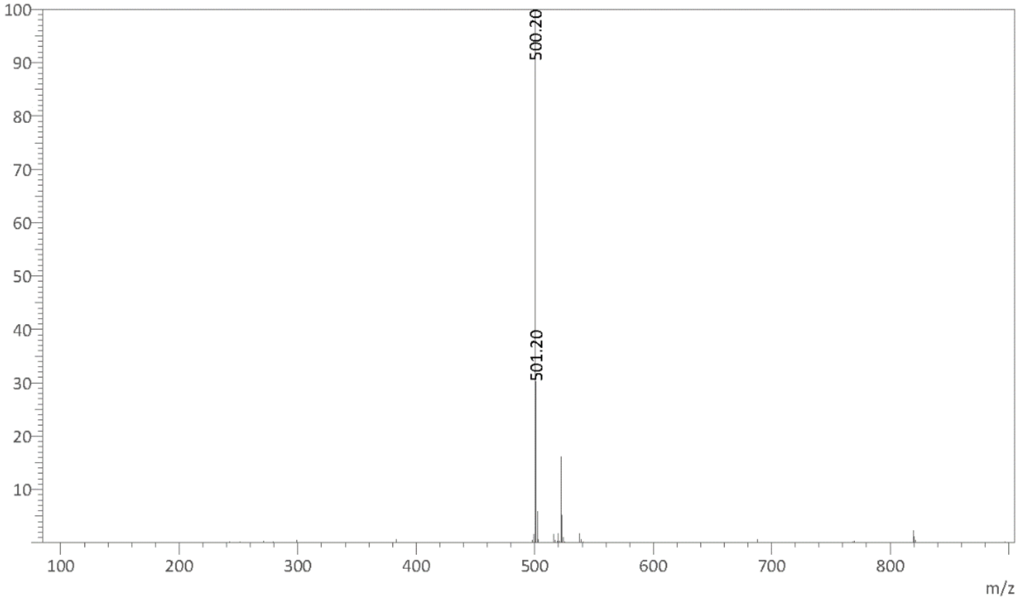

Analytical Data for Compound **53** (STX-721)

^{1}H NMR Report

Bruker_CD-A_400MHz

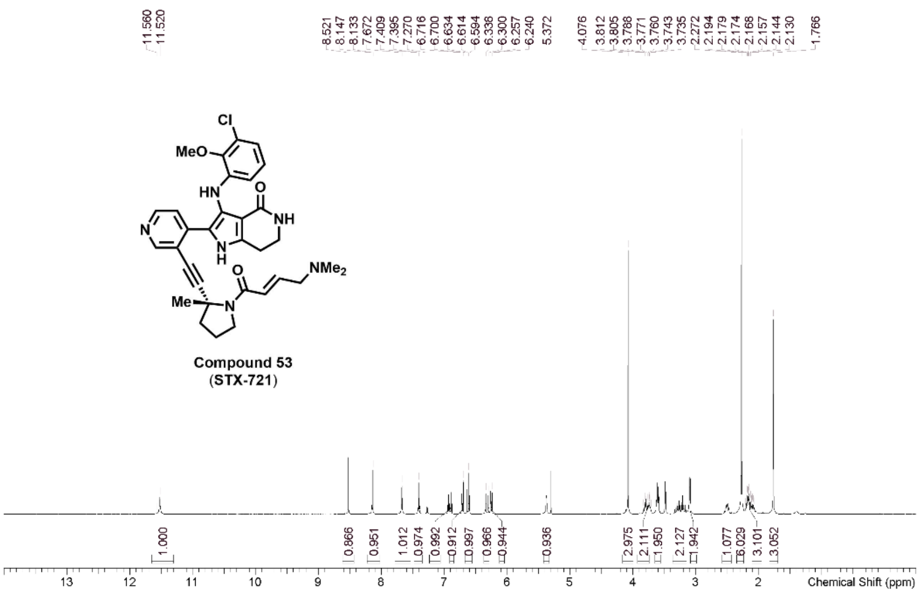

Acquisition Time (sec)	1.9999
Frequency (MHz)	400.1700
Nucleus	1H
Number of Transients	8
Origin	Avance
Original Points Count	16393
Owner	nmrsu
Points Count	65536
Pulse Sequence	zg30
Receiver Gain	101.00
SW(cyclical) (Hz)	8196.72
Solvent	CHLOROFORM-d
Spectrum Offset (Hz)	2465.2842
Spectrum Type	standard
Sweep Width (Hz)	8196.60
Temperature (degree C)	25.150

LCMS Report

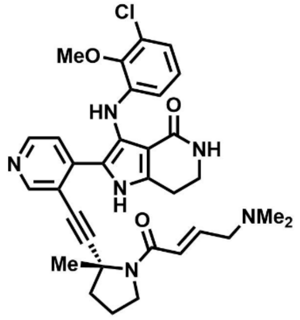

Compound 53
(STX-721)

Injection Vol : 1uL

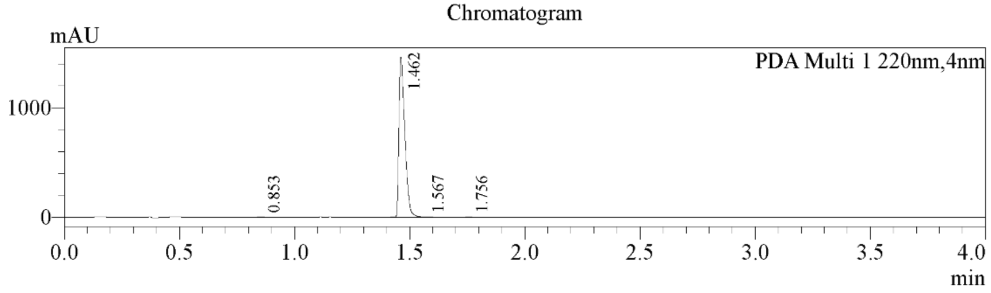

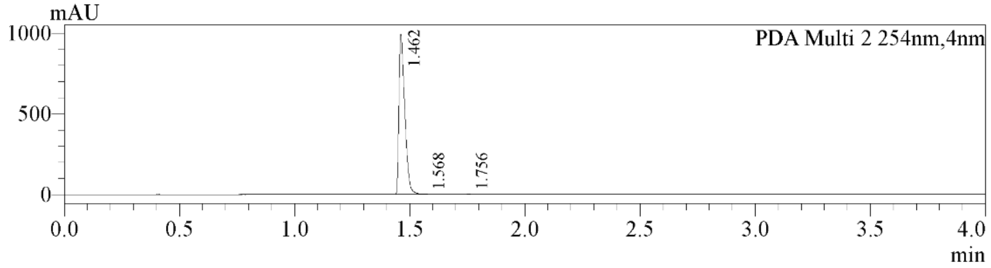

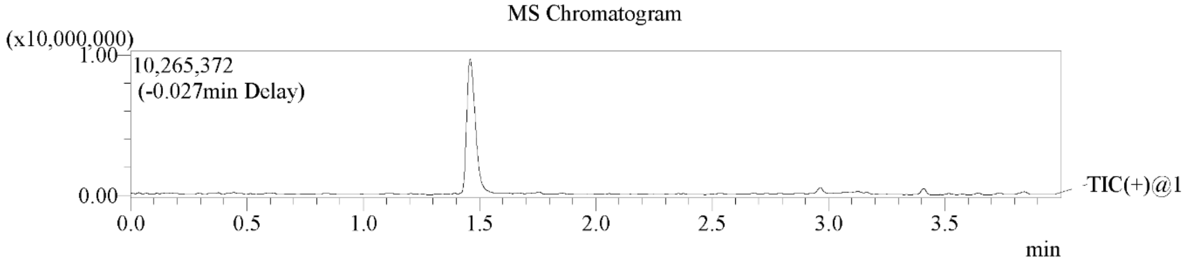

Integration Result

ELSD

PDA Ch1 220nm

Peak#	Ret. Time	Height	Height%	USP Width	Area	Area%
1	0.853	2878	0.195	0.032	3517	0.134
2	1.462	1464440	99.426	0.048	2604735	99.545
3	1.567	2923	0.198	0.819	3749	0.143
4	1.756	2660	0.181	0.047	4633	0.177

PDA Ch2 254nm

Peak#	Ret. Time	Height	Height%	USP Width	Area	Area%
1	1.462	992378	99.656	0.047	1747930	99.706
2	1.568	1759	0.177	0.597	2344	0.134
3	1.756	1663	0.167	0.046	2817	0.161

Mass Spectrum

RetTime: 1.462 DateFile: D:\Data\2022\2203\220317\EC7088-10-P1F1.lcd

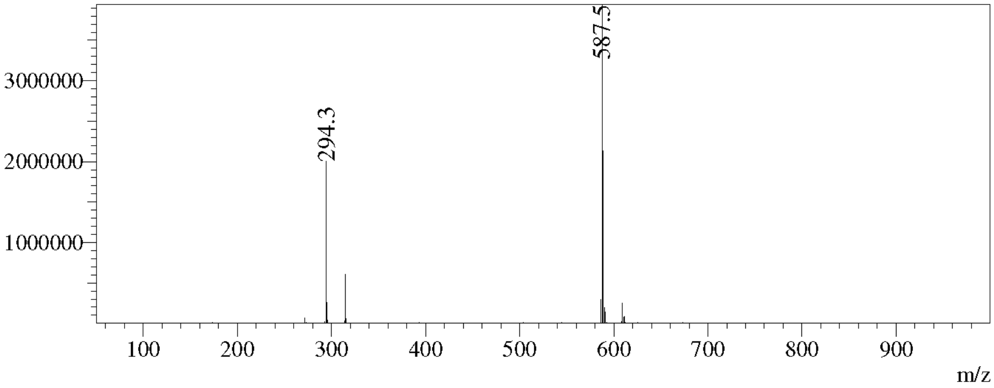

HPLC Report

Injection Vol : 1uL

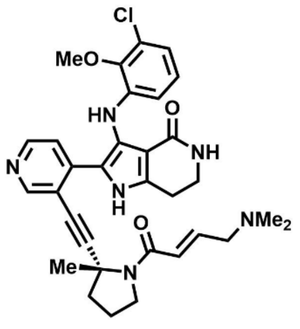

Compound 53
(STX-721)

Chromatogram

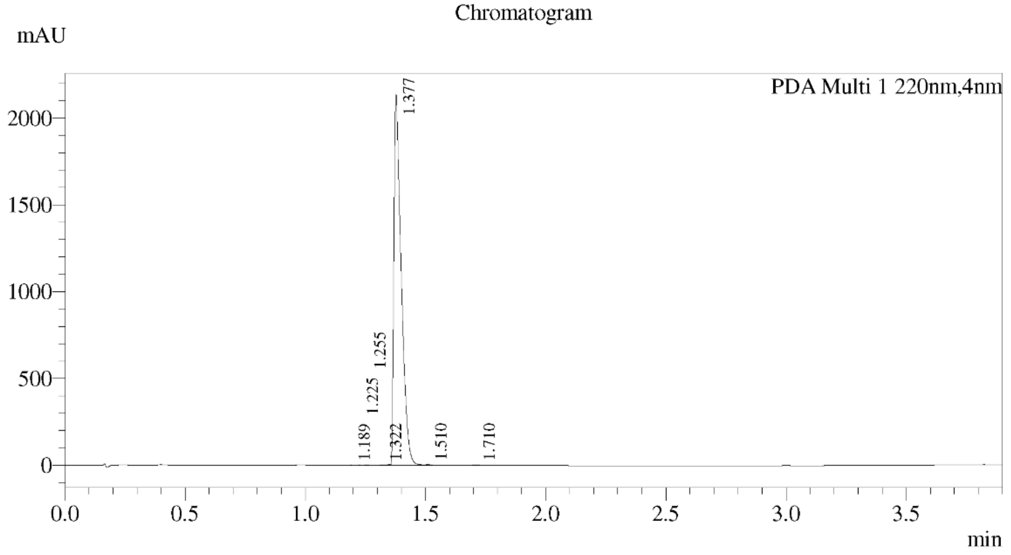

PDA Ch1 220nm

Peak#	Ret. Time	USP Width	Height	Height%	Area	Area%
1	1.189	0.026	1447	0.067	1555	0.034
2	1.225	0.034	1446	0.067	1666	0.037
3	1.255	0.029	3162	0.147	3486	0.076
4	1.322	0.030	3962	0.184	4888	0.107
5	1.377	0.057	2136865	99.134	4529511	99.374
6	1.510	0.080	4809	0.223	8514	0.187
7	1.710	0.057	3835	0.178	8436	0.185
Total			2155526	100.000	4558057	100.000

HPLC Report

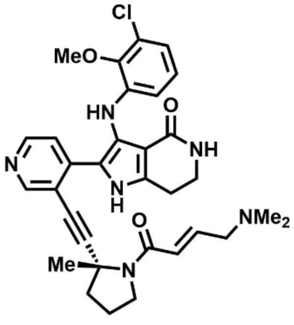

53
(STX-721)

Injection Vol : 1uL

Chromatogram

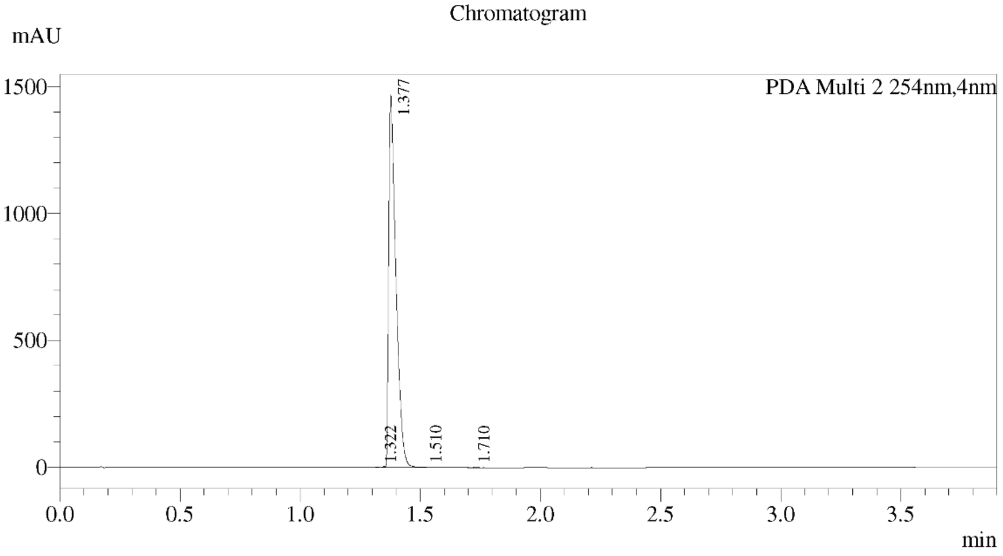

PDA Ch2 254nm

Peak#	Ret. Time	USP Width	Height	Height%	Area	Area%
1	1.322	0.030	2409	0.163	3040	0.099
2	1.377	0.056	1467387	99.463	3070279	99.551
3	1.510	0.082	3097	0.210	5507	0.179
4	1.710	0.056	2422	0.164	5307	0.172
Total			1475316	100.000	3084133	100.000

X-ray crystallographic data

X-ray co-crystal structures of compounds 23 & 39

Table S15 X-ray Crystallographic Data for Compounds 23 & 39

	Compound **39** with EGFR ex20insNPG	Compound **23** with EGFR ex20insNPG	Compound **23** with EGFR WT
Accession Code	9FQS	9FQP	9FRD
Resolution (Å)	1.78 (2.01-1.78)	2.50 (2.61-2.50)	2.06 (2.09-2.06)
Space group	$C222_1$	$C222_1$	I23
Unit cell			
a, b, c / Å	40.1, 106.5, 175.8	40.8, 107.2, 175.4	145.2; 145.2; 145.2
α, β, γ / °	90.0, 90.0, 90.0	90.0, 90.0, 90.0	90.0, 90.0, 90.0
Number of reflections			
Total	367748 (33144)	39852 (4262)	802325 (41004)
Unique	27685 (2768)	12861 (1286)	31693 (1561)
R_{meas}	0.127 (1.894)	0.088 (1.035)	0.093 (2.986)
R_{pim}	0.047 (0.748)	0.049 (0.600)	0.018 (0.582)
$CC_{1/2}$	0.998 (0.620)	0.979 (0.568)	1.00 (0.559)
Multiplicity	13.3 (12.0)	3.1 (3.3)	25 (26.3)
Completeness (%)			
Spherical	75.8 (25.6)	92.9 (77.3)	100.0 (100.0)
Ellipsoidal	91.6 (58.2)	92.9 (77.4)	
Mean I/sigma(I)	12.7 (1.6)	8.5 (1.5)	22.3 (1.4)
Refinement:			
Resolution range (Å)	34.5-1.78 (1.89-1.78)	33.9-2.50 (2.54-2.50)	72.69-2.06
R-work	0.197 (0.275)	0.243 (0.314)	0.168 (0.267)
R-free	0.238 (0.320)	0.275 (0.359)	0.197 (0.271)
Number of non-hydrogen atoms	2770	2463	2482
r.m.s.d. bonds (Å)	0.010	0.008	0.009
r.m.s.d. angles (°)	1.02	0.88	1.57
Ramachandran plot (%)			
Favored	98.0	95.8	97.1
Allowed	2.0	4.0	3.0
Outlier	0.0	0.0	0.0
Clashscore	1.56	0.00	0.79

*Values in parentheses are for highest-resolution shell.

X-ray structure determination of compound 32

Single yellow plate-shaped crystals of Compound **32** recrystallized from a mixture of DCM / MeOH and pentane by solvent layering. A suitable crystal with dimensions 0.27 × 0.20 × 0.04 mm^3 was selected and mounted on a nylon loop with paratone oil on a XtaLAB Synergy, Dualflex, HyPix diffractometer. The crystal was kept at a steady T = 99.9(3) K during data collection. The structure was solved with the ShelXT[18, 19] solution program using dual methods and by using Olex2 as the graphical interface. The model was refined with ShelXL using full matrix least squares minimisation on F^2. The Flack parameter was refined to 0.019(6). Determination of absolute structure using Bayesian statistics on Bijvoet differences using the Olex2 results in 0.031(5). These values confirm the absolute stereochemistry unambiguously to be S at C6 and R at C14. The compound was desposited in the CCDC with deposition number 2362348.

Figure S4. Drawing of X-ray structure of Compound **32**. The disordered solvent is not shown for simplicity.

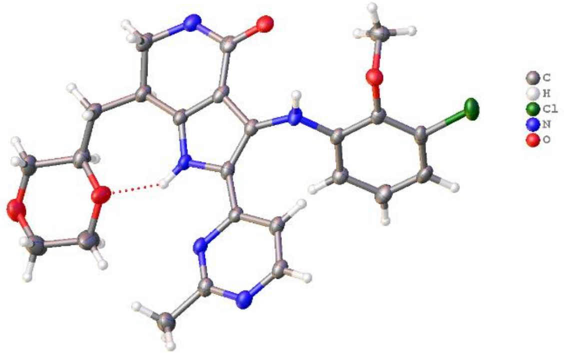

Table S16. Crystal Data and Structure Refinement for Compound 32

Formula	$C_{26}H_{27.5}ClF_3N_5O_7$
$D_{calc.}$/ g cm^{-3}	1.330
μ/mm^{-1}	1.705
Formula Weight	614.48
Colour	yellow
Shape	plate-shaped
Size/mm^3	0.27×0.20×0.04
T/K	99.9(3)
Crystal System	monoclinic
Flack Parameter	0.019(6)
Hooft Parameter	0.031(5)
Space Group	$P2_1$
a/Å	12.32112(11)
b/Å	17.92686(16)
c/Å	14.14295(10)
α/°	90
β/°	100.7407(8)
γ/°	90

V/Å^3	3069.15(4)
Z	4
Z'	2
Wavelength/Å	1.54184
Radiation type	Cu K$_\alpha$
Θ_{min}/°	3.180
Θ_{max}/°	80.439
Measured Refl's.	44949
Indep't Refl's	12783
Refl's I≥2 σ(I)	12224
R_{int}	0.0421
Parameters	723
Restraints	185
Largest Peak	1.806
Deepest Hole	-1.522
GooF	1.040
wR_2 (all data)	0.2036
wR_2	0.1996
R_1 (all data)	0.0748
R_1	0.0721

X-ray structure determination of STX-721

Single yellow block-shaped crystals of **STX-721** recrystallized from a mixture of DCM and toluene by slow evaporation. A suitable crystal with dimensions 0.23 × 0.15 × 0.14 mm^3 was selected and mounted on a nylon loop with paratone oil on a XtaLAB Synergy, Dualflex, HyPix diffractometer. The crystal was kept at a steady T = 100.00(10) K during data collection. The structure was solved with the ShelXT[18, 19] solution program using iterative methods and by using Olex2 1.5 as the graphical interface. The model was refined with **ShelXL** 2018/3 using full matrix least squares minimization on $\boldsymbol{F}^2$. The Flack parameter was refined to 0.022(12). Determination of absolute structure using Bayesian statistics on Bijvoet differences using the Olex2 results in 0.022(12). Absolute stereochemistry was unambiguously determined to be R at C4. The compound was deposited in the CCDC with deposition number 2362347.

Figure S5. Drawing of X-ray structure of **STX-721**. The disordered solvent is not shown for simplicity.

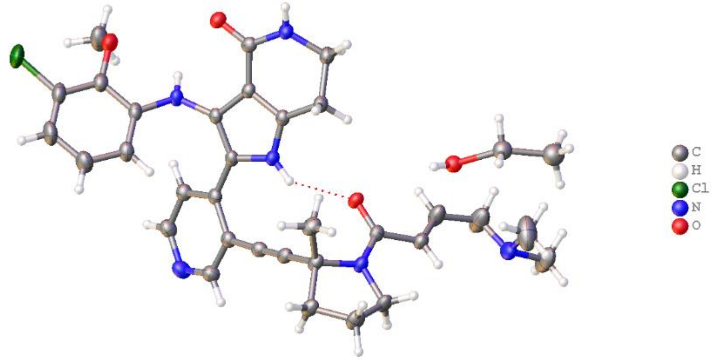

Table S17. Crystal Data and Structure Refinement for Compound 53 (STX-721).

Formula	$C_{33}H_{38}ClN_6O_{3.5}$
$D_{calc.}$/ g cm^{-3}	1.278
μ/mm^{-1}	1.430
Formula Weight	610.14
Color	yellow
Shape	block-shaped
Size/mm^3	0.23×0.15×0.14
T/K	100.00(10)
Crystal System	tetragonal
Flack Parameter	0.022(12)
Hooft Parameter	0.022(12)
Space Group	$P4_12_12$
a/Å	13.10468(5)
b/Å	13.10468(5)
c/Å	36.9221(3)
α/°	90
β/°	90
γ/°	90
V/Å^3	6340.73(7)
Z	8
Z'	1
Wavelength/Å	1.54184
Radiation type	Cu Kα
Θ_{min}/°	3.579
Θ_{max}/°	77.219
Measured Refl's.	79142

Indep't Refl's	6663
Refl's I≥2 σ(I)	6357
R_{int}	0.0707
Parameters	397
Restraints	0
Largest Peak	0.866
Deepest Hole	-0.357
GooF	1.058
wR_2 (all data)	0.1433
wR_2	0.1417
R_1 (all data)	0.0551
R_1	0.0533

References

1. Gajiwala, K. S.; Feng, J.; Ferre, R.; Ryan, K.; Brodsky, O.; Weinrich, S.; Kath, J. C.; Stewart, A., Insights into the aberrant activity of mutant EGFR kinase domain and drug recognition. *Structure* **2013,** *21* (2), 209-219.
2. Lukovic, E.; Gonzalez-Vera, J. A.; Imperiali, B., Recognition-domain focused chemosensors: versatile and efficient reporters of protein kinase activity. *J Am Chem Soc* **2008,** *130* (38), 12821-12827.
3. Shults, M. D.; Imperiali, B., Versatile fluorescence probes of protein kinase activity. *J Am Chem Soc* **2003,** *125* (47), 14248-14249.
4. Kuzmic, P., Program DYNAFIT for the analysis of enzyme kinetic data: application to HIV proteinase. *Anal Biochem* **1996,** *237* (2), 260-273.
5. Kuzmic, P., DynaFit--a software package for enzymology. *Methods Enzymol* **2009,** *467*, 247-280.
6. Pagliarini, R. A.; Henderson, J. A.; Milgram, B. C.; Borrelli, D. R.; Brooijmans, N.; Hilbert, B. J.; Huff, M. R.; Ito, T.; Kryukov, G. V.; Ladd, B.; Martin, B. R.; Motiwala, H.; O'Hearn, E.; Tsai, C.-F.; Wang, W.; Hata, A.; Bellier, J.; Kuzmic, P.; Guzman-Perez, A.; Jackson, E. L.; Stuart, D. D., STX-721, a Covalent EGFR/HER2 Exon 20 Inhibitor, Takes Advantage of Exon 20 Mutant Dynamic Protein States and Achieves Unique Mutant-Selectivity Across Human Cancer Models. *Clin. Cancer Res., Manuscript Submitted*.
7. Siegel, S.; Siegel, F.; Schulze, V.; Berger, M.; Graham, K.; Klar, U.; Knut, E.; Sülzle, D.; Bömer, U.; Korr, D.; Petersen, K.; Mönning, U.; Eberspächer, U.; Moosmayer, D.; Meyerson, M.; Greulich, H.; Kaplan, B.; Harb, H. Y.; Dinh, P. M. 4H-Pyrrolo[3,2-c]pyridin-4-one Derivatives. WO 2019081486, May 2, 2019.
8. Stamos, J.; Sliwkowski, M. X.; Eigenbrot, C., Structure of the epidermal growth factor receptor kinase domain alone and in complex with a 4-anilinoquinazoline inhibitor. *J Biol Chem* **2002,** *277* (48), 46265-46272.

9. Sastry, G. M.; Adzhigirey, M.; Day, T.; Annabhimoju, R.; Sherman, W., Protein and ligand preparation: parameters, protocols, and influence on virtual screening enrichments. *J Comput Aided Mol Des* **2013,** *27* (3), 221-234.
10. Sondergaard, C. R.; Olsson, M. H.; Rostkowski, M.; Jensen, J. H., Improved Treatment of Ligands and Coupling Effects in Empirical Calculation and Rationalization of pKa Values. *J Chem Theory Comput* **2011,** *7* (7), 2284-2295.
11. Olsson, M. H.; Sondergaard, C. R.; Rostkowski, M.; Jensen, J. H., PROPKA3: Consistent Treatment of Internal and Surface Residues in Empirical pKa Predictions. *J Chem Theory Comput* **2011,** *7* (2), 525-537.
12. Johnston, R. C.; Yao, K.; Kaplan, Z.; Chelliah, M.; Leswing, K.; Seekins, S.; Watts, S.; Calkins, D.; Chief Elk, J.; Jerome, S. V.; Repasky, M. P.; Shelley, J. C., Epik: pK(a) and Protonation State Prediction through Machine Learning. *J Chem Theory Comput* **2023,** *19* (8), 2380-2388.
13. Friesner, R. A.; Banks, J. L.; Murphy, R. B.; Halgren, T. A.; Klicic, J. J.; Mainz, D. T.; Repasky, M. P.; Knoll, E. H.; Shelley, M.; Perry, J. K.; Shaw, D. E.; Francis, P.; Shenkin, P. S., Glide: a new approach for rapid, accurate docking and scoring. 1. Method and assessment of docking accuracy. *J Med Chem* **2004,** *47* (7), 1739-1749.
14. Halgren, T. A.; Murphy, R. B.; Friesner, R. A.; Beard, H. S.; Frye, L. L.; Pollard, W. T.; Banks, J. L., Glide: a new approach for rapid, accurate docking and scoring. 2. Enrichment factors in database screening. *J Med Chem* **2004,** *47* (7), 1750-1759.
15. Lu, C.; Wu, C.; Ghoreishi, D.; Chen, W.; Wang, L.; Damm, W.; Ross, G. A.; Dahlgren, M. K.; Russell, E.; Von Bargen, C. D.; Abel, R.; Friesner, R. A.; Harder, E. D., OPLS4: Improving Force Field Accuracy on Challenging Regimes of Chemical Space. *J Chem Theory Comput* **2021,** *17* (7), 4291-4300.
16. Abel, R.; Young, T.; Farid, R.; Berne, B. J.; Friesner, R. A., Role of the active-site solvent in the thermodynamics of factor Xa ligand binding. *J Am Chem Soc* **2008,** *130* (9), 2817-2831.
17. Young, T.; Abel, R.; Kim, B.; Berne, B. J.; Friesner, R. A., Motifs for molecular recognition exploiting hydrophobic enclosure in protein-ligand binding. *Proc Natl Acad Sci U S A* **2007,** *104* (3), 808-813.
18. Sheldrick, G. M., Crystal structure refinement with SHELXL. *Acta Crystallogr C Struct Chem* **2015,** *71* (Pt 1), 3-8.
19. Sheldrick, G. M., SHELXT - integrated space-group and crystal-structure determination. *Acta Crystallogr A Found Adv* **2015,** *71* (Pt 1), 3-8.
